# Upgrading pillar[*n*]arenes to reversible photocontrolled self-folding hosts for photoswitchable guest uptake/release and self-assembly

**DOI:** 10.1039/d5sc09696k

**Published:** 2026-01-26

**Authors:** Ao-Ran Liu, Wen-Ping Gong, Qi Jin, Tian-Guang Zhan, Yu Hai, Li-Juan Liu, Kang-Da Zhang

**Affiliations:** a Key Laboratory of the Ministry of Education for Advanced Catalysis Materials, College of Chemistry and Materials Science, Zhejiang Normal University 688 Yingbin Road Jinhua 321004 China tgzhan@zjnu.cn haiyuyu@zjnu.edu.cn Kangda.Zhang@zjnu.cn

## Abstract

Photoresponsive macrocycles can serve as versatile supramolecular platforms for exploring remote-controllable self-assembly systems and materials. However, reconciling excellent host–guest properties with robust photocontrollable capabilities persists as a formidable yet pivotal challenge in the design and construction of photoresponsive macrocycles. Herein, we demonstrate a photocontrolled self-folding strategy to obtain a new class of photoswitchable macrocycle, AzoP[5/6]A, by directly introducing an azobenzene (azo) unit onto the pillararene macrocycle scaffold, which does not impair the guest binding ability yet allows for significant ON/OFF photoswitching. It transpires that when the azo unit adopts the *E*-configuration, these AzoP[5/6]A feature a guest-accessible cavity and exhibit comparable guest binding ability to that of the pristine alkylated pillararenes. However, as the azo unit undergoes photoisomerization from *E* to *Z*, these AzoP[5/6]A fold into configurations with blocked cavities self-filled with partial *Z*-azo modules, which causes a dramatic reduction of the binding affinity towards the guest molecules by up to 1 × 10^4^-fold, resulting in efficient release of guests from the macrocycle cavity. Furthermore, in the presence or absence of guests, these azopillararenes could all show high bidirectional *E* ⇆ *Z* photoconversion (up to ≥ 95%). These unique properties further benefit the fabrication of host–guest supramolecular polymeric networks featuring photoswitchable self-assembly behavior.

## Abstract 2

Introducing an azobenzene unit onto pillararene scaffold generates a new class of photocontrolled self-folding hosts with marked ON/OFF photoswitching performance, which offers a promising tool for photocontrolled supramolecular self-assembly.

## Graphical abstract

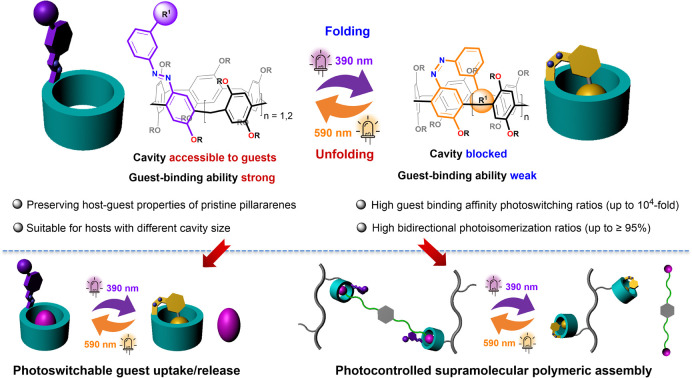

## Introduction

Macrocyclic molecules constitute a class of fundamentally important molecular building blocks, whose related research has long been a central focus in supramolecular chemistry.^1–17^ Beyond exploring new host macrocycles with highly selective recognition and strong guest binding affinities,^18–27^ another key aspect concerning macrocyclic chemistry is to introduce dynamic adaptivity into macrocycles to allow them to modulate their guest binding abilities and behaviors in response to external stimuli.^13,28–34^ Such self-modulation ability enables the stimuli-responsive macrocyclic hosts to undergo controllable release/uptake of guests, which permits these macrocycles to serve as essential platforms for developing molecular machines,^28,35,36^ biomedicines,^6,37,38^ switchable catalysts,^39,40^ absorption and separation technologies,^41,42^ and dynamic supramolecular materials.^14,31,32,48,49^

Of the various external stimuli, light stands out as an ideal source due to its inherent features: high spatiotemporal precision, non-invasiveness, cleanliness, and digital controllability.^43–47^ Benefiting from this, the guest release/uptake of photoresponsive macrocyclic hosts can be manipulated in a precise and remote-controllable manner, which is exceptionally advantageous in achieving on-demand control of the properties and functional output of host–guest systems.^48–50^ So far, the strategies for constructing host macrocycles with significant photocontrollability generally involve the incorporation of photoisomerization units such as azobenzenes,^41,51–57^ stiff stilbenes^42,58–60^ or diarylethenes^61,62^ into the macrocyclic molecular backbone. Accordingly, the photoisomerization of these active units could directly trigger variation of the macrocycle cavity size, shape and so on, enabling efficient photocontrolled manipulation of their guest binding ability. However, as photoresponsive units are embedded into the scaffolds of well-established macrocycles such as crown ethers,^1,3^ cyclodextrins,^4,5^ calixarenes,^6,7^ cucurbituril,^8–10^ pillararenes,^11,12^ and “blue box” (CBPQT^4+^),^13^ perturbation of the pristine skeleton structure is inevitable, resulting in the degradation of their original host–guest properties. Furthermore, the isomerization of the photoactive units is often hindered by ring strain or molecular crowding.^63^ Thus, seamlessly incorporating robust phototunable functionality into well-established macrocycles while preserving their original host–guest properties presents a formidable challenge. In light of the prominent and abundant host–guest properties of well-established macrocycles,^1–13^ breaking this limitation could unlock promising new avenues for exploiting photocontrollable supramolecular self-assemblies and materials.

**Fig. 1:**
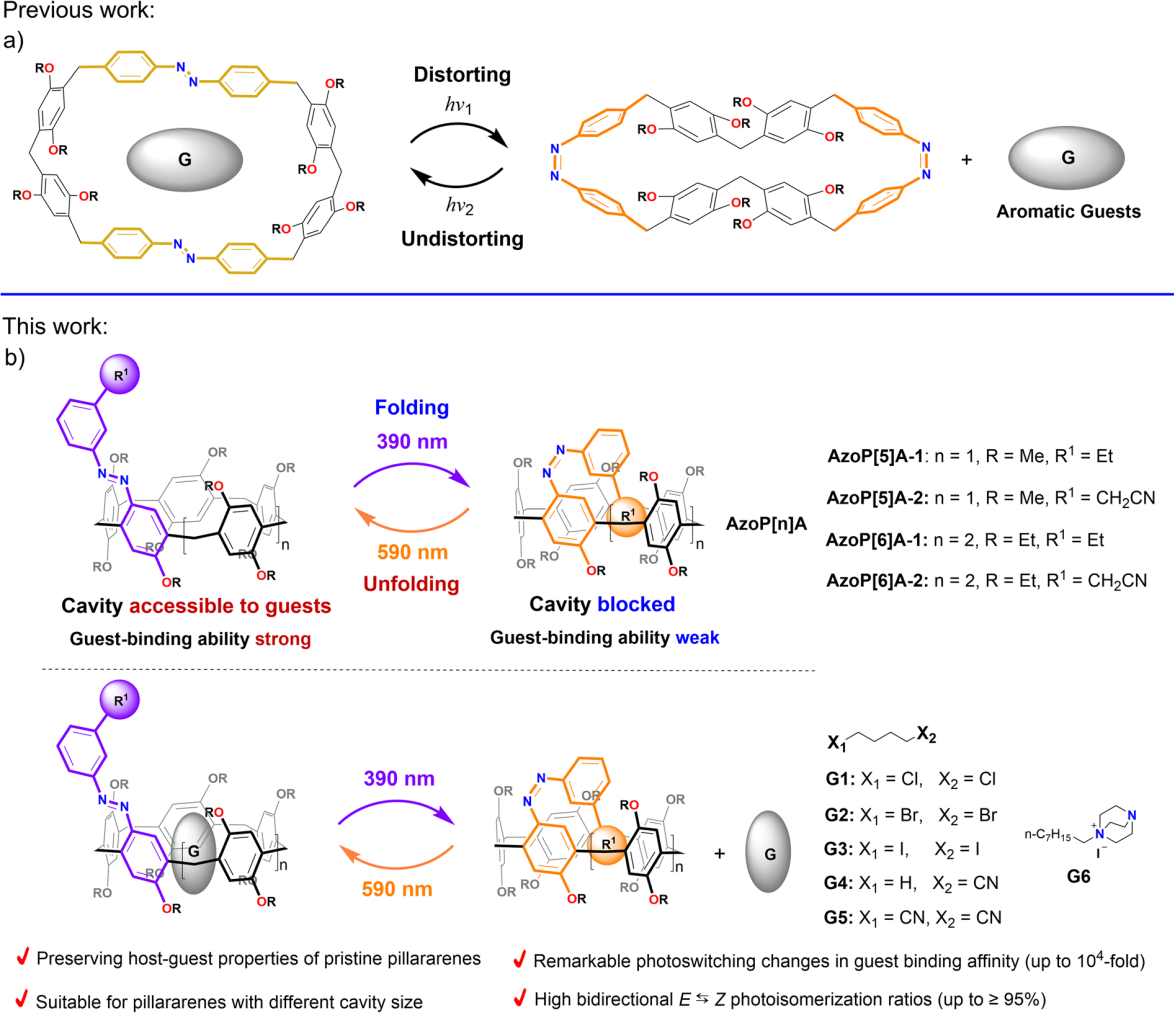
Schematic representation for (a) previously reported photoswitchable pillararene-inspired macrocycles based on an azo-embedded macrocyclic scaffold, and (b) this work: reversible photocontrolled self-folding pillararene macrocycles based on an azo-incorporated wall, enabling photoswitchable control over guest binding/releasing.

In less than two decades, pillararenes have emerged as a key class of macrocyclic host molecules with broad applicability across supramolecular chemistry and functional materials,^64–71^ because of their unique pillar shape, facile functionalization, and interesting and versatile host–guest properties.^11,12,64^ Nevertheless, robust photoresponsive pillararene macrocycles remain largely underexplored on account of limited strategies. Recent efforts confirmed that the incorporation of azobenzene units into the macrocycle scaffold of pillararenes could enable the creation of brand-new macrocycles featuring photocontrollable aromatic guest encapsulation and release (Fig. 1a),^41,56^ but this approach inevitably compromised the intrinsic host–guest properties of the pristine pillararenes, such as their good binding ability with alkane derivatives. In addition, although photoresponsive pillararene-based host systems can be obtained by covalently attaching photochromic units (azobenzenes, or stiff stilbenes) through flexible spacers at the portal sides of the pillararene macrocycles. However, achieving significant photocontrollable guest binding ability was difficult due to the lack of a high degree of preorganization and effective noncovalent interactions necessary to drive the formation of solid intramolecular self-inclusion complexes between pillararenes and photoresponsive units.^71–75^ Therefore, despite these achievements, it is highly desirable to develop an ingenious strategy for the construction of photoresponsive pillararene macrocycles featuring: (1) significant photocontrollable guest binding affinity, (2) high photoconversion yields, (3) well-retained original host–guest properties, and (4) adaptability across varied pillararenes.

To this end, as inspired by Rebek's pioneering works on the elegant design of photocontrolled self-filling cavitands,^40,52^ herein, we report the design and construction of a series of photoresponsive pillar[*n*]arenes (*n* = 5 or 6) with distinctive reversible photocontrolled self-folding behavior through directly introducing a photochromic azo unit onto the pillar[5/6]arenes' macrocyclic scaffold (AzoP[5]A-1,2 and AzoP[6]A-1,2 in Fig. 1b). When the azo unit is found in the *E*-configuration, the azo module could not be effectively self-included into the cavity of the *E*-isomeric AzoP[*n*]A, making these hosts possess a guest-accessible cavity. Moreover, the *E*-isomeric AzoP[*n*]A can exhibit guest binding ability comparable to the pristine alkylated pillararenes, as they have nearly identical cavity structures, suggesting that the host–guest properties of the pristine pillararenes are well preserved in these azo hosts. Upon 390 nm UV light irradiation, the azo unit undergoes highly efficient *E*-to-*Z* photoisomerization. The curved rigid configuration of the *Z*-azo unit enables the meta-substituted *R*^1^ group on the azo module to be located right inside the cavity (Fig. 1b), thereby allowing the *Z*-isomeric AzoP[*n*]A to adopt a stable self-folded conformation by forming intramolecular interactions between the *R*^1^ group and the inner cavity. This makes the *Z*-isomeric AzoP[*n*]A show a blocked cavity, resulting in a dramatic reduction in their binding affinities toward guest molecules. Notably, the difference in guest binding affinity between *E*- and *Z*-isomeric AzoP[*n*]A can be delicately tuned by varying the structure of the *R*^1^ group on the azo unit.

Furthermore, exposure to 590 nm yellow light irradiation could induce efficient reverse *Z*-to-*E* photoisomerization, enabling the AzoP[*n*]A to restore the guest-accessible cavity as well as the high guest binding ability. Hence, the host–guest complexation between the AzoP[*n*]A and guest molecules can be reversibly switched ON/OFF upon alternating UV and visible light irradiation. Additionally, to further test the potential of such AzoP[*n*]A in the construction of photocontrollable self-assemblies, the host–guest binding between AzoP[5]A-1 and nitrile guests was employed as an example of photoswitchable noncovalent crosslinking to achieve a reversible photoregulated supramolecular polymeric network.

## Results and discussion

### Reversible photocontrolled self-folding behaviours of the hosts AzoP[5]A-1,2

The synthesis and full structural assignments of the target photoresponsive pillar[5]arene (AzoP[5]A-1,2) and pillar[6]arene AzoP[6]A-1,2) macrocycles in Fig. 1b are presented in detail in the (SI). At the beginning of the research, the basic photoswitching properties of these azopillararenes were carefully evaluated by carrying out a series of UV-vis and ^1^H NMR spectroscopic experiments.

**Fig. 2:**
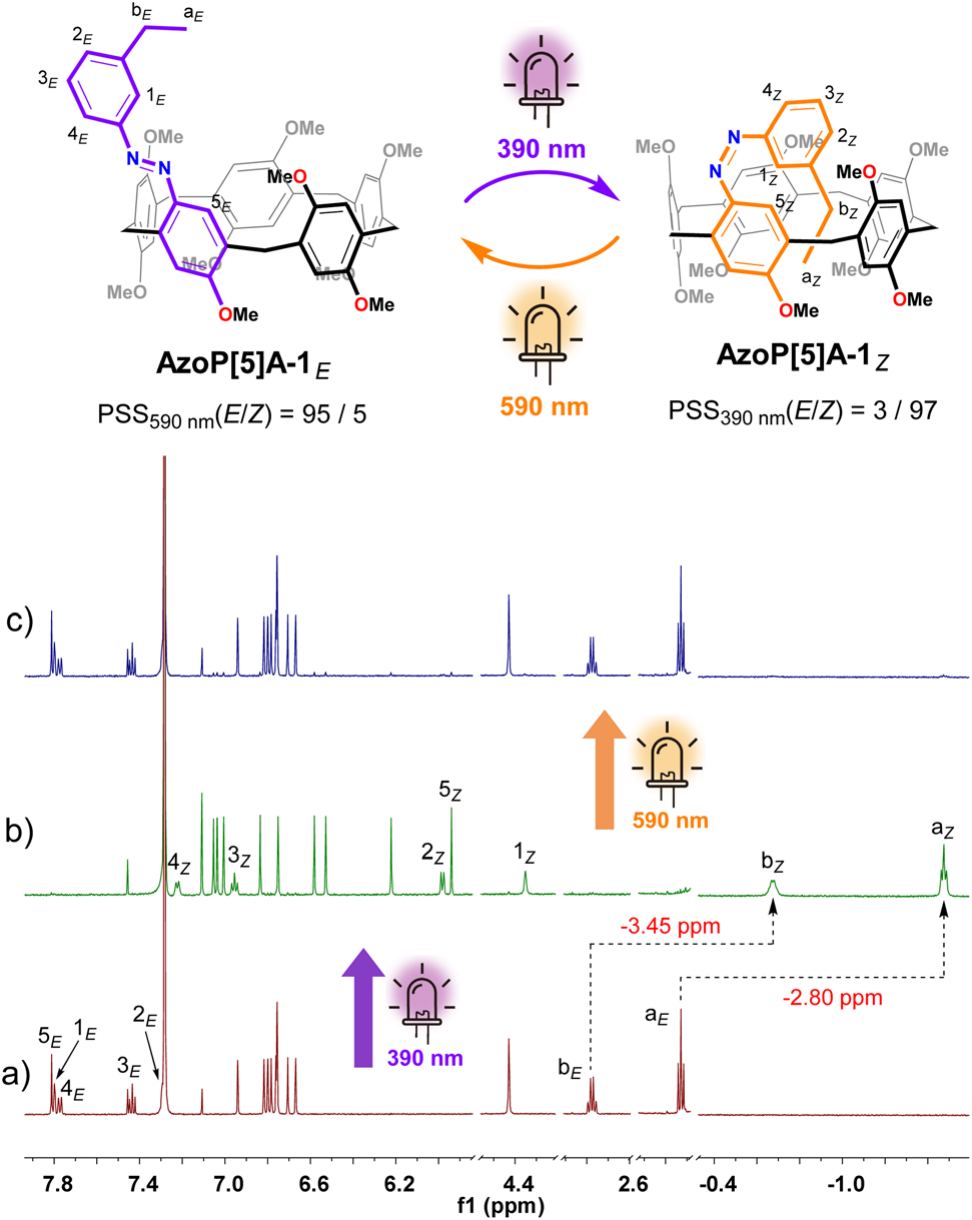
Schematic representation for the reversible *E*/*Z* photoisomerization of AzoP[5]A-1 and partial ^1^H NMR (600 MHz, CDCl_3_, 298 K) spectra for (a) the solution of AzoP[5]A-1*_E_* (2.0 mM), (b) the PSS_*Z*_ (390 nm) mixtures of AzoP[5]A-1 (2.0 mM), and (c) the PSS_*E*_ (590 nm) mixtures of AzoP[5]A-1 (2.0 mM).

For the pillar[5]arenes AzoP[5]A-1 and AzoP[5]A-2, UV-vis spectroscopic examinations were performed to determine the optimal light sources for triggering the *E* ⇆ *Z* photoisomerization of the azo units (390 nm UV light for the *E*-to-*Z* and 590 nm yellow light for the *Z*-to-*E* process) (Fig. S1). As shown in the recorded ^1^H NMR spectra (Fig. 2b and S8b), upon 390 nm UV light irradiation, AzoP5[A]-1 could undergo a nearly complete *E*-to-*Z* isomerization to reach a photostationary state (PSS) with a high content of *Z*-isomer AzoP5[A]-1*_Z_* (*E*/*Z* = 3/97). Notably, the *meta*-positioned ethyl group of the azo unit in AzoP[5]A-1*_Z_* exhibited upfield chemical shifts at −1.48 ppm (H-a_*Z*_) and −0.68 ppm (H-b_*Z*_) (Fig. 2b), which were significantly upfield shifted by −2.80 ppm and −3.45 ppm, respectively, as compared to the corresponding signals (H-a_*E*_ and H-b_*E*_) of the *E*-isomer AzoP[5]A-1*_E_* (Fig. 2a). This indicates that *Z*-isomer AzoP[5]A-1*_Z_* adopts a self-folded conformation with the ethyl group filling the cavity, stabilized by the formation of intramolecular C–H⋯π interactions between the ethyl group and the inner wall of the cavity. Upon subsequent 590 nm yellow light irradiation, the *Z*-to-*E* photoisomerization of AzoP[5]A-1 could be triggered to give a PSS mixture enriched with *E*-isomer (*E*/*Z* = 95/5), indicating the highly efficient reversible photoisomerization of AzoP[5]A-1 (Fig. 2c and S8c).

Such nearly quantitative photoinduced *E* ⇆ *Z* isomerization was also observed in the analogous AzoP[5]A-2 with a *meta*-positioned cyanoethyl group on the azo unit, which was introduced to enhance the intramolecular C–H⋯π interactions^76^ in the self-folded state to increase the difference in guest binding ability between the *E*- and *Z*-isomeric hosts. The ^1^H NMR experiments showed that the azo unit of AzoP[5]A-2 could undergo efficient *E* ⇆ *Z* photoisomerization upon irradiation with 390 nm and 590 nm light, affording high contents of *Z*-isomer AzoP[5]A-2*_Z_* (*E*/*Z* = 3/97) at PSS_390nm_ and *E*-isomer AzoP[5]A-2*_E_* (*E*/*Z* = 96/4) at PSS_590nm_ (Fig. S9), respectively. Similarly, the *Z*-isomer AzoP[5]A-2*_Z_* was found to adopt a folded conformation with the *meta* cyanoethyl group being self-included in the cavity, as indicated by the observed negative chemical shifts for the methylene proton (H-a_*Z*_) of the cyanoethyl group (Fig. S9b). Moreover, the time-dependent ^1^H NMR spectroscopic analysis revealed that both *Z*-isomers AzoP[5]A-1*_Z_* (*t*_1/2_ = 192.5 h) and AzoP[5]A-2*_Z_* (*t*_1/2_ = 2567 h) displayed high thermal stability (Fig. S10, S11, S14 and S15). The unusual stability of the *Z*-isomers was probably ascribed to the *Z*-isomers AzoP[5]A-1*_Z_* and AzoP[5]A-2*_Z_* presenting a solid self-folding conformation through the efficient formation of intramolecular C–H⋯π interactions between the ethyl or cyanoethyl group on the *Z*-azo unit and the cavity. This hypothesis has been further confirmed by the observation of significantly accelerated thermal Z → E isomerization rates of *Z*-isomeric AzoP[5]A-1*_Z_*/2*_Z_* hosts in the presence of excess complementary guests (Fig. S12, S13, S16 and S17). In addition, AzoP[5]A-1 and AzoP[5]A-2 displayed high light fatigue resistance, as indicated by the repetitive UV-vis absorption spectra showing almost no signs of degradation after ten photoswitching cycles (Fig. S4 and S5).

**Fig. 3:**
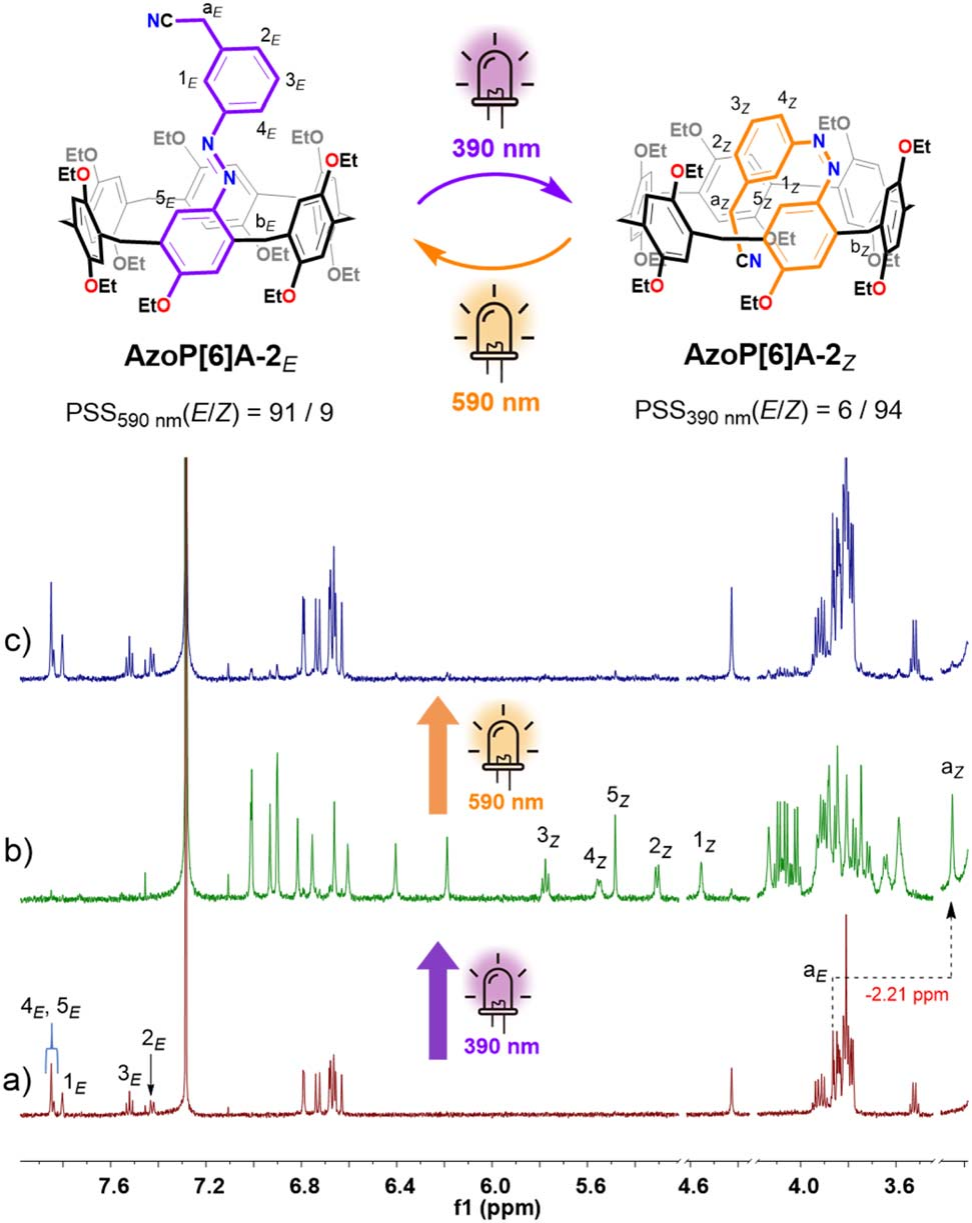
Schematic representation of the reversible *E*/*Z* photoisomerization of AzoP[6]A-2 and partial ^1^H NMR (600 MHz, CDCl_3_, 298 K) spectra for (a) the solution of AzoP[6]A-2*_E_* (2.0 mM), (b) the PSS_*Z*_ (390 nm) mixtures of AzoP[6]A-2 (2.0 mM), and (c) the PSS_*E*_ (590 nm) mixtures of AzoP[6]A-2 (2.0 mM).

In the cases of azopillar[6]arene analogs AzoP[6]A-1 and AzoP[6]A-2 (Fig. 1b), UV-vis spectroscopic examination suggested that 390 nm UV light and 590 nm yellow light were also optimal for triggering the *E* ⇆ *Z* photoisomerization processes (Fig. S2). Based on the ^1^H NMR measurements (Fig. 3, S18 and S19), the *Z*-isomer compositions of these pillar[6]arenes at PSS_390nm_ could be quantified as 95% for AzoP[6]A-1*_Z_* and 94% for AzoP[6]A-2*_Z_*, and their *E*-isomer proportions at PSS_590nm_ were determined as 97% for AzoP[6]A-1*_E_* and 91% for AzoP[6]A-2*_E_*, respectively. Meanwhile, both AzoP[6]A-1 and AzoP[6]A-2 exhibited good light fatigue resistance as well, indicated by the repetitive irradiation experiments (Fig. S6 and S7). More importantly, the unique ability of photocontrolled self-folding was also demonstrated in AzoP[6]A-1 and AzoP[6]A-2, as revealed by the observation of significantly up-shifted chemical shifts for the *meta* ethyl (H-a_*Z*_ and H-b_*Z*_ in Fig. S18b) or cyanoethyl group (H-a_*Z*_ in Fig. 3b and S19b) on the azo units, compared to the corresponding signals for the *E*-isomers (Fig. 3a and S18a). However, as depicted in Fig. S20–S23, relatively shorter half-lives for the *Z*-isomers AzoP[6]A-1*_Z_* (*t*_1/2_ = 20.6 h) and AzoP[6]A-2*_Z_* (*t*_1/2_ = 52.5 h) were observed, indicating the reduced thermal stability of the *Z*-isomeric AzoP[6]A as compared to AzoP[5]A*_Z_*. These results suggest that the self-folded conformation of AzoP[6]A*_Z_* is not as tight as that of AzoP[5]A*_Z_*, arising from the AzoP[6]A hosts having larger and more flexible cavities. These findings collectively substantiate the efficacy of our molecular design strategy in the rational construction of photoresponsive pillararenes with different cavity sizes.

### Photoswitchable guest binding properties of the hosts AzoP[5/6]A-1,2

After elucidating the reversible photocontrolled self-folding behavior of these azopillararenes, their photoregulated guest binding abilities were investigated. A series of *n*-alkanenitriles or *n*-halogenoalkanes derivatives (G1–G5) as well-known guests for pillar[5]arenes,^12^ and a cationic diazabicyclo[2.2.2]octane (DABCO) derivative G6 as a well-known guest for pillar[6]arenes,^12^ were chosen as the complementary guest molecules to these azopillar[5/6]arenes (Fig. 1b). The host–guest complexation behavior for both the *E*- and *Z*-isomers of AzoP[5]A-1,2 and AzoP[6]A-1,2 toward the selected guests G1–G6, respectively, were investigated by carrying out ^1^H NMR and UV-vis experiments (see Sections. 3–5 in the SI for detailed spectral data), which afforded the corresponding association constants as summarized in Table 1.

**Table 1:** Association constants for the 1 : 1 stoichiometric host–guest complexation of guests G1–G6 with AzoP[5]A or AzoP[6]A in CDCl_3_/CHCl_3_ at 298 K

Guests	Hosts	*K*_a_ M^−1^	*K*_a_^ON^ (E)/*K*_a_^oFF^ (Z)
G1	AzoP[5]A-1*_E_*	2252 ± 210^a^	777
	AzoP[5]A-1*_Z_*	2.9 ± 0.9^a^	
G2	AzoP[5]A-1*_E_*	1975 ± 128^a^	760
	AzoP[5]A-1*_Z_*	2.6 ± 0.8^a^	
G3	AzoP[5]A-1*_E_*	2311 ± 179^a^	745
	AzoP[5]A-1*_Z_*	3.1 ± 1.0^a^	
G4	AzoP[5]A-1*_E_*	507 ± 10^a^	> 507
	AzoP[5]A-1*_Z_*	<1^a^	
G5	AzoP[5]A-1*_E_*	2.7 × 10^4^^b^	722
	AzoP[5]A-1*_Z_*	37.4 ± 3.6^c^	
G5	AzoP[5]A-2*_E_*	2.5 × 10^4^^a^	10 870
	AzoP[5]A-2*_Z_*	2.3 ± 0.3^c^	
G6	AzoP[6]A-1*_E_*	491 ± 25^a^	7.4
	AzoP[6]A-1*_Z_*	66.7 ± 2.2^a^	
G6	AzoP[6]A-2*_E_*	521 ± 18^a^	121
	AzoP[6]A-2*_Z_*	4.8 ± 1.1^a^	

a The *K*_a_ values were determined by the ^1^H NMR titration method.

b The *K*_a_ values were determined by the ^1^H NMR single-point method.

c The *K*_a_ value was determined by the UV-vis absorption spectrophotometric titration method.

**Fig. 4:**
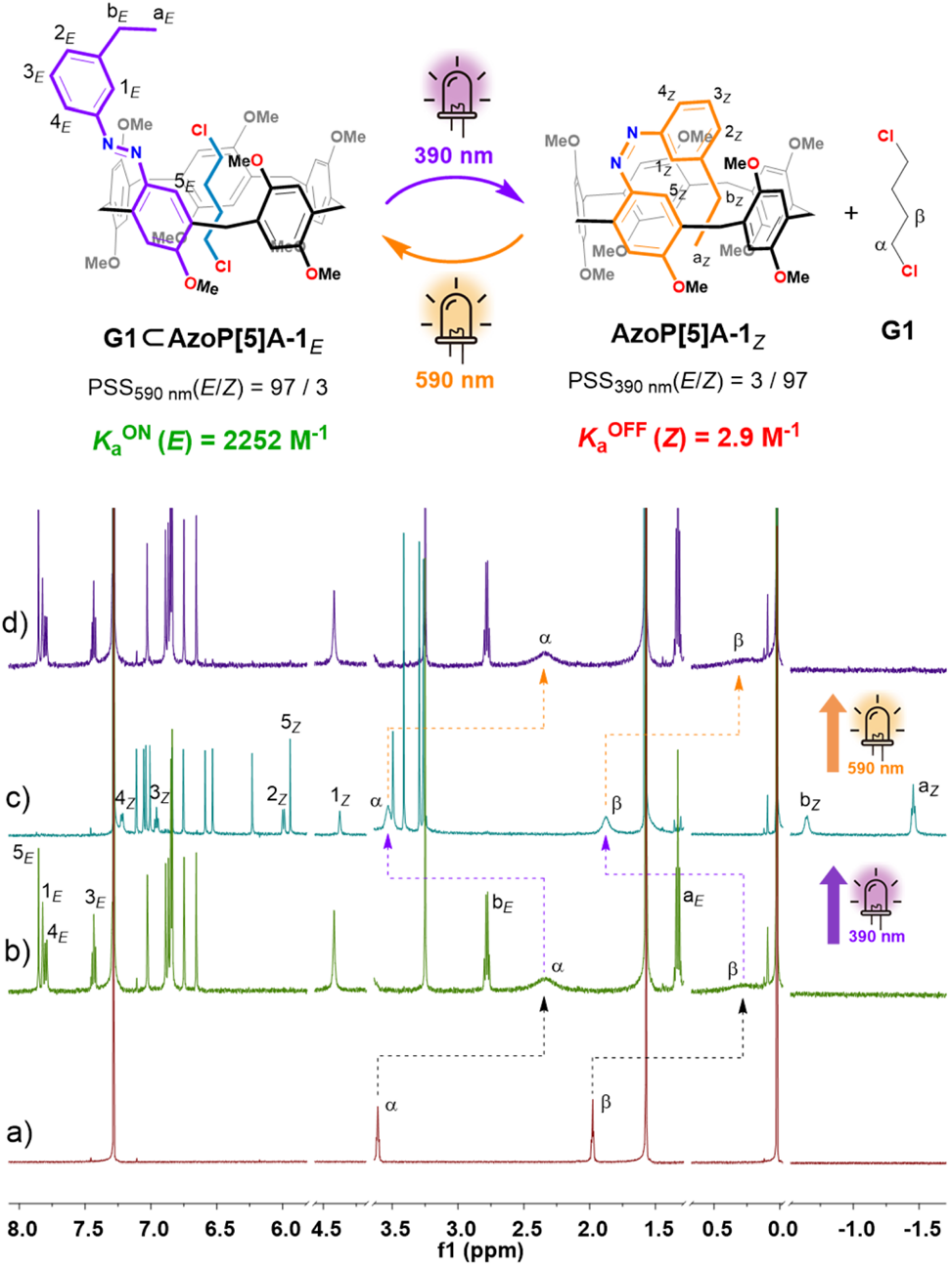
Schematic representation of the photoswitchable host–guest complexation of AzoP[5]A-1 and G1, and partial ^1^H NMR (600 MHz, CDCl_3_, 298 K) spectra for the solution of (a) G1 (2.0 mM) and (b) AzoP[5]A-1*_E_* (2.0 mM) and G1 (2.0 mM), (c) the PSS_*Z*_ (390 nm) mixtures of AzoP[5]A-1 (2.0 mM) and G1 (2.0 mM), and (d) the PSS_*E*_ (590 nm) mixtures of AzoP[5]A-1 (2.0 mM) and G1 (2.0 mM).

**Fig. 5:**
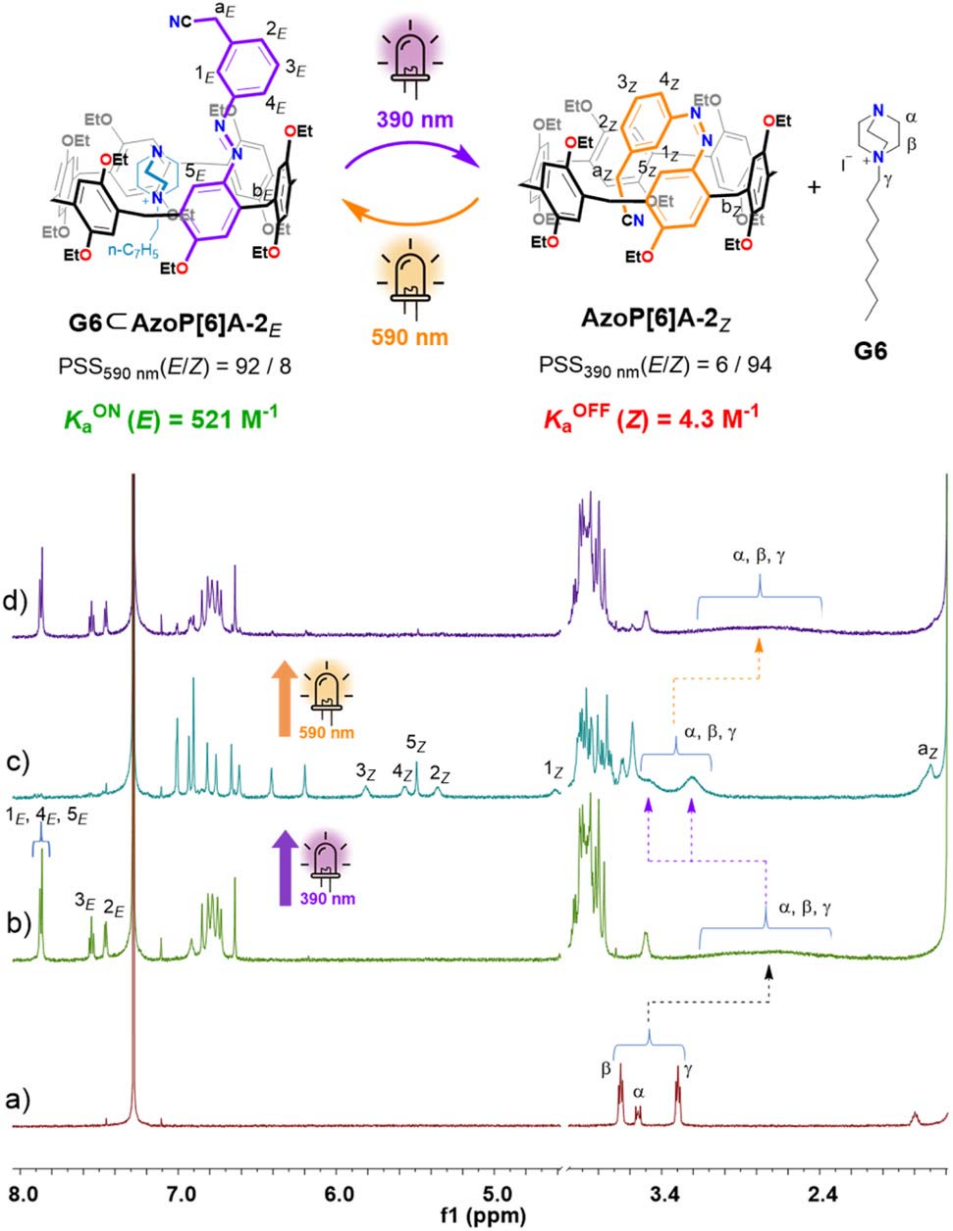
Schematic representation of the photoswitchable host–guest complexation of AzoP[6]A-2 and G6, and partial ^1^H NMR (600 MHz, CDCl_3_, 298 K) spectra for the solution of (a) G6 (2.0 mM) and (b) AzoP[6]A-2*_E_* (2.0 mM) and G6 (2.0 mM), (c) the PSS_*Z*_ (390 nm) mixtures of AzoP[6]A-2 (2.0 mM) and G6 (2.0 mM), and (d) the PSS_*E*_ (590 nm) mixtures of AzoP[6]A-2 (2.0 mM) and G6 (2.0 mM).

For the *E*-isomeric AzoP[5/6]A, it was found that the ^1^H NMR spectrum of an equimolar mixture of AzoP[5]A-1*_E_* and G1–G4 (Fig. 4b and S48 b–51b), as well as AzoP[6]A-1*_E_*/2*_E_* and G6 (Fig. 5b, S54b and S55b), exhibited significantly upfield shifted and broadened proton (H-α and H-β) signals of the guests G1–G4 and G6 upon binding to the hosts. These host–guest complexes were formed by fast exchange on the NMR timescale as reflected by the gradually shifted host and guest signals in the ^1^H NMR titration spectra (Fig. S24, S26, S28 and S30). Accordingly, the corresponding association constants for the complexes (G1–G4)⊂AzoP[5]A-1*_E_* could be determined as *K*_a_ = 2252, 1975, 2311 and 507 M^−1^ (Table 1), respectively. Regarding the complexes G6⊂AzoP[6]A-1*_E_*/2*_E_*, the ^1^H NMR titrations also afforded their association constants as *K*_a_ = 491 and 521 M^−1^ (Table 1, Fig. S38–S41), respectively. However, in the cases of G5 complexing with AzoP[5]A-1*_E_*,2*_E_*, slow exchange on the NMR timescale was observed, as supported by the observation of a new set of steady guest signals with negative chemical shifts, rather than gradual shifting upon titration of G5 (Fig. S32 and S36). Thus, ^1^H NMR single-point methods were used to measure the binding strength of complexes G5⊂AzoP[5]A-1*_E_*/2*_E_*, and the *K*_a_ values were determined to be 2.7 × 10^4^ and 2.5 × 10^4^ M^−1^ (Table 1, Fig. S35 and S37), respectively. These results emphasize that the chosen guests suitable for conventional pillararenes could also be effectively taken up by this new family of azo-modified pillararenes. More importantly, the obtained guest binding constants of these *E*-isomeric AzoP[5/6]A hosts were very close to those of their alkylated pillararene counterparts (MeP[5]A and *_E_*tP[6]A) (Table S2 and Fig. S42–S47). These findings indicate that the AzoP[5/6]A-1*_E_*,2*_E_* hosts well preserve the original guest binding capabilities of the pristine pillar[5/6]arenes.

Furthermore, such host–guest binding behavior between the AzoP[5/6]A and complementary guests was found to be reversibly photoregulable. After the prepared *E*-isomeric host–guest mixtures were irradiated by 390 nm UV light, the guests G1–G4 and G6 with moderate binding ability displayed downfield shifted chemical shifts almost close to those of the corresponding “free” guests (Fig. 4c, 5c, S48c–S51c, S54c and S55c). Even the G5⊂AzoP[5]A-1*_E_*/2*_E_* complexes with high binding constants were dissociated after irradiation with 390 nm UV light, as reflected by the significantly increased signals of the “free” guest G5 and almost disappeared signals of the encapsulated guest G5 in the ^1^H NMR spectra (Fig. S52c and S53c). These observations confirm the highly efficient photo-triggered release of the encapsulated guests from these pillararene hosts. Subsequently, for all the 390 nm UV light-irradiated mixtures, exposure to 590 nm yellow light irradiation could regenerate the host–guest complexes, as indicated by the observation of restored upfield shifted guest signals (Fig. 4d, 5d, and S48d–S55d). Notably, the ^1^H NMR experiments under alternate 390 nm/590 nm light irradiation conditions also revealed that the AzoP[5/6]A were capable of undergoing efficient *E* ⇆ *Z* photoisomerization in the presence of complementary guests, with the proportions of their *Z*-isomers at PSS_390nm_ or *E*-isomers at PSS_590nm_ being determined as both up to 97% (Fig. 4 and 5 and S48–S55).

Meanwhile, the binding constants between the *Z*-isomeric AzoP[5/6]A and chosen guests were measured by carrying out ^1^H NMR or UV-vis titration experiments (Section 5 in the ESI). The collective observations revealed that, upon addition of the guests, the recorded ^1^H NMR titration spectra displayed only minimal or even negligible chemical shift changes of the *Z*-isomeric hosts, with no sign of newly appearing signals (Fig. S56, S58, S60, S62, S64, S66, S68 and S70). Accordingly, extremely small binding constants (*K*_a_ < 5 M^−1^) were acquired for the complexes (G1–G4)⊂AzoP[5]A-1*_Z_* (Fig. S57, S59, S61 and S63), G5⊂AzoP[5]A-2*_Z_* (Fig. S67) and G6⊂AzoP[6]A-2*_Z_* (Fig. S71), whereas the complexes G5⊂AzoP[5]A-1*_Z_* and G6⊂AzoP[6]A-1*_Z_* showed moderate binding constants valued at *K*_a_ = 37.4 and 66.7 M^−1^ (Fig. S65 and S69), respectively. These results clearly demonstrate that the *Z*-isomeric AzoP[5/6]A hosts exhibit steeply declined guest binding affinities as compared to their *E*-isomeric counterparts, due to the host cavity being self-filled with *meta* cyanoethyl or ethyl groups of the *Z*-azo unit driven by intramolecular C–H⋯π interactions.

To provide more intuitive evaluation on the guest binding performance of such isomer-governed host–guest systems, the ON/OFF binding constant photoswitching ratios *K*_a_^ON^ (*E*)/*K*_a_^OFF^ (*Z*) were further quantified. As shown in Table 1, upon complexation with G1–G5, the *E*/*Z*-isomers of hosts AzoP[5]A-1/2 demonstrate remarkable photoswitchable guest binding affinity with high *K*_a_^ON^ (*E*)/*K*_a_^OFF^ (*Z*) values of > 500. Among them, the complexation between AzoP[5]A-2 and G5 displayed the most pronounced isomer-specific binding affinity difference, by showing a *K*_a_^ON^ (*E*)/*K*_a_^OFF^ (*Z*) value of 10 870. This could be attributed to the proton acidity of the *meta* cyanoethyl group on the azo unit being stronger than that of the *meta* ethyl group, enabling the former to enhance C–H⋯π interactions with the host cavity, resulting in the *Z*-isomeric AzoP[5]A-2*_Z_* adopting a more solid self-folded conformation than *Z*-isomeric AzoP[5]A-1*_Z_*. The stronger interaction ability between the cyanoethyl group and host cavity was also confirmed by the ^1^H NMR dilution experiment of AzoP[5]A-2*_E_* (Fig. S74 and S75), affording an intermolecular dimerization binding constant of *K*_dim_ = 10 M^−1^, which was higher than the *K*_dim_ (<1 M^−1^) of AzoP[5]A-1*_E_* (Fig. S72 and S73). Furthermore, similar phenomena were observed for the larger-sized pillar[6]arenes AzoP[6]A. As shown in Table 1, upon complexation with G6, the *meta*-cyanoethyl-modified AzoP[6]A-2 revealed a higher *K*_a_^ON^ (*E*)/*K*_a_^OFF^ (*Z*) value (121) as compared to that (7.4) of the *meta*-ethyl-modified AzoP[6]A-1. Nevertheless, the photoswitchable guest binding ability of AzoP[6]A-1/2 is less pronounced than that of their AzoP[5]A counterparts, arising from the increased size and conformational flexibility of pillar[6]arene's cavity that reduces its interaction ability with the *meta* cyanoethyl and ethyl group^12^ on the azo unit, as indicated by the nearly undetectable intermolecular dimerization (*K*_dim_ < 1 M^−1^) of AzoP[6]A-2*_E_* with the acidic cyanoethyl group (Fig. S76 and S77). These findings imply that these photoresponsive azopillararenes are expected to facilitate the development of host–guest self-assembly with significant photoswitchability.

### Photocontrollable host–guest interaction-mediated supramolecular polymeric assembly

**Fig. 6:**
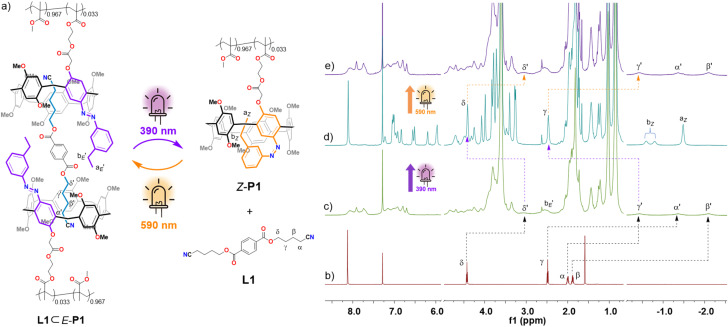
(a) Schematic representation of the photocontrolled supramolecular polymeric assembly of the ditopic guest L1 and polymer P1 with sidechain modified AzoP[5]A-1 units, and the partial ^1^H NMR spectra (600 MHz, CDCl_3_, 298 K) for the solution of (b) L1 (10 mM), (c) the mixture of *E*-P1 (78.2 mg/mL, 20 mM of AzoP[5]A-1 unit) and L1 (10 mM), (d) the PSS_*Z*_ (390 nm) mixtures of P1 (78.2 mg mL^−1^, 20 mM of AzoP[5]A-1 unit) and L1 (10 mM), and (e) the PSS_*E*_ (590 nm) mixtures of P1 (78.2 mg mL^−1^, 20 mM of AzoP[5]A-1 unit) and L1 (10 mM).

Given the broad utility of pillar[5]arene in supramolecular chemistry, together with the observation that AzoP[5]A-1 could reveal distinct photoswitchable guest binding affinity (Table 1) as well as almost negligible intermolecular interactions between the hosts themselves (Fig. S72 and S73), we further prepared a dinitrile guest L1 as a supramolecular crosslinker and a linear polymer P1 with its sidechains modifying the host AzoP[5]A-1 modules (Fig. 6a), to show the application of such azopillararene-based host–guest interactions for achieving photocontrolled supramolecular self-assemblies. Prior to this, the photoswitchable host–guest binding behavior of AzoP[5]A-1 with L1 was examined. The ^1^H NMR experiments revealed that the ditopic guest L1 was able to complex with two *E*-isomeric AzoP[5]A-1*_E_* to form a ternary complex L1⊂(AzoP[5]A-1*_E_*)_2_ (Fig. S78), which could undergo reversible photo-triggered dissociation/reformation behavior upon 390 nm and 590 nm light irradiation (Fig. S79), respectively. Moreover, the AzoP5A-1 unit on the sidechains of polymer P1 was able to undergo highly efficient *Z*/*E* photoisomerization upon irradiation by 390 nm and 590 nm light, resulting in PSS mixtures containing a high proportion of *Z*- or *E*-isomers (up to 97%) (Fig. S80). These results demonstrate the practicability of transforming the AzoP[5]A-1 based host–guest interactions into photoswitchable noncovalent crosslinking to control the polymeric self-assembly.

When the ditopic guest L1 was mixed with polymer *E*-P1 in chloroform solution, a host–guest interaction was formed between the L1 and sidechain AzoP[5]A-1*_E_* units of *E*-P1, as confirmed by the appearance of broadened and distinctly upfield shifted proton signals (H-α′, H-β′, H-γ′ and H-d′) of the encapsulated L1 (Fig. 6b, S81b and S82b). The formation of supramolecular polymeric assemblies was further confirmed by the 2D DOSY-NMR spectrometric investigation. The polymer *E*-P1 solution revealed a diffusion coefficient of *D*_1_ = 1.78 × 10^−11^ m^2^ s^−1^ (Fig. S83), whereas the mixture solution of *E*-P1 and L1 exhibited a reduced diffusion coefficient of *D*_2_ = 4.79 × 10^−12^ m^2^ s^−1^ (Fig. S84), suggesting the formation of polymeric self-assemblies with around 51-fold increased hydrodynamic volume as compared to the discrete *E*-P1 in the solution.

**Fig. 7:**
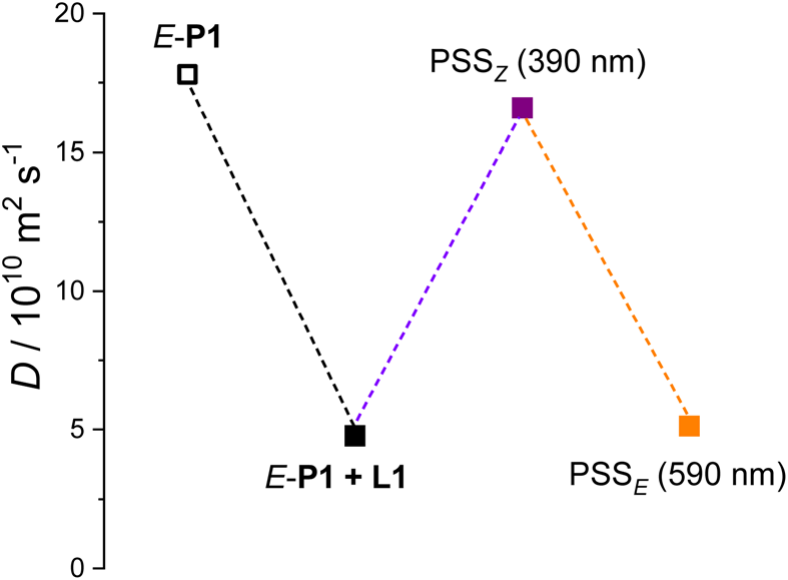
Recorded diffusion coefficient changes for the chloroform solution of *E*-P1 (78.2 mg mL^−1^), and the mixture of P1 (78.2 mg mL^−1^, with 20 mM AzoP[5]A-1 unit) and L1 (10 mM) in the pristine state, PSS_*Z*_ (390 nm) and PSS_*E*_ (590 nm), respectively.

Furthermore, after irradiation with 390 nm UV light, the polymeric self-assemblies were disassembled as indicated by the significant signals (H-β and H-γ) of the released “free” L1, as well as those of the *Z*-isomeric AzoP[5]A-1*_Z_* unit (H-a_*Z*_ and H-b_*Z*_) on the sidechain of P1 (Fig. 6c and S82c). Afterwards, the 390 nm UV light-irradiated mixture was exposed to 590 nm yellow light irradiation, leading to the reproduction of host–guest supramolecular polymeric assemblies, as evidenced by the restored signals of guest L1 captured by the sidechain AzoP[5]A-1*_E_* units (Fig. 6d and S82d). In parallel, such photoswitchable self-assembly processes were also validated by 2D DOSY-NMR analysis. As shown in Fig. 7, upon 390 nm UV light irradiation, the obtained PSS_*Z*_ mixture solution demonstrated a diffusion coefficient of *D*_3_ = 1.66 × 10^−11^ m^2^ s^−1^, close to the value of pure *E*-P1, indicating the disassembly of the polymeric self-assemblies (Fig. S85). After subsequent 590 nm yellow light irradiation, the mixture solution once again showed a decreased diffusion coefficient of *D*_4_ = 5.13 × 10^−12^ m^2^ s^−1^ comparable to that of the *E*-P1 and L1 mixture, suggesting the reformation of host–guest interaction-based polymeric self-assemblies (Fig. S86). These results demonstrate that the established photoresponsive pillararene-based host–guest interactions can be applied as a photoswitchable noncovalent driving force for the construction of photocontrollable supramolecular self-assembly systems. The unique spatiotemporal and noninvasive precision of structural regulation may benefit such photoresponsive supramolecular polymer networks for potential application in smart dynamic materials.

## Conclusions

In this study, we employed a reversible photocontrolled self-folding strategy for the first time to impart robust photoswitchability to pillararene macrocycles while well maintaining their original host–guest properties. Through rational introduction of a photochromic azo unit onto the pillararene scaffold, the resulting azopillar[5/6]arene macrocycles AzoP[5/6]A can undergo reversible self-folding/unfolding by the *Z*/*E* photoisomerization of the azo unit upon 390 nm/590 nm light irradiation. In the unfolded state, these hosts are found to bind guest molecules with affinities comparable to those of the pristine pillararenes, whereas in the folded state, the hosts' cavity turns out to be largely guest inaccessible as it is blocked by intramolecular C–H⋯π interactions with the *meta* ethyl or cyanoethyl group of the *Z*-azo unit. Such unique traits endow the macrocycles AzoP[5/6]A with the ability to function as smart hosts whose guest binding ability can be effectively switched ON/OFF by light stimuli. Benefiting from this, novel photocontrolled supramolecular polymeric assemblies can be readily constructed by integrating such azopillararenes into polymer chains to regulate host–guest interaction-directed supramolecular polymerization. Given the established significance and wide applicability of pillararenes, these azopillararenes are a promising family of photoresponsive hosts, enabling the construction of novel spatiotemporally programmable functional supramolecular systems and materials.

## Author contributions

K. -D. Z., and T. -G. Z. conceived and supervised the project. K. -D. Z. designed the experiments with Y. H. and L. -J. L. providing data analysis and discussions. A. -R. L. and W. -P. G. performed the synthesis, the characterizations of the compounds, and the NMR and UV-vis spectroscopic experiments, while Q. J. aided in the synthesis. T. -G. Z. and K. -D. Z. wrote and edited the manuscript. All the authors discussed the results and reviewed the manuscript.

## Conflicts of interest

There are no conflicts to declare.

## Supplementary Material

SC-OLF-D5SC09696K-s001

## Data Availability

The data supporting this article have been included as part of the supplementary information (SI). Supplementary information: experimental methods, and characterisation data. See DOI: https://doi.org/10.1039/d5sc09696k.
