# Supplementary material for: Upgrading pillar[n]arenes to reversible photocontrolled self-folding hosts for photoswitchable guest uptake/release and self-assembly
Source: Chem Sci. 2026 Jan 26;17(14):7047–55. doi: 10.1039/d5sc09696k (PMC12902930; doi:10.1039/d5sc09696k)
Supplement: SC-017-D5SC09696K-s001 [file SC-017-D5SC09696K-s001.pdf]

Supplementary Information (SI) for Chemical Science.
This journal is © The Royal Society of Chemistry 2026

Electronic Supplementary Information

Upgrading Pillar[n]arenes to Reversible Photocontrolled Self-Folding Hosts for Photoswitchable Guest Uptake/Release and Self-Assembly

Ao-Ran Liu,† Wen-Ping Gong,† Qi Jin, Tian-Guang Zhan,* Yu Hai,* Li-Juan Liu and Kang-Da Zhang*

Key Laboratory of the Ministry of Education for Advanced Catalysis Materials, College of Chemistry and Materials Science, Zhejiang Normal University, 688 Yingbin Road, Jinhua 321004, China.

E-mail: tgzhan@zjnu.cn; haiyuyu@zjnu.edu.cn; Kangda.Zhang@zjnu.cn

† These authors contributed equally to this work.

Table of Contents

Section 1: General Methods and Materials S2

Section 2: Synthesis Procedures and Characterization S3

Section 3: Photoswitching Properties of **AzoP[5]A-1,2** and **AzoP[6]A-1,2** S16

Section 4: Guest Binding Properties of the *E*-isomeric **AzoP[5/6]A** macrocycles S31

Section 5: Photoswitchable Host-Guest Complexation of **AzoP[5/6]A** and **G1 ~ G6** S46

Section 6: Guest Binding Properties of the *Z*-isomeric **AzoP[5/6]A** macrocycles S54

Section 7: Photocontrolled Host-Guest Interaction Mediated Macromolecular Self-Assembly S65

Section 8: The ^{1}H, ^{13}C NMR, 2D COSY, NOESY NMR Spectra for New Compounds S72

References S97

Section 1: Materials and General Methods

Materials: All reagents were used as received from the commercial suppliers without further purification; the solvents have been purified by standard procedures before use. The intermediate compounds **1**[S1], **5**[S2], and **8**[S3] were synthesized according to the reported procedures.

UV-Vis Spectroscopy: The UV-Vis absorption spectra were recorded on an Agilent Technologies Cary 60 UV-Vis spectrometer.

Nuclear Magnetic Resonance (NMR) Spectroscopy: The solution ^{1}H NMR and ^{13}C NMR spectra were recorded on a Bruker AVANCE 400 and 600 spectrometers, and the chemical shifts (δ in ppm) were determined by using a residual proton of the solvent as standard. The 2D COSY, NOESY and DOSY NMR spectra were collected on a Bruker AVANCE 600 spectrometer.

Light Source: The irradiation experiments were performed by using a 50 W LED lamp (390-395 nm) equipped with narrow band pass filter (390 nm) and a 100 W LED lamp (590-595 nm) equipped with narrow band pass filter (590 nm).

Section 2: Synthesis Procedures and Characterization

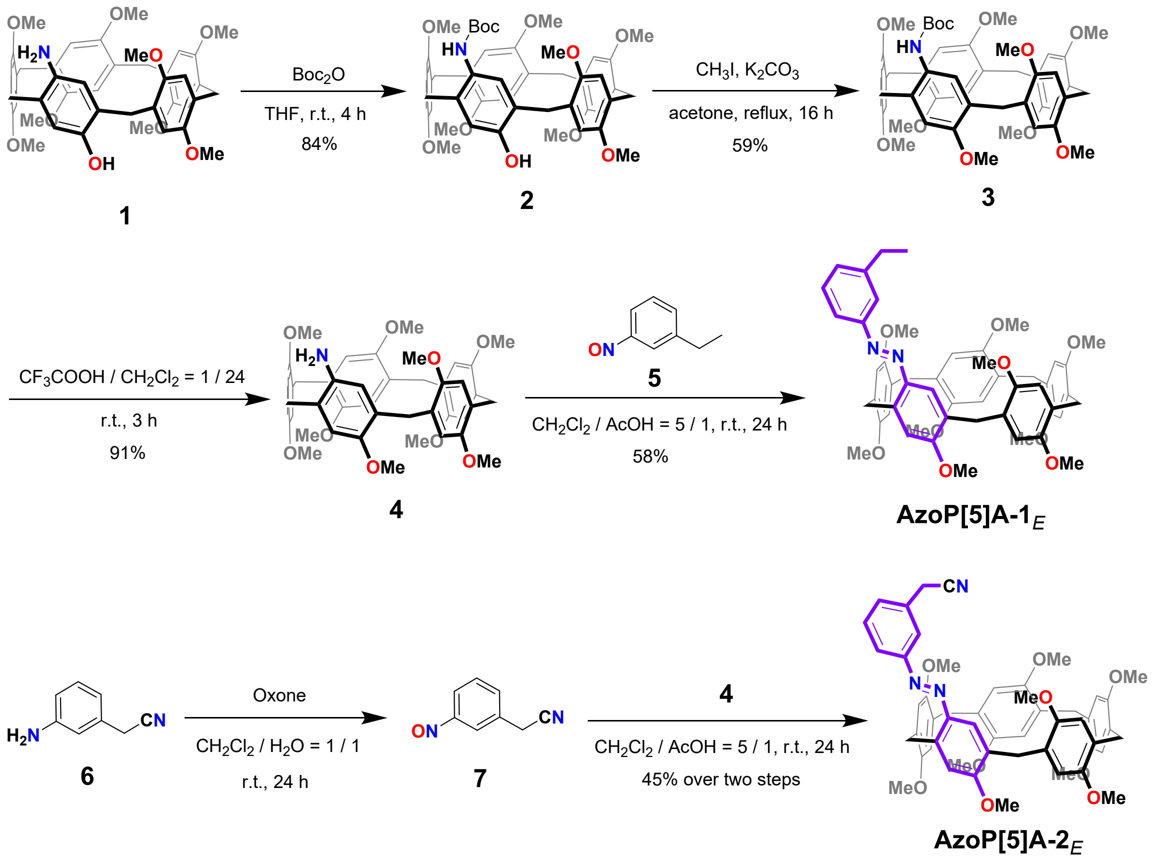

Scheme S1. Synthetic routs for the photoresponsive pillar[5]arenes **AzoP[5]A-1** and **AzoP[5]A-2**.

Compound 2: To a solution of compound **1**[S1] (340 mg, 0.471 mmol) in anhydrous THF (18 mL) was added Boc_2O (205 mg, 0.939 mmol), the resulting mixture was stirred at room temperature under nitrogen gas atmosphere for 4 hours. After the thin-layer chromatography (TLC) indicating the reaction was completed, the reaction mixture was concentrated to give light-yellow solid, which was then suspended in n-hexane (5 mL). After ultrasonication treatment for 5 minutes, the suspension was filtrated, and the collected solid was dried under vacuum to afford compound **2** as light-yellow solid (325 mg, 84%). ^1H NMR (600 MHz, DMSO-d_6, 298 K) δ (ppm): 9.05 (s, 1H), 8.19 (s, 1H), 6.90 (s, 1H), 6.86 (s, 1H), 6.82 (s, 1H), 6.80 (s, 1H), 6.78 (s, 2H), 6.76 (s, 1H), 6.75 (s, 1H), 6.72 (s, 1H), 6.71 (s, 1H), 3.77 (s, 3H), 3.71 (s, 6H), 3.70 (s, 3H), 3.68 (s, 2H), 3.66 (br, 5H), 3.64 (br, 7H), 3.58 (s, 3H), 3.49 (s, 2H), 1.44 (s, 9H). ^{13}C NMR (150 MHz, DMSO-d_6, 298 K) δ (ppm): 154.39, 152.42, 150.89, 150.59, 150.57, 150.46, 150.42, 150.40, 150.36, 136.29, 129.51, 129.06, 128.39, 128.37, 128.31, 128.14, 127.93, 127.87, 127.84, 127.11, 126.57, 118.04, 114.59, 114.44, 114.24, 114.15, 114.14, 130.90, 113.68, 113.49, 78.35, 56.47, 56.45, 56.15, 56.13, 56.05, 56.02, 55.96, 55.84, 31.92, 30.01, 29.28, 28.94, 28.72, 28.52. HRMS (ESI) Calcd. for $C_{48}H_{55}NNaO_{11}$ $[M+Na]^+$: 844.3371, Found: 844.3660.

Compound 3: To the solution of compound **2** (100 mg, 0.122 mmol) and K_2CO_3 (87.0 mg, 0.630 mmol) in acetone (2 mL) was added CH_3I (10 mL, 0.164 mmol), and the resulting mixture was heated up to

reflux under nitrogen gas atmosphere for 16 hours. The reaction mixture was allowed to cool naturally to room temperature, then the mixture was filtered and the filtrate was collected. The solvent was removed from the filtrate under reduced pressure to give a white solid, which was dissolved in dichloromethane (50 mL), and washed sequentially with water (3 × 50 mL) and saturated brine (3 × 50 mL). The collected organic phase was dried over anhydrous sodium sulfate (Na_2SO_4). After removing the desiccant by filtration, the filtrate was collected and concentrated by evaporation under reduced pressure. The obtained crude product was further purified by flash chromatography using an eluent mixture of EtOAc/PE = 1/8 (v/v), affording a white solid which was suspended in n-hexane (2 mL) and ultrasonicated for 5 minutes. The solid was collected by filtration and dried under vacuum to yield compound **3** as a white solid (60.0 mg, 59%). ^{1}H NMR (400 MHz, DMSO-d_6, 298 K) δ (ppm): 8.25 (s, 1H), 6.93 (s, 1H), 6.90 (s, 1H), 6.88 (s, 1H), 6.84 (s, 1H), 6.82 (s, 1H), 6.76 (s, 1H), 6.74 (s, 3H), 6.70 (s, 1H), 3.80 (s, 3H), 3.77 (s, 3H), 3.69-3.68 (m, 13H), 3.66 (br, 5H), 3.64 (s, 2H), 3.62 (s, 6H), 3.60 (s, 2H), 3.59 (s, 3H). ^{13}C NMR (100 MHz, DMSO-d_6, 298 K) δ (ppm): 154.32, 154.17, 150.84, 150.41, 150.39, 150.34, 150.33, 150.22, 150.18, 136.25, 129.12, 128.59, 128.32, 128.30, 128.25, 128.09, 127.89, 127.78, 127.65, 126.95,114.29, 114.27, 114.05, 113.90, 113.60, 113.45, 113.36, 113.30, 78.52, 56.18, 56.04, 56.01, 55.99, 55.88, 55.83, 55.71, 55.31, 32.67, 29.89, 29.39, 28.83, 28.66, 27.71. HRMS (ESI) Calcd. for $C_{49}H_{57}N_2NaO_{11}$ $[M+Na]^+$: 858.3829, Found: 858.3822.

Compound 4: Compound **3** (100 mg, 0.120 mmol) was dissolved in a mixture of dichloromethane (12 mL) and trifluoroacetic acid (TFA, 0.50 mL), and the reaction mixture was stirred at room temperature under a nitrogen atmosphere for 2 hours. The reaction progress was monitored by TLC, which indicated complete consumption of the starting material. The solvent was removed by evaporation under reduced pressure to give a white solid, which was dissolved in 20 mL dichloromethane and then sequentially washed with saturated sodium bicarbonate ($NaHCO_3$) aqueous solution (3 × 10 mL) and saturated brine (3 × 10 mL). The separated organic phase dried over anhydrous Na_2SO_4, and the desiccant was further removed by filtration. The collected filtrate was concentrated via evaporation under reduced pressure to yield compound **4** as a white solid (80.0 mg, 91%). ^{1}H NMR (400 MHz, DMSO-d_6, 298 K) δ (ppm): 6.88 (s, 1 H), 6.79-6.78 (m, 3 H), 6.76-6.75 (m, 3 H), 6.74 (s, 1 H), 6.69 (s, 1 H), 6.39 (s, 1 H), 4.33 (s, 2 H), 3.68-3.64 (m, 18 H), 3.64 (s, 3 H), 3.63 (s, 3 H), 3.62 (s, 3 H), 3.61 (s, 3 H), 3.58 (s, 3 H), 3.56 (br, 4 H). ^{13}C NMR (100 MHz, $CDCl_3$, 298 K) δ (ppm): 151.46, 151.03, 151.02, 150.98, 150.96, 150.93, 150.89, 150.33, 150.20, 137.66, 129.45, 129.12, 128.97, 128.45, 128.33, 127.86, 127.06, 125.27, 119.16, 114.79, 114.71, 114.39, 114.35, 114.31, 114.29, 114.10, 113.39, 56.51, 56.25, 56.13, 56.06, 56.02, 55.98, 55.93, 55.88, 55.86, 31.39, 30.59, 30.07, 29.67, 28.84. HRMS (ESI) Calcd. for $C_{44}H_{50}NO_9$ $[M+H]^+$: 736.3486, Found: 736.3472.

Compound AzoP[5]A-1$_E$: Compound **4** (100 mg, 0.136 mmol) and compound **5**[S2] (28.0 mg, 0.207 mmol) were dissolved in a mixture solution of glacial acetic acid (1 mL) and dichloromethane (5 mL). The reaction mixture was stirred at room temperature under a nitrogen atmosphere for 16 hours, with the reaction progress monitored by TLC. After the complete consumption of the starting material, the reaction was quenched by the addition of saturated $NaHCO_3$ aqueous solution, by which the pH value of the mixture was turned to 8.5. Then, the mixture was transferred to a separatory funnel to separate the organic phase, which was washed with saturated brine (3 × 20 mL). The combined organic phase was dried over anhydrous Na_2SO_4, and the desiccant was further removed by filtration. The filtrate was collected and concentrated via evaporation under reduced pressure to give crude product, which was purified by flash chromatography using a mixture of PE/EtOAc = 12/1 (v/v) as the eluent, affording a yellow viscous liquid.

The obtained viscous liquid was then suspended in n-hexane (2 mL) and the suspension was ultrasonicated for 5 minutes, after which the solid precipitate was collected by filtration. The collected solid was dried under vacuum to yield compound **AzoP[5]A-1$_E$** as an orange-yellow solid (67.0 mg, 58%). ^{1}H NMR (400 MHz, CD_3CN, 298 K) δ (ppm): 7.86 (s, 1H), 7.82 (d, J = 8.0 Hz, 1H), 7.77 (s, 1H), 7.50 (t, J = 8.0 Hz, 1H), 7.39 (d, J = 8.0 Hz, 1H), 7.17 (s, 1H), 6.96 (s, 2H), 6.94 (s, 1H), 6.93 (s, 1H), 6.92 (s, 1H), 6.88 (s, 1H), 6.79 (s, 1H), 6.68 (s, 1H), 4.38 (s, 2H), 3.93 (s, 3H), 3.82 (s, 3H), 3.80-3.79 (m, 5H), 3.78 (s, 3H), 3.76 (s, 3H), 3.75 (s, 3H), 3.73 (s, 5H), 3.70 (s, 2H), 3.68 (s, 2H), 3.64 (s, 3H), 3.25 (s, 3H), 2.80 (q, J = 7.6 Hz, 2H), 1.32 (t, J = 7.6 Hz, 3H). ^{13}C NMR (100 MHz, $CDCl_3$, 298 K) δ (ppm): 159.97, 153.51, 151.39, 151.05, 150.83, 150.81, 150.74, 150.65, 150.62, 145.23, 143.64, 142.37, 129.45, 128.87, 128.49, 128.33, 128.16, 128.13, 128.06, 128.00, 127.96, 127.66, 122.06, 119.99, 118.27, 114.44, 114.30, 114.25, 114.13, 114.07, 113.98, 113.80, 112.04, 55.96, 55.88, 55.87, 55.83, 55.75, 55.67, 55.21, 55.13, 33.00, 29.92, 29.97, 29.73, 29.32, 28.81, 15.55. HRMS (ESI) Calcd. for $C_{52}H_{57}N_2O_9$ $[M+H]^+$: 853.4064, Found: 853.4046.

Compound AzoP[5]A-2$_E$. To a solution of compound **6** (27.2 mg, 0.274 mmol) in dichloromethane (6 mL) was added an aqueous solution of potassium peroxymonosulfate (Oxone) (252 mg, 0.411 mmol, in 6 mL H_2O). The biphasic mixture was stirred at room temperature for 24 hours, until TLC monotoration indicated the complete consumption of starting material. The reaction mixture was transferred to a separatory funnel to separate the organic phase, which was washed sequentially with 6 mL hydrochloric acid aqueous solution (HCl_{aq}, 1.0 mol/L), saturated $NaHCO_3$ aqueous solution (10 mL) and saturated brine (10 mL), respectively. The combined organic phase was dried over anhydrous Na_2SO_4, which was further removed by filtration. The filtrate was collected and concentrated via evaporation under reduced pressure, affording compound **7** as a brown liquid, which was directly used in the next step without further purification.

To a solution of compound **4** (100 mg, 0.136 mmol) in a mixture of glacial acetic acid and dichloromethane (1/5, v/v, 6 mL total volume) was added the crude compound **7** from above step. The reaction mixture was stirred at room temperature under a nitrogen atmosphere for 24 hours, when TLC confirming the complete consumption of the starting material. The reaction was quenched by careful addition of saturated $NaHCO_3$ aqueous solution until pH = 8 was reached. The reaction mixture was transferred to a separatory funnel for phase separation, and the organic phase was washed with saturated brine (3 × 20 mL). After that, the combined organic phase was dried over anhydrous Na_2SO_4, which was further removed by filtration. The collected filtrate was concentrated via evaporation under reduced pressure to give crude product, which was purified by flash chromatography using a mixture eluent of PE/EtOAc = 25/1 (v/v), affording a yellow viscous liquid. The resulting viscous liquid was suspended in n-hexane (5 mL) and ultrasonicated for 5 minutes, then the precipitated solid was collected by filtration. The recieved solid was dried under vacuum to yield compound **AzoP[5]A-2**$_E$ as an orange-yellow solid (53.0 mg, 45%). ^{1}H NMR (400 MHz, CD_3CN, 298 K) δ (ppm): 8.00-7.97 (m, 2 H), 7.82 (s, 1 H), 7.62 (t, *J* = 7.6 Hz, 1 H), 7.51 (d, *J* = 7.6 Hz, 1 H), 7.18 (s, 1 H), 6.97 (s, 1 H), 6.96 (s, 1 H), 6.94 (s, 1 H), 6.93 (s, 1 H), 6.92 (s, 1 H), 6.88 (s, 1 H), 6.80 (s, 1 H), 6.67 (s, 1 H), 4.40 (s, 2 H), 3.99 (s, 2 H), 3.93 (s, 3 H), 3.83 (s, 3 H), 3.80 (br, 5 H), 3.78 (s, 3 H), 3.76 (d, 6 H), 3.73 (d, 5 H), 3.73 (s, 2 H), 3.68 (s, 2 H), 3.64 (s, 3 H), 3.27 (s, 3 H). ^{13}C NMR (100 MHz, $CDCl_3$, 298 K) δ (ppm): 160.54, 153.99, 150.94, 150.89, 150.86, 150.75, 150.69, 150.64, 143.79, 143.37, 133.17, 130.58, 130.34, 129.74, 129.32, 129.17, 129.10, 129.04, 128.84, 128.61, 128.37, 123.24, 122.14, 119.06, 117.71, 114.17, 113.82, 113.66, 113.60, 113.55, 112.29, 56.28, 55.90, 55.83, 55.77, 55.76, 55.73, 55.69, 55.34, 30.21, 29.44, 29.40, 29.37, 23.39. HRMS (ESI) Calcd. for $C_{52}H_{54}N_3O_9$ $[M+H]^+$: 864.3855, Found: 864.3853.

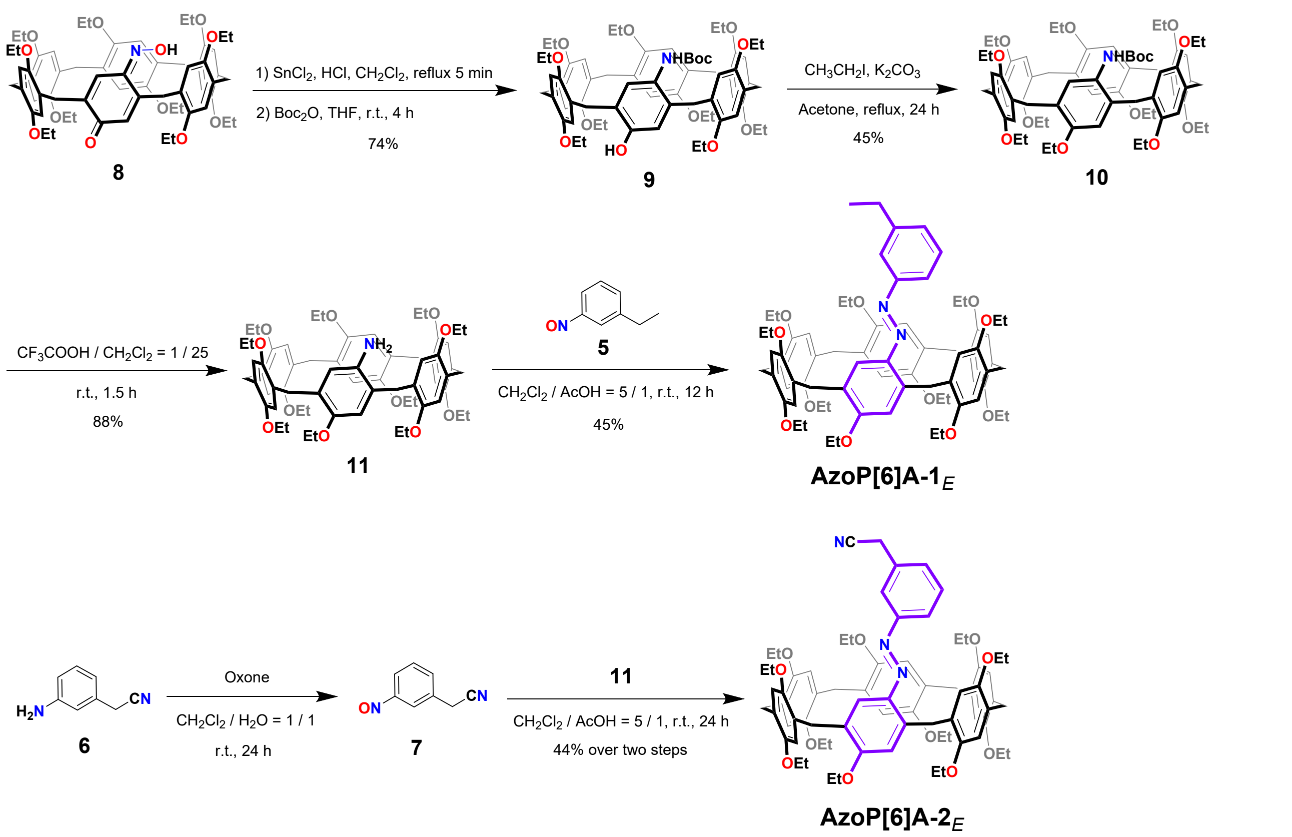

Scheme S2. Synthetic routs for the photoresponsive pillar[6]arenes **AzoP[6]A-1** and **AzoP[6]A-2**.

Compound 9: To the solution of compound **8**[S3] (500 mg, 0.487 mmol) in dichloromethane (35 mL) was added a solution of $SnCl_2$•$2H_2O$ (1.10 g, 4.86 mmol) in concentrated hydrochloric acid (1.8 mL). The mixture was heated up to reflux under a nitrogen atmosphere for 5 minutes, with the TLC confirming the consumption of starting material. The reaction mixture was cooled to room temperature and adjusted to pH = 7~8 by the addition of saturated $NaHCO_3$ aqueous solution. The organic phase was separated and washed with saturated bine (3 × 20 mL) and dried over anhydrous Na_2SO_4. The desiccant was further removed by filtration, and the collected filtrate was concentrated *via* evaporation under reduced pressure to afford a yellow solid. The obtained yellow solid was then dissolved in anhydrous THF (15 mL), to which Boc_2O (120 mg, 0.550 mmol) was added, and the mixture was stirred at room temperature under a nitrogen atmosphere for another 4 hours. After TLC confirmed the reaction was completed, the solvent was removed by evaporation under reduced pressure. The resulting crude product was purified by flash column chromatography using a mixture of EtOAc/PE = 1/15 as the eluent to afford compound **9** as a pale-yellow solid (400 mg, 74%). ^{1}H NMR (400 MHz, $CDCl_3$, 298 K) δ (ppm): 7.55 (br, 2 H), 7.48 (s, 1 H), 6.87 (s, 1 H), 6.82 (s, 1H), 6.78 (s, 1 H), 6.76 (s, 1 H), 6.73-6.72 (m, 3 H), 6.69 (s, 1 H), 6.68 (s, 1 H), 6.66 (s, 1 H), 6.65 (s, 1 H), 4.07 (q, J = 7.2 Hz, 2 H), 3.99-3.93 (m, 6 H), 3.91-3.79 (m, 22 H), 3.71 (s, 2 H), 1.57 (s, 9 H), 1.49 (t, J = 6.8 Hz, 3 H), 1.44-1.38 (m, 9 H), 1.35- 1.27 (m, 22 H). ^{13}C NMR (150 MHz, $CDCl_3$, 298 K) δ (ppm): 154.20, 151.62, 151.00, 150.57, 150.54, 150.34, 148.94, 147.81, 131.85, 129.11, 128.97, 128.43, 128.31, 128.29, 128.05, 127.91, 127.49, 127.16, 126.78, 126.69, 125.30, 124.48, 117.77,

115.70, 115.56, 115.50, 115.41, 115.18, 115.04, 115.01, 114.74, 114.57, 114.33, 79.43, 65.03, 64.37, 64.35, 64.31, 64.30, 64.22, 64.10, 64.05, 63.80, 31.72, 30.99, 30.95, 30.87, 30.76, 30.71, 28.53, 15.21, 15.19, 15.16, 15.12, 15.04, 14.65. HRMS (ESI) Calcd. for $C_{67}H_{85}NNaO_{13}$ $[M+Na]^+$: 1134.5914, Found: 1134.5920.

Compound 10: To a solution of compound **9** (100 mg, 0.0899 mmol) and iodoethane (14.0 mg, 0.0900 mmol) in 2 mL acetone was added K_2CO_3 (62.0 mg, 0.449 mmol), the mixture was heated up to reflux under a nitrogen atmosphere for 24 hours. After naturally cooling down to room temperature, the reaction mixture was filtrated, and the collected filtrate was concentrated *via* evaporation under reduced pressure to give a white solid. It was dissolved in 50 mL CH_2Cl_2 and sequentially washed by water (3 × 50 mL) and saturated brine (3 × 50 mL), respectively. The organic phase was dried over anhydrous Na_2SO_4, which was further removed by filtration, and the collected filtrate was concentrated *via* evaporation under reduced pressure. The resulting crude product was purified by flash column chromatography using a mixture of EtOAc/PE = 1/15 as the eluent to afford compound **10** as a white solid (46.1 mg, 45%). ^1H NMR (600 MHz, $CDCl_3$, 298 K) δ (ppm): 7.53, (br, 1 H), 7.06 (br, 1 H), 6.78 (s, 1 H), 6.77 (s, 1 H), 6.74 (s, 1 H), 6.73-6.71 (m, 4 H), 6.69-6.67 (m, 4 H), 3.96-3.76 (m, 34 H), 1.49 (br, 9 H), 1.40-1.36 (m, 9 H), 1.34-1.28 (m, 24 H). ^{13}C NMR (150 MHz, $CDCl_3$, 298 K) δ (ppm): 153.98, 153.54, 150.87, 150.68, 150.58, 1580.53, 150.51, 150.48, 150.31,149.50, 131.02, 129.05, 128.40, 128.33, 128.13, 128.11, 128.05, 127.85, 127.82, 127.44, 126.69, 115.59, 115.43, 115.35, 115.31, 115.09, 114.93, 114.37, 113.51, 79.27, 64.35, 64.34, 64.30, 64.26, 64.20, 64.18, 64.16, 64.11, 64.03, 64.00, 63.82, 32.71, 30.97, 30.91, 30.78, 30.62, 30.34, 28.39, 15.24, 15.20, 15.15, 15.13, 15.09, 14.99. HRMS (ESI) Calcd. for $C_{69}H_{89}NNaO_{13}$ $[M+Na]^+$: 1162.6227, Found: 1162.6220.

Compound 11: Compound **10** (100 mg, 0.0877 mmol) was dissolved in a mixture of dichloromethane (7.5 mL) and trifluoroacetic acid (0.30 mL), and the mixture was stirred at room temperature under a nitrogen atmosphere for 1.5 hours. After the consumption of starting material was confirmed by TLC, the reaction mixture was concentrated *via* evaporation under reduced pressure to afford a white solid. The obtained solid was dissolved in dichloromethane (20 mL), and sequentially washed with saturated $NaHCO_3$ aqueous solution (3 × 10 mL) and saturated brine (3 × 10 mL). The organic phase was collected and dried over anhydrous Na_2SO_4, which was filtered off and the filtrate was concentrated *via* evaporation under reduced pressure. The resulting crude product was purified by flash column chromatography using a mixture solvent of EtOAc/PE = 1/10 as the eluent, to afford compound **11** as a white solid (80.2 mg, 88%). ^1H NMR (400 MHz, DMSO-d_6, 298 K) δ (ppm): 6.77 (s, 1 H), 6.70 (br, 2 H), 6.67 (s, 1 H), 6.65 (s, 1 H), 6.63 (br, 2 H), 6.60 (s, 1 H), 6.57 (s, 1 H), 6.51 (s, 1 H), 6.19 (s, 1 H), 4.00 (br, 2 H), 3.88-3.67 (m, 30 H), 3.61 (s, 2 H), 3.58 (s, 2 H), 1.25-1.12 (m, 33 H). ^{13}C NMR (150 MHz, $CDCl_3$, 298 K) δ (ppm):

151.00, 150.61, 150.52, 150.51, 150.47, 150.43, 150.39, 150.22, 149.70, 149.57, 138.07, 129.52, 128.55, 128.24, 128.11, 127.85, 127.84, 127.82, 127.75, 127.72, 127.70, 126.35, 124.03, 118.32, 116.06, 116.03, 115.70, 115.47, 115.41, 115.28, 115.16, 114.99, 114.85, 113.24, 64.98, 64.47, 64.31, 64.16, 64.00, 63.99, 63.95, 63.90, 63.79, 32.19, 31.17, 31.14, 30.97, 30.29, 29.57, 15.29, 15.25, 15.21, 15.18, 15.08, 15.06, 15.00. HRMS (ESI) Calcd. for $C_{64}H_{82}NO_{11}$ $[M+H]^+$: 1040.5883, Found: 1040.5878.

Compound AzoP[6]A-1*E*: Compound **11** (80.0 mg, 0.0769 mmol) and compound **5**[S2] (20.0 mg, 0.148 mmol) were dissolved in a mixed solvent of acetic acid (1 mL) and dichloromethane (5 mL). The reaction mixture was stirred at room temperature under a nitrogen atmosphere for 12 hours, when TLC confirmed the consumption of starting material. Saturated $NaHCO_3$aqueous solution was added to adjust the reaction mixture to pH = 8, then, the organic phase was separated and washed with saturated brine (3 × 20 mL) and dried over anhydrous Na_2SO_4. The desiccant was filtered off and the collected filtrate was concentrated *via* evaporation under reduced pressure to afford a yellow solid. The crude product was purified by flash column chromatography using a mixture solvent of EtOAc/PE = 1/25 as the eluent to afford a yellow viscous liquid, which was further suspended in methanol (5 mL) and sonicated for 5 minutes. After filtration, the solid was collected and dried under vacuum to yield compound **AzoP[6]A-1*E*** as an orange-yellow solid (40.3 mg, 45%). ^{1}H NMR (400 MHz, CD_3CN, 298 K) δ (ppm): 7.70 (s, 1 H), 7.67-7.64 (m, 2 H), 7.45 (t, *J* = 7.6 Hz, 1 H), 7.36 (d, *J* = 7.6 Hz, 1 H), 6.99 (s, 1 H), 6.86 (s, 1 H), 6.71 (s, 1 H), 6.68-6.66 (m, 2 H), 6.65 (s, 1 H), 6.62-6.59 (m, 4 H), 6.58 (s, 1 H), 4.38 (s, 2 H), 4.05 (q, *J* = 6.8 Hz, 2 H), 3.94 (q, *J* = 6.8 Hz, 2 H), 3.84-3.68 (m, 26 H0, 3.40 (q, *J* = 6.8 Hz, 2 H), 2.77 (q, *J* = 7.6 Hz, 2 H), 1.39 (t, *J* = 6.8 Hz, 3 H), 1.34-1.11 (m, 30 H), 1.01 (t, *J* = 6.8 Hz, 3 H). ^{13}C NMR (100 MHz, $CDCl_3$, 298 K) δ (ppm): 159.75, 153.71, 150.89, 150.51, 150.46, 150.45, 150.38, 150.34, 150.09, 145.10, 143.73, 141.85, 129.27, 128.79, 128.09, 128.07, 127.97, 127.93, 127.89, 127.81, 127.65, 127.62, 126.88, 122.11, 119.94, 118.81, 115.66, 115.42, 115.34, 115.30, 115.09, 115.05, 114.99, 114.49, 112.67, 64.22, 64.11, 64.07, 64.01, 63.89, 63.77, 63.70, 63.22, 31.90, 31.36, 30.96, 30.91, 30.70, 30.68, 28.85, 15.57, 15.30, 15.24, 15.17, 15.15, 14.97, 14.92. HRMS (ESI) Calcd. for $C_{72}H_{89}N_2O_{11}$ $[M+H]^+$: 1157.6461, Found: 1157.6453.

Compound **AzoP[6]A-2*E***. To a solution of compound **6** (27.2 mg, 0.274 mmol) in 6 mL dichloromethane was added an aqueous solution of Oxone (252 mg, 0.411 mmol, in 6 mL H_2O), the biphasic mixture was stirred at room temperature for 24 hours. After TLC indicated the complete consumption of starting material, the reaction mixture was transferred to a separatory funnel to separate the organic phase, which was washed sequentially with 4 mL hydrochloric acid aqueous solution (HCl_{aq}, 1.0 mol/L), saturated $NaHCO_3$ aqueous solution (10 mL) and saturated brine (10 mL), respectively. The combined organic phase was dried over anhydrous Na_2SO_4, which was further removed by filtration. The filtrate was

collected and concentrated *via* evaporation under reduced pressure, affording compound **7** as a brown liquid and directly used in the next step without further purification.

The recieved crude compound **7** was added into a solution of compound **11** (80.0 mg, 0.0769 mmol) in a mixture of glacial acetic acid and dichloromethane (1/5, v/v, 6 mL total volume), the reaction mixture was stirred at room temperature under a nitrogen atmosphere for 12 hours. When TLC confirmed the complete consumption of the starting material, the reaction was quenched by careful addition of saturated $NaHCO_3$ aqueous solution to turn the mixture to pH = 8. Then, the reaction mixture was transfered to a separatory funnel for phase sepration, and the organic phase was washed with saturated brine (3 × 20 mL). The combined organic phase was dried over anhydrous Na_2SO_4, which was further removed by filtration, and the collected filtrate was concentrated *via* evaporation under reduced pressure. The resulting crude product was purified by flash chromatography using a mixture eluent of PE/EtOAc = 25/1 (v/v), affording a yellow viscous liquid, which was further suspended in methonal (5 mL) and ultrasonicated for 5 minutes. The precipitated solid was collected by filtration, and dried under vacuum to yield compound **AzoP[6]A-2**$_E$ as an orange-yellow solid (39.5 mg, 44%). ^{1}H NMR (600 MHz, $CDCl_3$, 298 K) δ (ppm): 7.83-7.82 (m, 2 H), 7.78 (s, 1 H), 7.50 (t, *J* = 7.6 Hz, 1 H), 7.40 (d, *J* = 7.6 Hz, 1 H), 6.77 (s, 1 H), 6.76 (s, 1 H), 6.72 (s, 1 H), 6.70 (s, 1 H), 6.66-6.65 (m, 3 H), 6.64 (br, 2 H), 6.63 (s, 1 H), 6.60 (s, 1 H), 4.40 (s, 2 H), 3.93-3.75 (m, 32 H), 3.49 (q, *J* = 7.2 Hz, 2 H), 1.39-1.33 (m, 9 H), 1.30-1.22 (m, 21 H), 1.10 (t, *J* = 6.6 Hz, 3 H). ^{13}C NMR (150 MHz, $CDCl_3$, 298 K) δ (ppm): 160.27, 153.95, 150.85, 150.50, 150.47, 150.42, 150.40, 150.37, 150.10, 143.58, 142.46, 130.85, 129.73, 128.68, 128.22, 128.14, 128.11, 127.94, 127.85, 127.78, 127.65, 127.54, 126.72, 122.30, 122.09, 118.82, 117.57, 115.57, 115.44, 115.40, 115.30, 115.23, 115.16, 115.12, 115.07, 114.57, 112.57, 64.17, 64.13, 64.10, 64.06, 64.02, 64.00, 63.99, 63.95, 63.93, 63.67, 63.25, 31.95, 31.40, 30.97, 30.95, 30.72, 30.63, 23.60, 15.27, 15.17, 15.15, 15.14, 15.13, 15.11, 15.01, 14.84. HRMS (ESI) Calcd. for $C_{72}H_{86}N_3O_{11}$ $[M+H]^+$: 1168.6257, Found:1168.6249.

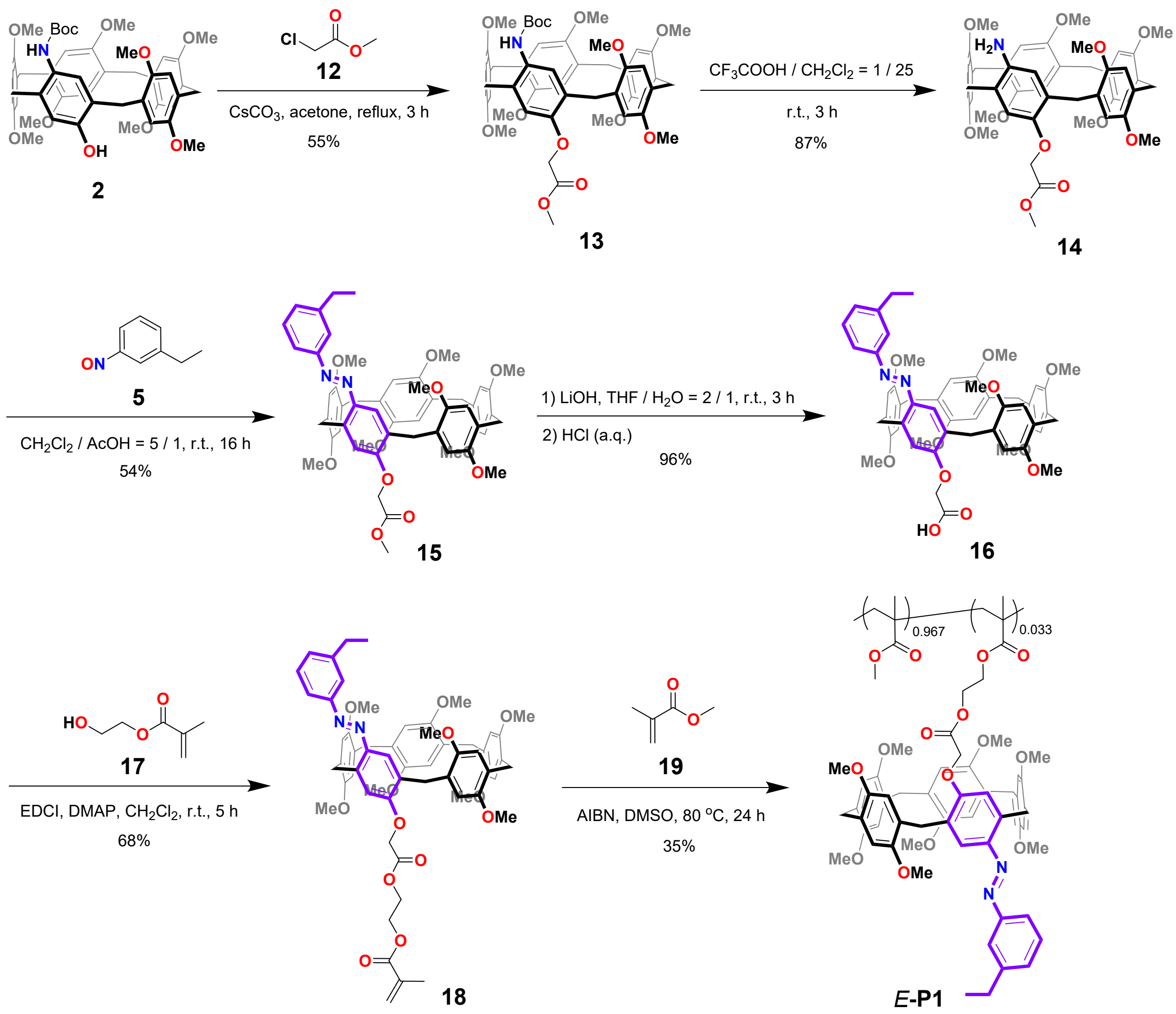

Scheme S3. Synthetic rout for the polymer **P1**.

Compound 13: To a mixture of compound **2** (100 mg, 0.117 mmol) and K_2CO_3 (50.0 mg, 0.153 mmol) in 2 mL acetone was added compound **12** (50.0 mg, 0.463 mmol), the reaction mixture was heated up to reflux for 3 hours. After naturally cooling down to room temperature, the mixture was filtrated, and the collected filtrate was concentrated *via* evaporation under reduced pressure to give a white solid. It was dissolved in 50 mL dichloromethane and sequentially washed by water (10 mL) and saturated brine (10 mL), respectively, and dried over anhydrous Na_2SO_4. The desiccant was further removed by filtration, and the collected filtrate was concentrated *via* evaporation under reduced pressure. The resulting crude product was purified by flash chromatography using a mixture eluent of PE/EtOAc = 1/1 (v/v), affording a yellow viscous liquid, which was further suspended in n-hexane (5 mL) and ultrasonicated for 5 minutes. The precipitated solid was collected by filtration, and dried under vacuum to yield compound **13** as a white solid (58.0 mg, 55%). ^{1}H NMR (400 MHz, DMSO-d_6, 298 K) δ (ppm): 8.28 (s, 1H), 7.04 (s, 1H), 6.92 (s, 1H), 6.86 (s, 1H), 6.82 (d, 2H), 6.77 (s, 1H), 6.76 (s, 2H), 6.74 (d, 2H), 4.78 (s, 2H), 3.75 (s, 3H), 3.72 (s, 2H), 3.69 (s, 3H), 3.68-3.66 (m, 11H), 3.65-3.63 (m, 10H), 3.61 (s, 3H), 3.58 (s, 2H), 3.38 (s, 3H), 1.45 (s, 9H). ^{13}C NMR (100 MHz, $CDCl_3$, 298 K) δ (ppm): 169.71, 153.94, 152.02, 151.36, 151.10,

150.94, 150.83, 150.70, 150.67, 150.59, 149.16, 131.14, 129.22, 129.10, 128.88, 128.63, 128.37, 128.35, 128.26, 128.11, 127.94, 127.14, 125.95, 114.70, 114.52, 114.26, 114.20, 114.12, 113.90, 113.85, 112.93, 112.67, 79.41, 65.80, 56.23, 56.10, 55.96, 55.91, 55.83, 55.82, 55.81, 55.77, 51.07, 31.51, 30.10, 29.59, 28.50. HRMS (ESI) Calcd. for $C_{51}H_{59}NNaO_{13}$ $[M+Na]^+$: 916.3884, Found: 916.3879.

Compound 14: To a solution of compound **13** (40.0 mg, 0.0447 mmol) in dichloromethane (5 mL) was added TFA (0.3 mL), the mixture was stirred at room temperature under a nitrogen atmosphere for 3 hours. After TLC confirmed the consumption of starting material, the reaction mixture was concentrated *via* evaporation under reduced pressure to give a white solid, which was further dissolved in dichloromethane (20 mL) and sequentially washed by saturated $NaHCO_3$ aqueous solution (3 × 10 mL) and saturated brine (3 × 10 mL), respectively. The combined organic phase was dried over anhydrous Na_2SO_4 and the desiccant was further removed by filtration. The collected filtrate was concentrated *via* evaporation under reduced pressure to afford compound **14** a white solid (31.0 mg, 87%). ^1H NMR (400 MHz, DMSO-d_6, 298 K) δ (ppm): 6.94 (s, 1H), 6.86 (s, 1H), 6.82 (s, 1H), 6.79 (s, 1H), 6.78 (s, 1H), 6.77-6.76 (m, 3H), 6.58 (s, 1H), 6.46 (s, 1H), 4.61 (s, 2H), 4.47 (br, 2H), 3.68 (br, 7H), 3.67 (s, 3H), 3.66-3.64 (m, 14H), 3.62-3.60 (m, 8H), 3.54 (s, 2H), 3.37 (s, 3H). ^{13}C NMR (100 MHz, $CDCl_3$, 298 K) δ (ppm): 170.12, 151.28, 150.92, 150.90, 150.86, 150.74, 150.72, 150.71, 150.00, 148.67, 138.12, 129.44, 128.89, 128.50, 128.45, 128.33, 128.26, 127.92, 127.44, 125.33, 119.49, 114.56, 114.53, 114.43, 114.24, 114.21, 114.07, 113.81, 113.19, 66.50, 56.24, 56.07, 55.97, 55.95, 55.86, 55.83, 55.80, 51.31, 30.91, 30.05, 29.81, 29.74, 29.61. HRMS (ESI) Calcd. for $C_{46}H_{52}NO_{11}$ $[M+H]^+$: 794.3540, Found: 794.3534.

Compound 15: Compound **14** (100 mg, 0.126 mmol) and compound **5** (35.0 mg, 0.259 mmol) were dissolved in a mixture solvent of glacial acetic acid (1 mL) and dichloromethane (5 mL), the reaction mixture was stirred at room temperature under a nitrogen atmosphere for 16 hours. When TLC indicated the complete consumption of the starting material, saturated $NaHCO_3$ aqueous solution was carefully added to quench the reaction and turn the mixture to pH = 8. Then, the reaction mixture was transfered to a separatory funnel for phase sepration, and the organic phase was washed with saturated brine (3 × 20 mL). The combined organic phase was dried over anhydrous Na_2SO_4, which was further removed by filtration, and the collected filtrate was concentrated *via* evaporation under reduced pressure. The resulting crude product was purified by flash chromatography using a mixture eluent of PE/EtOAc = 9/1 (v/v), affording a yellow viscous liquid, which was further suspended in n-hexane (2 mL) and ultrasonicated for 5 minutes. The precipitated solid was collected by filtration, and dried under vacuum to yield compound **15** as an orange-yellow solid (62.0 mg, 54%). ^1H NMR (400 MHz, $CDCl_3$, 298 K) δ (ppm): 7.82 (s, 1H), 7.81 (s, 1H), 7.79 (d, J = 8.0 Hz, 1H), 7.45 (t, J = 8.0 Hz, 1H), 7.31 (d, J = 7.2 Hz, 1H), 6.90 (s, 1H), 6.86 (s, 1H), 6.84 (s, 1H), 6.80 (s, 1H), 6.79 (s, 1H), 6.77 (s, 1H), 6.74 (s, 1H), 6.72 (s,

1H), 6.63 (s, 1H), 4.58 (s, 2H), 4.41 (s, 2H), 3.85 (s, 2H), 3.80 (s, 2H), 3.77 (s, 2H), 3.76-3.75 (m, 5H), 3.73 (s, 3H), 3.70 (s, 6H), 3.68 (s, 6H), 3.61 (s, 3H), 3.46 (s, 3H), 2.79 (q, J = 7.6 Hz, 2H), 2.50 (s, 3H), 1.33 (t, J = 7.6 Hz, 3H). ^{13}C NMR (150 MHz, $CDCl_3$, 298 K) δ (ppm): 169.00, 158.19, 153.51, 151.46, 150.83, 150.59, 150.52, 150.51, 150.48, 150.35, 150.33, 145.28, 143.73, 142.73, 129.66, 128.94, 128.71, 128.67, 128.29, 128.26, 128.13, 128.10, 127.88, 127.10, 122.14, 120.00, 118.60, 114.78, 114.28, 114.20, 114.05, 113.79, 113.77, 113.50, 113.36, 111.83, 64.75, 56.07, 55.89, 55.83, 55.75, 55.66, 55.58, 55.53, 51.05, 31.10, 29.74, 29.50, 29.02, 28.94, 28.84, 15.56. HRMS (ESI) Calcd. for $C_{54}H_{59}N_2O_{11}$ $[M+H]^+$: 911.4119, Found: 911.4113.

Compound 16: To a solution of compound **15** (40.0 mg, 0.0439 mmol) in THF (1.4 mL) was dropwise added a solution of LiOH•H_2O (81.0 mg, 1.93 mmol) in water (0.7 mL), the reaction mixture was stirred at room temperature under a nitrogen atmosphere for 3 hours. After TLC confirmed the reaction was completed, aqueous solution of hydrochloric acid (1 M) was added to adjust the reaction mixture to pH = 3~4. Then, the reaction mixture was transfered to a separatory funnel for phase sepration, and the organic phase was washed with saturated brine (3 × 20 mL), and the combined organic phase was dried over anhydrous Na_2SO_4. The desiccant was filtered off, and the collected filtrate was concentrated *via* evaporation under reduced pressure to afford compound **16** as a yellow solid (38.0 mg, crude yield 96%). Note: the obtained compound **16** was used directly in the next step without further purification.

Compound 18: To a solution of compound **16** (55.0 mg, 0.0613 mmol) in dichloromethane (3.0 mL) was dropwise added another solution of DMAP (8.0 mg, 0.065 mmol), EDCI (16.0 mg, 0.0845 mmol) and compound **17** (8.5 mg, 0.0653 mmol) in dichloromethane (1.0 mL) while kept cooling in an ice-bath, the reaction mixture was then stirred at room temperature for 5 hours. After TLC indicated the consumption of starting material, the reaction was quenched by adding dichloromethane (20 mL). The separated organic phase was sequentially washed with aqueous hydrochloric acid (1M, 20 mL), saturated $NaHCO_3$ aqueous solution (20 mL), and saturated brine (20 mL), respectively. The combined organic phase was dried over anhydrous Na_2SO_4, and the desiccant was further filtrate off. The collected filtrate was concentrated *via* evaporation under reduced pressure to afford crude product, which was purified by flash column chromatography using a binary solvent of PE/EtOAc = 10/1 (v/v) as eluent to yield compound **18** as an orange-yellow solid (42.0 mg, 68% yield). ^{1}H NMR (400 MHz, $CDCl_3$, 298 K) δ (ppm): 7.79 (s, 1H), 7.77-7.74 (m, 2H), 7.44 (t, J = 7.6 Hz, 1H), 7.29 (d, J = 7.6 Hz, 1H), 6.90 (s, 2H), 6.89 (s, 1H), 6.81 (s, 1H), 6.75 (s, 2H), 6.72 (s, 1H), 6.65 (s, 1H), 6.53 (s, 1H), 5.68 (s, 1H), 5.36 (s, 1H), 4.57 (s, 2H), 4.39 (s, 2H), 3.83 (s, 2H), 3.78 (s, 2H), 3.77-3.76 (m, 8H), 3.74 (s, 2H), 3.72 (s, 3H), 3.71 (s, 3H), 3.65 (s, 3H), 3.64 (s, 3H), 3.53 (s, 3H), 3.35 (s, 3H), 3.19 (br, 2H), 3.12 (br, 2H), 2.77 (q, J = 7.6 Hz, 2H), 1.32 (t, J = 7.6 Hz, 3H). ^{13}C NMR (150 MHz, $CDCl_3$, 298 K) δ (ppm): 168.29, 166.53, 158.33, 153.51, 151.66,

150.88, 150.62, 150.58, 150.53, 150.33, 150.23, 145.30, 143.81, 142.61, 135.46, 129.66, 128.95, 128.92, 128.46, 128.35, 128.26, 128.07, 127.83, 127.73, 127.66, 126.83, 126.17, 122.25, 119.83, 118.56, 115.59, 114.37, 114.26, 113.74, 113.55, 113.52, 113.48, 113.32, 112.27, 64.65, 62.88, 61.48, 56.22, 55.86, 55.69, 55.65, 55.59, 55.49, 55.31, 55.22, 31.33, 29.71, 29.70, 28.91, 28.82, 28.67, 18.32, 15.51. HRMS (ESI) Calcd. for $C_{59}H_{65}N_2O_{13}$ $[M+H]^+$: 1009.4487, Found: 1009.4480.

Polymer *E*-**P1:** A glass tube was charged with compound **18** (50.0 mg, 0.0496 mmol), methyl methacrylate (**19**) (150 mg, 1.53 mmol), AIBN (0.4 mg, 0.0024 mmol) and anhydrous DMSO (1.5 mL). The reaction system was subjected to three freeze-pump-thaw cycles under liquid nitrogen, after which the tube was allowed to warm up to room temperature and then sealed. The reaction mixture was heated up to 80 °C and stirred for 24 hours. After cooling to room temperature, the reaction solution was added dropwise to chilled diethyl ether (50 mL), precipitating an orange-yellow solid. The resulting solid was dissolved in chloroform (15 mL) and washed with water (3 × 5 mL). The combined organic phase was dried over anhydrous Na_2SO_4, which was further filtrate off, and the collected filtrate was concentrated *via* evaporation under reduced pressure. The residue was dried under vacuum to afford the orange-yellow polymeric solid *E*-**P1** (70.0 mg, 35% yield based on monomer **18**, Mn, GPC = 39.2 kDa).

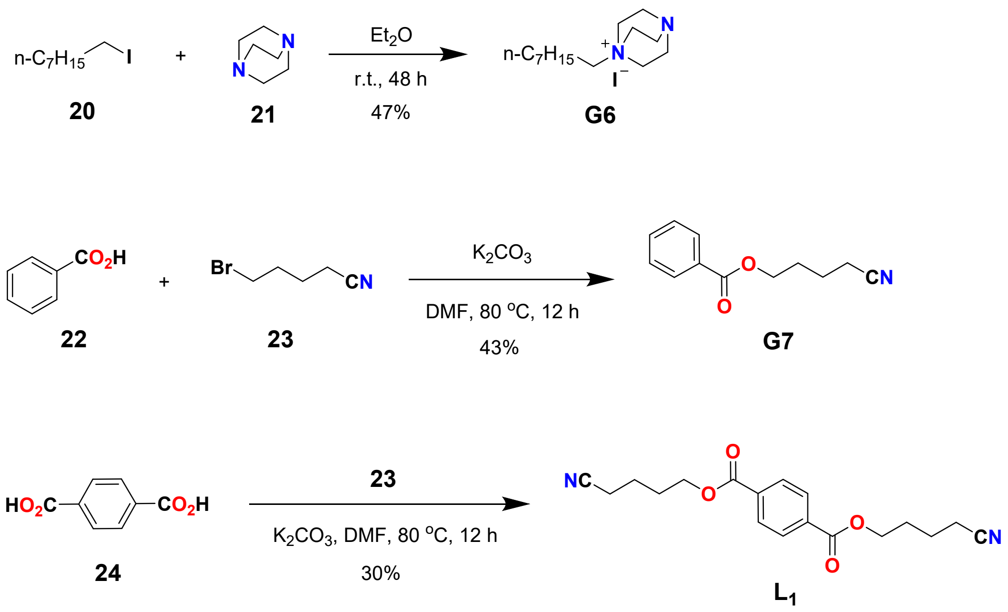

Scheme S4. Synthetic routs for the guests of **G6** and **G7**, as well as the crosslinker **L1**.

Compound G6: Compounds **20** (720 mg, 3.00 mmol) and **21** (336 mg, 3.00 mmol) were dissolved in anhydrous diethyl ether (30 mL), the mixture was stirred at room temperature for 48 hours. The reaction mixture was concentrated *via* evaporation under reduced pressure to remove the solvent, and the resulting residue was dissolved in an appropriate amount of chloroform. Anhydrous diethyl ether was then added dropwise to this solution until turbidity was observed. The dense phase (lower layer) was collected and

dried in a vacuum oven at 45 °C for 2 hours, affording compound **6** as a colorless transparent liquid (501 mg, 47%). ^{1}H NMR (600 MHz, $CDCl_3$, 298 K) δ (ppm): 3.61 (t, *J* = 7.2 Hz, 6 H), 3.44-3.41 (m, 2 H), 3.25 (t, *J* = 7.2 Hz, 6 H), 1.79-1.74 (m, 2 H), 1.37-1.30 (m, 4 H), 1.27-1.19 (m, 6 H), 0.85 (t, *J* = 7.2 Hz, 3 H). ^{13}C NMR (150 MHz, $CDCl_3$, 298 K) δ (ppm): 64.75, 52.54, 45.34, 31.59, 29.09, 29.02, 26.34, 22.54, 22.20, 14.06. HRMS (ESI) Calcd. for $C_{14}H_{29}N$ $[M-I]^+$: 225.2326, Found: 225.2315.

Compound G7: To the solution of compound **22** (500 mg, 4.09 mmol) and anhydrous K_2CO_3 (566 mg, 4.10 mmol) in 5 mL DMF was added compound **23** (531 mg, 3.28 mmol), the reaction mixture was heated up at 80 °C and stirred for 12 hours. After naturally cooling down to room temperature, the reaction mixture was filtered, and the collected filtrate was concentrated *via* evaporation under reduced pressure to give a white solid. The obtained crude solid was dissolved in ethyl acetate (50 mL) and sequentially washed with water (3 × 50 mL) and saturated brine (3 × 50 mL). The combined organic phase was dried over anhydrous Na_2SO_4, which was further filtrate off, and the collected filtrate was concentrated *via* evaporation under reduced pressure. The received crude product was purified by flash column chromatography using dichloromethane as the eluent to afford compound **G7** as a white solid (357 mg, 43%). ^{1}H NMR (400 MHz, $CDCl_3$, 298 K) δ (ppm): 8.06 (d, *J* = 7.6 Hz, 2H), 7.60 (t, *J* = 7.2 Hz, 1H), 7.48 (t, *J* = 7.6 Hz, 2H), 4.40 (t, *J* = 6.0 Hz, 2H), 2.48 (t, *J* = 6.8 Hz, 2H), 2.02-1.95 (m, 2H), 1.91-1.84 (m, 2H).

Compound L1: To the solution of compound **24** (300 mg, 1.81 mmol) and anhydrous K_2CO_3 (497 mg, 3.60 mmol) in 5 mL DMF was added compound **23** (525 mg, 3.24 mmol), the reaction mixture was heated at 80 °C and stirred for 12 hours. After naturally cooling down to room temperature, the reaction mixture was filtered, and the collected filtrate was concentrated *via* evaporation under reduced pressure to give a white solid. The obtained crude solid was dissolved in ethyl acetate (50 mL) and sequentially washed with water (3 × 50 mL) and saturated brine (3 × 50 mL). The combined organic phase was dried over anhydrous Na_2SO_4, which was further filtrate off, and the collected filtrate was concentrated *via* evaporation under reduced pressure. The received crude product was purified by flash column chromatography using dichloromethane as the eluent to afford compound $\mathbf{L_1}$ as a white solid (177 mg, 30%). ^{1}H NMR (600 MHz, $CDCl_3$, 298 K) δ (ppm): 8.13 (s, 4H), 4.43 (t, *J* = 6.0 Hz, 4H), 2.49 (t, *J* = 7.2 Hz, 4H), 2.03-1.98 (m, 4H), 1.91-1.86 (m, 4H). ^{13}C NMR (150 MHz, $CDCl_3$, 298 K) d: 165.60, 133.91, 129.90, 119.19, 64.14, 27.72, 22.31, 16.99. HRMS (ESI) Calcd. for $C_{18}H_{21}N_2O_4$ $[M+H]^+$: 329.1495, Found: 329.1501.

Section 3: Photoswitching Properties of AzoP[5]A-1,2 and AzoP[6]A-1,2

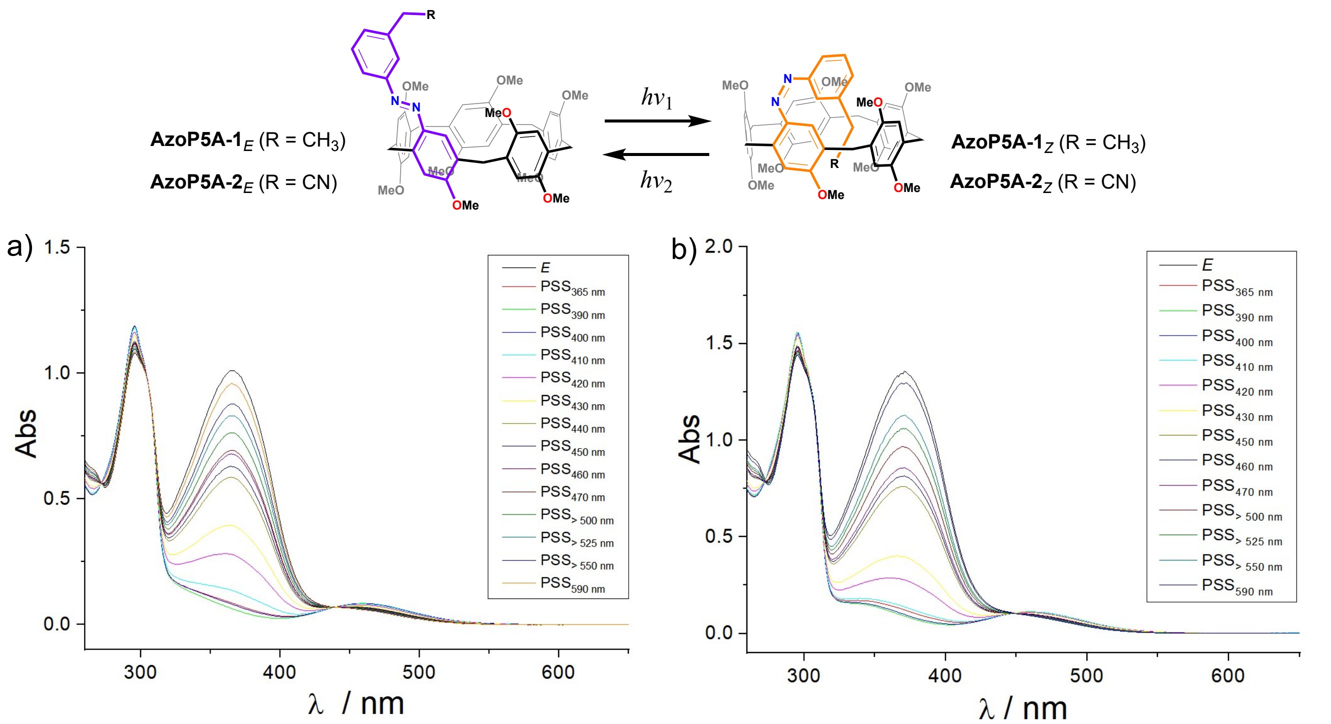

Fig. S1 Schematic representation for the *Z/E* photoisomerization of **AzoP[5]A-1** and **AzoP[5]A-2**, as well as the UV-Vis absorption spectra of a) **AzoP[5]A-1** (0.06 mM), and b) **AzoP[5]A-2** (0.08 mM) in the pristine state and at the PSS after irradiation with various light sources recorded in $CHCl_3$ at 25 ^oC.

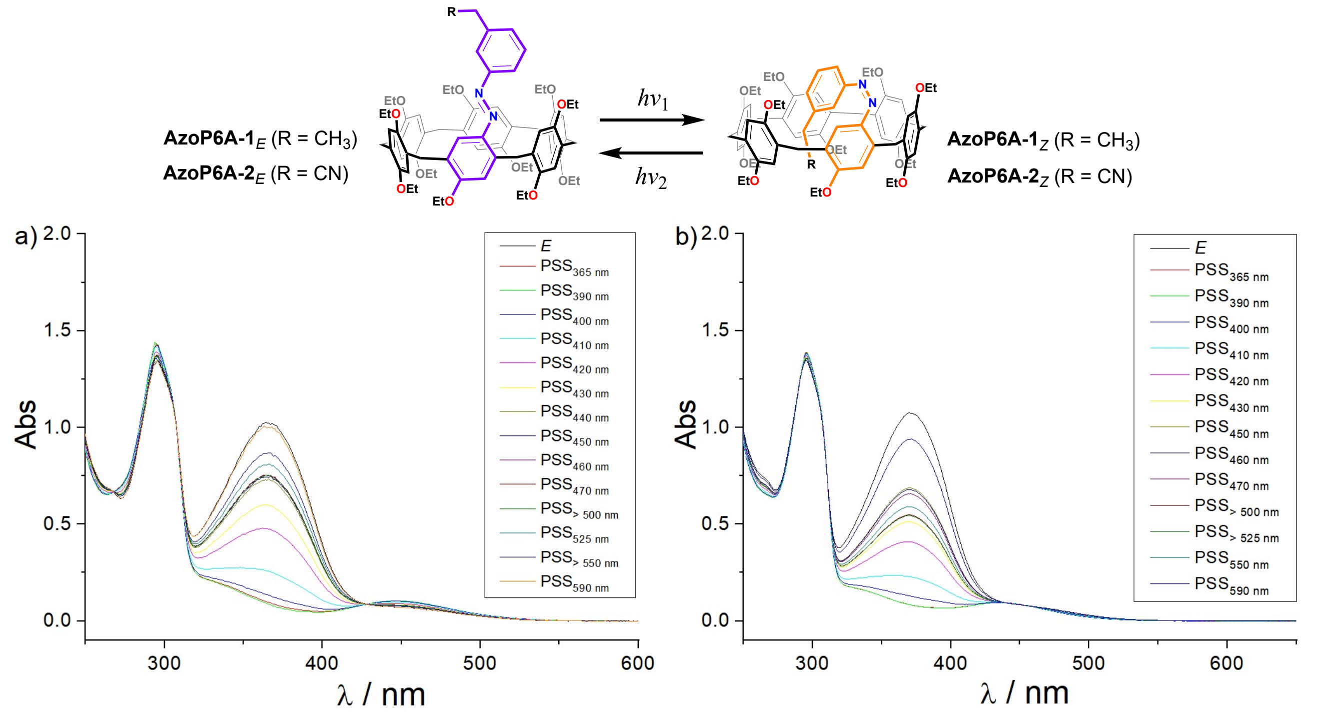

Fig. S2 Schematic representation for the *Z/E* photoisomerization of **AzoP[6]A-1** and **AzoP[6]A-2**, as well as the UV-Vis absorption spectra of a) **AzoP[6]A-1** (0.05 mM), and b) **AzoP[6]A-2** (0.05 mM) in the pristine state and at the PSS after irradiation with various light sources recorded in $CHCl_3$ at 25 ^oC.

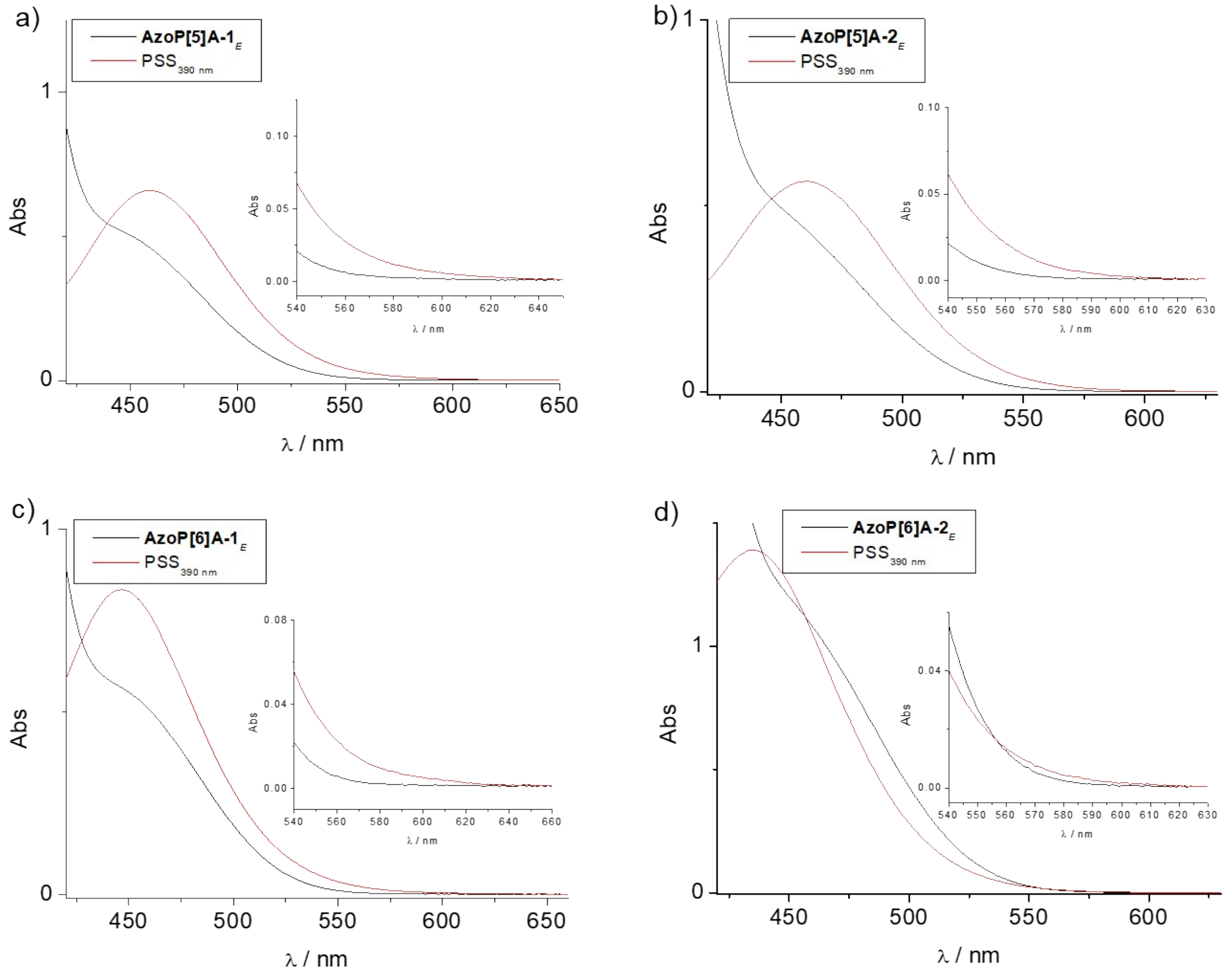

Fig. S3 The UV/Vis absorption spectra of a) **AzoP[5]A-1**$_E$ (0.4 mM) before (black line) and after (red line) irradiation, b) **AzoP[5]A-2**$_E$ (0.4 mM) before (black line) and after (red line), c) **AzoP[6]A-1**$_E$ (0.4 mM) before (black line) and after (red line), and d) **AzoP[6]A-2**$_E$ (0.8 mM) before (black line) and after (red line) irradiation by using 390 nm UV light in $CHCl_3$. Insets show the UV/Vis absorption spectra at $\lambda \geqslant 540$ nm of the solution before and after UV irradiation. The absorption spectra were record at 25 ^oC.

Determination of photochromic quantum yields. Photochromic quantum yields were determined according to the standard procedure described in previous literatures.[S4,S5] Potassium ferrioxalate ($K_3[Fe(C_2O_4)_3]$) was used as actinometer[S6] for the photocyclization quantum yields. Firstly, the light intensity was determined by following steps: i) Prepare 3 mL of a solution of 0.003 M $K_3[Fe(C_2O_4)_3]$ and 0.025 M H_2SO_4; ii) 0.5 mL of phenanthroline solution (0.05 wt% in 0.25 M H_2SO_4/0.8 M NaOAc) was added; iii) measuring the absorbance before and after irradiated for 4 seconds. The light intensity I_0 was calculated *via* equation 1.

$$I_0 = \frac{\Delta A_{510\,nm}}{\Delta t \times \varepsilon_{510\,nm} \times \Phi_{irr} \times 1000} \times \frac{3.5\,mL}{3.0\,mL}$$ Eq. 1

Where $\Delta A_{510\,\mathrm{nm}}$ is the difference value of the absorption at 510 nm before and after irradiation with 390 nm, Δt is the irradiation time, ε_{510} nm is 11100 $M^{-1}cm^{-1}$ and Φ_{irr} is the quantum yield at 390 nm (1.13).

For the determination of the quantum yield equation 2 was used:

$$\Phi = \frac{\Delta A / \Delta t}{(1 - 10^{-A'}) \times \varepsilon \times I_0 \times 1000}$$ Eq. 2

Where $\Delta A/\Delta t$ is the change rate of absorbance upon irradiation at excitation wavelength; ε is the extinction coefficient at observed wavelength; $1\text{-}10^{-A'}$ is the percentage of the absorbed photons by the solution at irradiation wavelength, A′ is the absorbance of *trans* at excitation wavelength; I_0 is the light intensity calculated in Eq. 1.

For the *cis* to *trans* quantum yields, aberchrome 670 was used as an actinometer.[S7] The light intensity was measured as following: i) 3 mL of aberchrome 670 solution (1×10^{-4} M) in toluene was irradiated with 365 nm light for 50 s; ii) irradiate it back with 590 nm light for 40 s; iii) measuring the absorbance at 519 nm before and after the 590 nm light irradiation. The light intensity I_0 was calculated via equation 3.

$$I_0 = \frac{\Delta A_{519\,nm}}{\Delta t \times \varepsilon_{519\,nm} \times \Phi_{irr} \times 1000 \times (1 - 10^{-A'})}$$ Eq. 3

Where $\Delta A_{519\,\mathrm{nm}}$ is the difference value of the absorption at 519 nm before and after irradiation with 590 nm; Δt is the irradiation time; $\varepsilon_{519\,\mathrm{nm}}$ is 7760 $M^{-1}cm^{-1}$; Φ_{irr} is the quantum yield at 590 nm (0.27), and A′ is the absorbance of *cis* at excitation wavelength. The *cis* to *trans* quantum yields were also calculated via equation 2.

Table S1. Photoisomerization quantum yields of different hosts in $CHCl_3$.

Hosts	$\varepsilon_{E(390\,\mathrm{nm})}$	$\varepsilon_{Z(590\,\mathrm{nm})}$	$\Phi_{E\ to\ Z}$ (%)[a]	$\Phi_{Z\ to\ E}$ (%)[b]
**AzoP[5]A-1**	11371	20.1	47	21
**AzoP[5]A-2**	13437	10.8	64	34
**AzoP[6]A-1**	13658	17.8	39	21
**AzoP[6]A-2**	16302	3.8	58	37

[a] 390 nm was used as excitation wavelength for *E* to *Z* photoisomerization. [b] 590 nm was used as excitation wavelength for *Z* to *E* photoisomerization.

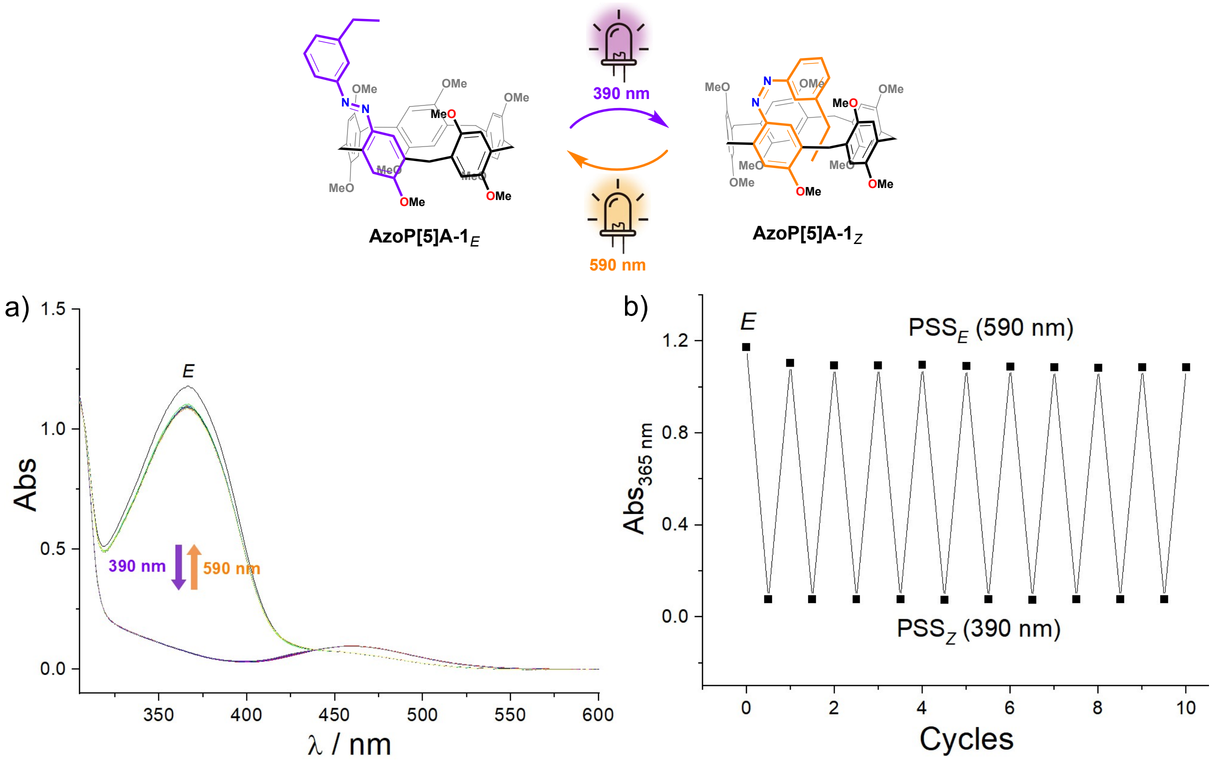

Fig. S4 a) The UV-Vis absorption spectra of **AzoP[5]A-1** (0.07 mM), and b) plot of corresponding absorption λ at 365 nm after 390 nm UV light irradiation, and UV light-irradiated **AzoP[5]A-1** solution after 590 nm yellow light irradiation before next UV irradiation in $CHCl_3$. The absorption spectra were record at 25 °C.

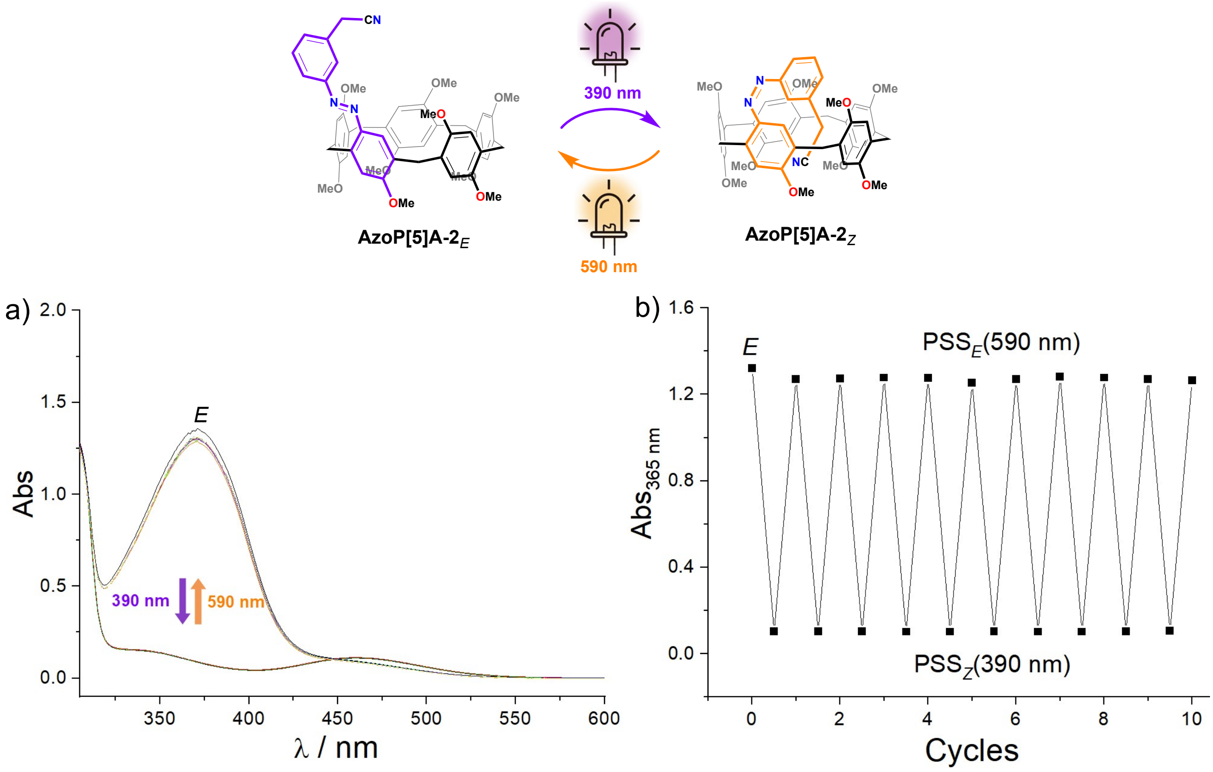

Fig. S5 a) The UV-Vis absorption spectra of **AzoP[5]A-2** (0.08 mM), and b) plot of corresponding absorption λ at

365 nm after 390 nm UV light irradiation, and UV light-irradiated **AzoP[5]A-2** solution after 590 nm yellow light irradiation before next UV irradiation in $CHCl_3$. The absorption spectra were record at 25 °C.

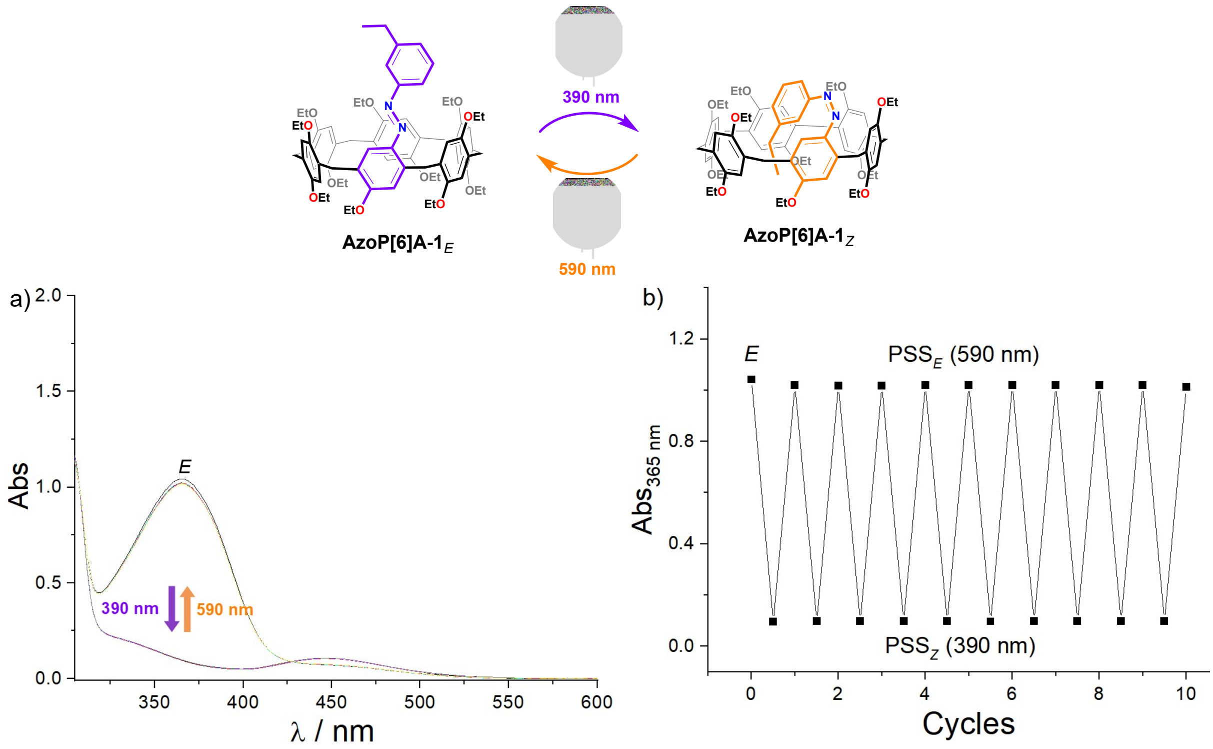

Fig. S6 a) The UV-Vis absorption spectra of **AzoP[6]A-1** (0.05 mM), and b) plot of corresponding absorption λ at 365 nm after 390 nm UV light irradiation, and UV light-irradiated **AzoP[6]A-1** solution after 590 nm yellow light irradiation before next UV irradiation in $CHCl_3$. The absorption spectra were record at 25 °C.

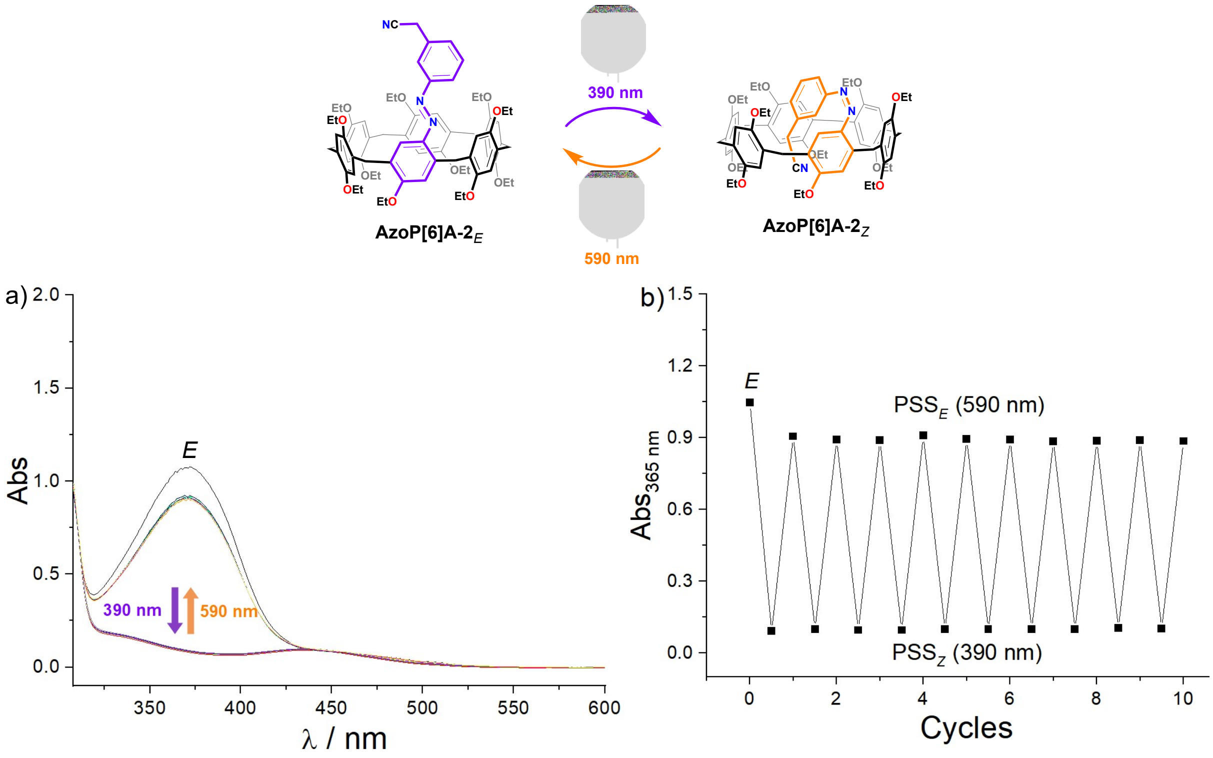

Fig. S7 a) The UV-Vis absorption spectra of **AzoP[6]A-2** (0.05 mM), and b) plot of corresponding absorption λ at 365 nm after 390 nm UV light irradiation, and UV light-irradiated **AzoP[6]A-2** solution after 590 nm yellow light

irradiation before next UV irradiation in $CHCl_3$. The absorption spectra were record at 25 °C.

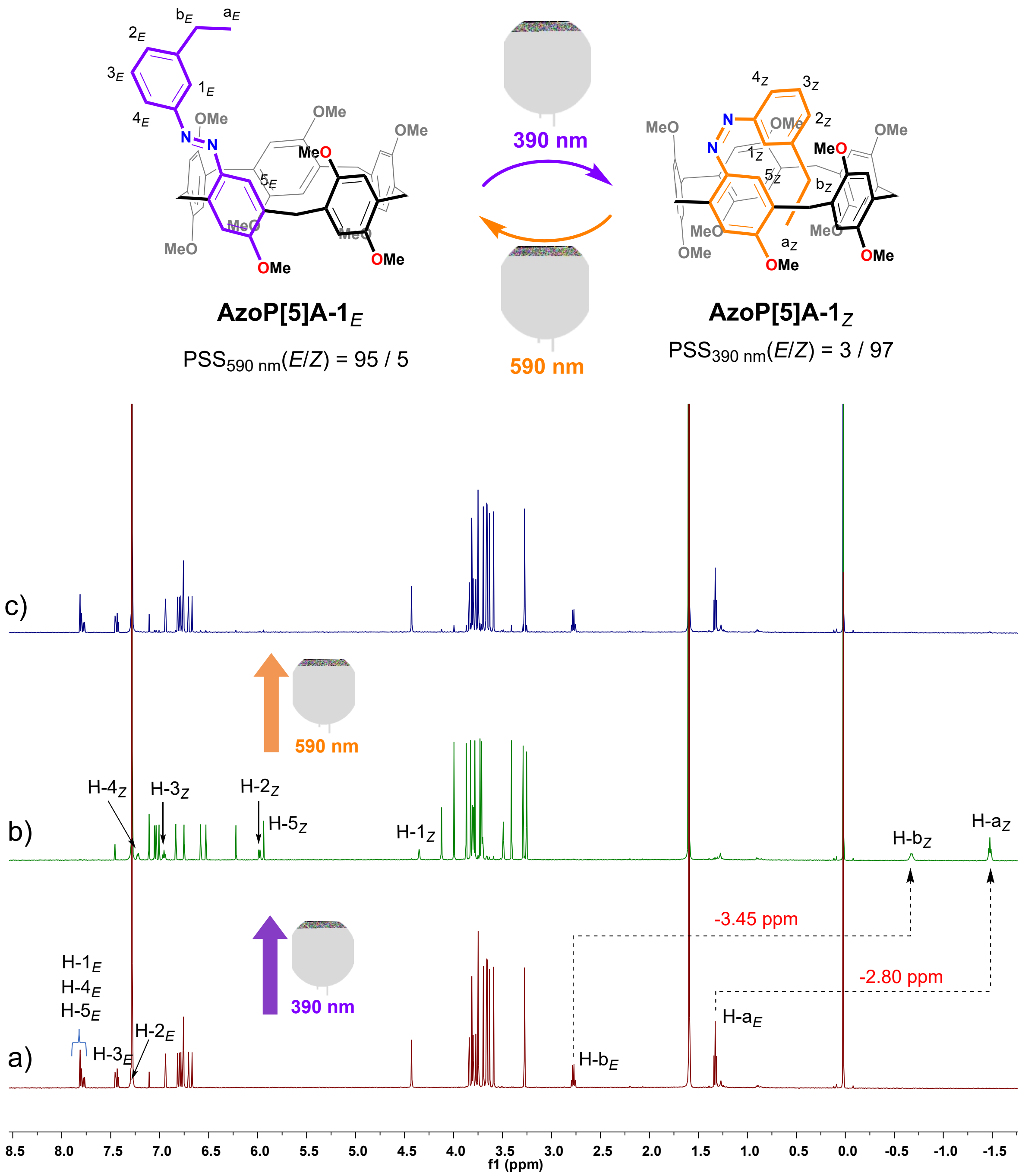

Fig. S8 Schematic illustration of reversible photoinduced self-folding behavior of **AzoP[5]A-1** upon irradiation by 390 nm / 590 nm light irradiation, as well as the partial ^{1}H NMR spectra (600 MHz, $CDCl_3$, 298K) for the solution of a) **AzoP[5]A-1**$_E$ (2.0 mM), b) the PSS$_Z$ (390 nm) mixtures of **AzoP[5]A-1** (2.0 mM), and c) the PSS$_E$ (590 nm) mixtures of **AzoP[5]A-1** (2.0 mM).

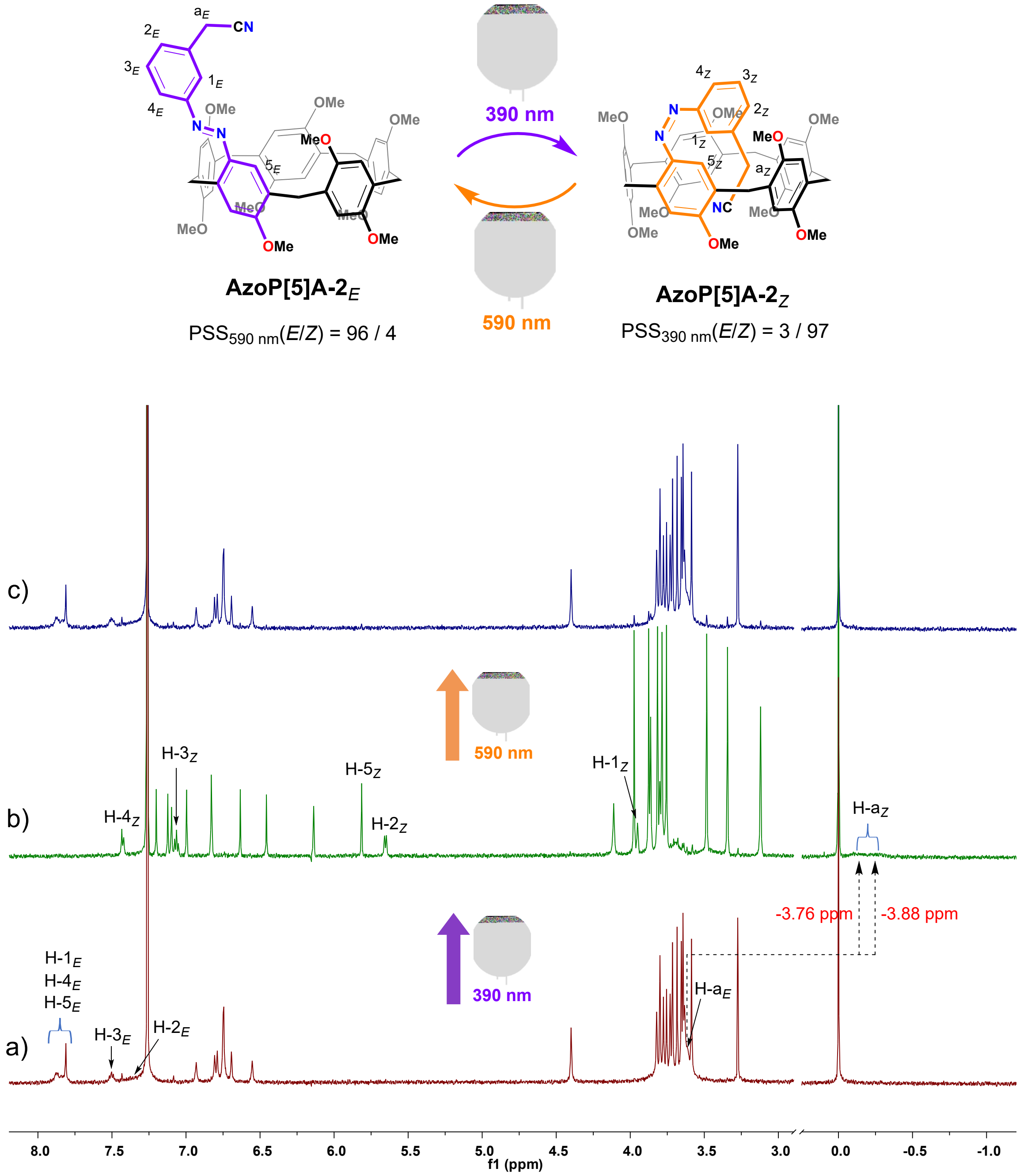

Fig. S9 Schematic illustration of reversible photoinduced self-folding behavior of **AzoP[5]A-2** upon irradiation by 390 nm / 590 nm light irradiation, as well as the partial ^{1}H NMR spectra (600 MHz, $CDCl_3$, 298K) for the solution of a) **AzoP[5]A-2**$_E$ (2.0 mM), b) the PSS$_Z$ (390 nm) mixtures of **AzoP[5]A-2** (2.0 mM), and c) the PSS$_E$ (590 nm) mixtures of **AzoP[5]A-2** (2.0 mM).

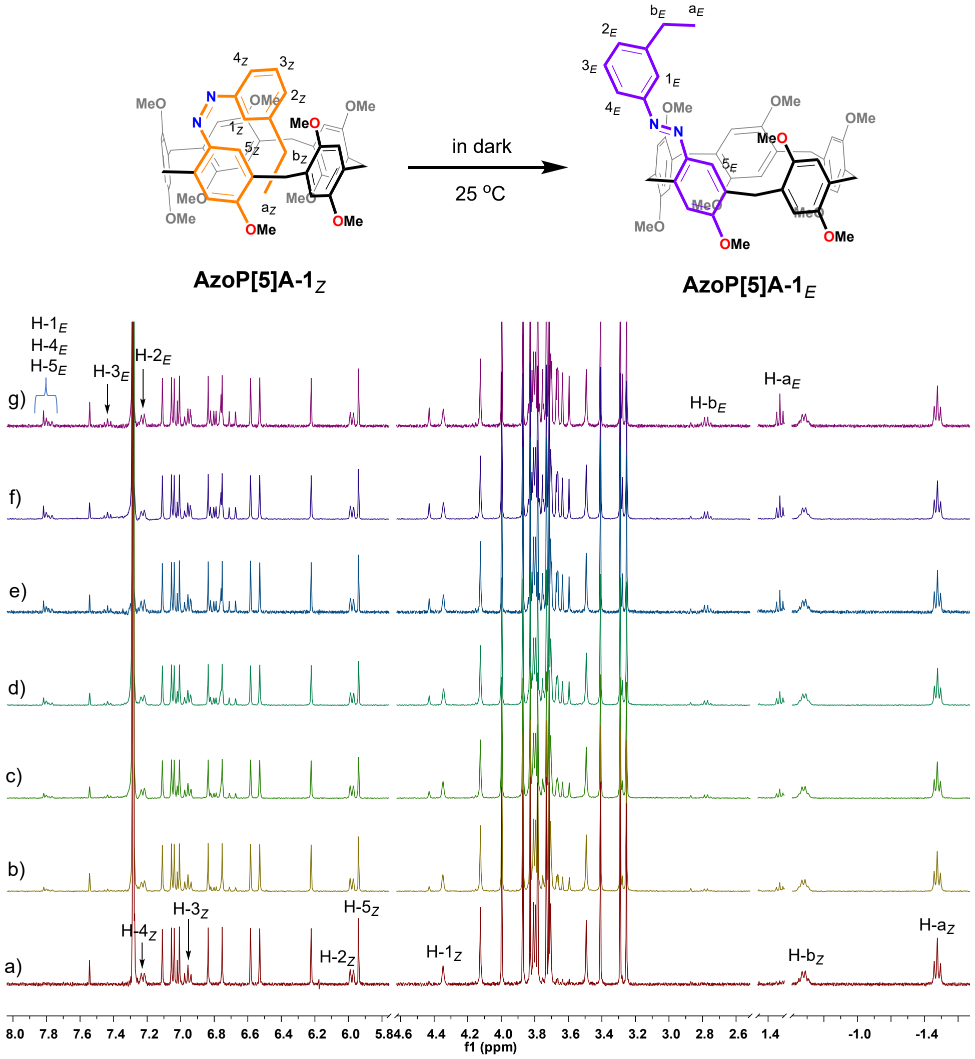

Fig. S10 Schematic representation of the *Z*-to-*E* thermal relaxation behavior of **AzoP[5]A-1**, and partial ^{1}H NMR spectra (400 MHz, 2.0 mM, $CDCl_3$, 298 K) for the solution of the PSS_Z (390 nm) mixtures of **AzoP[5]A-1** under conditions of a) as prepared, and after the rest of b) 16 h 58 min, c) 25 h 22 min, d) 41 h 53 min, e) 49 h 22 min, f) 65 h 43 min, and g) 74 h at 25 ^{o}C in dark.

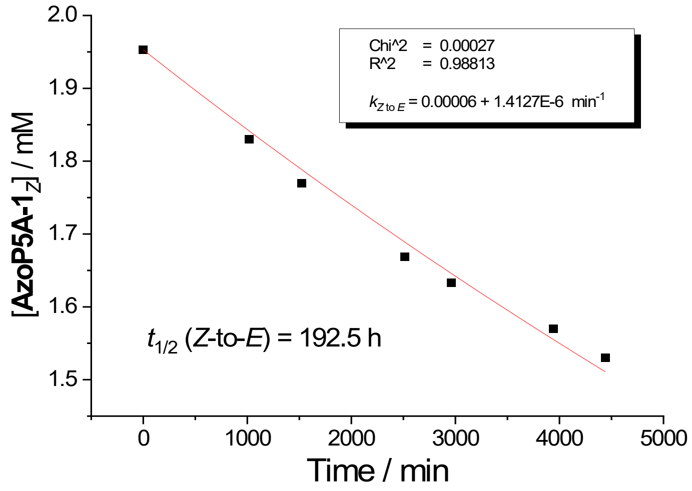

Fig. S11 Time dependent concentration changing plots and the fitting curve of **AzoP[5]A-1**$_Z$ at 25 ^{o}C in dark based on the data of Fig. S10.

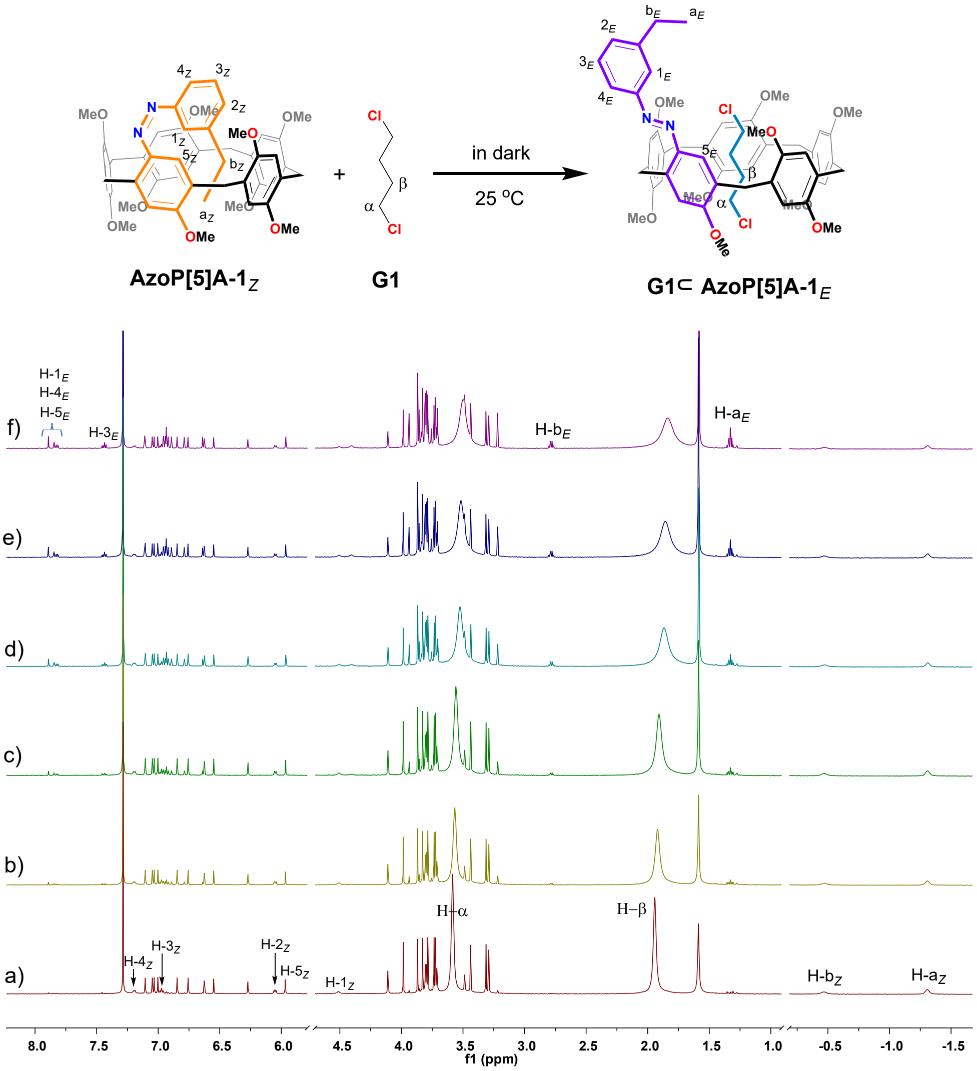

Fig. S12 Schematic representation of the *Z*-to-*E* thermal relaxation behavior of **AzoP[5]A-1** in the presence of 10 equiv. of **G1**, and partial ^{1}H NMR spectra (600 MHz, $CDCl_3$, 298 K) for the solution of the PSS_Z (390 nm) mixtures of **AzoP[5]A-1** (2.0 mM) after addition of 10 equiv. of **G1** and rest for a) 0 min, b) 4 h 39 min, c) 8 h 26 min, d) 26 h 32 min, e) 34 h 7 min, and f) 47 h 48 min at 25 ^{o}C in dark.

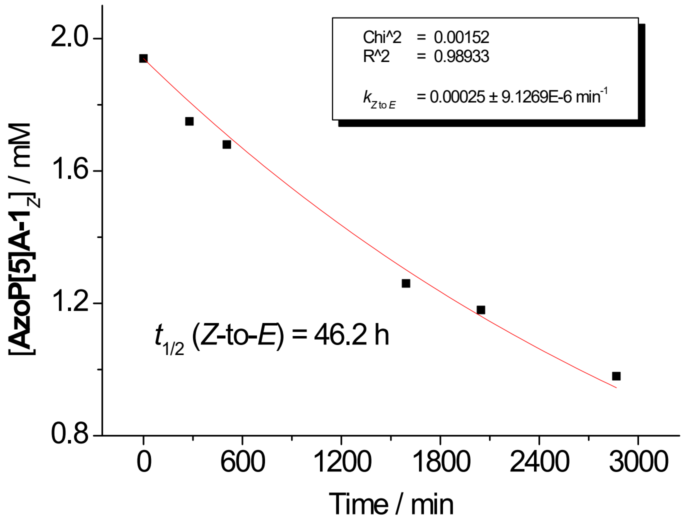

Fig. S13 Time dependent concentration changing plots and the fitting curve of **AzoP[5]A-1**$_Z$ in the presence of **G1** at 25 ^{o}C in dark based on the data of Fig. S12.

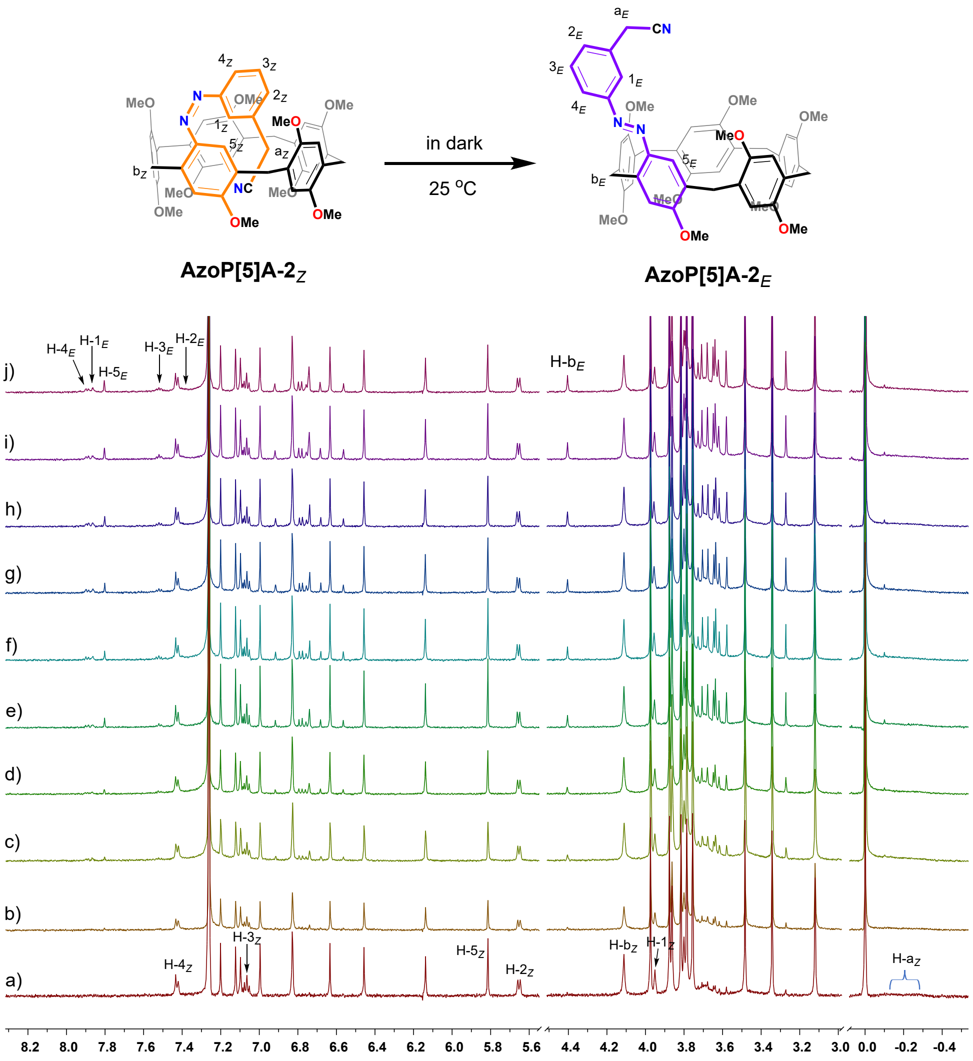

Fig. S14 Schematic representation of the *Z*-to-*E* thermal relaxation behavior of **AzoP[5]A-2**, and partial ^{1}H NMR spectra (400 MHz, 2.0 mM, $CDCl_3$, 298 K) for the solution of the PSS_Z (390 nm) mixtures of **AzoP[5]A-2** under conditions of a) as prepared, and after the rest of b) 2 days and 3 hours, c) 5 days and 5 hours, d) 8 days and 4 hours, e) 13 days and 3 hours, f) 16 days and 9 hours, g) 19 days and 2 hours, h) 22 days and 3 hours, i) 27 days and j) 29 days and 23 hours at 25 °C in dark.

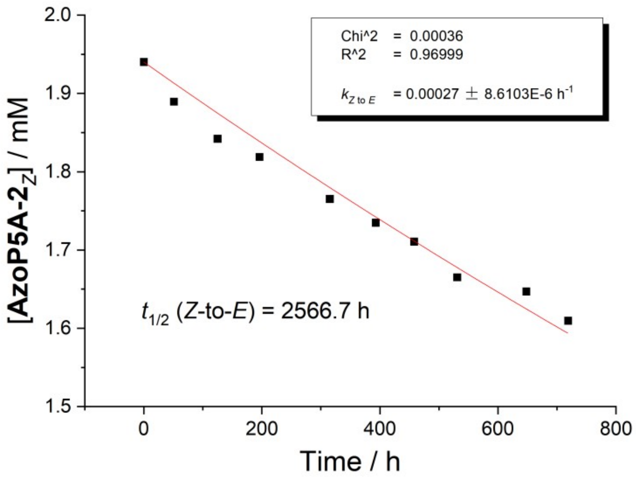

Fig. S15 Time dependent concentration changing plots and the fitting curve of **AzoP[5]A-2**$_Z$ at 25 °C in dark based on the data of Fig. S14.

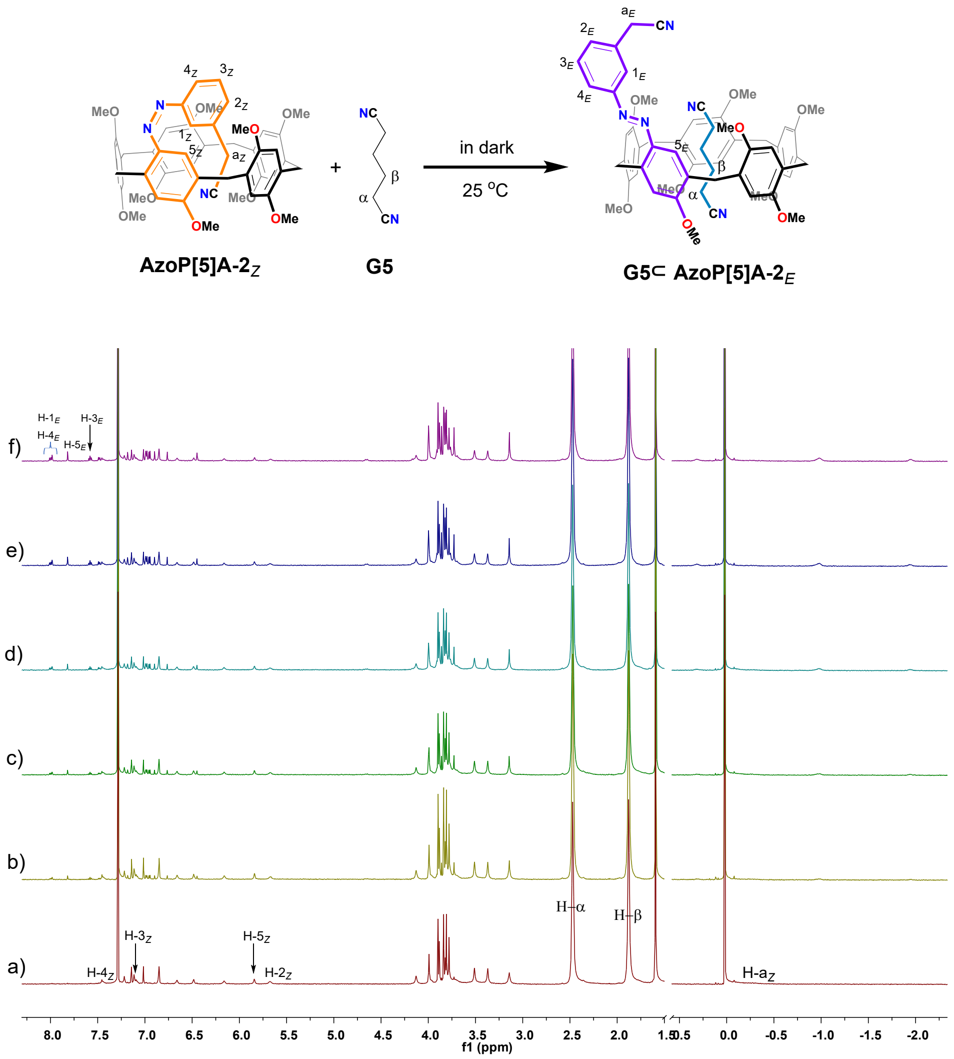

Fig. S16 Schematic representation of the *Z*-to-*E* thermal relaxation behavior of **AzoP[6]A-2** in the presence of 10 equiv. of **G5**, and partial ^{1}H NMR spectra (600 MHz, $CDCl_3$, 298 K) for the solution of the PSS_Z (390 nm) mixtures of **AzoP[6]A-2** (2.0 mM) after addition of 10 equiv. of **G5** for a) 0 min, b) 24 h, c) 48 h, d) 72 h, e) 103 h, and f) 127 h at 25 °C in dark.

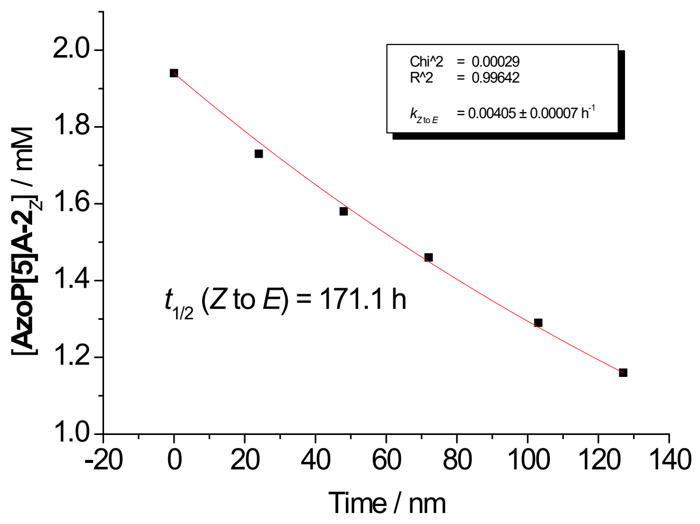

Fig. S17 Time dependent concentration changing plots and the fitting curve of **AzoP[5]A-2**$_Z$ in the presence of **G5** at 25 °C in dark based on the data of Fig. S16.

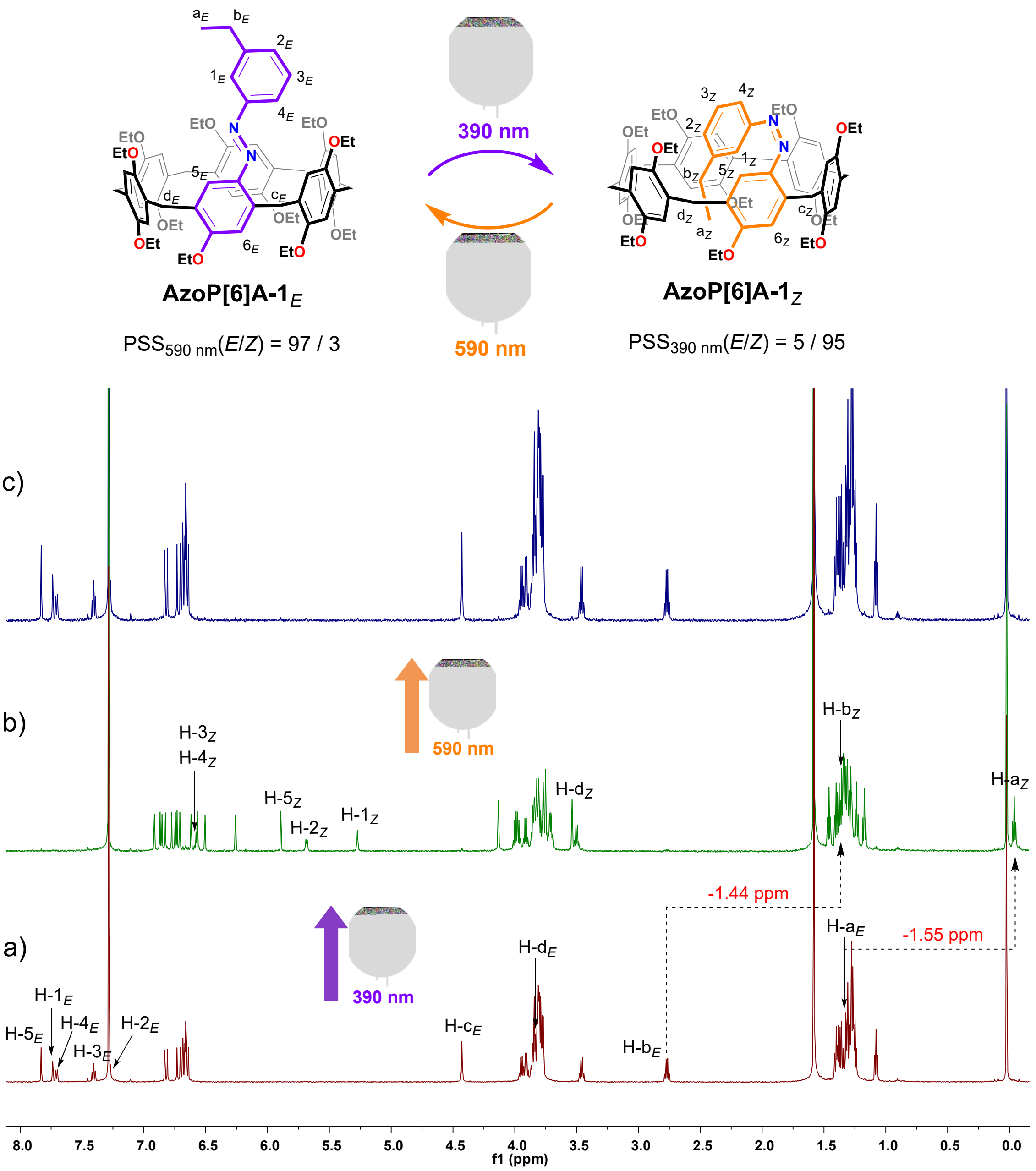

Fig. S18 Schematic illustration of reversible photoinduced self-folding behavior of **AzoP[6]A-1** upon irradiation by 390 nm / 590 nm light irradiation, as well as the partial ^{1}H NMR spectra (600 MHz, $CDCl_3$, 298K) of the solution of (a) **AzoP[6]A-1**$_E$ (2.0 mM), (b) the PSS_Z (390 nm) mixtures of **AzoP[6]A-1** (2.0 mM), and (c) the PSS_E (590 nm) mixtures of **AzoP[6]A-1** (2.0 mM).

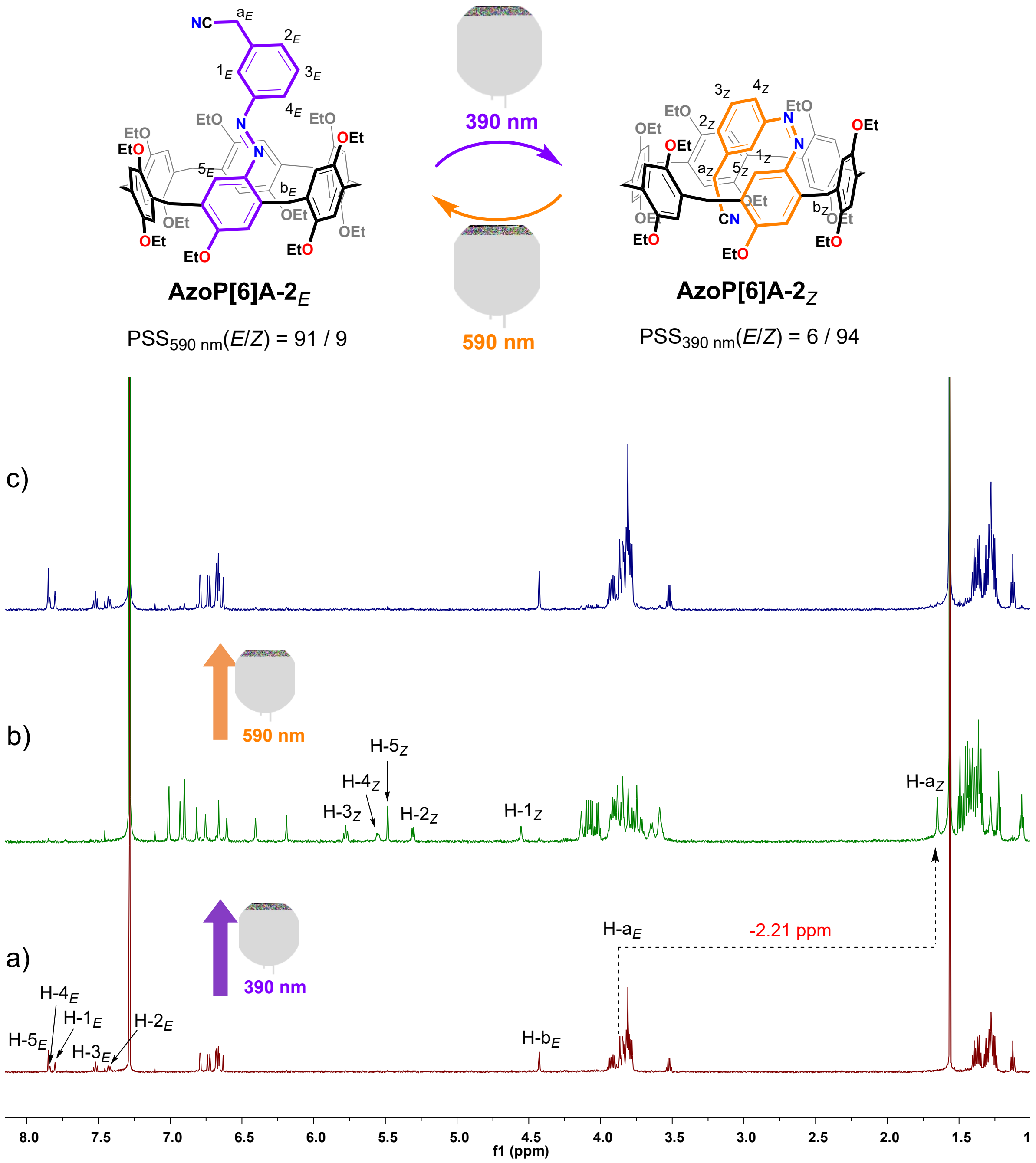

Fig. S19 Schematic illustration of reversible photoinduced self-folding behavior of **AzoP[6]A-2** upon irradiation by 390 nm / 590 nm light irradiation, as well as the partial ^{1}H NMR spectra (600 MHz, $CDCl_3$, 298K) of the solution of (a) **AzoP[6]A-2**$_E$ (2.0 mM), (b) the PSS$_Z$ (390 nm) mixtures of **AzoP[6]A-2** (2.0 mM), and (c) the PSS$_E$ (590 nm) mixtures of **AzoP[6]A-2** (2.0 mM).

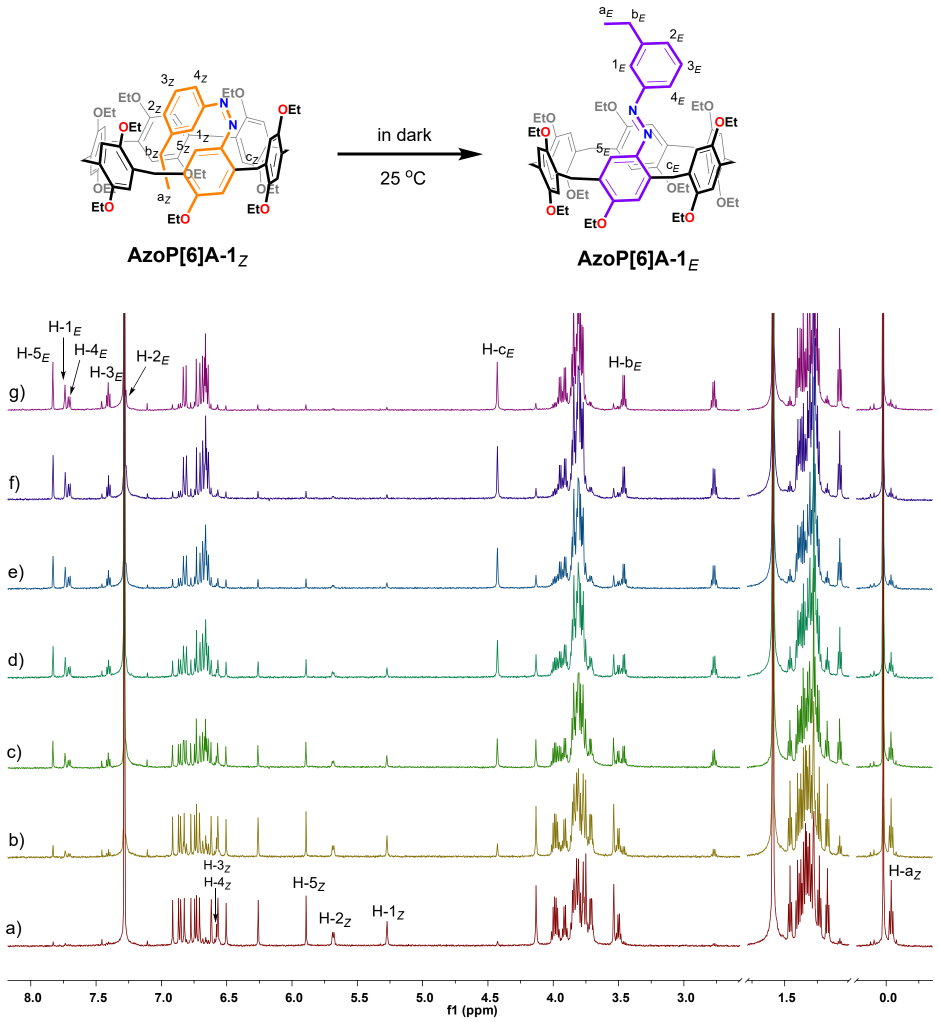

Fig. S20 Schematic representation of the *Z*-to-*E* thermal relaxation behavior of **AzoP[6]A-1**, and partial ^{1}H NMR spectra (600 MHz, 2.0 mM, $CDCl_3$, 298 K) for the solution of the PSS_Z (390 nm) mixtures of **AzoP[6]A-1** under conditions of a) as prepared, and after the rest of b) 5 hours and 3 min, c) 20 hours and 36 min, d) 29 hours and 32 min, e) 44 hours and 37 min, f) 58 hours and 27 min, and g) 68 hours and 44 min at 25 °C in dark.

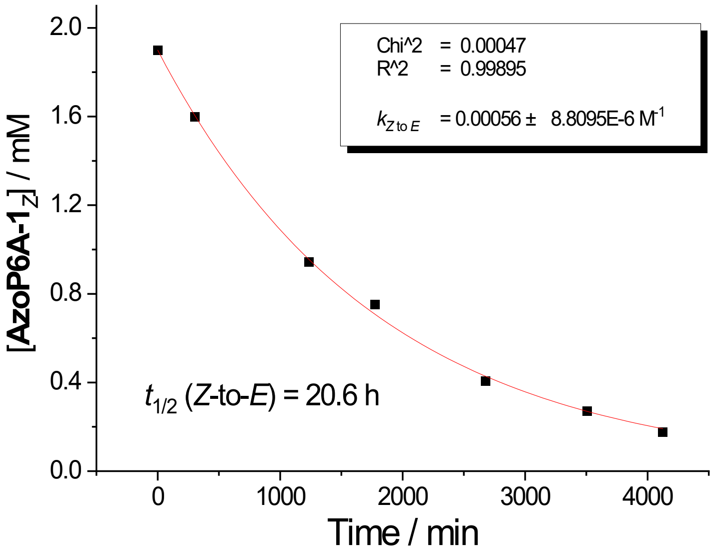

Fig. S21 Time dependent concentration changing plots and the fitting curve of **AzoP[6]A-1**$_Z$ at 25 °C in dark based on the data of Fig. S20.

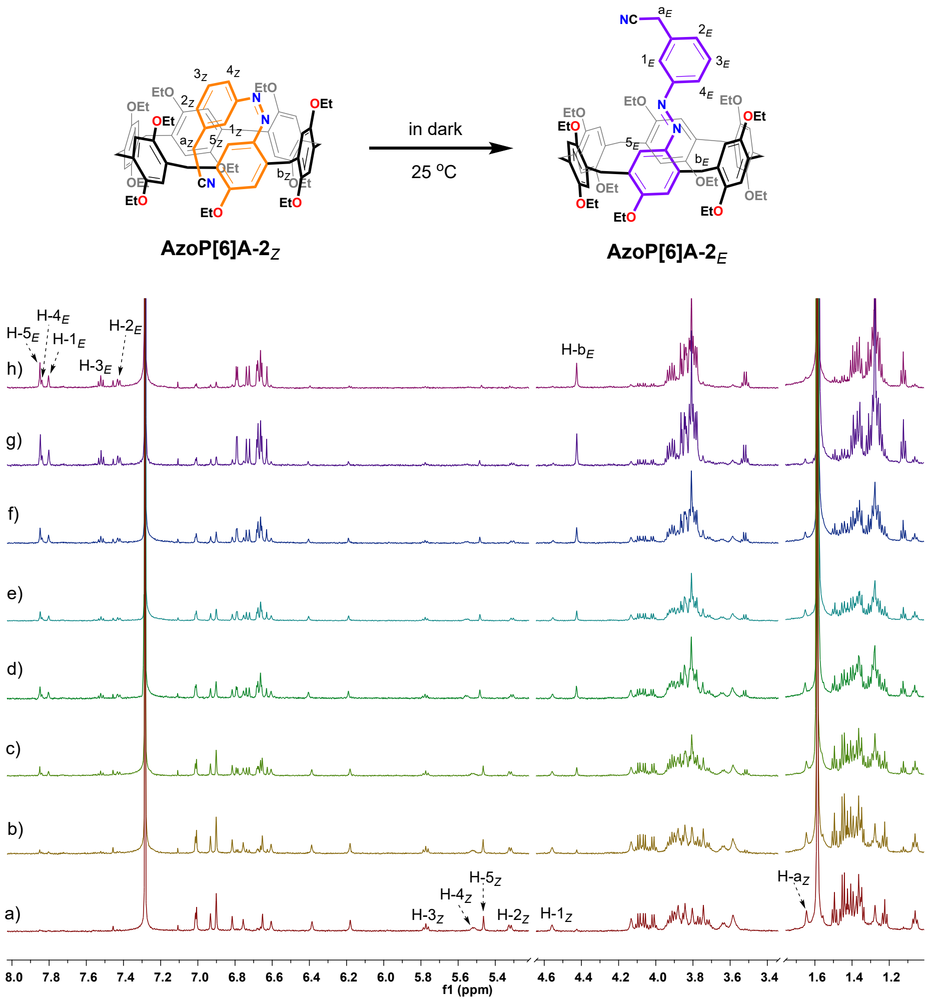

Fig. S22 Schematic representation of the *Z*-to-*E* thermal relaxation behavior of **AzoP[6]A-2**, and partial ^{1}H NMR spectra (600 MHz, 2.0 mM, $CDCl_3$, 298 K) for the solution of the PSS_Z (390 nm) mixtures of **AzoP[6]A-2** under conditions of a) as prepared, and after the rest of b) 5 hours and 57 min, c) 29 hours and 13 min, d) 49 hours and 26 min, e) 52 hours and 25 min, f) 80 hours and 22 min, g) 140 hours and 7 min, and h) 167 hours and 3 min at 25 °C in dark.

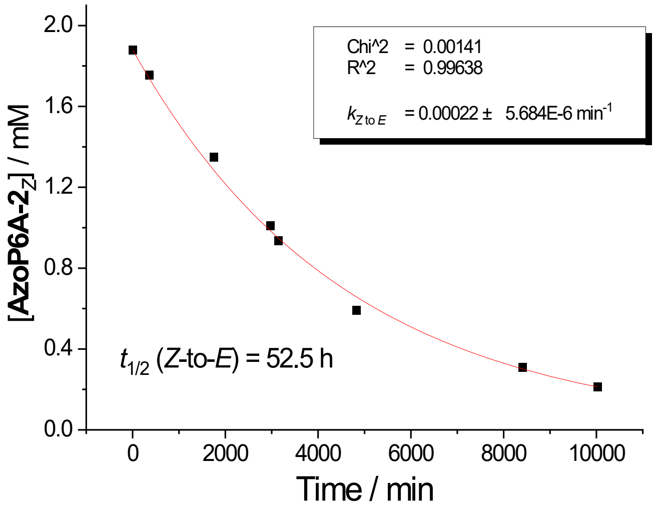

Fig. S23 Time dependent concentration changing plots and the fitting curve of **AzoP[6]A-2**$_Z$ at 25 °C in dark based on the data of Fig. S22.

Section 4: Guest Binding Properties of the *E*-isomeric AzoP[5/6]A macrocycles

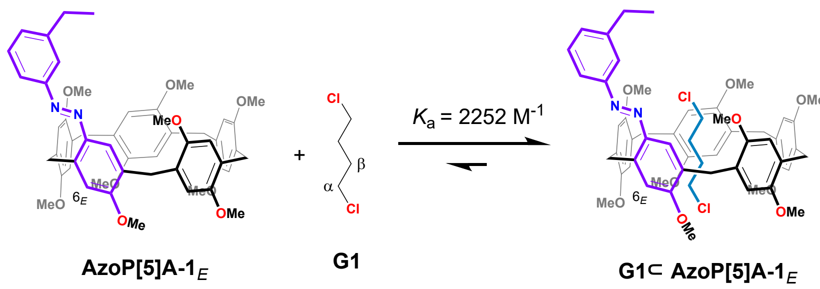

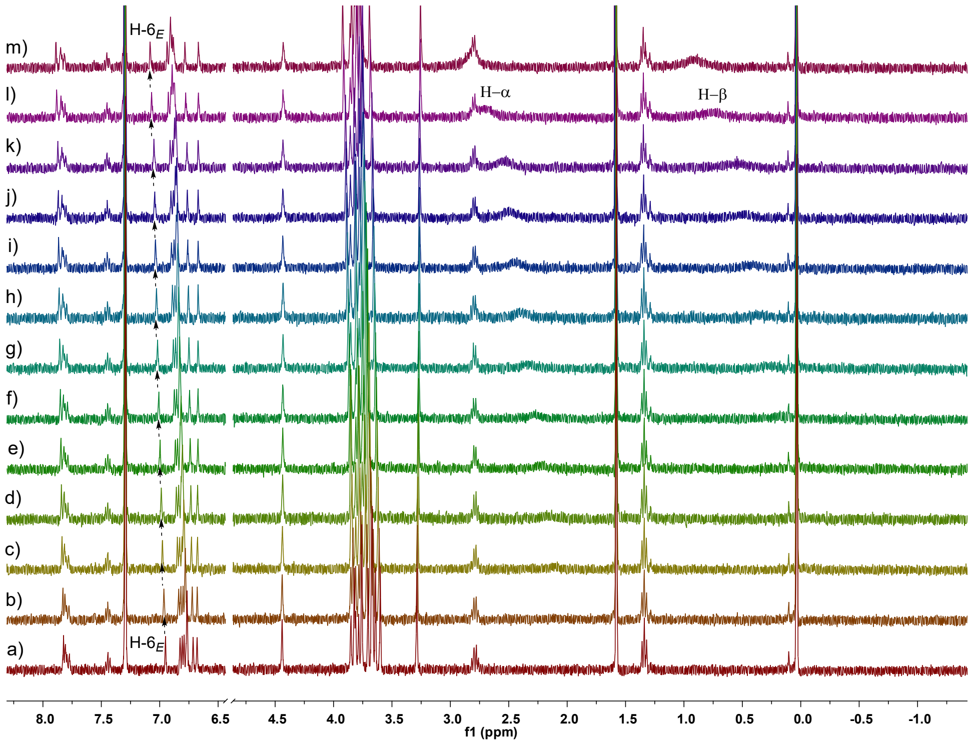

Fig. S24 Schematic representation for the complexation behavior of **AzoP[5]A-1**$_E$ and **G1**, and partial ^{1}H NMR spectra (400 MHz, $CDCl_3$, 298 K) for the solution of **AzoP[5]A-1**$_E$ (2.0 mM) in the presence of a) 0, b) 0.1, c) 0.2, d) 0.3, e) 0.4, f) 0.5, g) 0.6, h) 0.7, i) 0.8, j) 0.9, k) 1.0, l) 1.5, and m) 2.0 equiv. of **G1**.

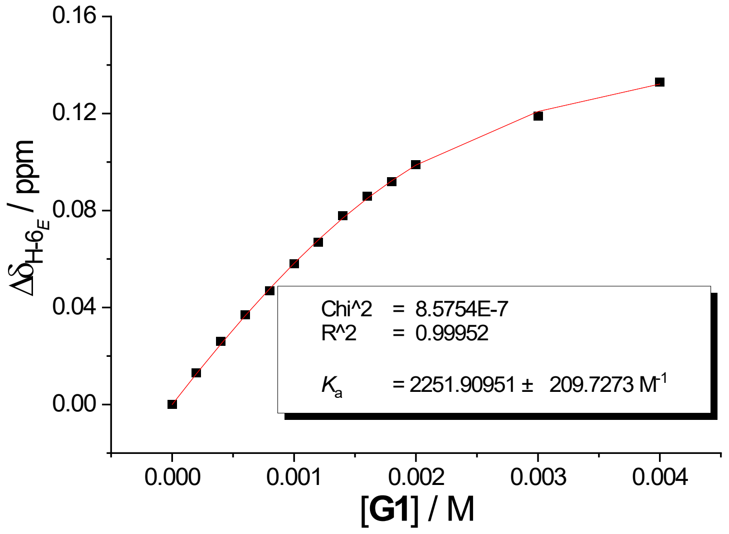

Fig. S25 Plot of the chemical shift changes of proton H-6$_E$ of **AzoP[5]A-1**$_E$ versus the concentration of **G1**. The fit of the data for H-6$_E$ (δ ppm) from Fig. S24 to a 1:1 binding model.

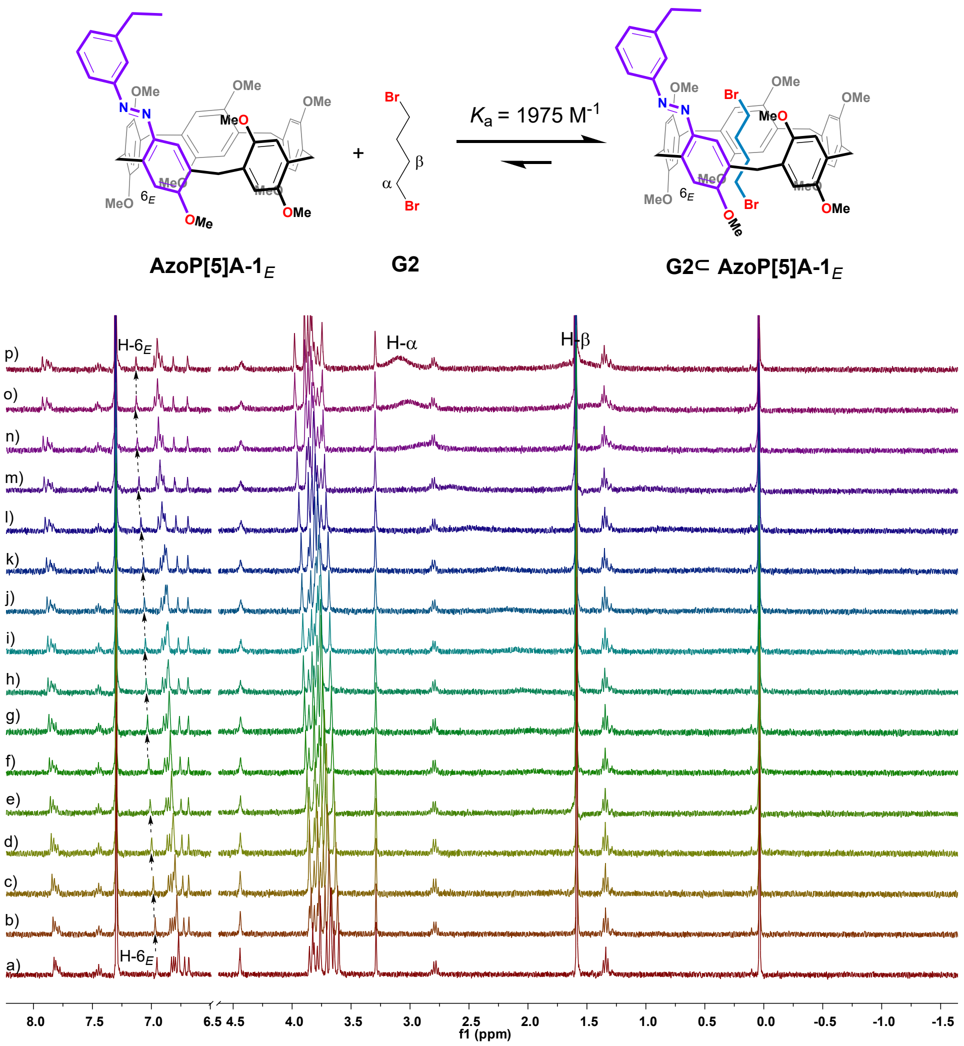

Fig. S26 Schematic representation for the complexation of **AzoP[5]A-1**$_E$ and **G2**, and partial ^{1}H NMR spectra (400 MHz, $CDCl_3$, 298 K) for the solution of **AzoP[5]A-1**$_E$ (2.0 mM) in the presence of a) 0, b) 0.1, c) 0.2, d) 0.3, e) 0.4, f) 0.5, g) 0.6, h) 0.7, i) 0.8, j) 0.9, k) 1.0, l) 1.5, m) 2.0, n) 3.0, o) 4.0, and p) 5.0 equiv. of **G2**.

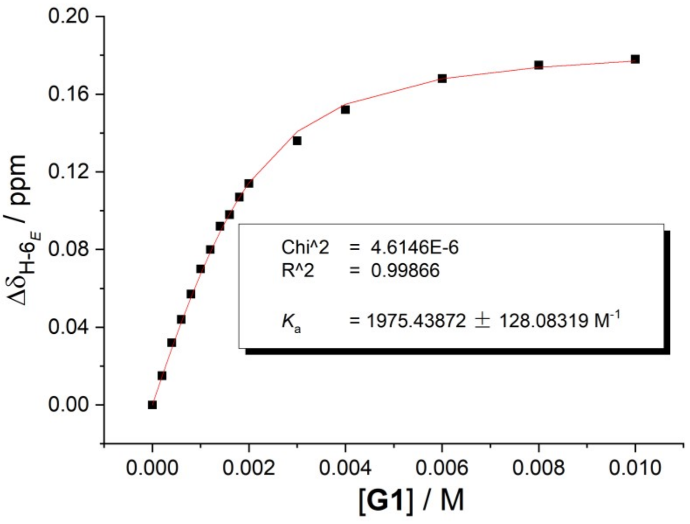

Fig. S27 Plot of the chemical shift changes of proton H-6$_E$ of **AzoP[5]A-1**$_E$ versus the concentration of **G2**. The fit of the data for H-6$_E$ (δ ppm) from Fig. S26 to a 1:1 binding model.

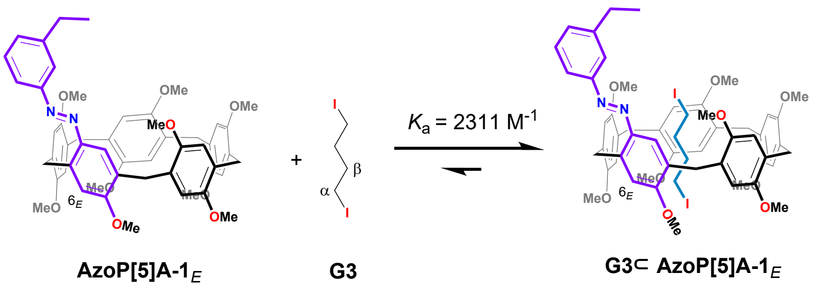

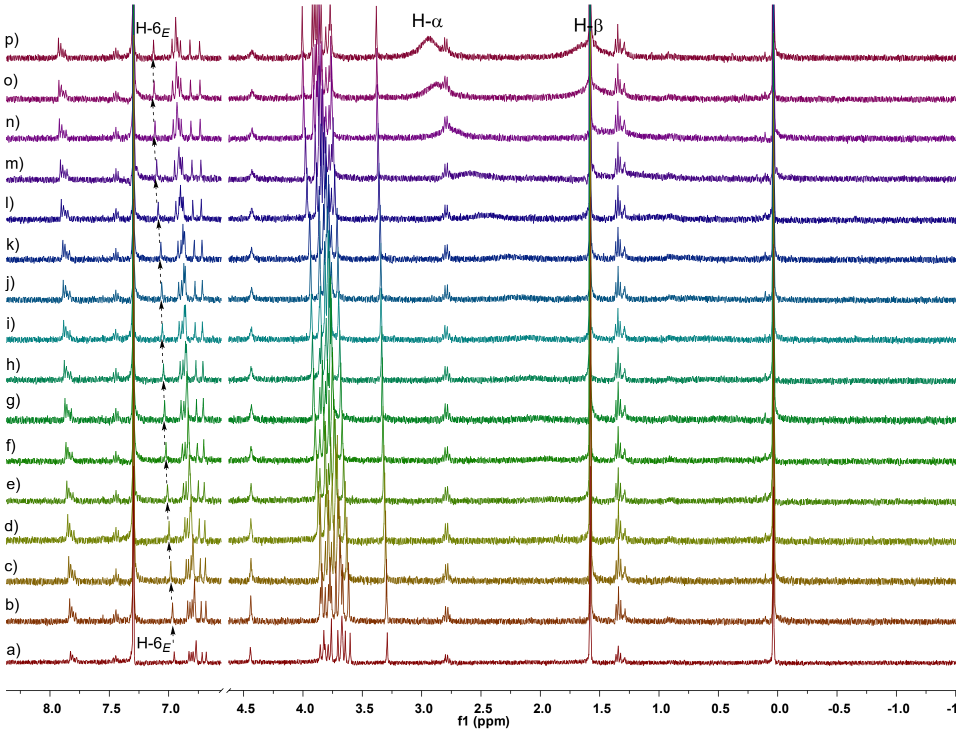

Fig. S28 Schematic representation for the complexation of **AzoP[5]A-1**$_E$ and **G3**, and partial ^{1}H NMR spectra (400 MHz, $CDCl_3$, 298 K) for the solution of **AzoP[5]A-1**$_E$ (2.0 mM) in the presence of a) 0, b) 0.1, c) 0.2, d) 0.3, e) 0.4, f) 0.5, g) 0.6, h) 0.7, i) 0.8, j) 0.9, k) 1.0, l) 1.5, m) 2.0, n) 3.0, o) 4.0, and p) 5.0 equiv. of **G3**.

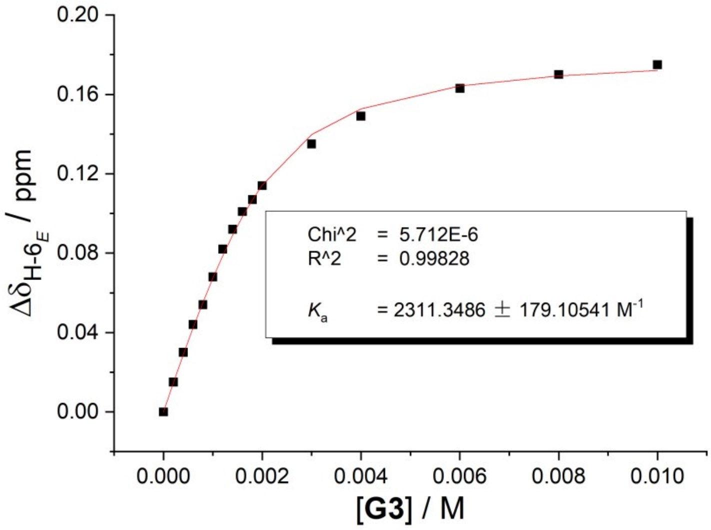

Fig. S29 Plot of the chemical shift changes of proton H-6$_E$ of **AzoP[5]A-1**$_E$ versus the concentration of **G3**. The fit of the data for H-6$_E$ (δ ppm) from Fig. S28 to a 1:1 binding model.

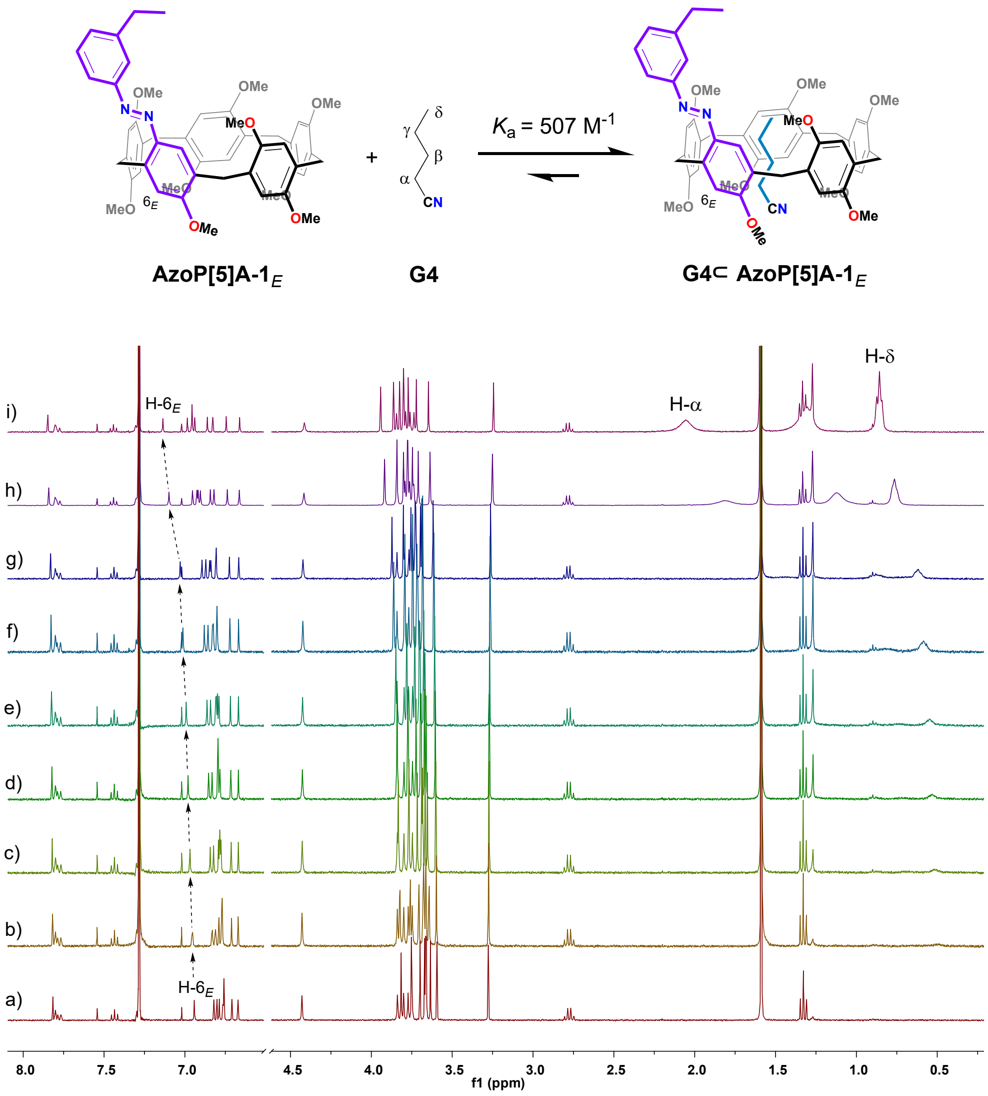

Fig. S30 Schematic representation for the complexation behavior of **AzoP[5]A-1**$_E$ and **G4**, and partial ^{1}H NMR spectra (400 MHz, $CDCl_3$, 298 K) for the solution of **AzoP[5]A-1**$_E$ (2.0 mM) in the presence of a) 0, b) 0.125, c) 0.25, d) 0.375, e) 0.5, f) 0.75, g) 1.0, h) 3.0, and i) 7.0 equiv. of **G4**.

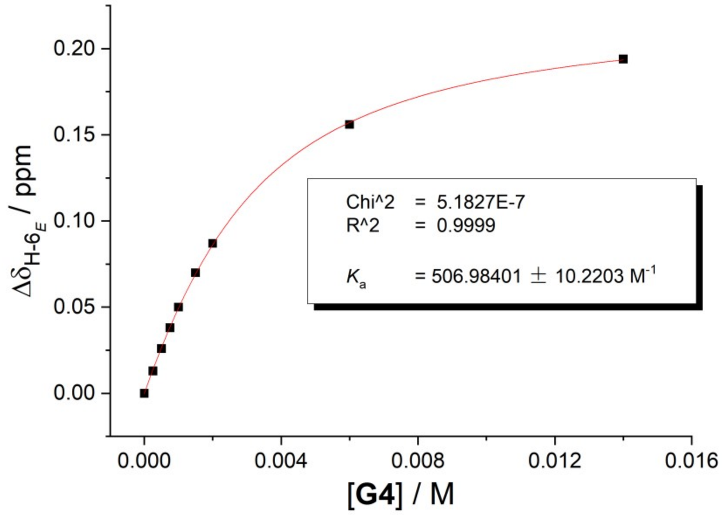

Fig. S31 Plot of the chemical shift changes of proton H-6$_E$ of **AzoP5A-1**$_E$ versus the concentration of **G4**. The fit of the data for H-6$_E$ (δ ppm) from Fig. S30 to a 1:1 binding model.

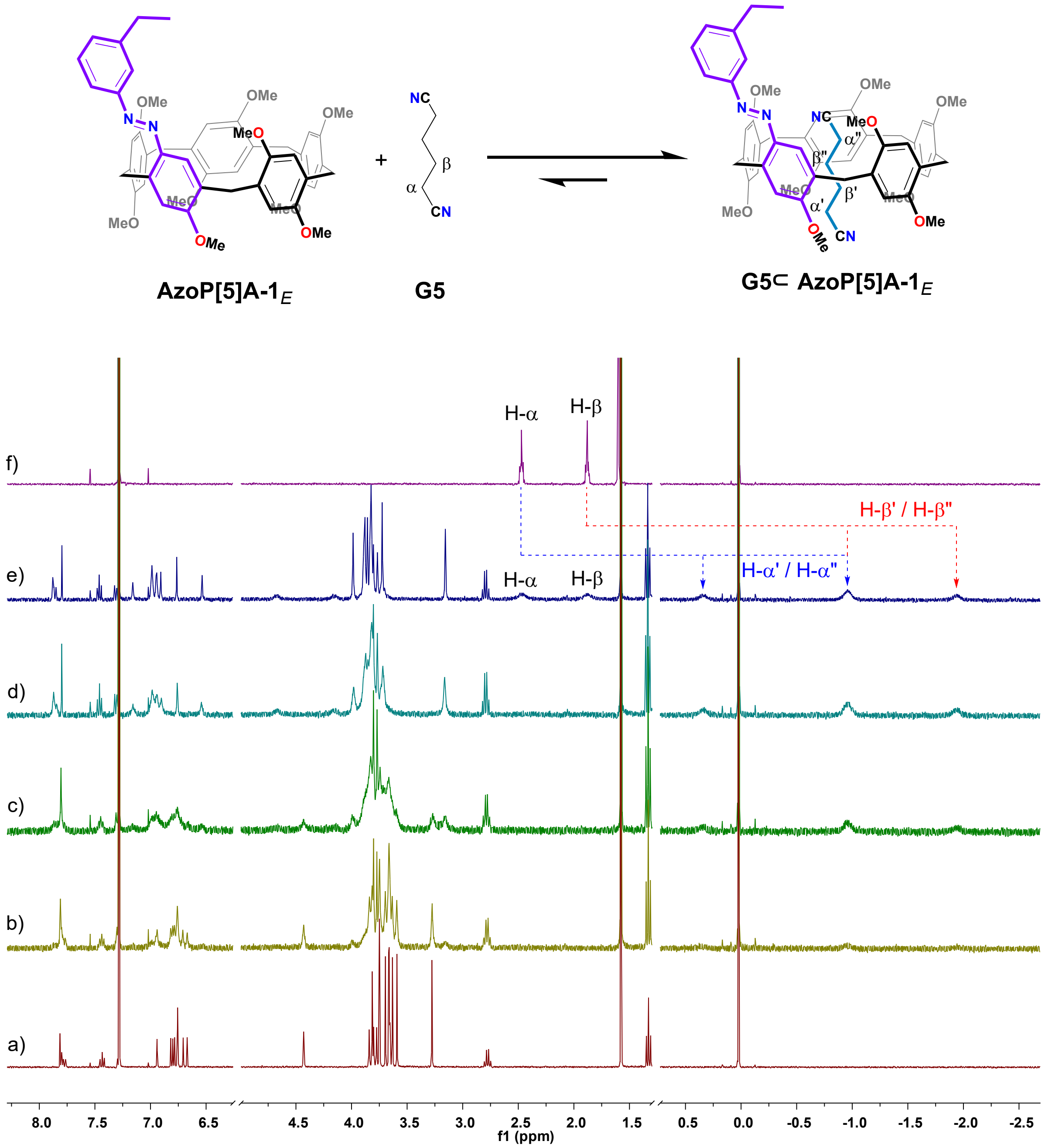

Fig. S32 Schematic representation for the complexation behavior of **AzoP[5]A-1**$_E$ and **G5**, as well as the partial ^{1}H NMR spectra (400 MHz, $CDCl_3$, 298 K) for the solution of f) **G5** (3.0 mM), and **AzoP[5]A-1**$_E$ (2.0 mM) in the presence of a) 0, b) 0.25, c) 0.50, d) 1.0, and e) 1.5 equiv. of **G5**.

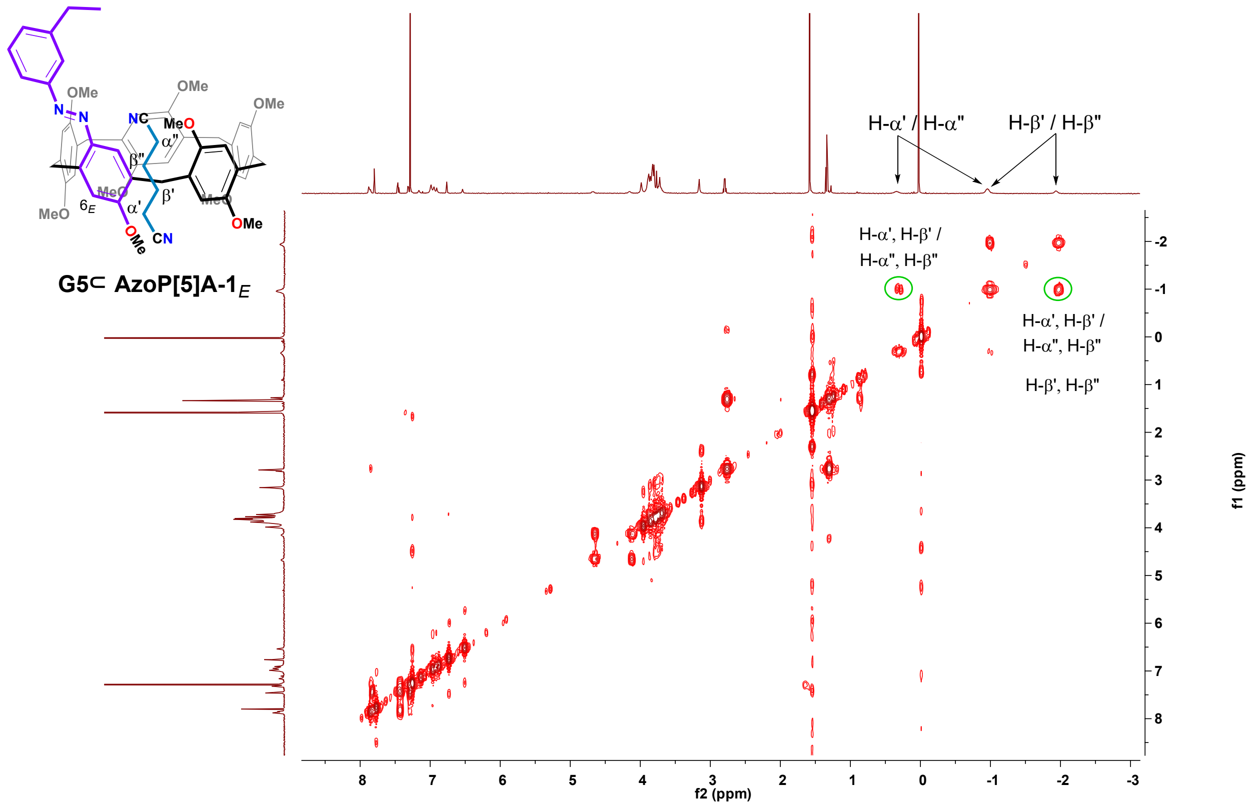

Fig. S33 2D COSY-NMR spectrum (600 MHz, $CDCl_3$, 298 K) of the solution of **AzoP[5]A-1**$_E$ (4.0 mM) and **G5** (4.0 mM).

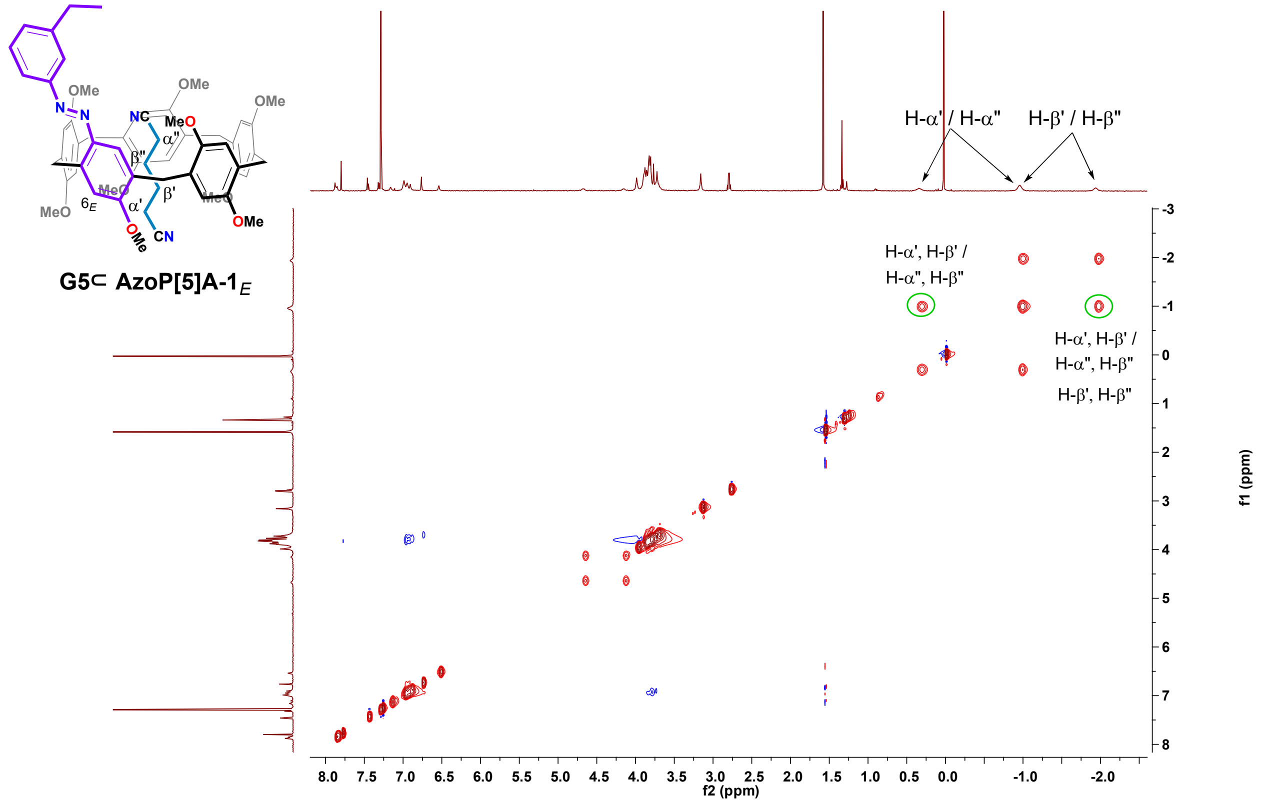

Fig. S34 2D NOESY-NMR spectrum (600 MHz, $CDCl_3$, 298 K) of the solution of **AzoP[5]A-1**$_E$ (4.0 mM) and **G5** (4.0 mM).

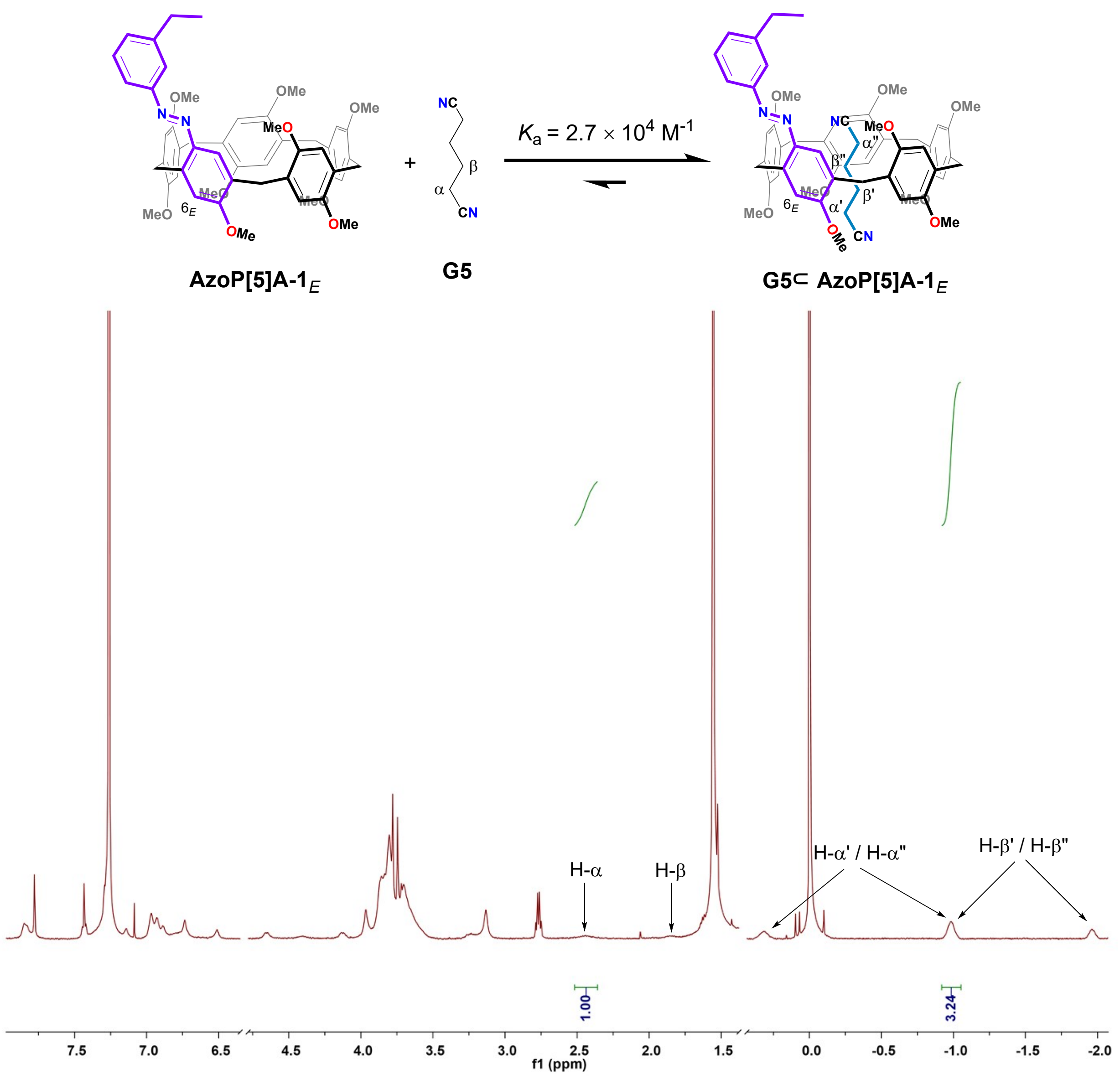

Fig. S35 Schematic representation for the complexation behavior of **AzoP[5]A-1**$_E$ and **G5**, as well as the partial ^{1}H NMR spectra (600 MHz, $CDCl_3$, 298 K) for the solution of **AzoP[5]A-1**$_E$ (0.5 mM) and **G5** (0.5 mM).

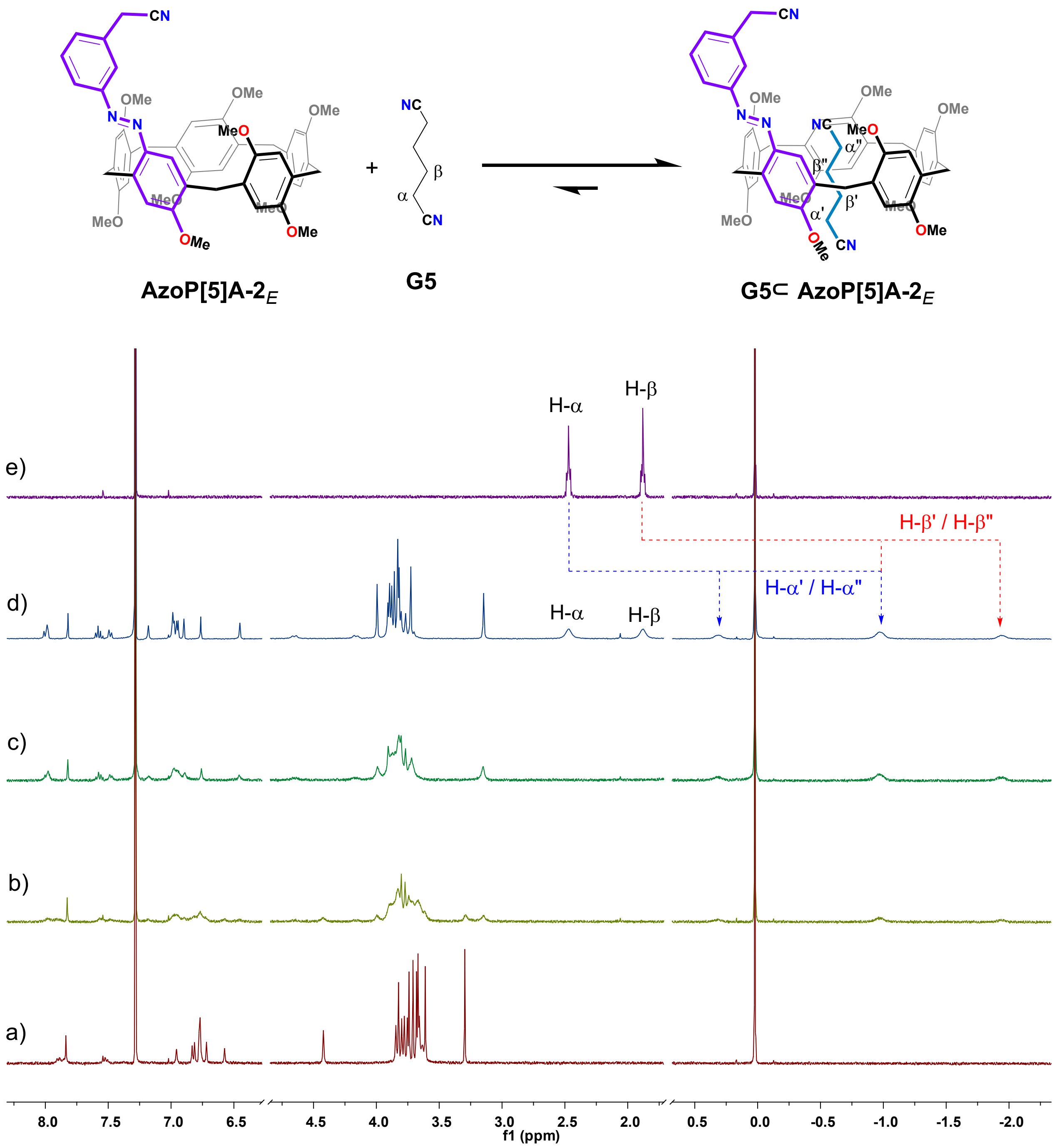

Fig. S36 Schematic representation for the complexation behavior of **AzoP[5]A-2**$_E$ and **G5**, as well as the partial ^{1}H NMR spectra (400 MHz, $CDCl_3$, 298 K) for the solution of e) **G5** (2.0 mM), and **AzoP[5]A-1**$_E$ (2.0 mM) in the presence of a) 0, b) 0.50, c) 1.0, and d) 1.5 equiv. of **G5**.

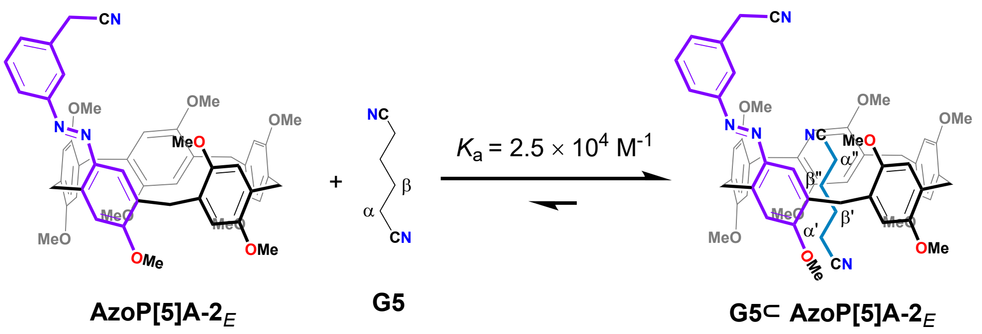

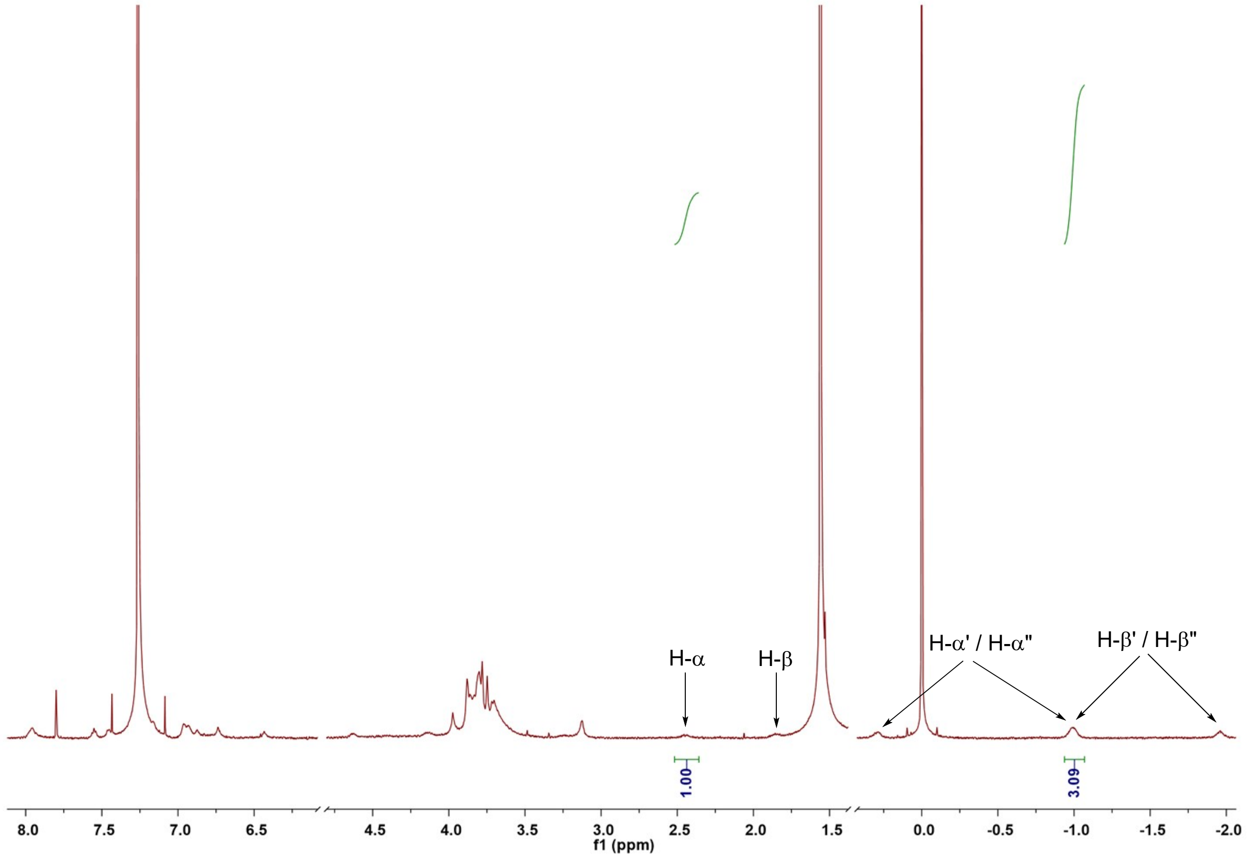

Fig. S37 Schematic representation for the complexation behavior of **AzoP[5]A-2**$_E$ and **G5**, as well as the partial ^{1}H NMR spectra (600 MHz, $CDCl_3$, 298 K) for the solution of **AzoP[5]A-2**$_E$ (0.5 mM) and **G5** (0.5 mM).

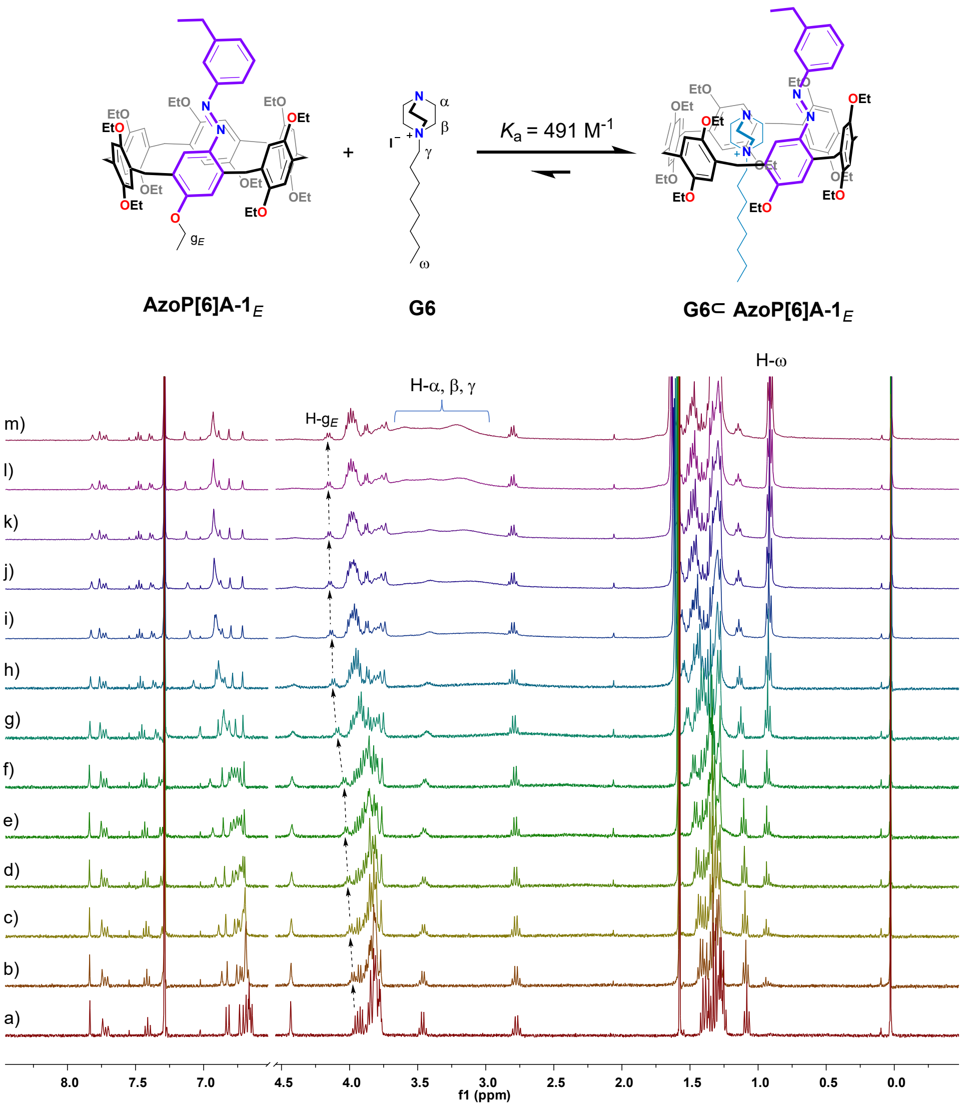

Fig. S38 Schematic representation of the complexation behavior of **AzoP[6]A-1**$_E$ and **G6**, as well as the partial ^{1}H NMR spectra (400 MHz, $CDCl_3$, 298 K) for the solution of **AzoP[6]A-1**$_E$ (2.0 mM) in the presence of a) 0, b) 0.2, c) 0.4, d) 0.6, e) 0.8, f) 1.0, g) 2.0, h) 3.0, i) 4.0, j) 5.0, k) 6.0, l) 7.0, and m) 8.0 equiv. of **G6**.

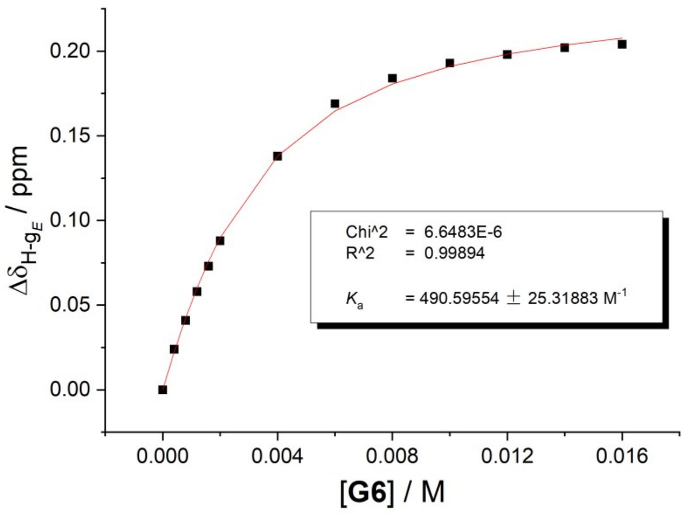

Fig. S39 Plot of the chemical shift changes of proton H-g$_E$ of **AzoP[6]A-1**$_E$ versus the concentration of **G6**. The fit of the data for H-g$_E$ (δ ppm) from Fig. S38 to a 1:1 binding model.

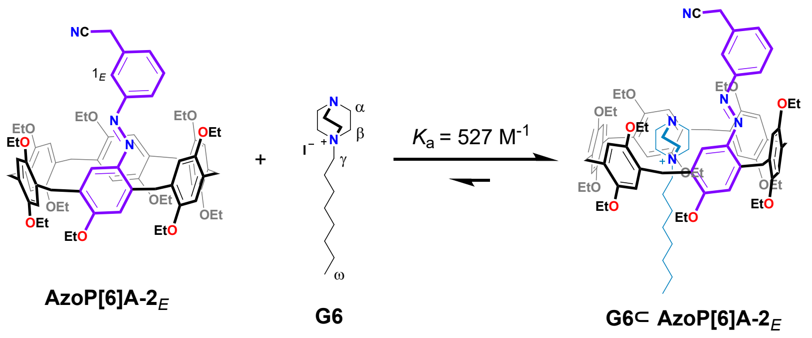

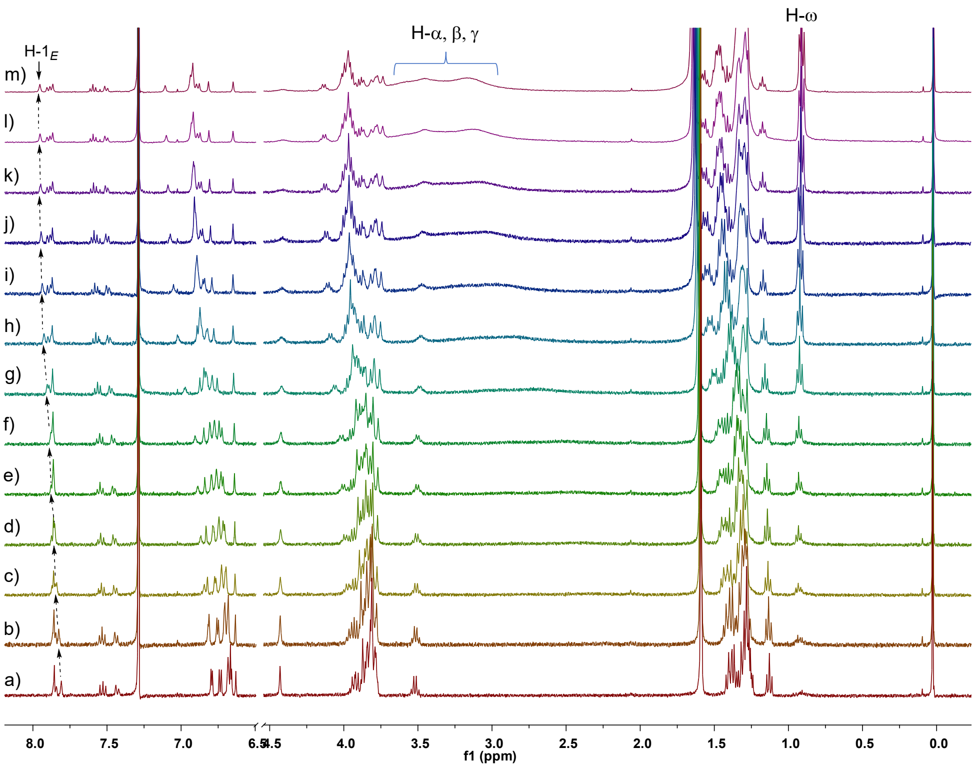

Fig. S40 Schematic representation of the complexation behavior of **AzoP[6]A-2$_E$** and **G6**, as well as the partial ^{1}H NMR spectra (400 MHz, $CDCl_3$, 298 K) for the solution of **AzoP[6]A-2$_E$** (2.0 mM) in the presence of a) 0, b) 0.2, c) 0.4, d) 0.6, e) 0.8, f) 1.0, g) 2.0, h) 3.0, i) 4.0, j) 5.0, k) 6.0, l) 7.0, and m) 8.0 equiv. of **G6**.

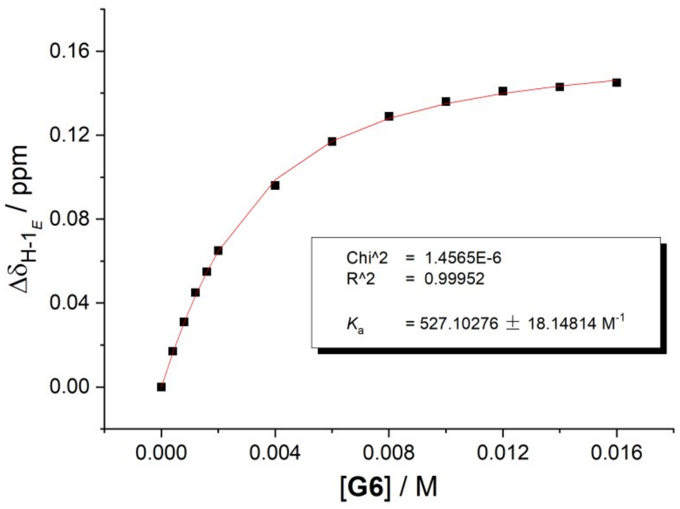

Fig. S41 Plot of the chemical shift changes of proton H-1$_E$ of **AzoP[6]A-1$_E$** versus the concentration of **G6**. The fit of the data for H-1$_E$ (δ ppm) from Fig. S40 to a 1:1 binding model.

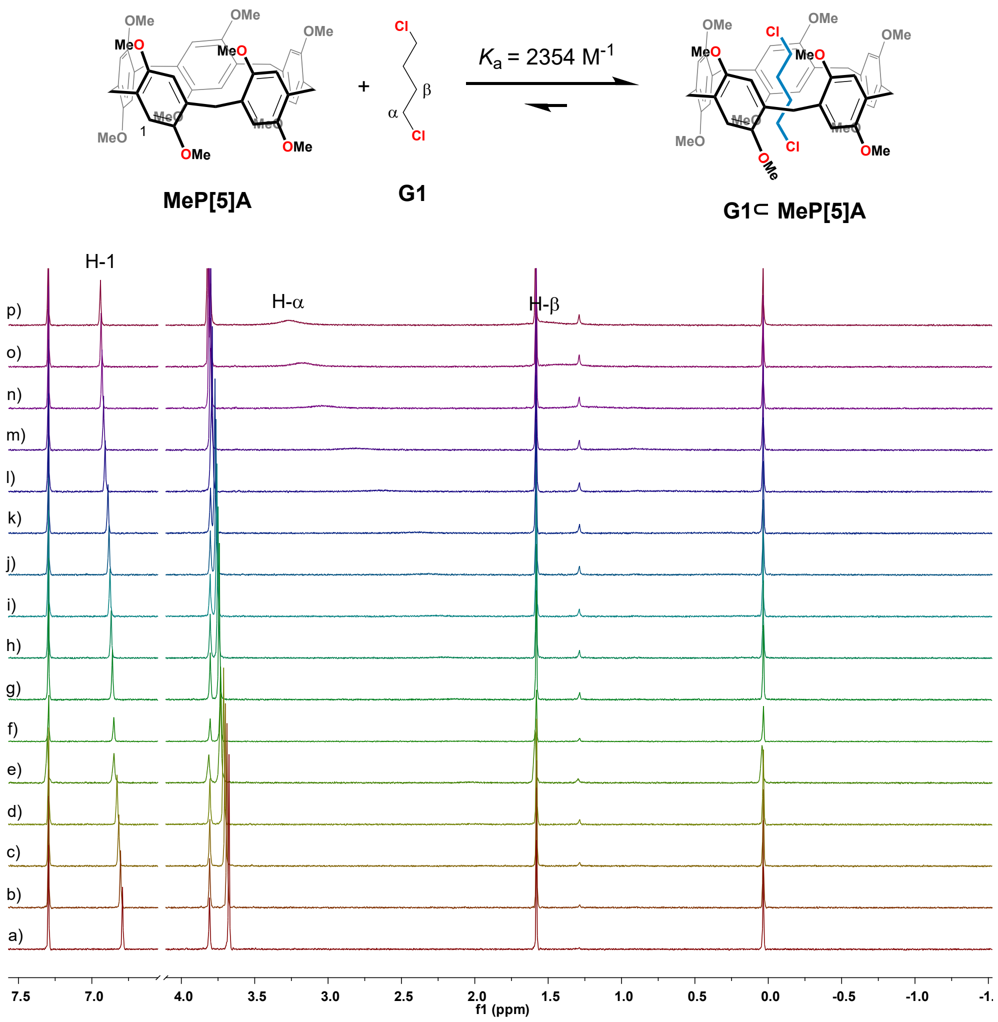

Fig. S42 Schematic representation for the complexation of **MeP[5]A** and **G1**, as well as the partial ^{1}H NMR spectra (400 MHz, $CDCl_3$, 298 K) for the solution of **MeP[5]A** (2.0 mM) in the presence of a) 0, b) 0.1, c) 0.2, d) 0.3, e) 0.4, f) 0.5, g) 0.6, h) 0.7, i) 0.8, j) 0.9, k) 1.0, l) 1.5, m) 2.0, n) 3.0, o) 4.0, and p) 5.0 equiv. of **G1**.

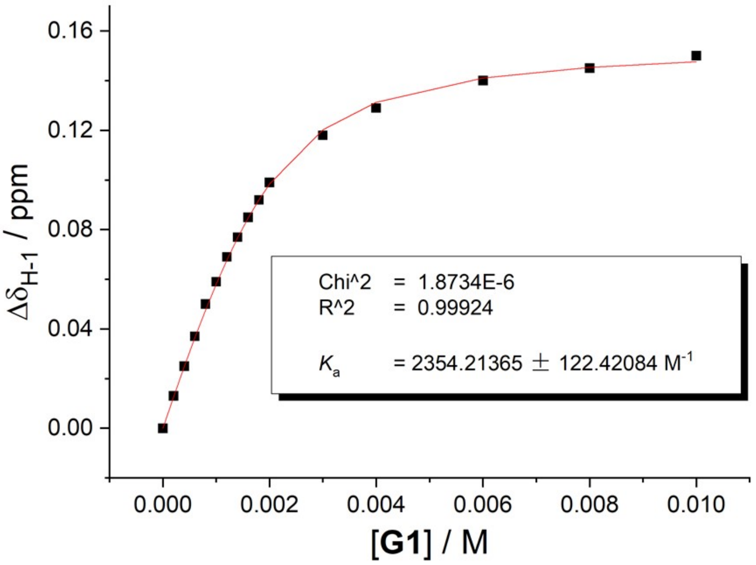

Fig. S43 Plot of the chemical shift changes of proton H-1 of **MeP[5]A** versus the concentration of **G1**. The fit of the data for H-1 (δ ppm) from Fig. S42 to a 1:1 binding model.

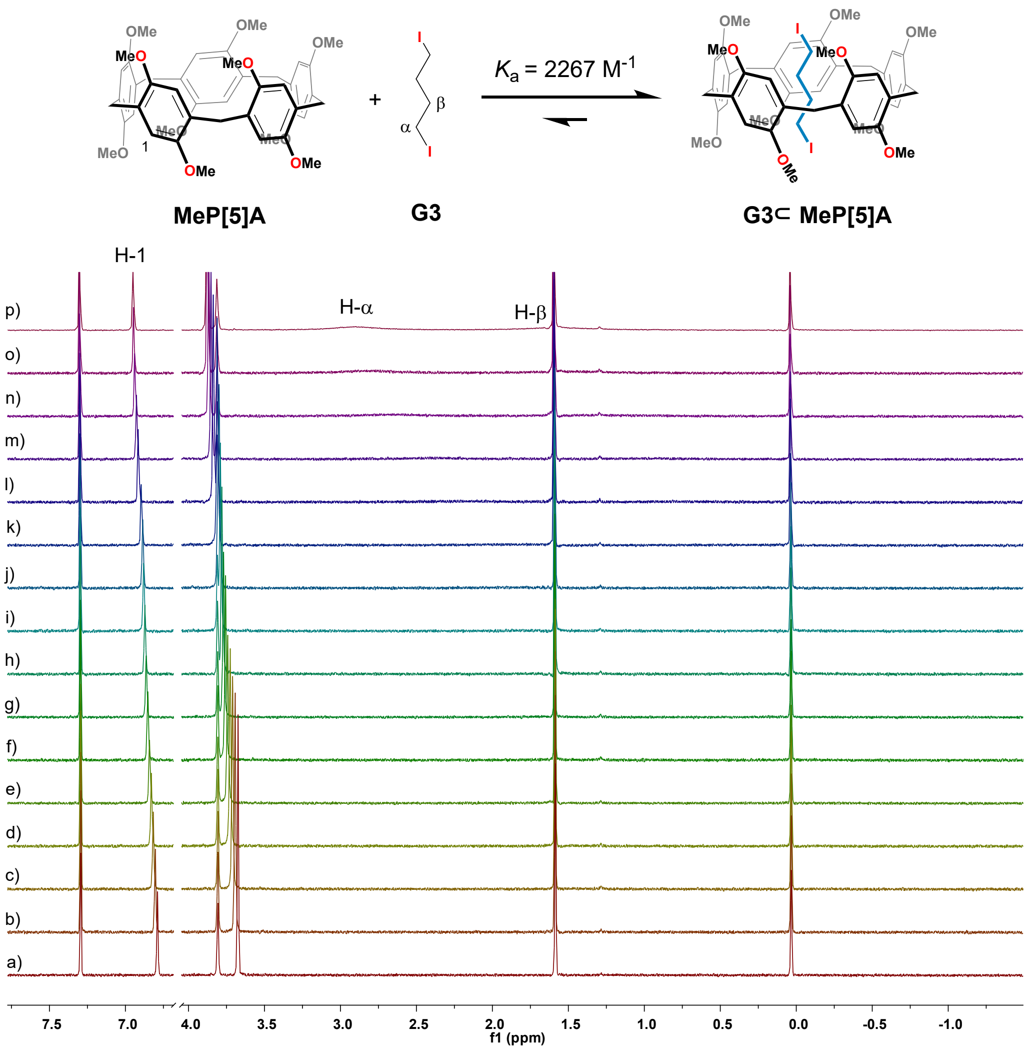

Fig. S44. Schematic representation for the complexation of **MeP[5]A** and **G3**, as well as the partial ^{1}H NMR spectra (400 MHz, $CDCl_3$, 298 K) for the solution of **MeP[5]A** (2.0 mM) in the presence of a) 0, b) 0.1, c) 0.2, d) 0.3, e) 0.4, f) 0.5, g) 0.6, h) 0.7, i) 0.8, j) 0.9, k) 1.0, l) 1.5, m) 2.0, n) 3.0, o) 4.0, and p) 5.0 equiv. of **G3**.

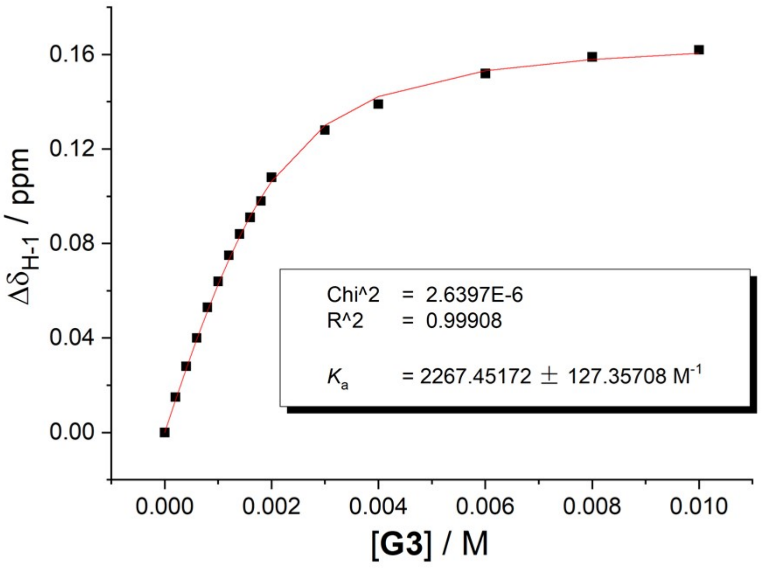

Fig. S45 Plot of the chemical shift changes of proton H-1 of **MeP[5]A** versus the concentration of **G3**. The fit of the data for H-1 (δ ppm) from Fig. S44 to a 1:1 binding model.

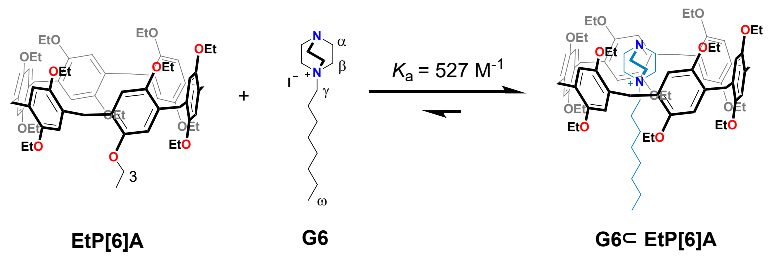

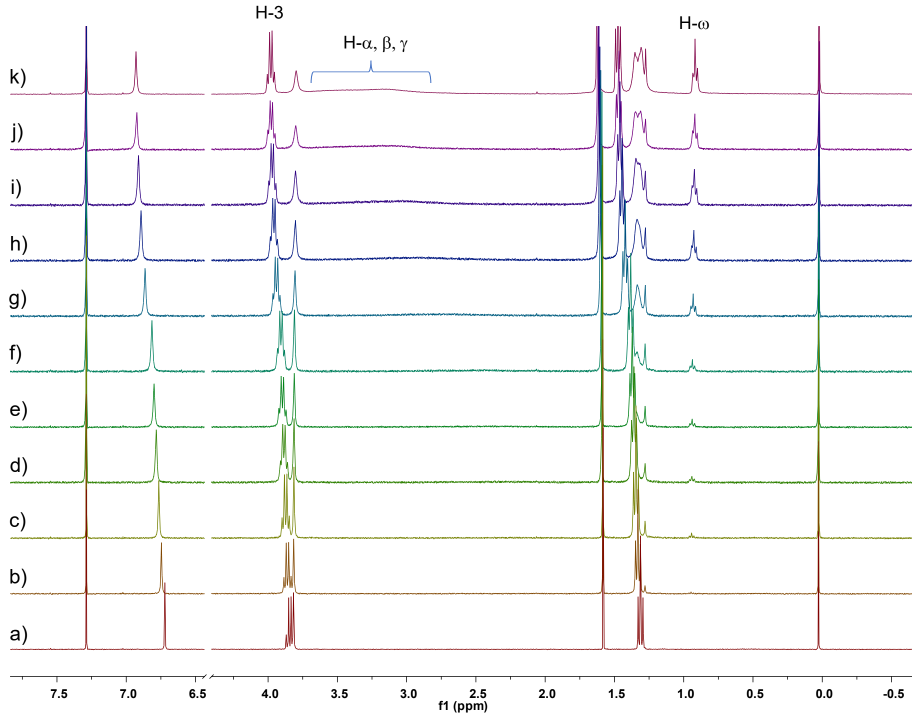

Fig. S46 Schematic representation of the complexation behavior of **EtP[6]A** and **G6**, as well as the partial ^{1}H NMR spectra (400 MHz, $CDCl_3$, 298 K) for the solution of **EtP[6]A** (2.0 mM) in the presence of a) 0, b) 0.2, c) 0.4, d) 0.6, e) 0.8, f) 1.0, g) 2.0, h) 3.0, i) 4.0, j) 5.0 and k) 6.0 equiv. of **G6**.

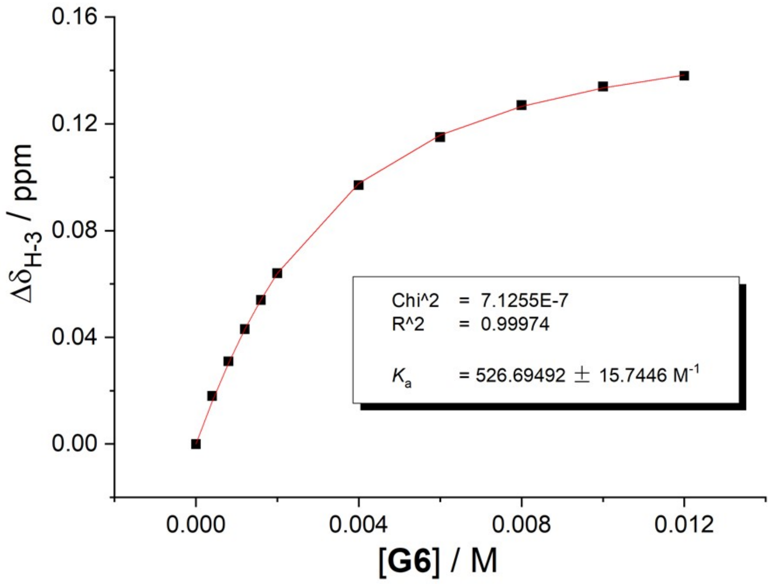

Fig. S47 Plot of the chemical shift changes of proton H-3 of **EtP[6]A** versus the concentration of **G6**. The fit of the data for H-3 (δ ppm) from Fig. S46 to a 1:1 binding model.

Table S2. Association constants for the 1:1 host-guest complexations between guests **G1** ~ **G6** with the *E*-isomeric **AzoP[5]A** and **AzoP[6]A**, as well as **MeP[5]A** and **EtP[6]A**, respectively, in the solution at 298 K.

Guests	Hosts	K_a / M^{-1}	Solvent
**G1**	**MeP[5]A**	2354 ± 122 [a]	$CDCl_3$
	**AzoP[5]A-1$_E$**	2252 ± 210 [a]	$CDCl_3$
**G2**	**MeP[5]A**	(1.6 ± 0.1) × 10^3 [b]	$CDCl_3$
	**AzoP[5]A-1$_E$**	1975 ± 128 [a]	$CDCl_3$
**G3**	**MeP[5]A**	2267 ± 127 [a]	$CDCl_3$
	**AzoP[5]A-1$_E$**	2311 ± 179 [a]	$CDCl_3$
**G4**	**MeP[5]A**	570 ± 40 [c]	$CDCl_3$
	**AzoP[5]A-1$_E$**	507 ± 10 [a]	$CDCl_3$
**G5**	**MeP[5]A**	(2.4 ± 0.4) × 10^4 [d]	$CDCl_3$ / DMSO (v/v, 9/1)
	**AzoP[5]A-1$_E$**	2.7 × 10^4 [e]	$CDCl_3$
	**AzoP[5]A-2$_E$**	2.5 × 10^4 [e]	$CDCl_3$
**G6**	**EtP[6]A**	527 ± 16 [a]	$CDCl_3$
	**AzoP[5]A-1$_E$**	491 ± 25 [a]	$CDCl_3$
	**AzoP[5]A-2$_E$**	527 ± 18 [a]	$CDCl_3$

[a] The K_a values were determined by ^{1}H NMR titration method.
[b] The K_a value was reported in Ref. S8.
[c] The K_a value was reported in Ref. S9.
[d] The K_a value was reported in Ref. S10.
[e] The K_a value was determined by ^{1}H NMR single-point method.

Section 5: Photoswitchable Host-Guest Complexation of AzoP[5/6]A and G1 ~ G6.

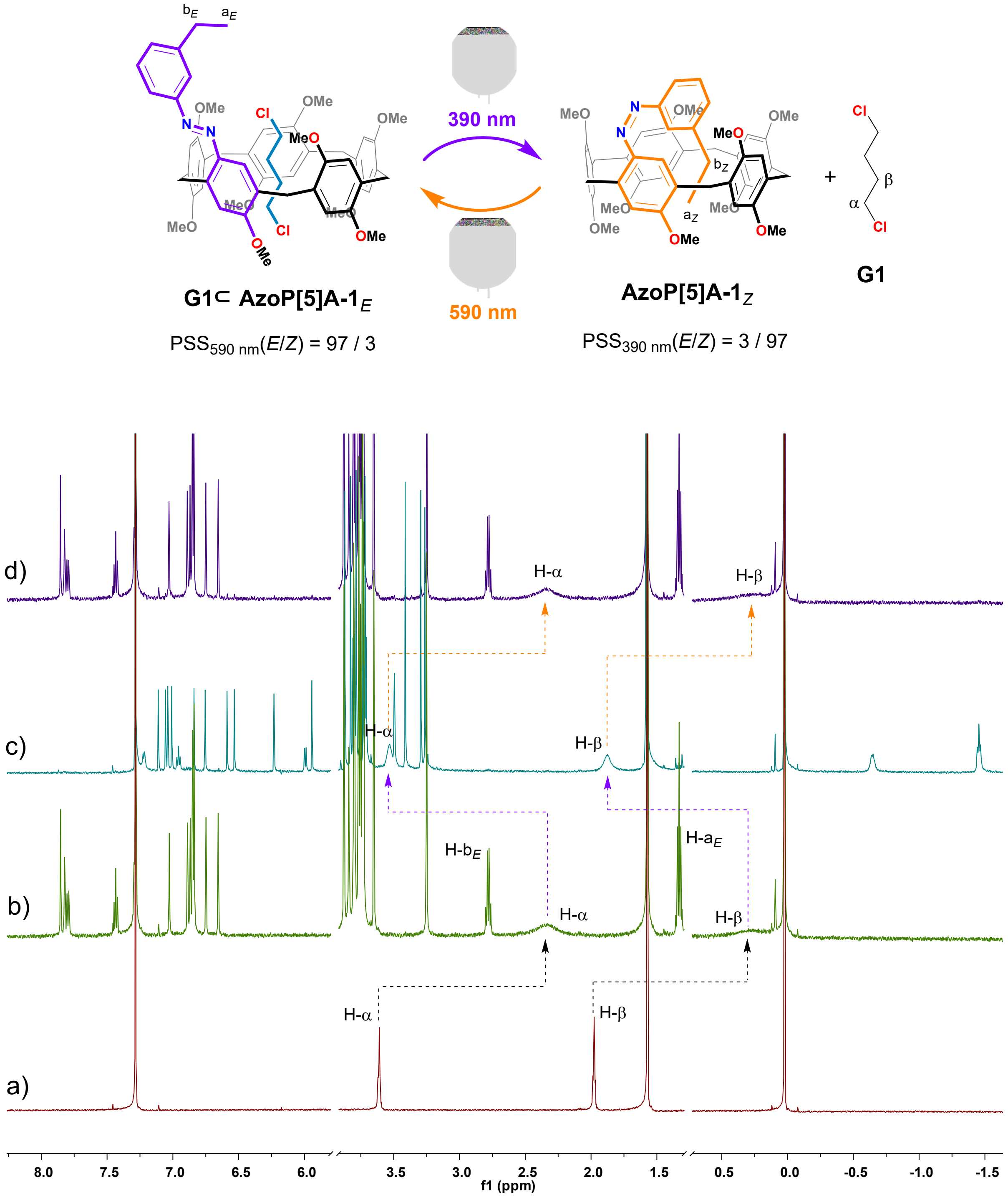

Fig. S48 Schematic representation for the *E*/*Z* photoisomerization of **AzoP[5]A-1** in the presence of **G1**, and partial ^{1}H NMR (600 MHz, $CDCl_3$, 298 K) spectra for the solution of a) **G1** (2.0 mM), b)**AzoP[5]A-1**$_E$ (2.0 mM) and **G1** (2.0 mM), c) the PSS$_Z$ (390 nm) mixtures of **AzoP[5]A-1** (2.0 mM) and **G1** (2.0 mM), and d) the PSS$_E$ (590 nm) mixtures of **AzoP[5]A-1** (2.0 mM) and **G1** (2.0 mM).

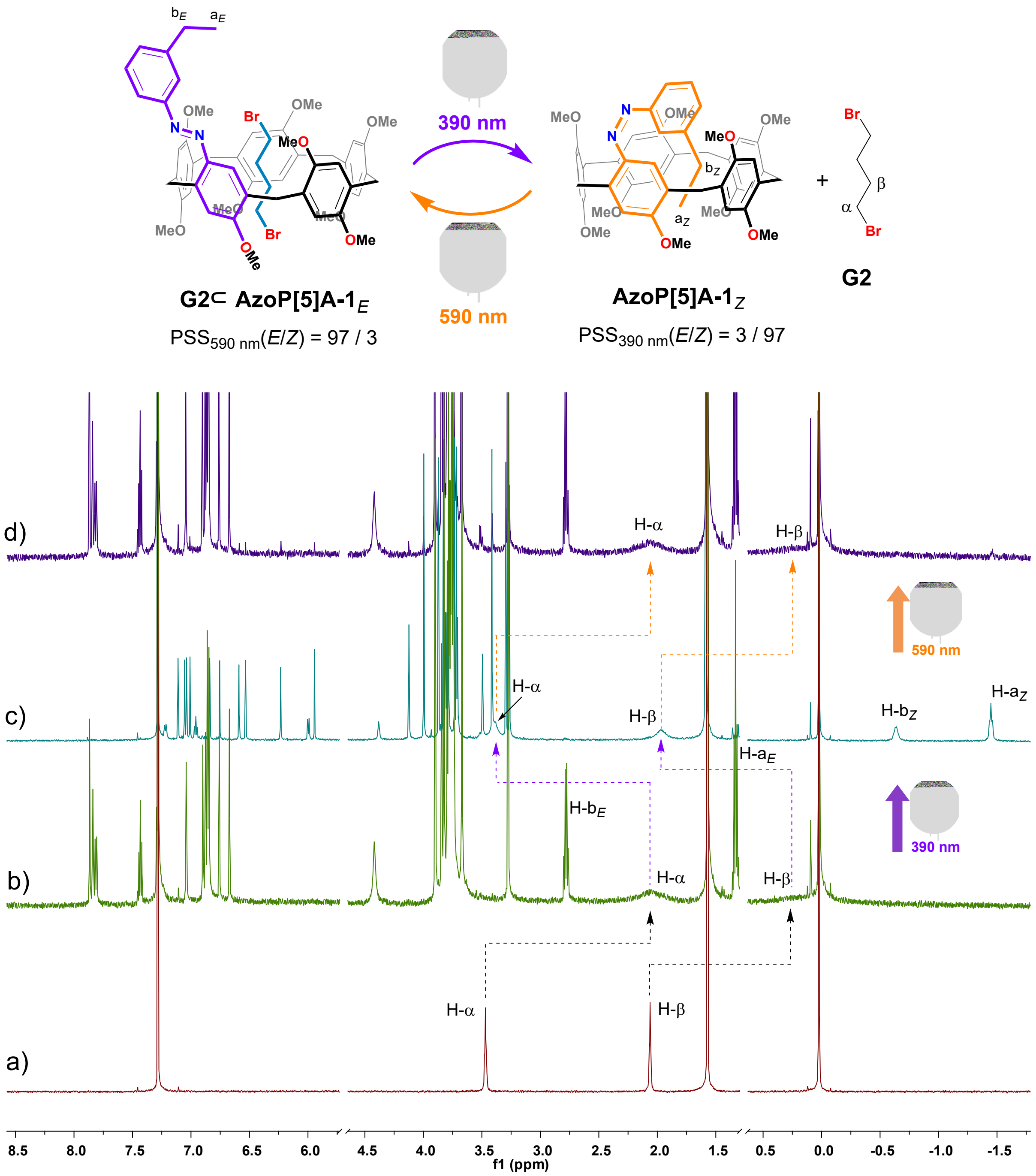

Fig. S49 Schematic representation for the *E/Z* photoisomerization of **AzoP[5]A-1** in the presence of **G2**, and partial ^{1}H NMR (600 MHz, $CDCl_3$, 298 K) spectra for the solution of a) **G2** (2.0 mM), b) **AzoP[5]A-1**$_E$ (2.0 mM) and **G2** (2.0 mM), c) the PSS$_Z$ (390 nm) mixtures of **AzoP[5]A-1** (2.0 mM) and **G2** (2.0 mM), and d) the PSS$_E$ (590 nm) mixtures of **AzoP[5]A-1** (2.0 mM) and **G2** (2.0 mM).

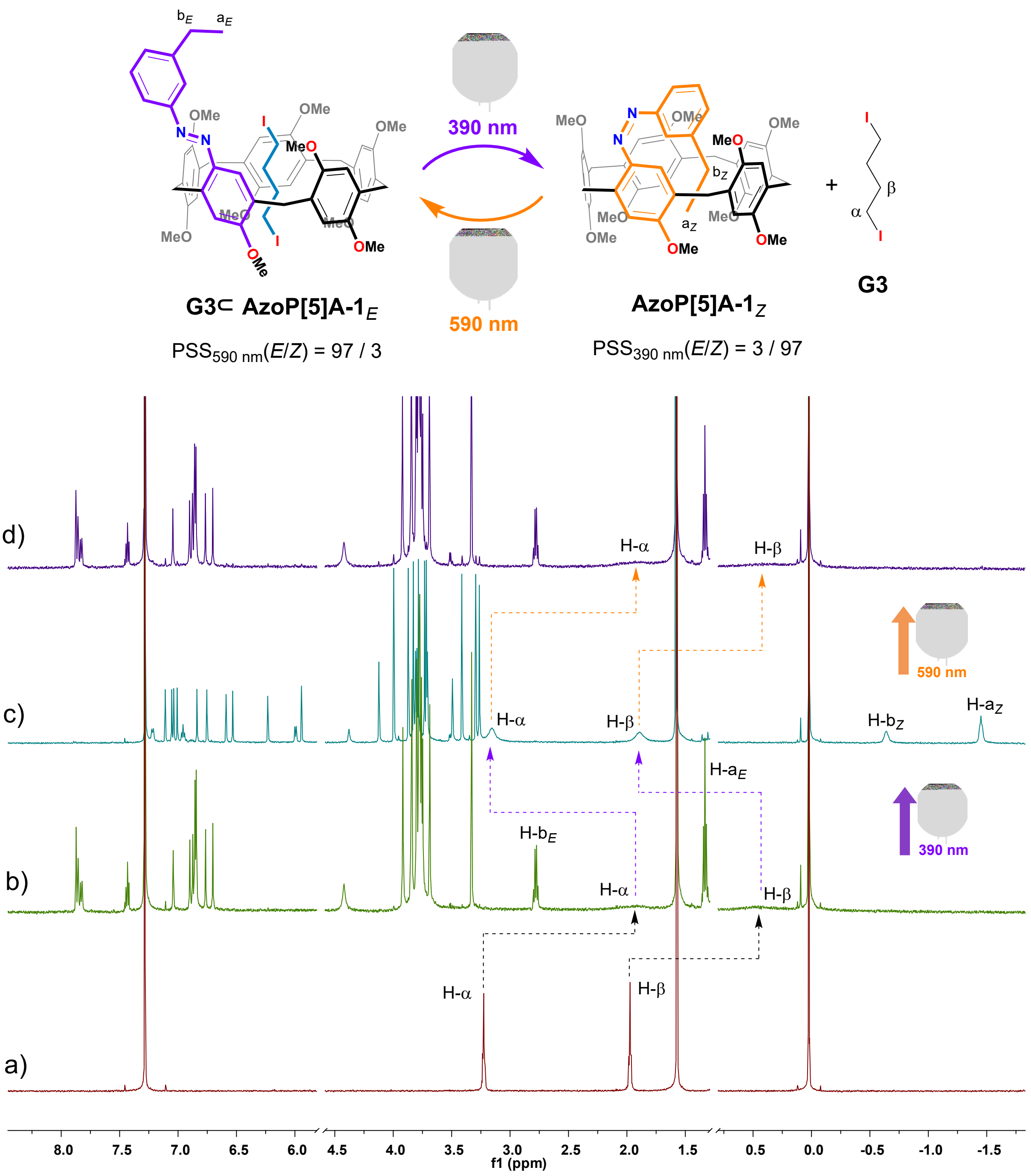

Fig. S50 Schematic representation for the *E/Z* photoisomerization of **AzoP[5]A-1** in the presence of **G3**, and partial ^{1}H NMR (600 MHz, $CDCl_3$, 298 K) spectra for the solution of a) **G3** (2.0 mM), b) **AzoP[5]A-1**$_E$ (2.0 mM) and **G3** (2.0 mM), c) the PSS$_Z$ (390 nm) mixtures of **AzoP[5]A-1** (2.0 mM) and **G3** (2.0 mM), and d) the PSS$_E$ (590 nm) mixtures of **AzoP[5]A-1** (2.0 mM) and **G3** (2.0 mM).

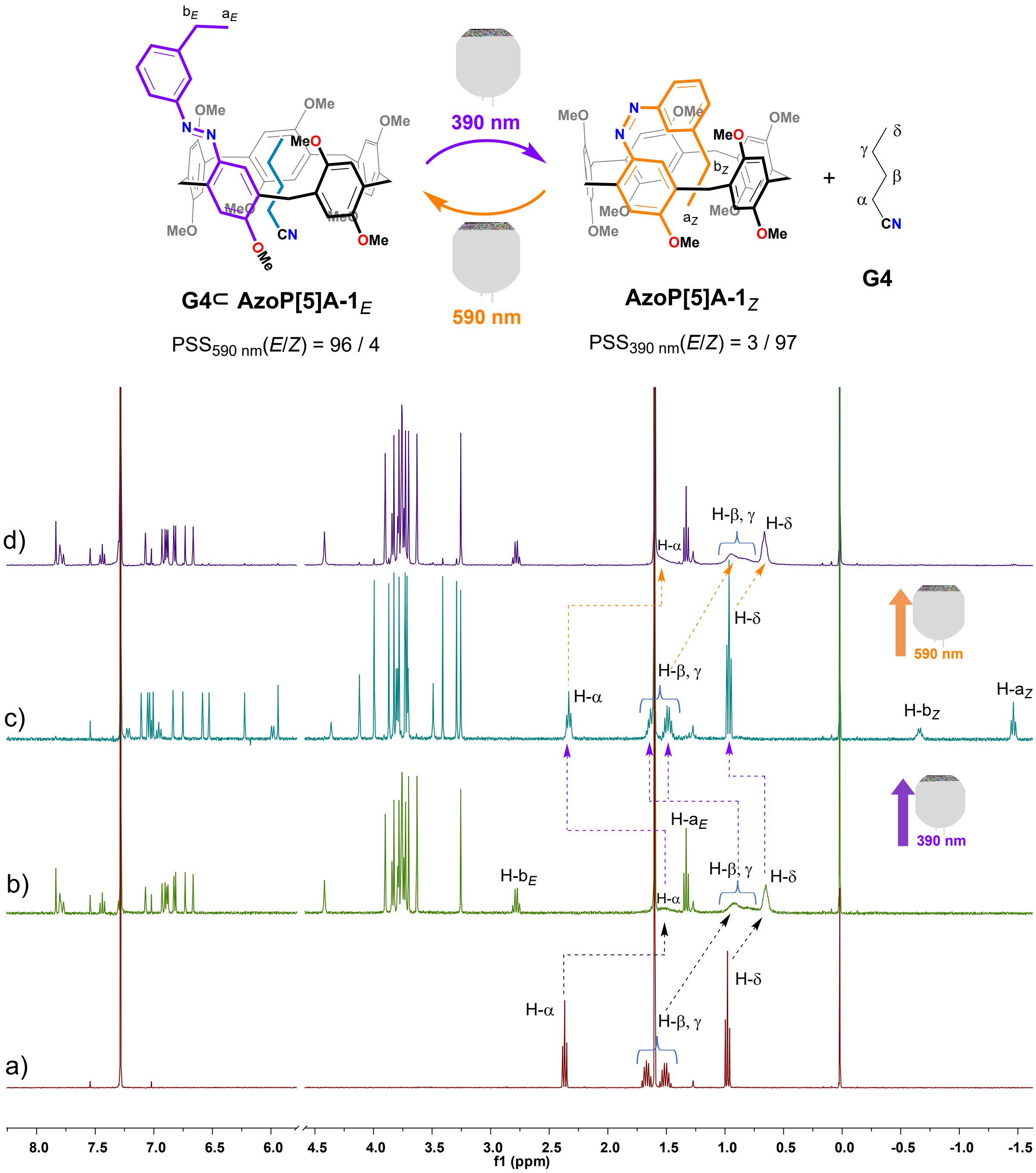

Fig. S51 Schematic representation for the *E*/*Z* photoisomerization of **AzoP[5]A-1** in the presence of **G4**, and partial ^{1}H NMR (400 MHz, $CDCl_3$, 298 K) spectra for the solution of a) **G4** (4.0 mM), b) **AzoP[5]A-1**$_E$ (2.0 mM) and **G4** (4.0 mM), c) the PSS$_Z$ (390 nm) mixtures of **AzoP[5]A-1** (2.0 mM) and **G4** (4.0 mM), and d) the PSS$_E$ (590 nm) mixtures of **AzoP[5]A-1** (2.0 mM) and **G4** (4.0 mM).

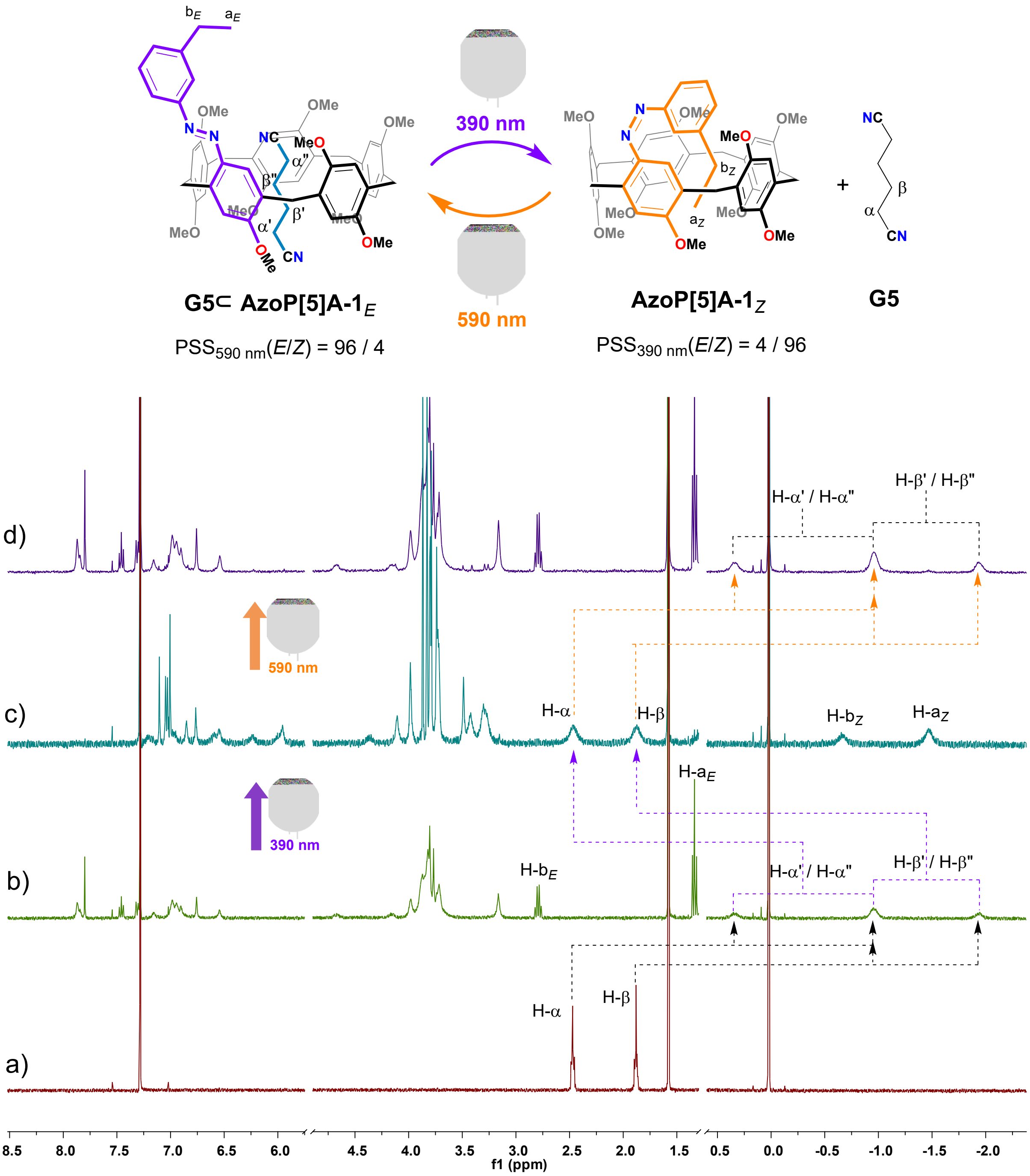

Fig. S52 Schematic representation for the *E/Z* photoisomerization of **AzoP[5]A-1** in the presence of **G5**, and partial ^{1}H NMR (400 MHz, $CDCl_3$, 298 K) spectra for the solution of a) **G5** (2.0 mM), b) **AzoP[5]A-1**$_E$ (2.0 mM) and **G5** (2.0 mM), c) the PSS$_Z$ (390 nm) mixtures of **AzoP[5]A-1** (2.0 mM) and **G5** (2.0 mM), and d) the PSS$_E$ (590 nm) mixtures of **AzoP[5]A-1** (2.0 mM) and **G5** (2.0 mM).

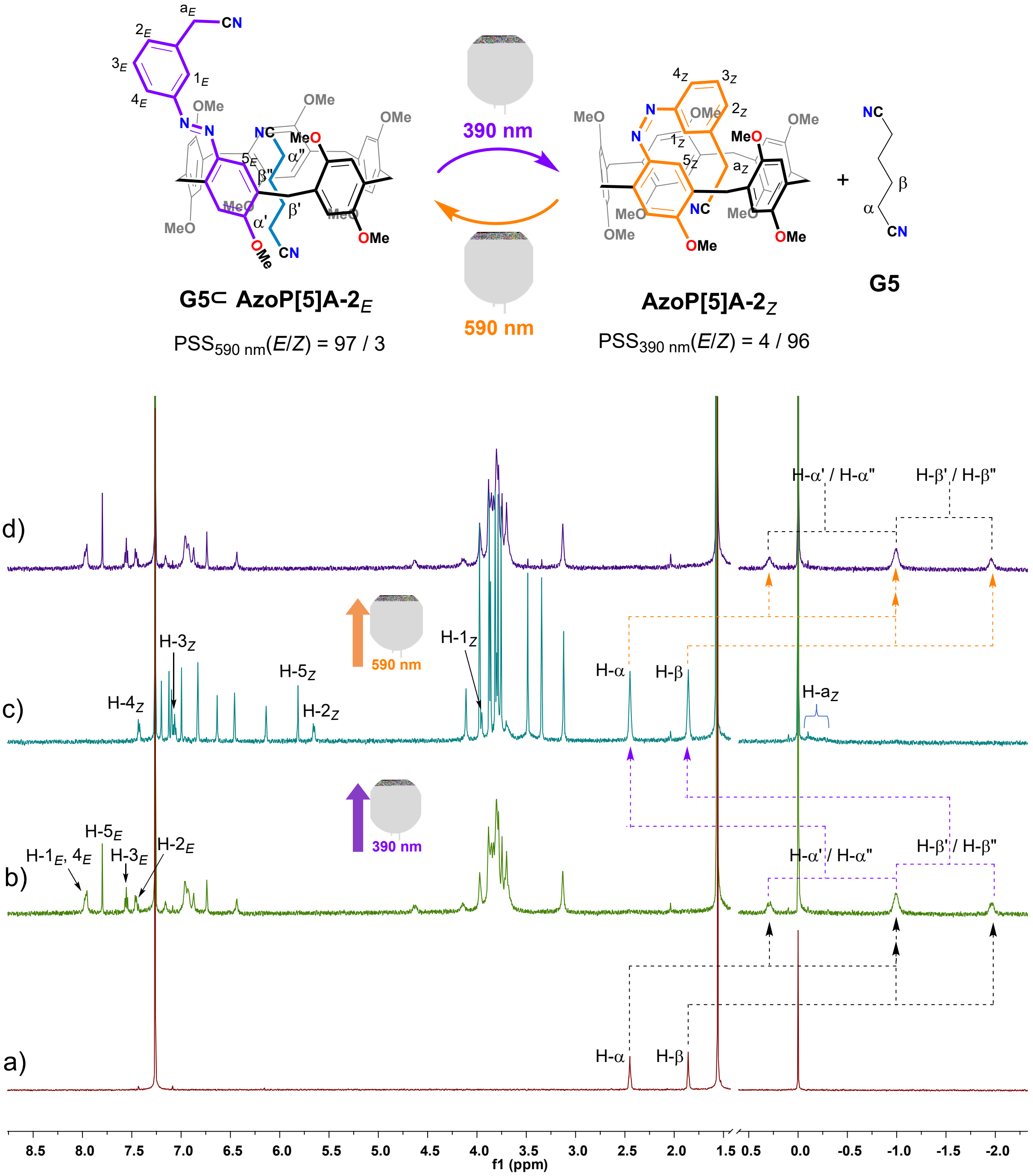

Fig. S53 Schematic representation for the *E*/*Z* photoisomerization of **AzoP[5]A-2** in the presence of **G5**, and partial ^{1}H NMR (400 MHz, $CDCl_3$, 298 K) spectra for the solution of a) **G5** (2.0 mM), b) **AzoP[5]A-2**$_E$ (2.0 mM) and **G5** (2.0 mM), c) the PSS_Z (390 nm) mixtures of **AzoP[5]A-2** (2.0 mM) and **G5** (2.0 mM), and d) the PSS_E (590 nm) mixtures of **AzoP[5]A-2** (2.0 mM) and **G5** (2.0 mM).

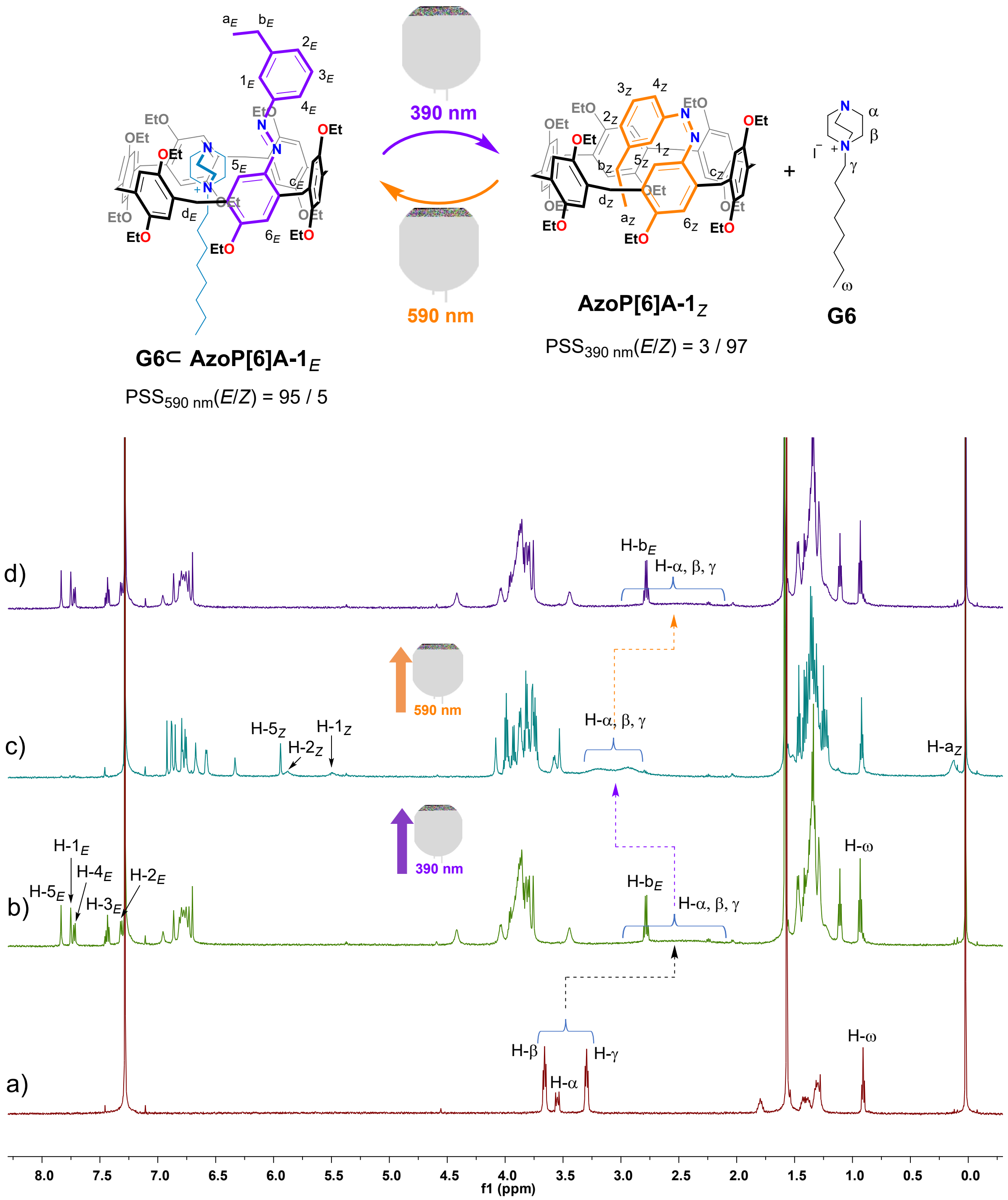

Fig. S54 Schematic representation for the *E/Z* photoisomerization of **AzoP[6]A-1** in the presence of **G6**, and partial ^{1}H NMR (600 MHz, $CDCl_3$, 298 K) spectra for the solution of a) **G6** (2.0 mM), b) **AzoP[6]A-1**$_E$ (2.0 mM) and **G6** (2.0 mM), c) the PSS$_Z$ (390 nm) mixtures of **AzoP[6]A-1** (2.0 mM) and **G6** (2.0 mM), and d) the PSS$_E$ (590 nm) mixtures of **AzoP[6]A-1** (2.0 mM) and **G6** (2.0 mM).

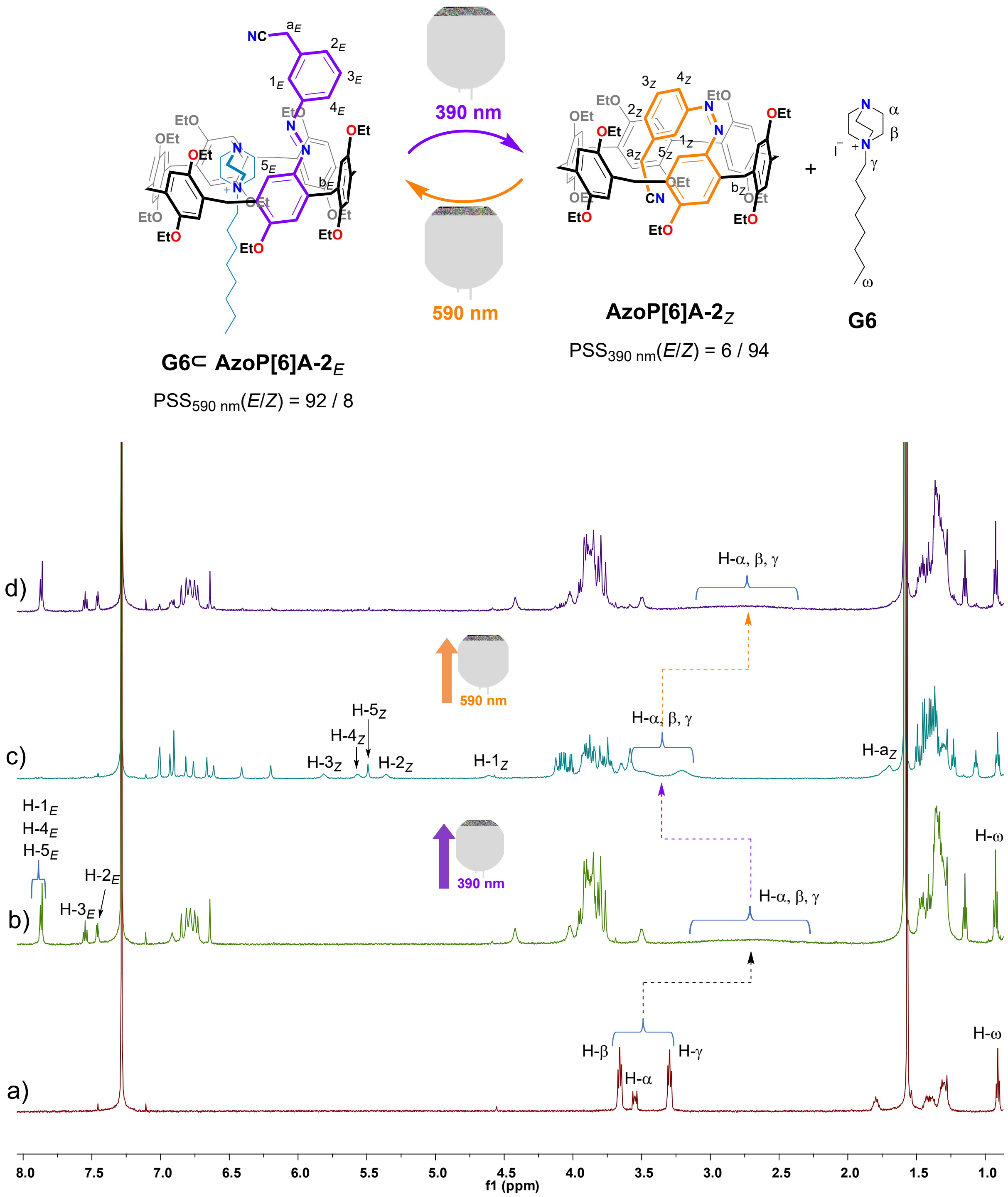

Fig. S55 Schematic representation for the *E/Z* photoisomerization of **AzoP[6]A-2** in the presence of **G6**, and partial ^{1}H NMR (600 MHz, $CDCl_3$, 298 K) spectra for the solution of a) **G6** (2.0 mM), b) **AzoP[6]A-2**$_E$ (2.0 mM) and **G6** (2.0 mM), c) the PSS$_Z$ (390 nm) mixtures of **AzoP[6]A-2** (2.0 mM) and **G6** (2.0 mM), and d) the PSS$_E$ (590 nm) mixtures of **AzoP[6]A-2** (2.0 mM) and **G6** (2.0 mM).

Section 6: Guest Binding Properties of the *Z*-isomeric AzoP[5/6]A macrocycles

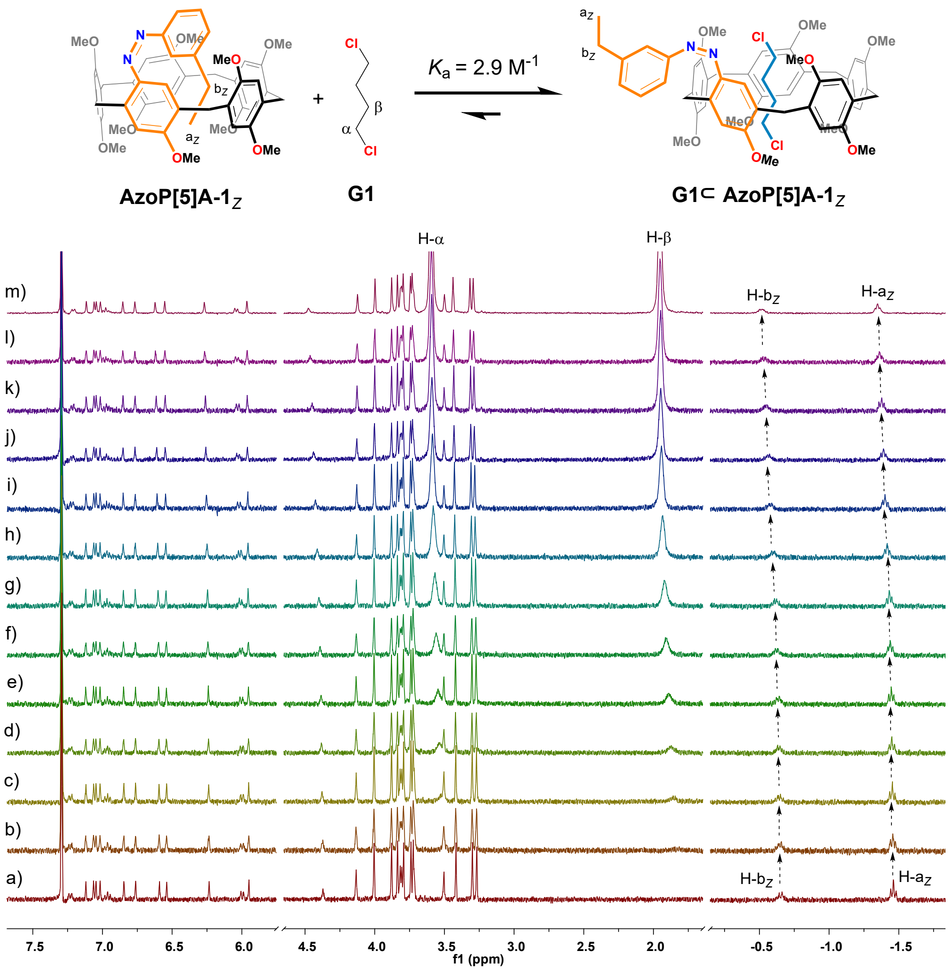

Fig. S56 Schematic representation for the complexation behavior of **AzoP[5]A-1$_Z$** and **G1**, and partial ^{1}H NMR spectra (400 MHz, $CDCl_3$, 298 K) for the solution of the PSS$_Z$ (390 nm) mixtures of **AzoP[5]A-1$_Z$** (2.0 mM) in the presence of a) 0, b) 0.25, c) 0.5, d) 0.75, e) 1.0, f) 1.5, g) 2.0, h) 3.0, i) 4.0, j) 5.0, k) 6.0, l) 7.0, and m) 8.0 equiv. of **G1**.

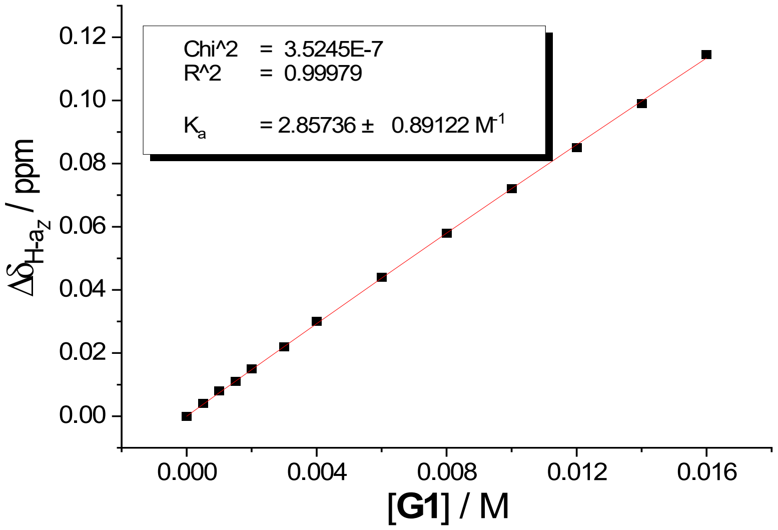

Fig. S57 Plot of the chemical shift changes of proton H-a$_Z$ of **AzoP[5]A-1$_Z$** versus the concentration of **G1**. The fit of the data for H-a$_Z$ (δ ppm) from Fig. S56 to a 1:1 binding model.

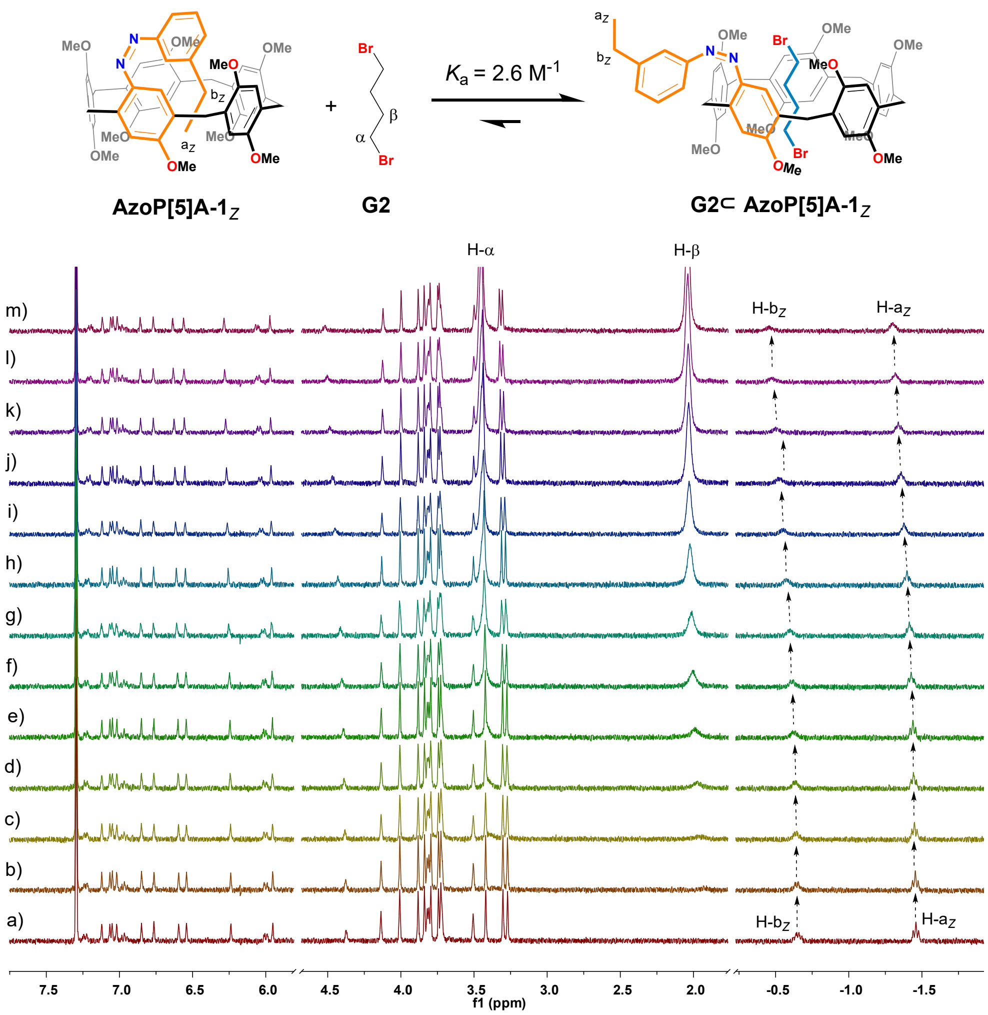

Fig. S58 Schematic representation for the complexation behavior of **AzoP[5]A-1**$_Z$ and **G2**, and partial ^{1}H NMR spectra (400 MHz, $CDCl_3$, 298 K) for the solution of the PSS$_Z$ (390 nm) mixtures of **AzoP[5]A-1**$_Z$ (2.0 mM) in the presence of a) 0, b) 0.25, c) 0.5, d) 0.75, e) 1.0, f) 1.5, g) 2.0, h) 3.0, i) 4.0, j) 5.0, k) 6.0, l) 7.0, and m) 8.0 equiv. of **G2**.

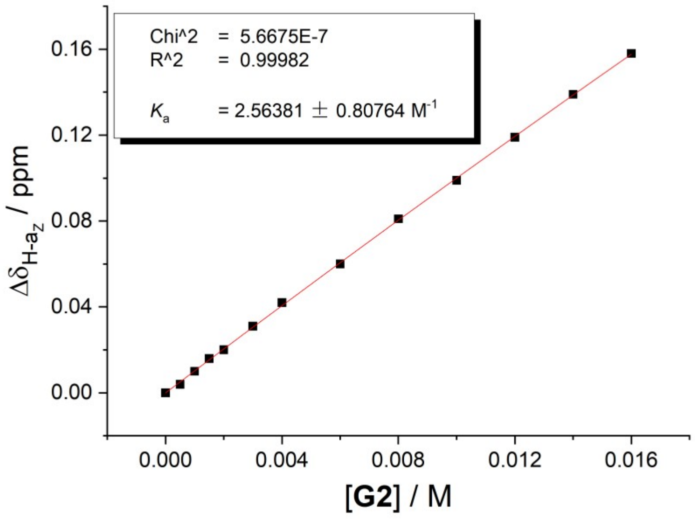

Fig. S59 Plot of the chemical shift changes of proton H-a$_Z$ of **AzoP[5]A-1**$_Z$ versus the concentration of **G2**. The fit of the data for H-a$_Z$ (δ ppm) from Fig. S58 to a 1:1 binding model.

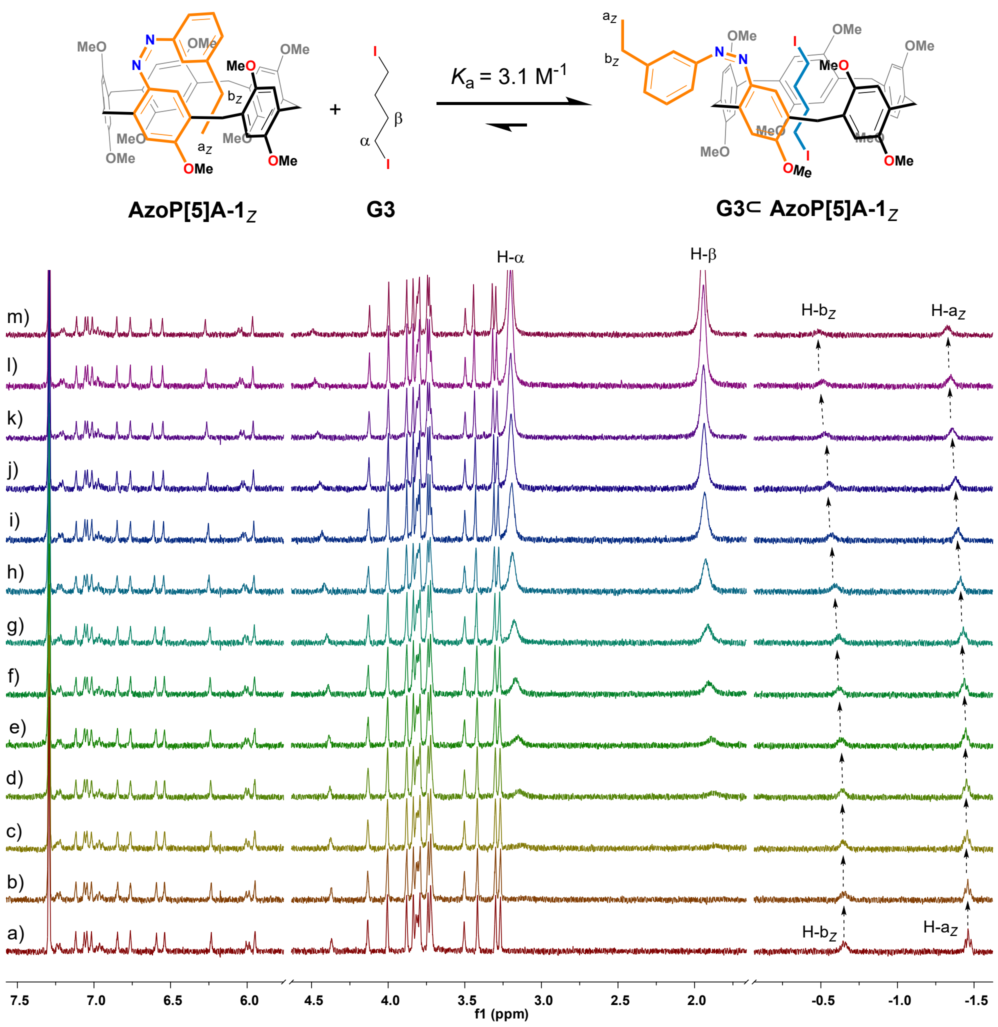

Fig. S60 Schematic representation for the complexation behavior of **AzoP[5]A-1**$_Z$ and **G3**, and partial ^{1}H NMR spectra (400 MHz, $CDCl_3$, 298 K) for the solution of the PSS$_Z$ (390 nm) mixtures of **AzoP[5]A-1**$_Z$ (2.0 mM) in the presence of a) 0, b) 0.25, c) 0.5, d) 0.75, e) 1.0, f) 1.5, g) 2.0, h) 3.0, i) 4.0, j) 5.0, k) 6.0, l) 7.0, and m) 8.0 equiv. of **G3**.

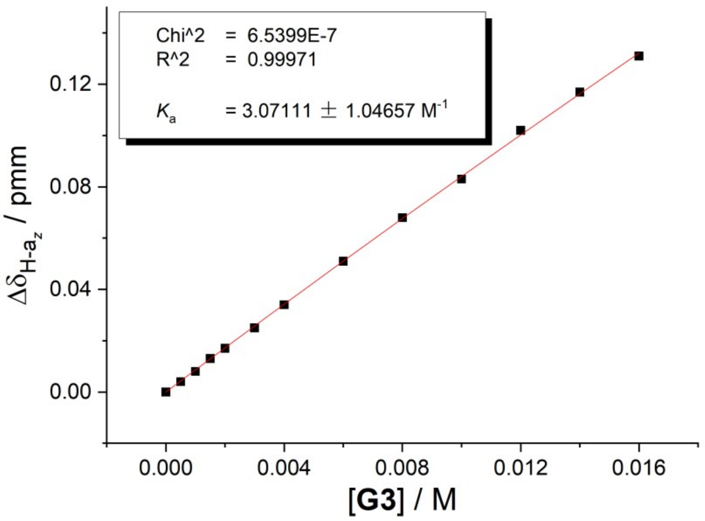

Fig. S61 Plot of the chemical shift changes of proton H-a$_Z$ of **AzoP[5]A-1**$_Z$ versus the concentration of **G3**. The fit of the data for H-a$_Z$ (δ ppm) from Fig. S60 to a 1:1 binding model.

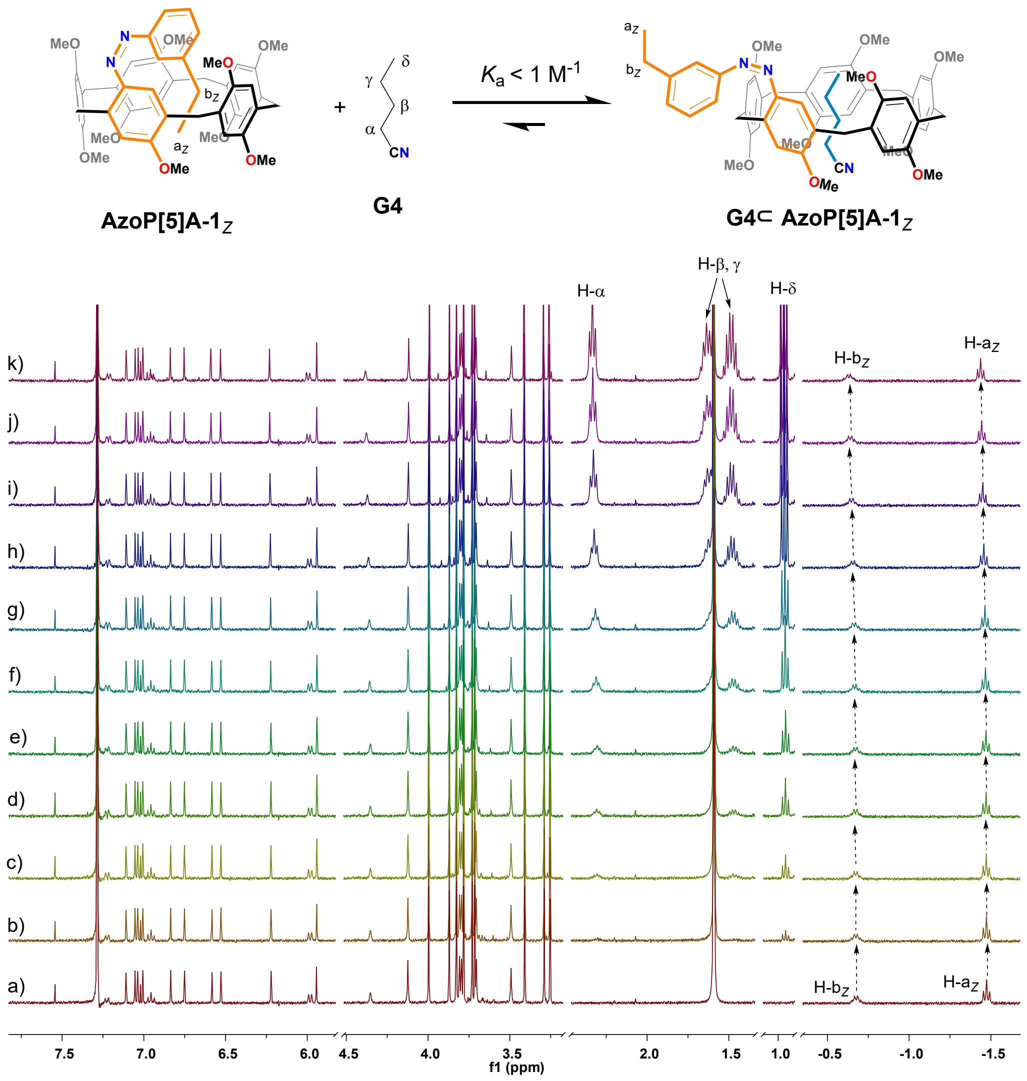

Fig. S62 Schematic representation for the complexation behavior of **AzoP[5]A-1**$_Z$ and **G4**, and partial ^{1}H NMR spectra (400 MHz, $CDCl_3$, 298 K) for the solution of the PSS$_Z$ (390 nm) mixtures of **AzoP[5]A-1**$_Z$ (2.0 mM) in the presence of a) 0, b) 0.25, c) 0.5, d) 0.75, e) 1.0, f) 1.6, g) 2.3, h) 3.8, i) 5.0, j) 6.8, and k) 8.0 equiv. of **G4**.

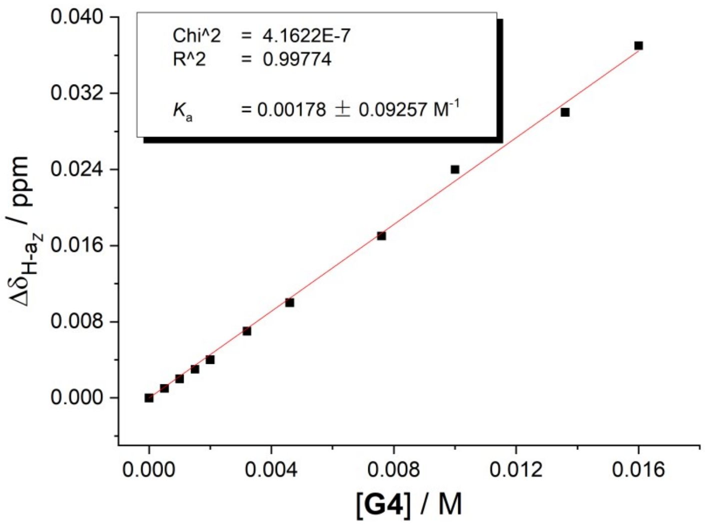

Fig. S63 Plot of the chemical shift changes of proton H-a$_Z$ of **AzoP[5]A-1**$_Z$ versus the concentration of **G4**. The fit of the data for H-a$_Z$ (δ ppm) from Fig. S62 to a 1:1 binding model.

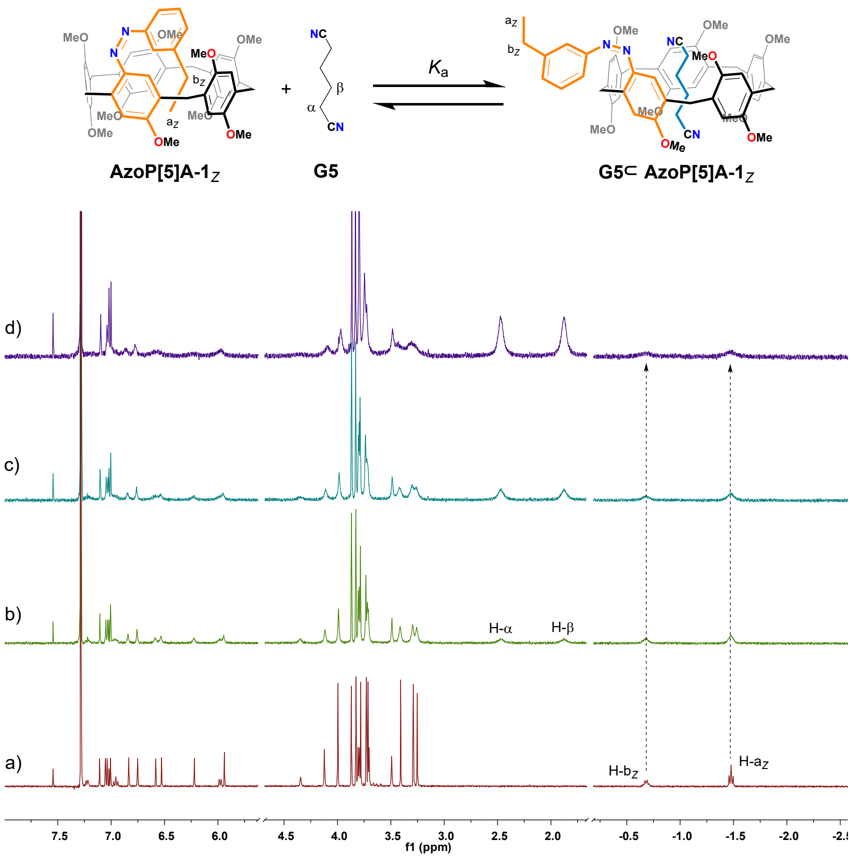

Fig. S64 Schematic representation for the complexation behavior of **AzoP[5]A-1**$_Z$ and **G5**, and partial ^{1}H NMR spectra (400 MHz, $CDCl_3$, 298 K) of the solution of the PSS$_Z$ (390 nm) mixtures of **AzoP[5]A-1** (2.0 mM) in the presence of a) 0, b) 0.50, c) 1.0, and d) 2.0 equiv. of **G5**. Note: when the guest **G5** was gradually introduced, the proton signals (a$_Z$ and b$_Z$) of **AzoP[5]A-1**$_Z$ only become broadened but exhibit negligible chemical shift changes, thus, the K_a value could not be determined by ^{1}H NMR titration or single-point method.

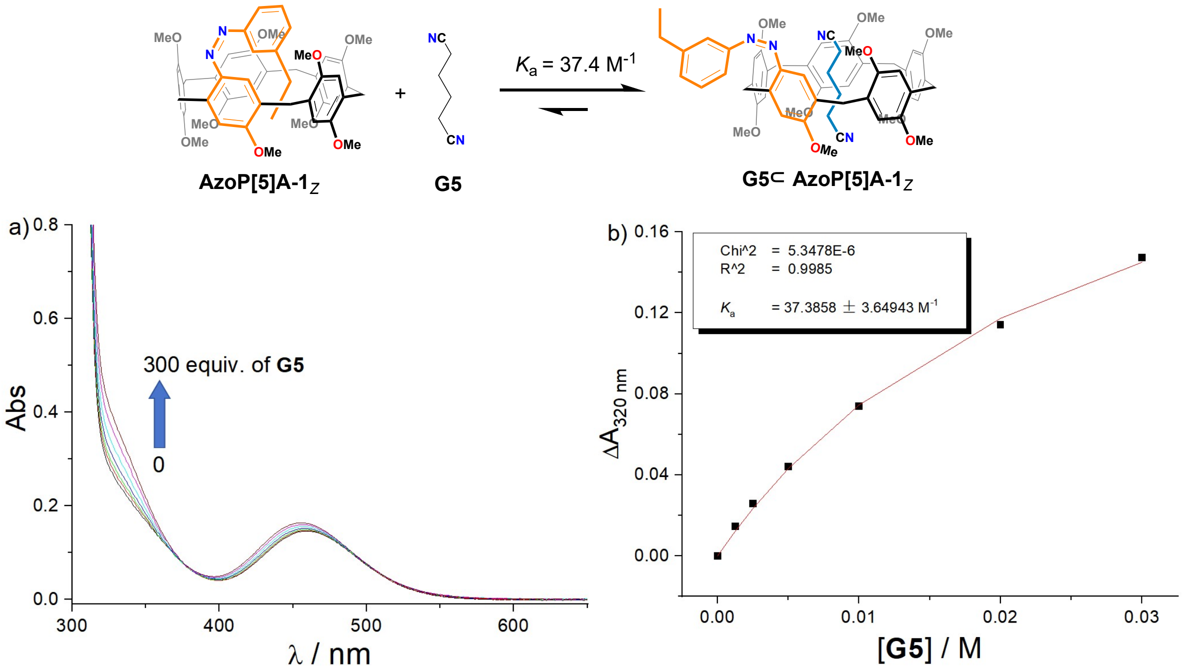

Fig. S65 a) UV-vis absorption spectral of the PSS$_Z$ (390 nm) mixtures of **AzoP[5]A-1** (0.1 mM) in $CHCl_3$ at 298 K in presence of 0 to 300 equiv. of **G5**. b) Plot of the absorbance changes at 320 nm versus the concentration of **G5**. The solid line represents the best least-squares fit of the data to a 1:1 binding model.

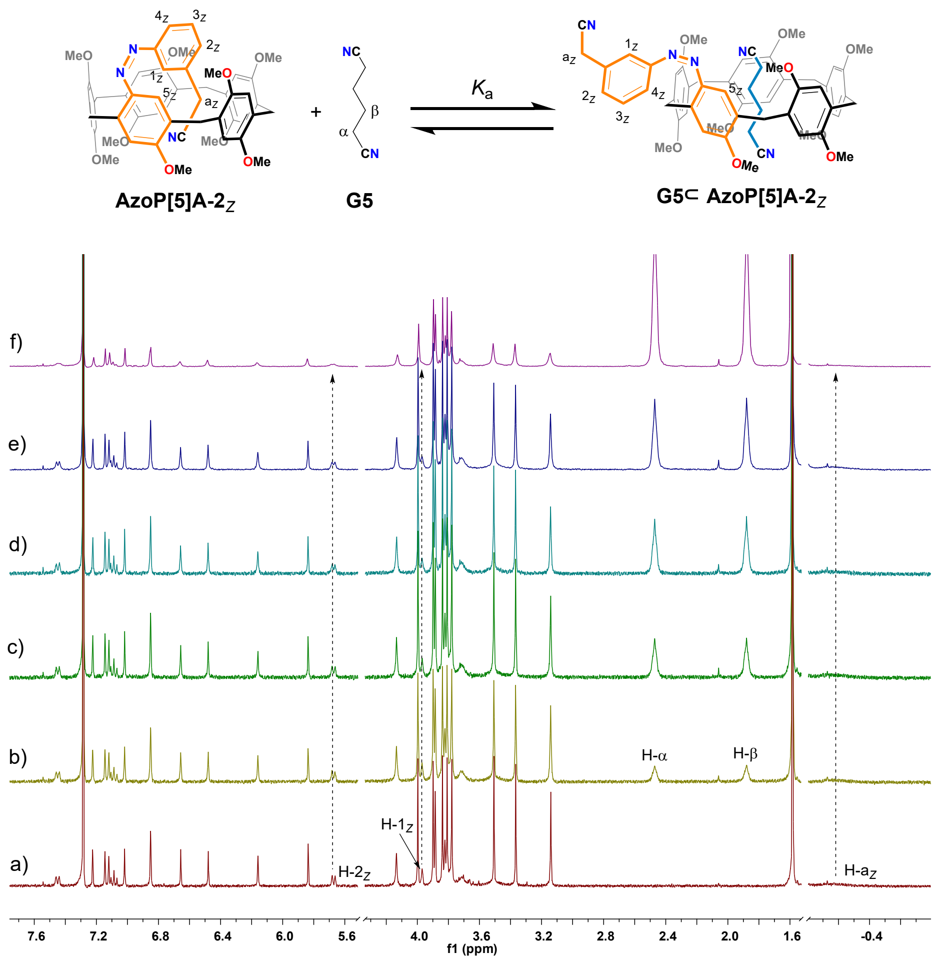

Fig. S66 Schematic representation for the complexation behavior of **AzoP[5]A-2**$_Z$ and **G5**, as well as the partial ^{1}H NMR spectra (400 MHz, $CDCl_3$, 298 K) for the PSS$_Z$ (390 nm) mixtures of **AzoP[5]A-2** (2.0 mM) in the presence of a) 0, b) 0.50, c) 1.0, d) 1.5, e) 2.0 and f) 8.0 equiv. of **G5**. Note: when the guest **G5** was gradually introduced, the proton signals (a$_Z$ and b$_Z$) of **AzoP[5]A-2**$_Z$ only become broadened but exhibit negligible chemical shift changes, thus, the K_a value could not be determined by ^{1}H NMR titration or single-point method.

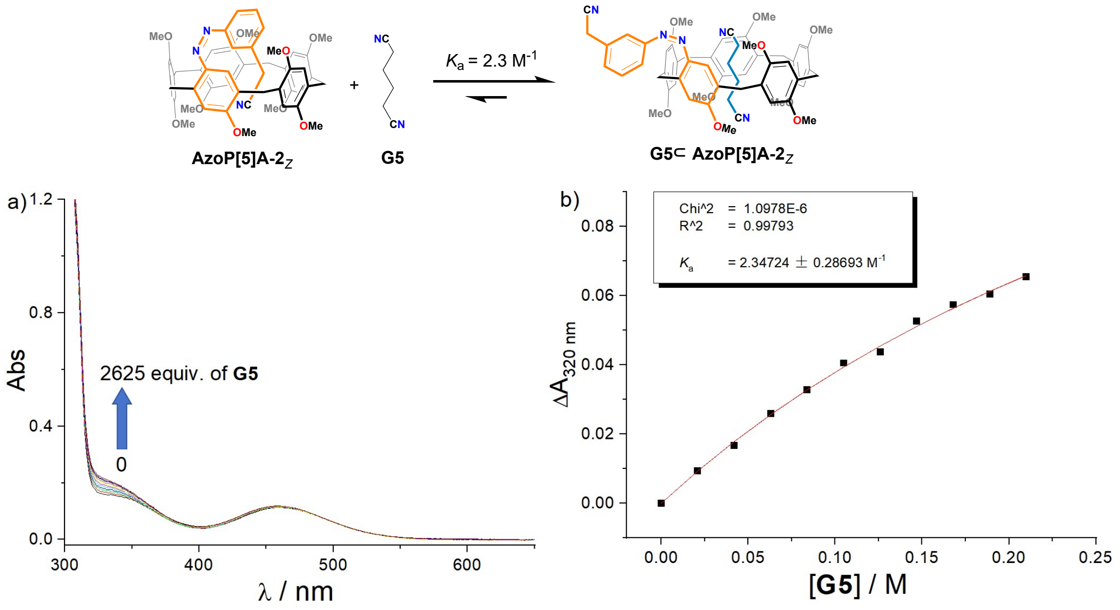

Fig. S67. a) UV-vis absorption spectral of the PSS$_Z$ (390 nm) mixtures of **AzoP[5]A-2** (0.08 mM) in $CHCl_3$ at 298 K in presence of 0 to 2625 equiv. of **G5**. b) Plot of the absorbance changes at 320 nm versus the concentration of **G5**. The solid line represents the best least-squares fit of the data to a 1:1 binding model.

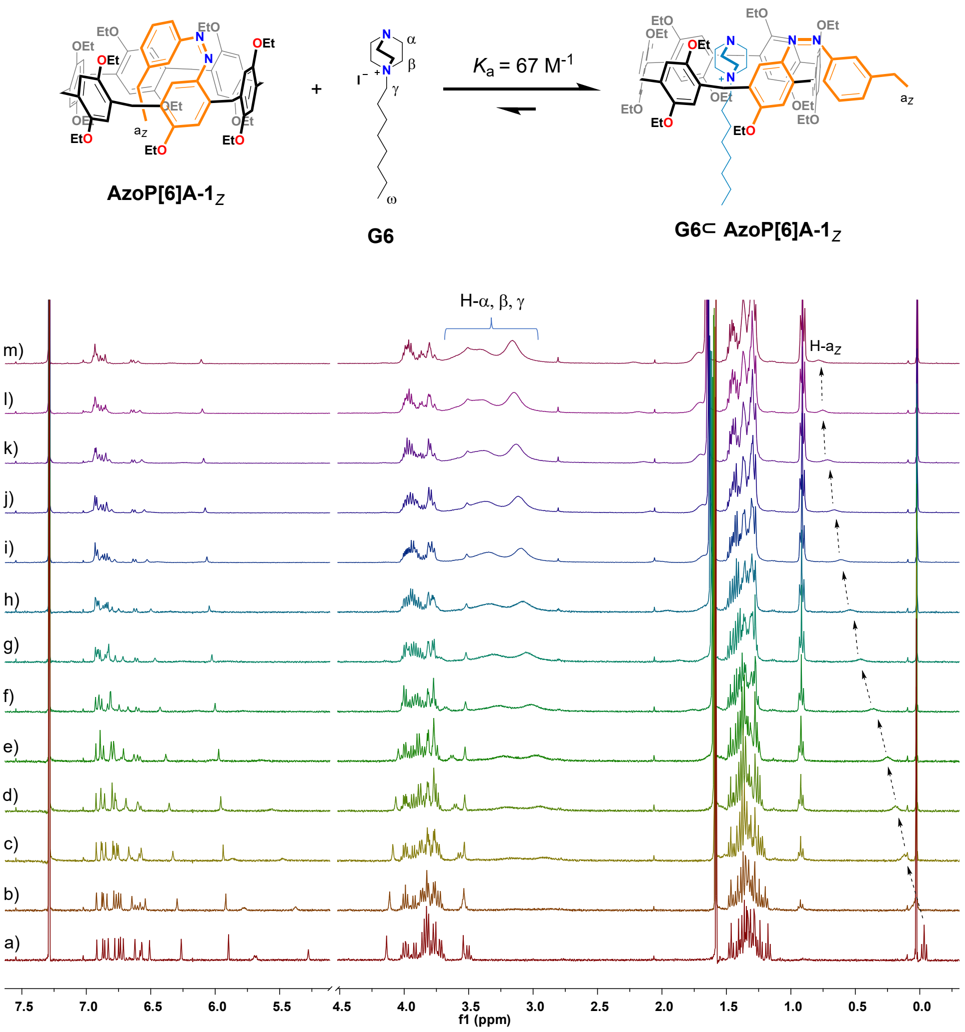

Fig. S68 Schematic representation of the complexation behavior of **AzoP[6]A-1**$_Z$ and **G6**, as well as the partial ^{1}H NMR spectra (400 MHz, $CDCl_3$, 298 K) for the PSS$_Z$ (390 nm) mixtures of **AzoP[6]A-1** (2.0 mM) in the presence of a) 0, b) 0.5, c) 1.0, d) 1.5, e) 2.0, f) 3.0, g) 4.0, h) 5.0, i) 6.0, j) 7.0, k) 8.0, l) 9.0, and m) 10 equiv. of **G6**.

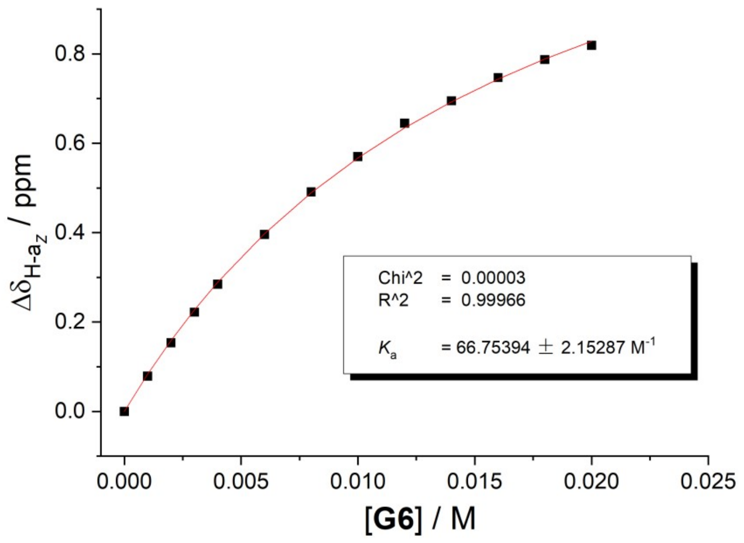

Fig. S69 Plot of the chemical shift changes of proton H-a$_Z$ of **AzoP[6]A-1**$_Z$ versus the concentration of **G6**. The fit of the data for H-a$_Z$ (δ ppm) from Fig. S68 to a 1:1 binding model.

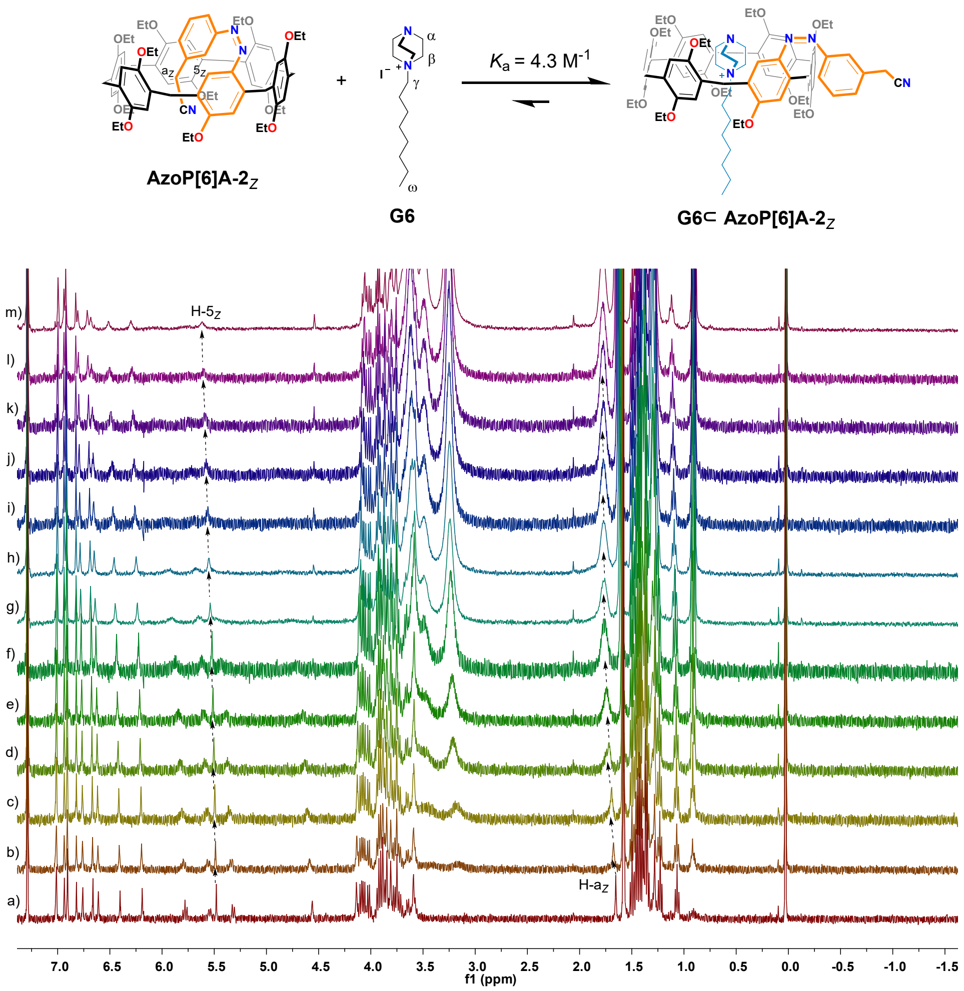

Fig. S70 Schematic representation of the complexation behavior of **AzoP[6]A-2**$_Z$ and **G6**, as well as the partial ^{1}H NMR spectra (400 MHz, $CDCl_3$, 298 K) for the PSS$_Z$ (390 nm) mixtures of **AzoP[6]A-2** (2.0 mM) in the presence of a) 0, b) 0.5, c) 1.0, d) 1.5, e) 2.0, f) 3.0, g) 4.0, h) 5.0, i) 6.0, j) 7.0, k) 8.0, l) 9.0, and m) 10 equiv. of **G6**.

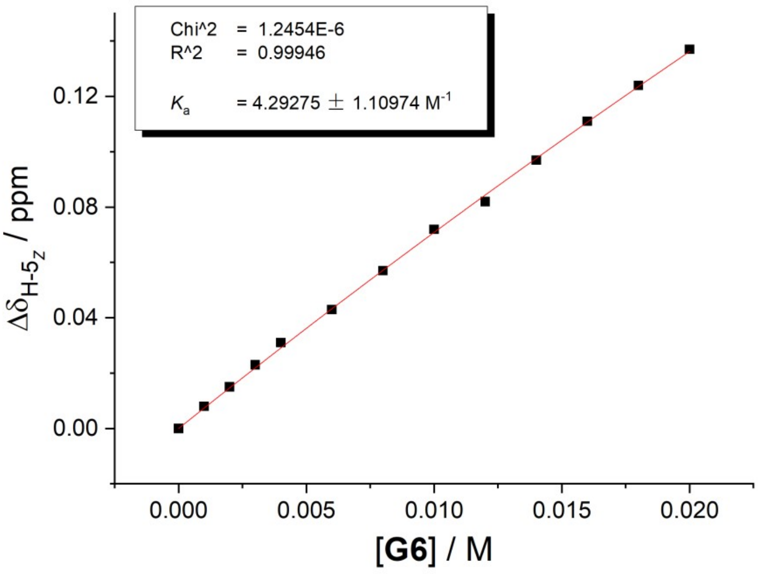

Fig. S71 Plot of the chemical shift changes of proton H-5$_Z$ of **AzoP[6]A-2**$_Z$ versus the concentration of **G6**. The fit of the data for H-5$_Z$ (δ ppm) from Fig. S70 to a 1:1 binding model.

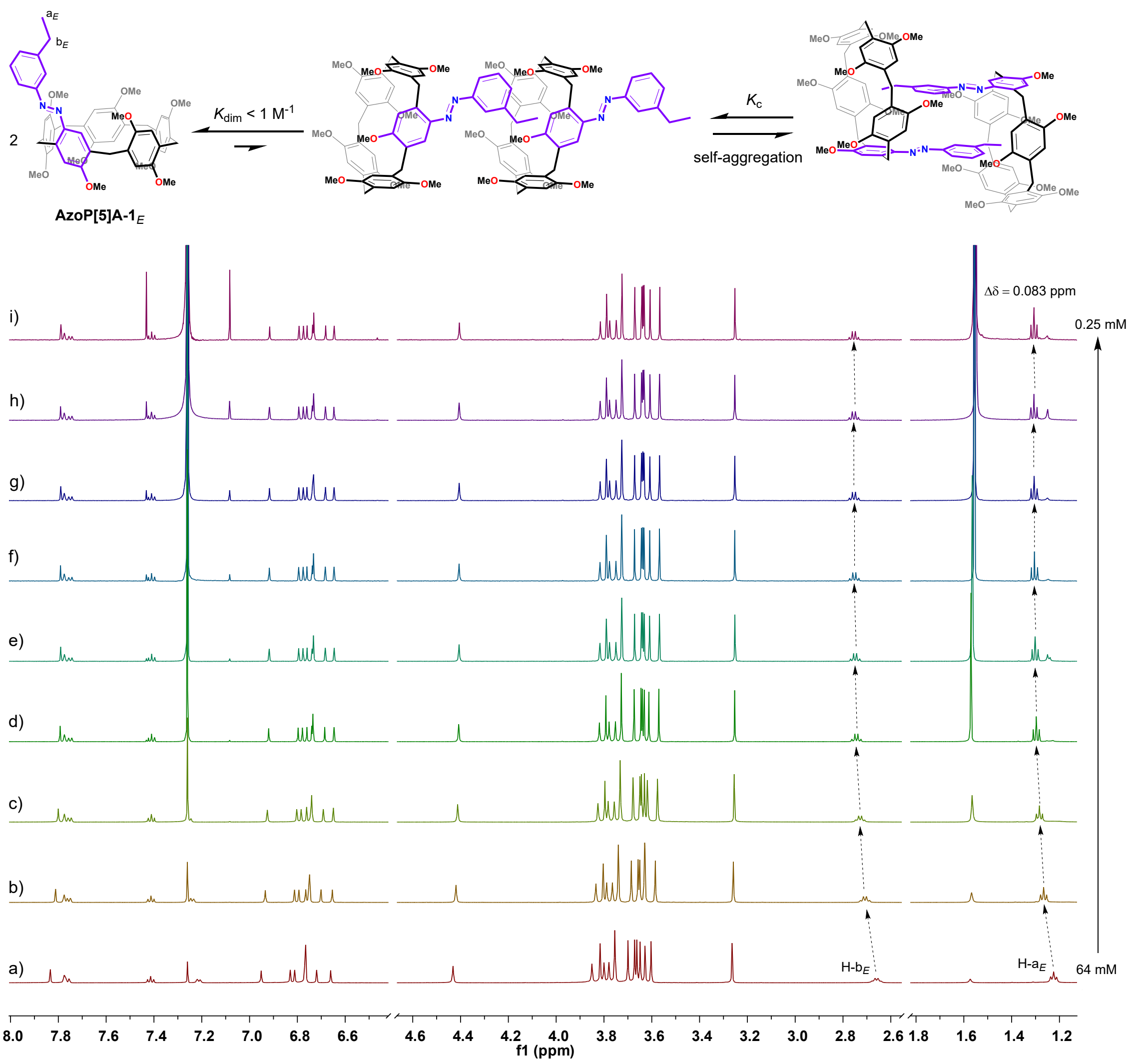

Fig. S72 ^{1}H NMR spectra (600 MHz, $CDCl_3$, 298K) of the solution of **AzoP[5]A-1**$_E$ at different concentrations of a) 64 mM, b) 32 mM, c) 16 mM, d) 8.0 mM, e) 4.0 mM, f) 2.0 mM, g) 1.0 mM, h) 0.5 mM and i) 0.25 mM.

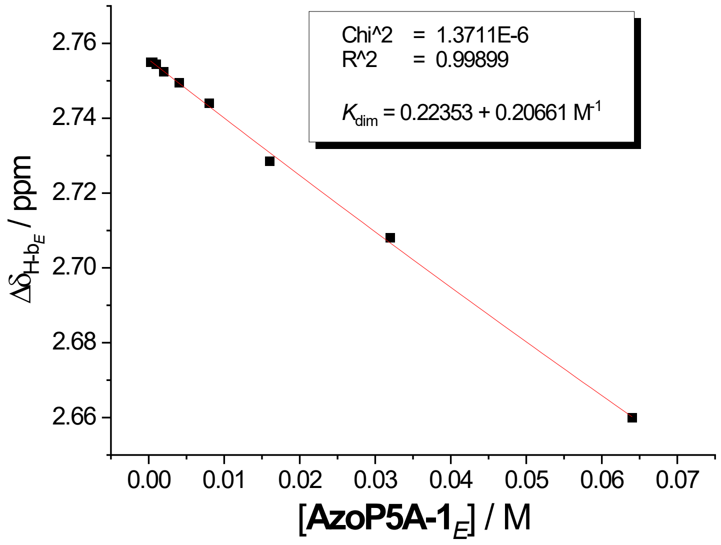

Fig. S73 Plot of the chemical shift of proton H-b$_E$ of **AzoP[5]A-1**$_E$ versus the concentration of itself. The fit of the data for H-b$_E$ (δ ppm) from Fig. S72 to a dimer aggregation model.

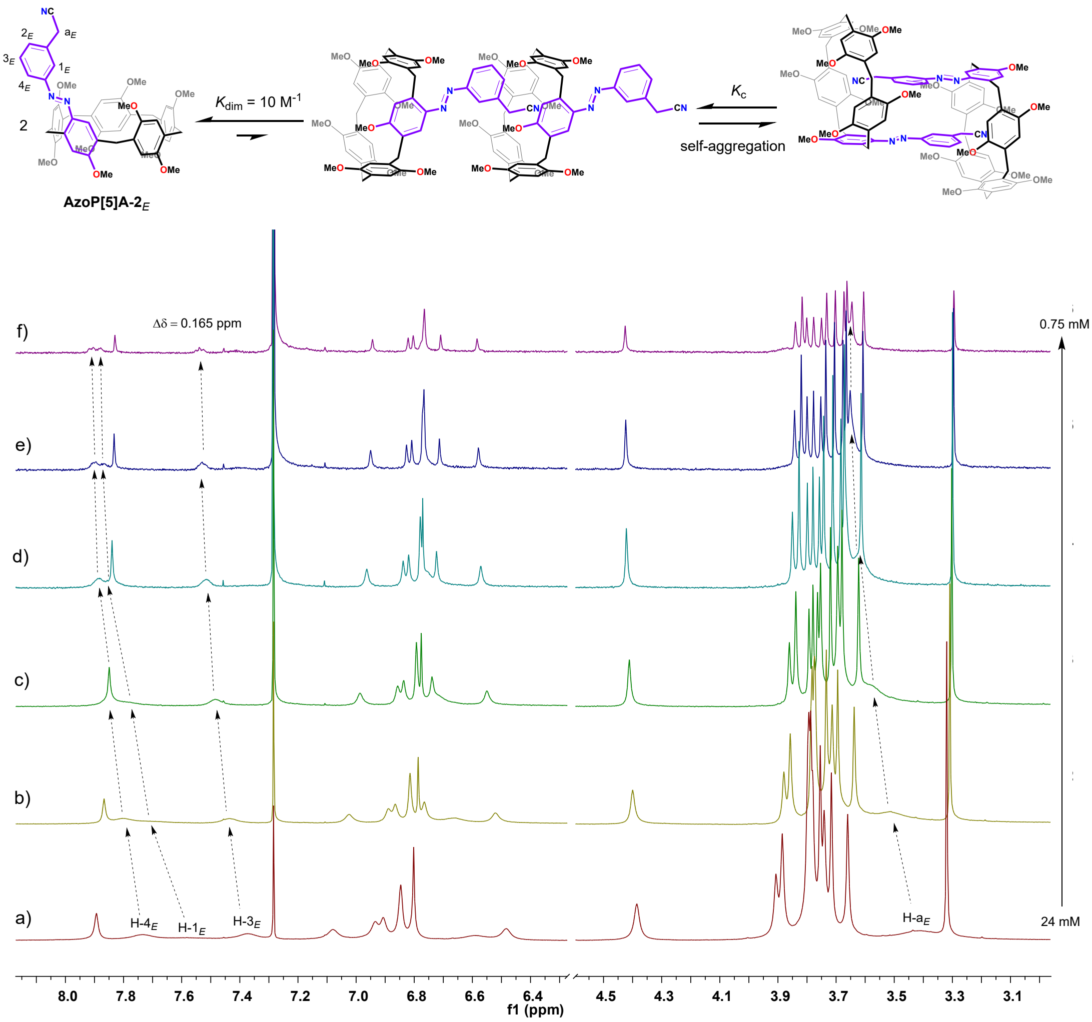

Fig. S74 ^{1}H NMR spectra (600 MHz, $CDCl_3$, 298K) of the solution of **AzoP[5]A-2**$_E$ at different concentrations of a) 24 mM, b) 12 mM, c) 6.0 mM, d) 3.0 mM, e) 1.5 mM, and f) 0.75 mM.

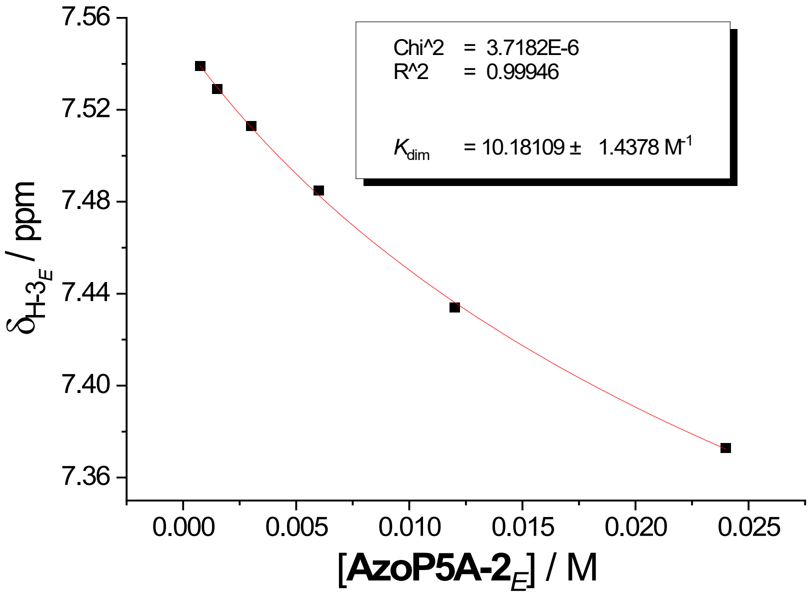

Fig. S75 Plot of the chemical shift of proton H-a$_E$ of **AzoP[5]A-2**$_E$ versus the concentration of itself. The fit of the data for H-b$_E$ (δ ppm) from Fig. S74 to a dimer aggregation model.

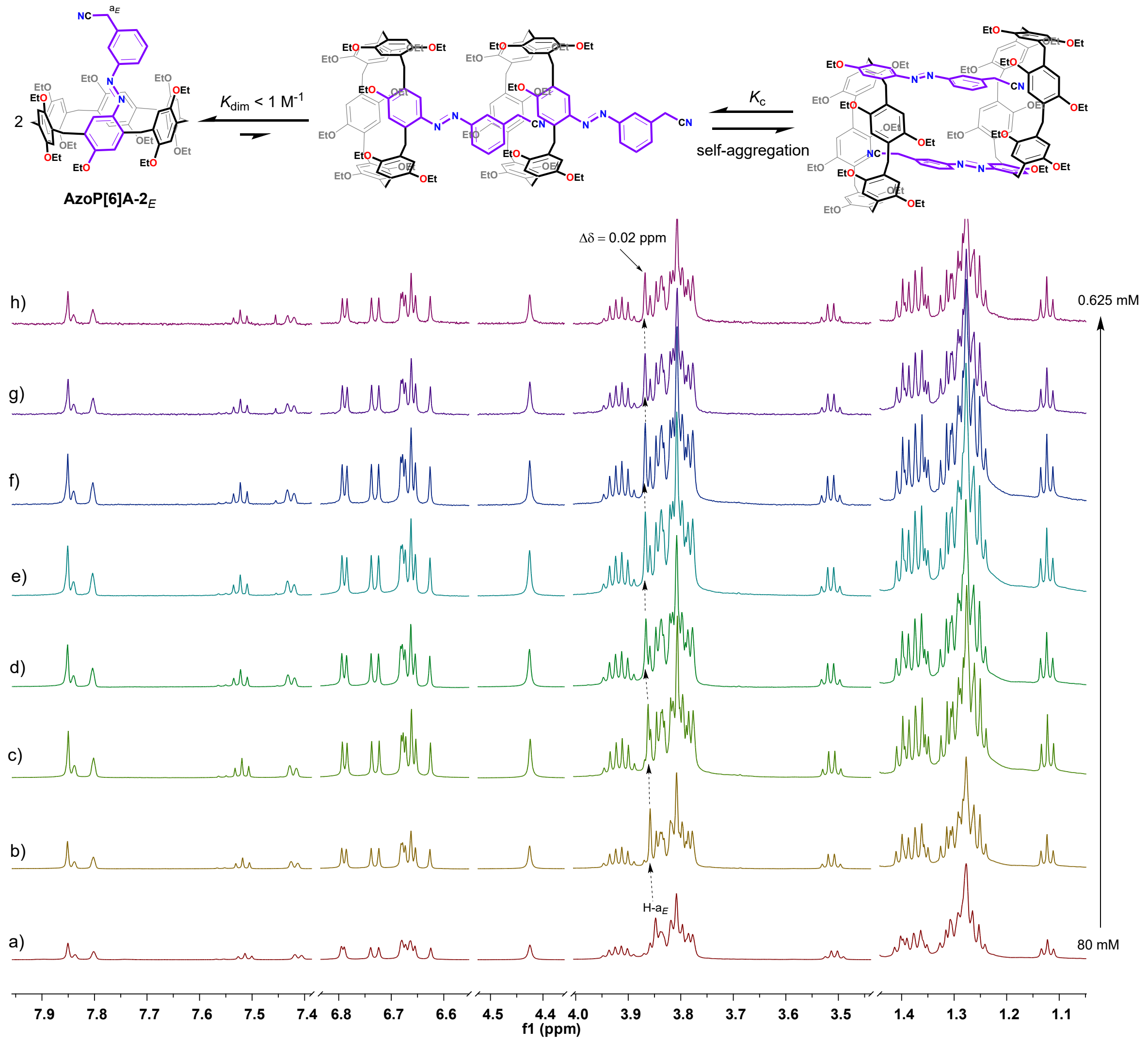

Fig. S76 ^{1}H NMR spectra (600 MHz, $CDCl_3$, 298K) of the solution of **AzoP[6]A-2**$_E$ at different concentrations of a) 80 mM, b) 40 mM, c) 20 mM, d) 10 mM, e) 5.0 mM, f) 2.5 mM, g) 1.25 mM, and h) 0.625 mM.

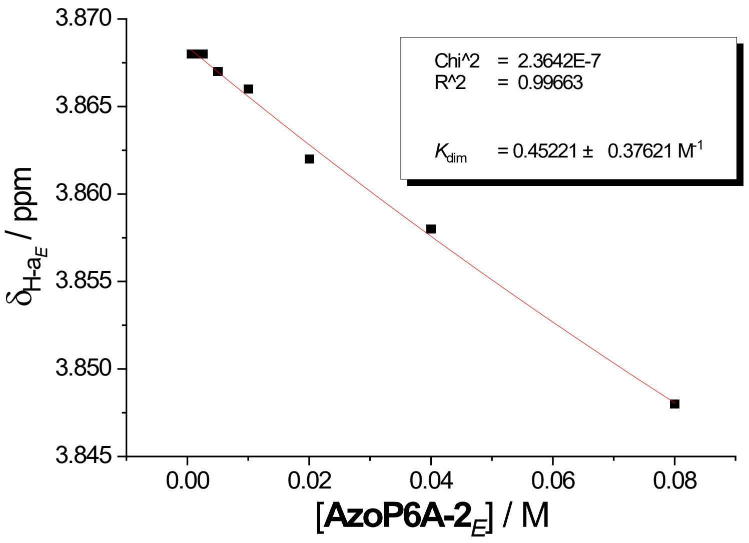

Fig. S77 Plot of the chemical shift of proton H-a$_E$ of **AzoP[6]A-2**$_E$ versus the concentration of itself. The fit of the data for H-b$_E$ (δ ppm) from Fig. S76 to a dimer aggregation model.

Section 7: Photocontrolled Host-Guest Interaction Mediated Macromolecular Self-Assembly.

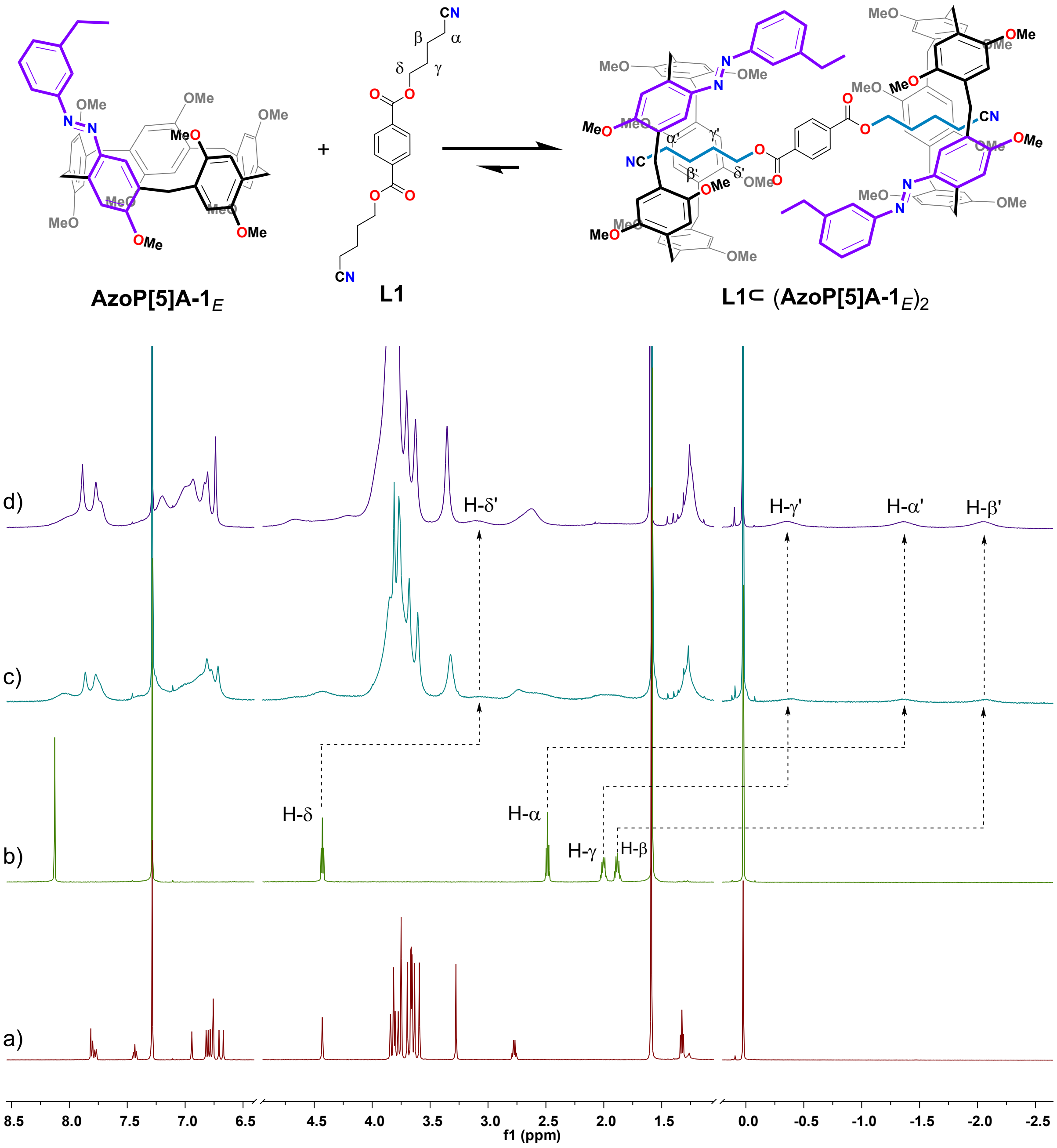

Fig. S78 Schematic representation of the complexation behavior of **AzoP[5]A-1**$_E$ and **L1**, as well as the partial ^{1}H NMR spectra (600 MHz, $CDCl_3$, 298 K) for the solution of a) **AzoP[5]A-1**$_E$ (5.0 mM), b) **L1** (2.5 mM), c) the mixture of **AzoP[5]A-1**$_E$ (2.5 mM) and **L1** (2.5 mM), and d) the mixture of **AzoP[5A-1**$_E$ (20 mM) and **L1** (10 mM)
.

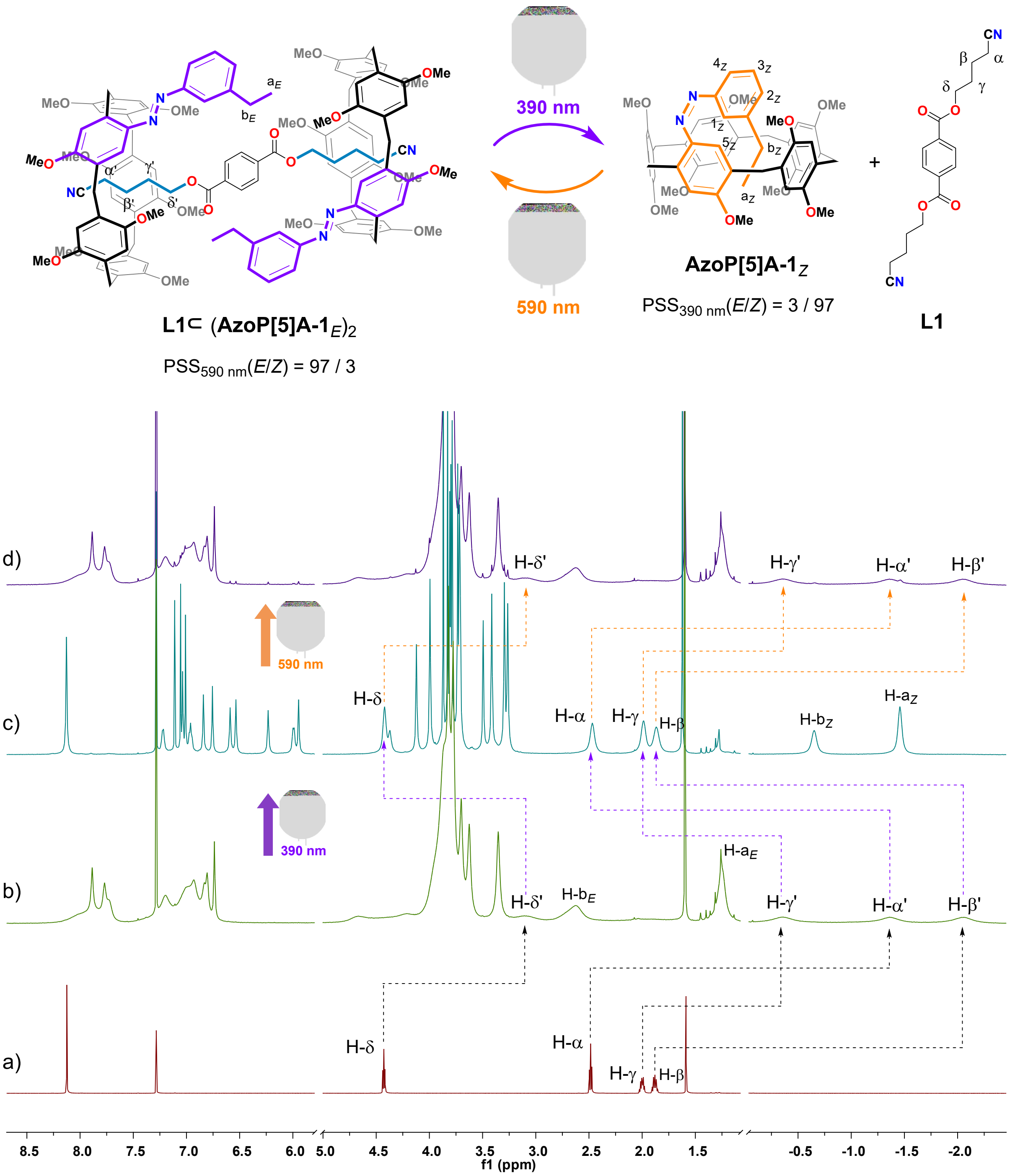

Fig. S79 Schematic representation of the photoswitchable host-guest complexation of **AzoP[5]A-1** and **L1**, as well as the partial ^{1}H NMR spectra (600 MHz, $CDCl_3$, 298 K) for the solution of a) **L1** (10 mM), b) **AzoP[5]A-1**$_E$ (20 mM) and **L1** (10 mM), c) the PSS$_Z$ (390 nm) mixtures of **AzoP[5]A-1** (20 mM) and **L1** (10 mM), and d) the PSS$_E$ (590 nm) mixtures of **AzoP[5]A-1** (20 mM) and **L1** (10 mM).

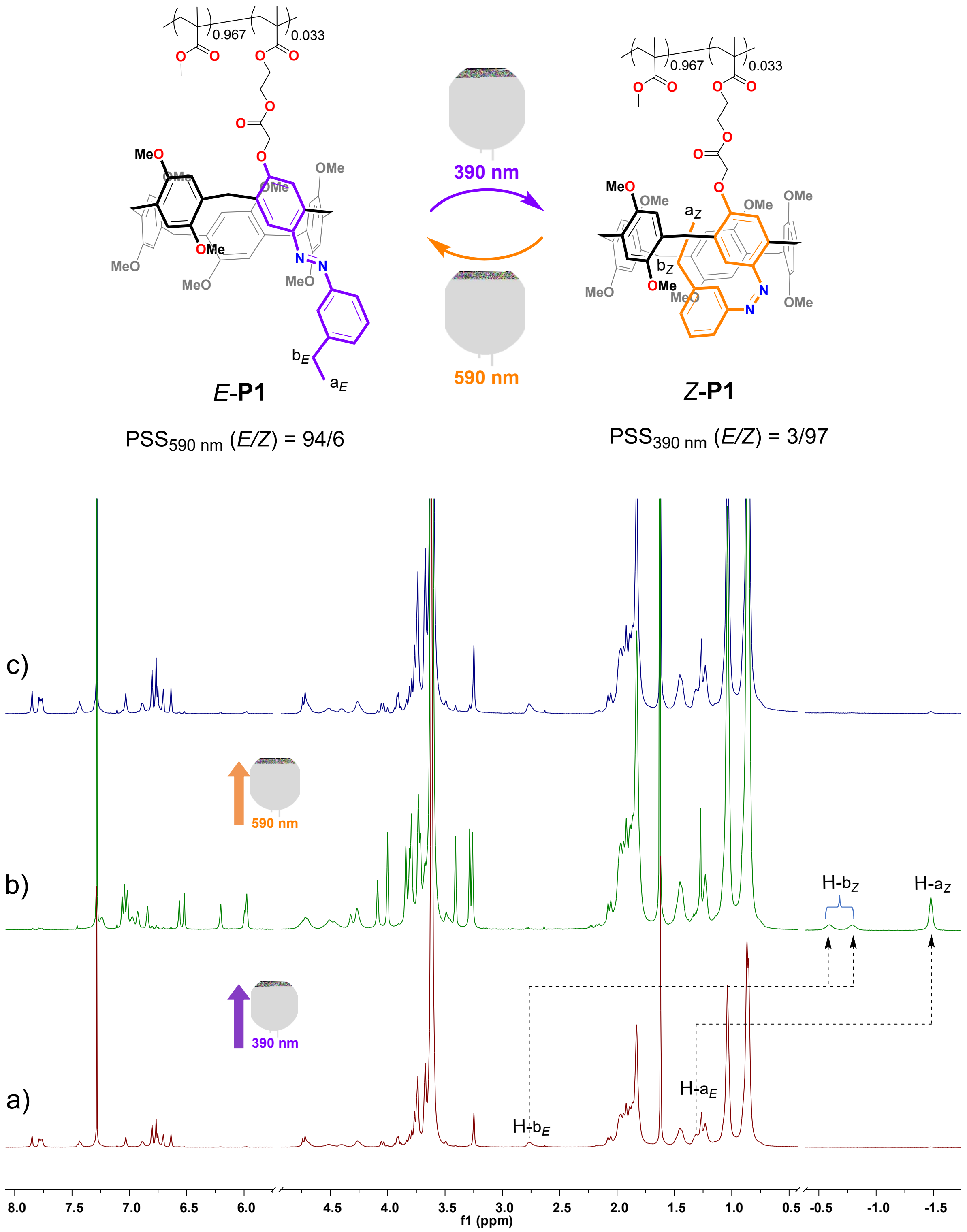

Fig. S80 Schematic representation of the *E*/*Z* photoisomerization of **P1**, and the partial ^{1}H NMR spectra (600 MHz, $CDCl_3$, 298 K) for the solution of a) *E*-**P1** (19.6 mg / mL), b) the PSS_Z (390 nm) mixtures of **P1** (19.6 mg / mL), and c) the PSS_E (590 nm) mixtures of **P1** (19.6 mg / mL).

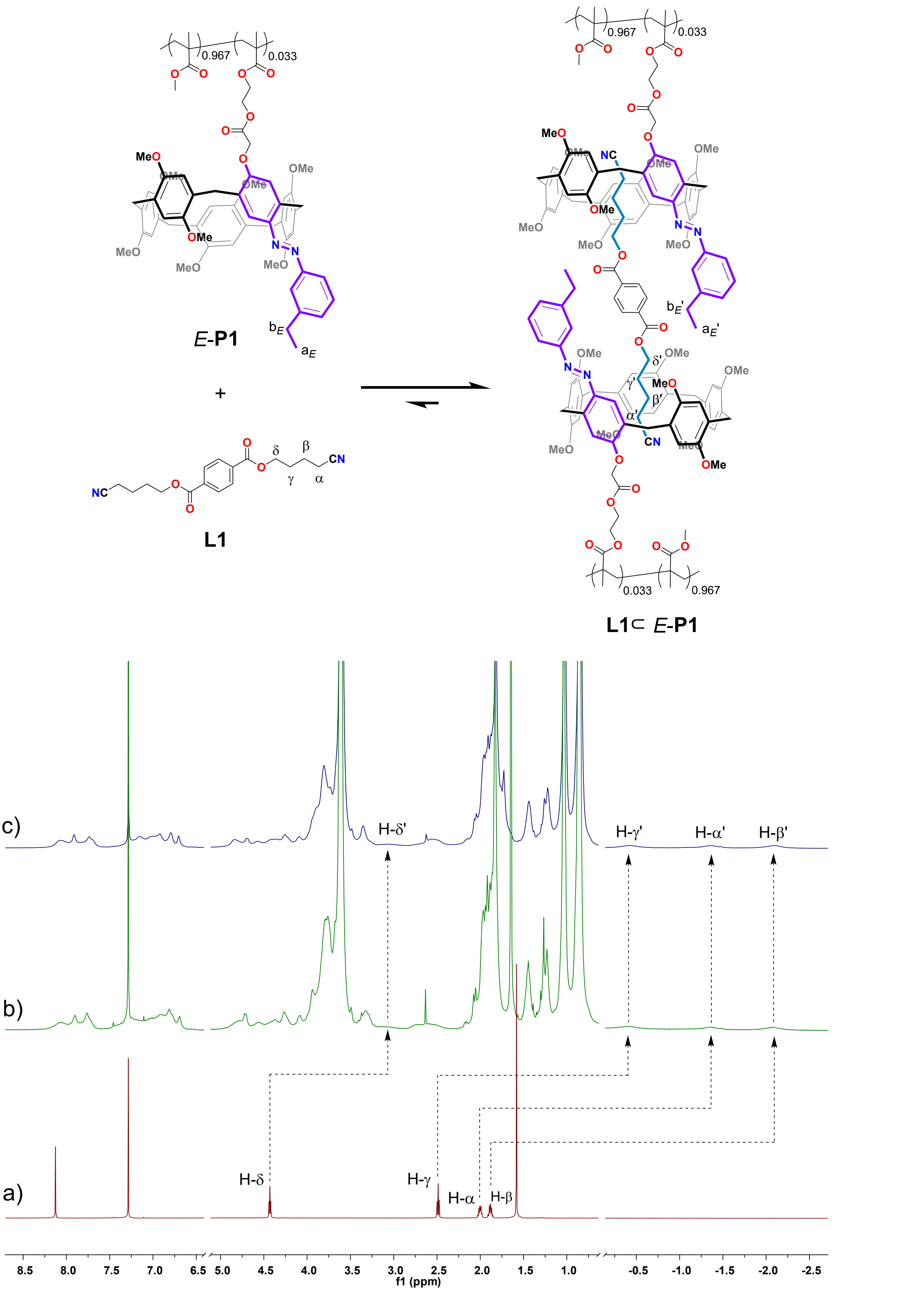

Fig. S81 Schematic representation for the complexation behavior of *E*-**P1** and **L1**, as well as the partial ^{1}H NMR spectra (600 MHz, $CDCl_3$, 298 K) for the solution of a) **L1** (2.5 mM), b) the mixture of *E*-**P1** (19.6 mg /mL, 5.0 mM of **AzoP[5]A-1** unit) and **L1** (2.5 mM), and c) the mixture of *E*-**P1** (78.2 mg /mL, 20 mM of **AzoP[5]A-1** unit) and **L1** (10 mM).

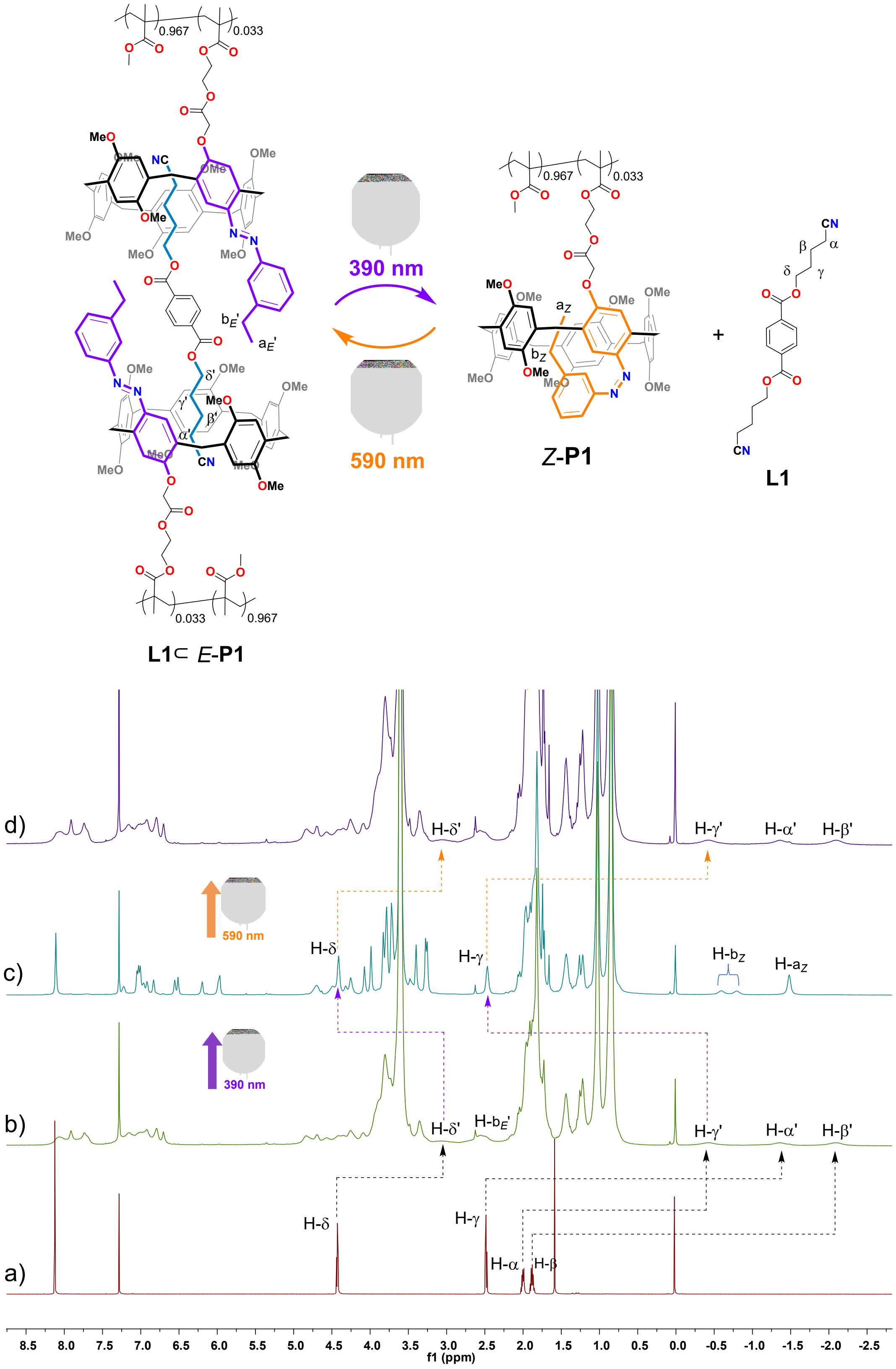

Fig. S82 Schematic representation for the photocontrolled supramolecular polymeric assembly based on a guest linker **L1** and polymer **P1** with **AzoP[5]A-1** unit modified sidechains, as well as the partial ^{1}H NMR spectra (600 MHz, $CDCl_3$, 298 K) for the solution of a) **L1** (10 mM), b) the mixture of *E*-**P1** (78.2 mg /mL, 20 mM of **AzoP[5]A-1** unit) and **L1** (10 mM), c) the PSS_Z (390 nm) mixtures of **P1** (78.2 mg /mL, 20 mM of **AzoP[5]A-1** unit) and **L1** (10 mM), and d) the PSS_E (590 nm) mixtures of **P1** (78.2 mg /mL, 20 mM of **AzoP[5]A-1** unit) and **L1** (10 mM).

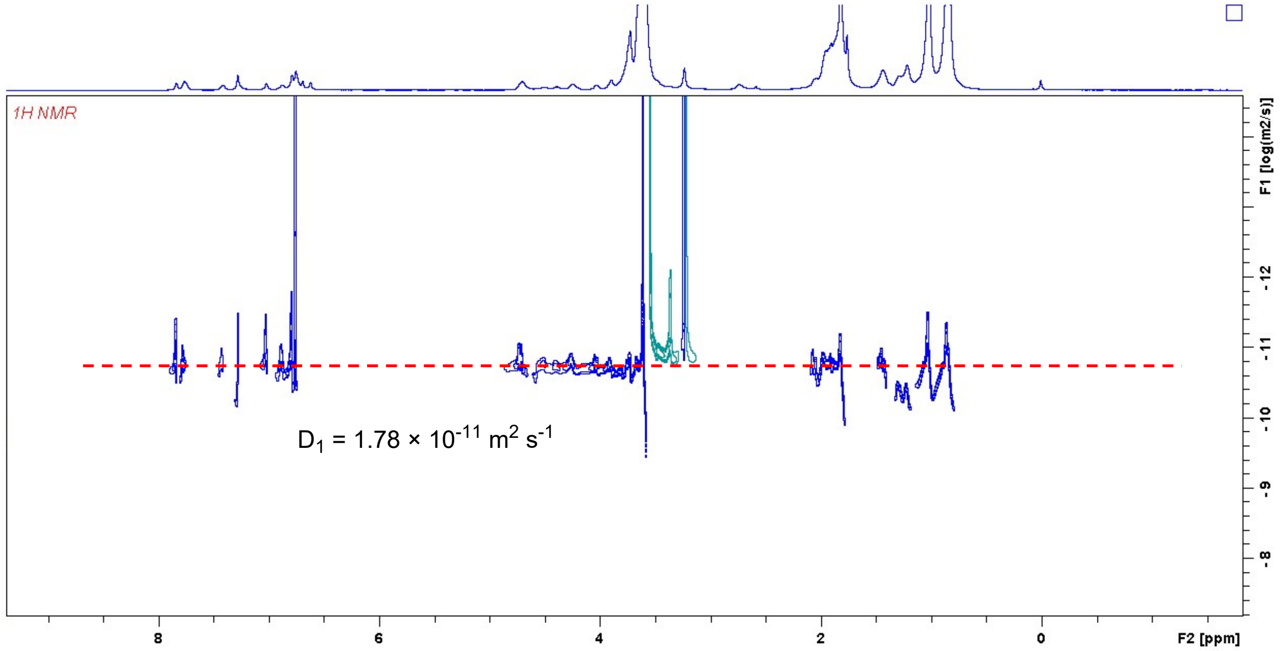

Fig. S83 2D DOSY-NMR spectrum (600 MHz, $CDCl_3$, 298 K) for the solution of *E*-**P1** (78.2 mg /mL).

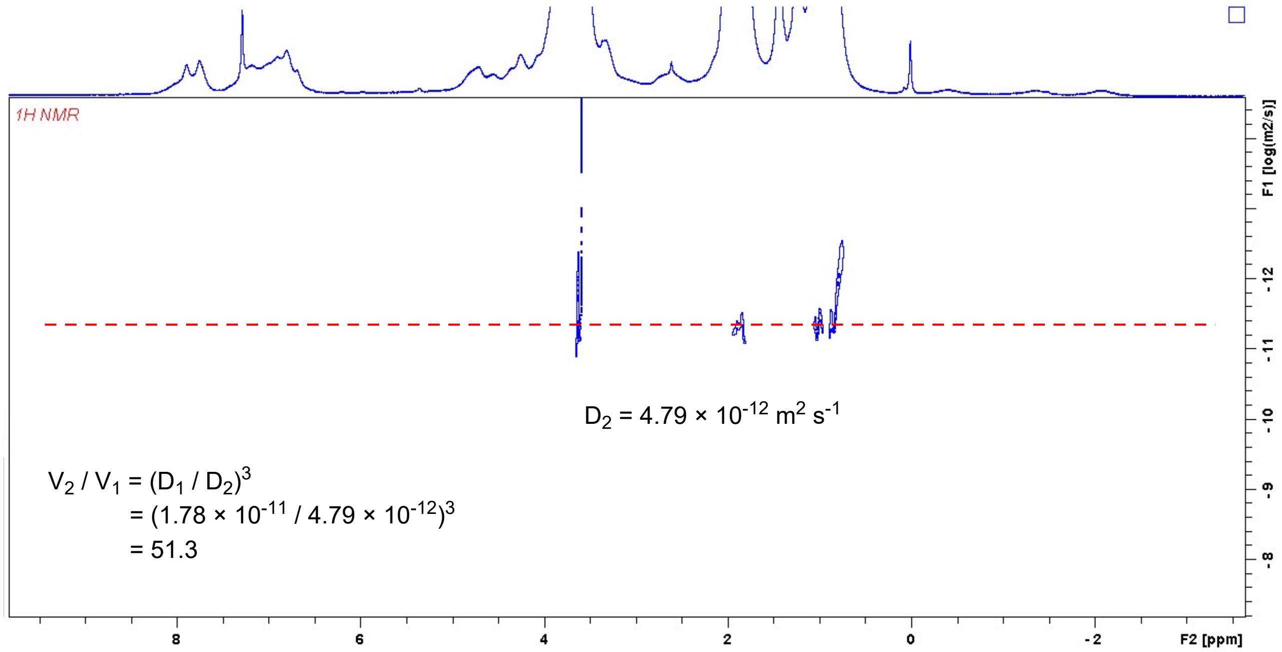

Fig. S84 2D DOSY-NMR spectrum (600 MHz, $CDCl_3$, 298 K) for the solution of *E*-**P1** (78.2 mg /mL, 20 mM of **AzoP[5]A-1**$_E$ unit) and **L1** (10 mM).

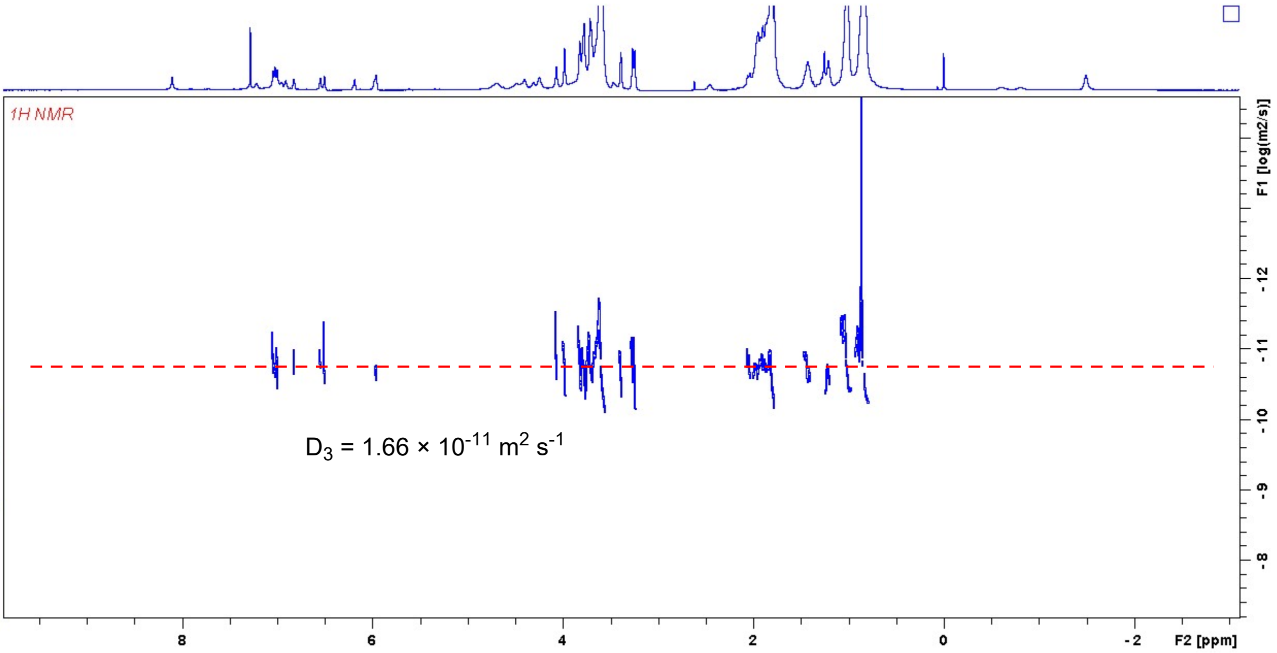

Fig. S85 2D DOSY-NMR spectrum (600 MHz, $CDCl_3$, 298 K) for the solution of PSS_Z (390 nm) mixtures of **P1** (78.2 mg /mL, 20 mM of **AzoP[5]A-1** unit) and **L1** (10 mM).

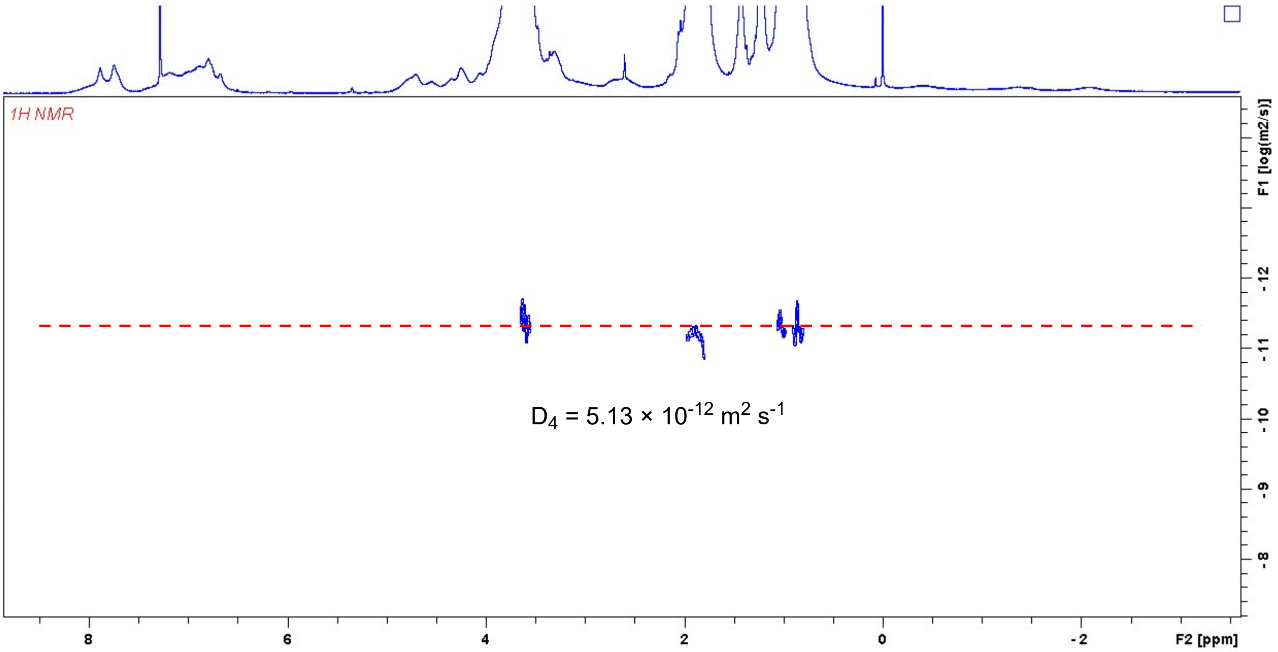

Fig. S86 2D DOSY-NMR spectrum (600 MHz, $CDCl_3$, 298 K) for the solution of PSS_E (590 nm) mixtures of **P1** (78.2 mg /mL, 20 mM of **AzoP[5]A-1** unit) and **L1** (10 mM).

Section 8: The ^{1}H and ^{13}C NMR, 2D COSY and NOESY NMR Spectra for the New Compounds

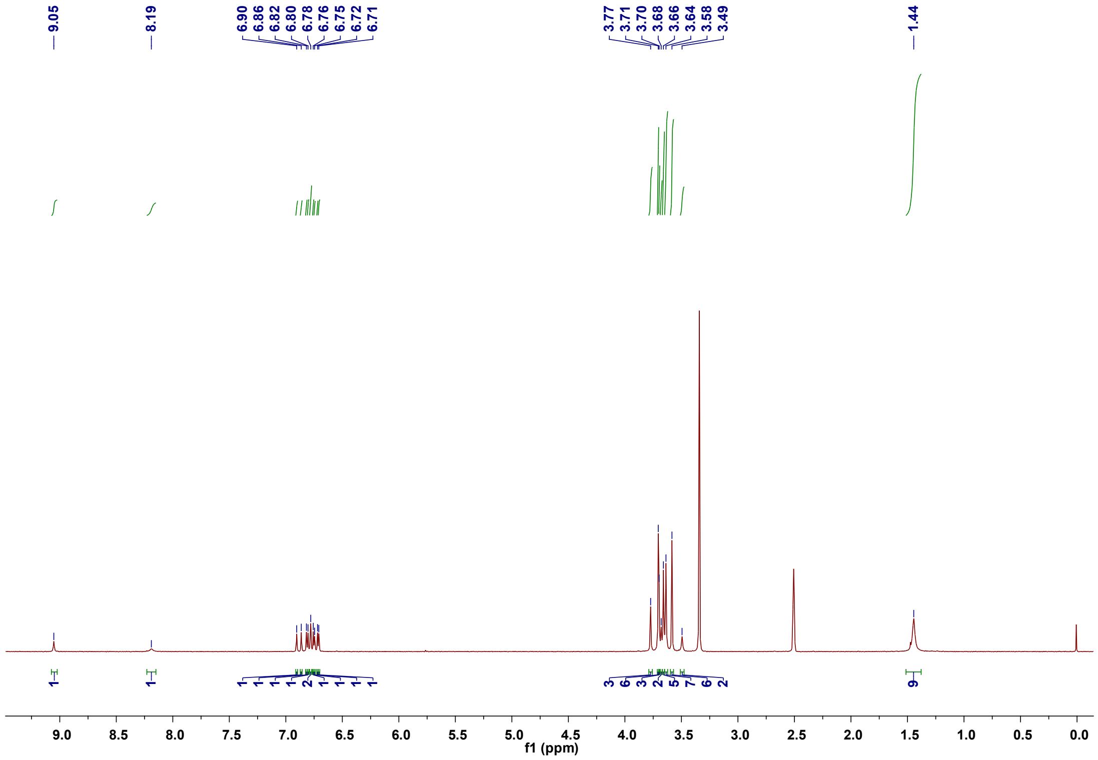

^{1}H NMR spectrum (600 MHz, DMSO-d_6, 298 K) of compound **2**.

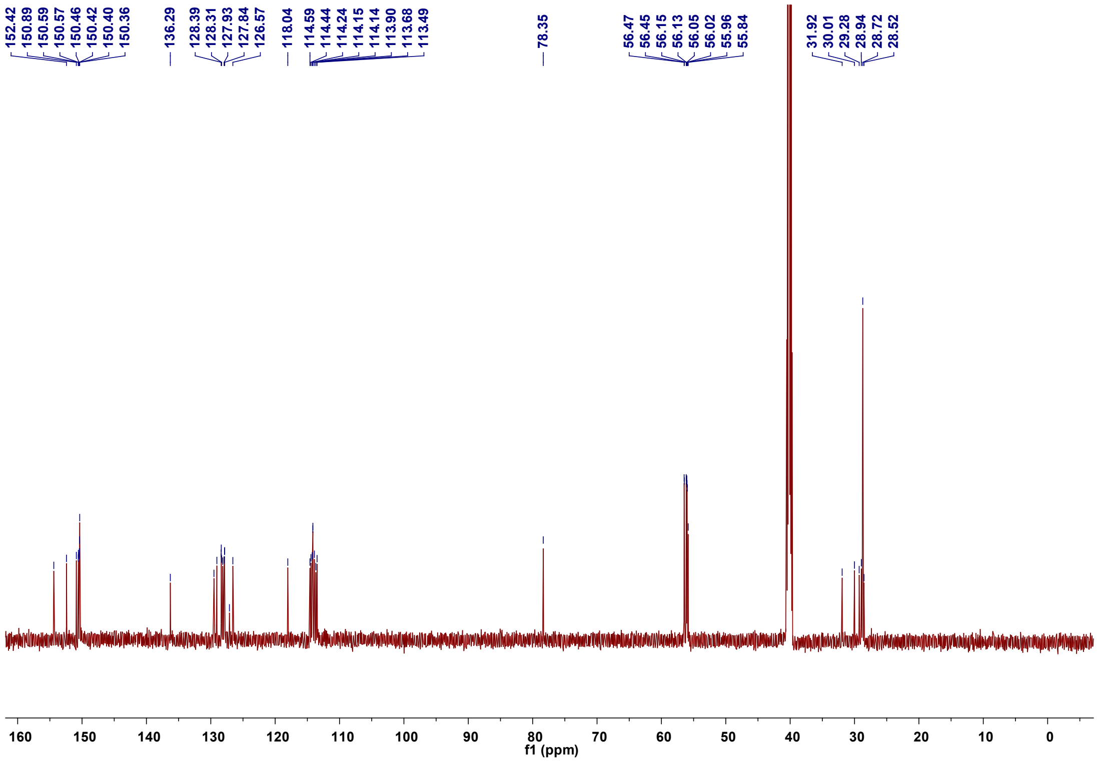

^{13}C NMR spectrum (150 MHz, DMSO-d_6, 298 K) of compound **2**.

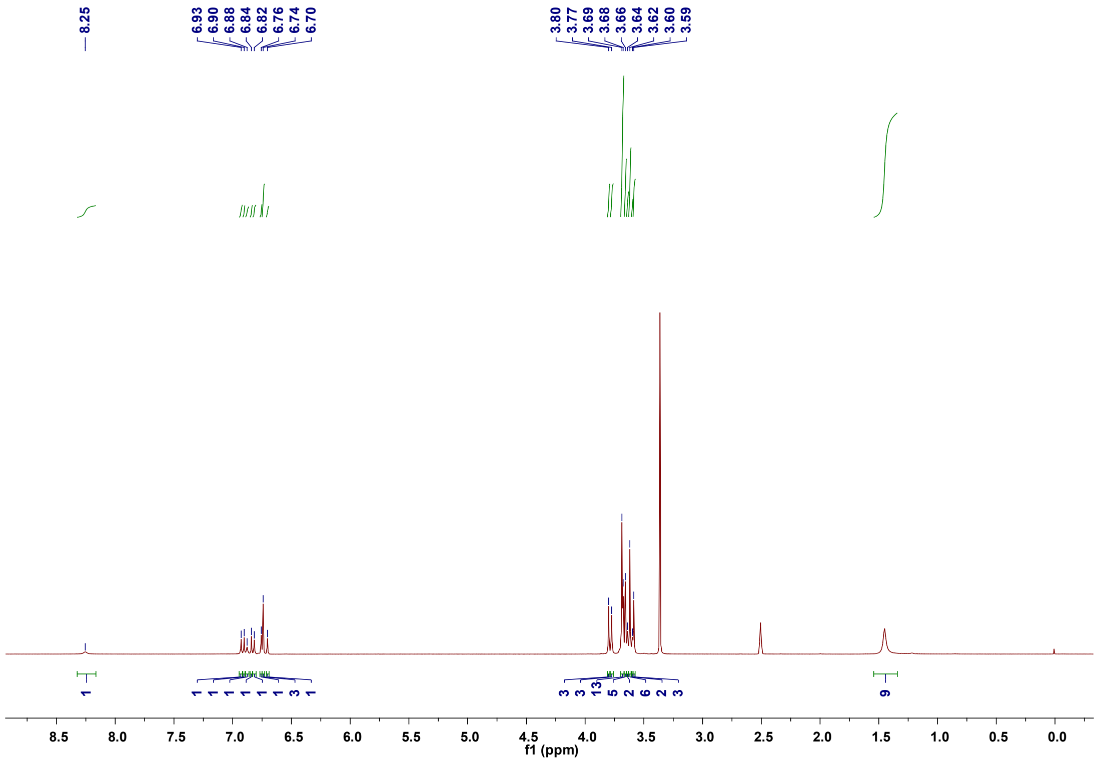

^{1}H NMR spectrum (400 MHz, DMSO-d_6, 298 K) of compound **3**.

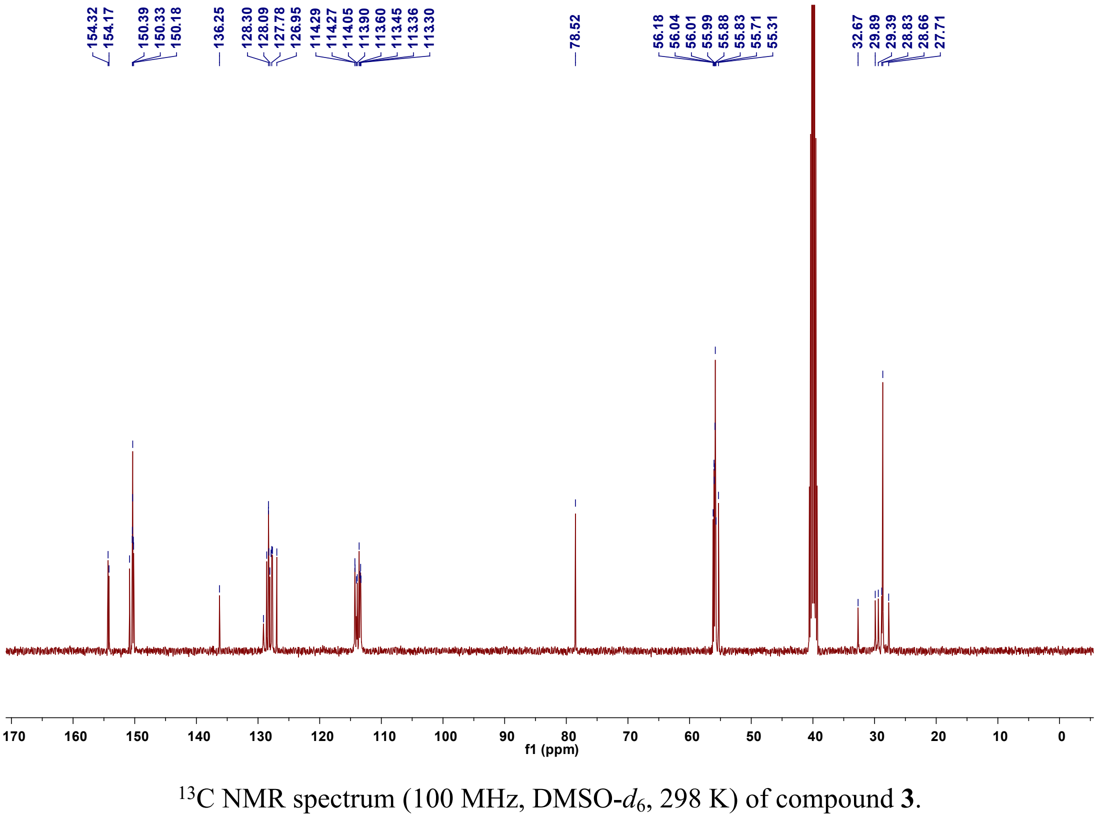

^{13}C NMR spectrum (100 MHz, DMSO-d_6, 298 K) of compound **3**.

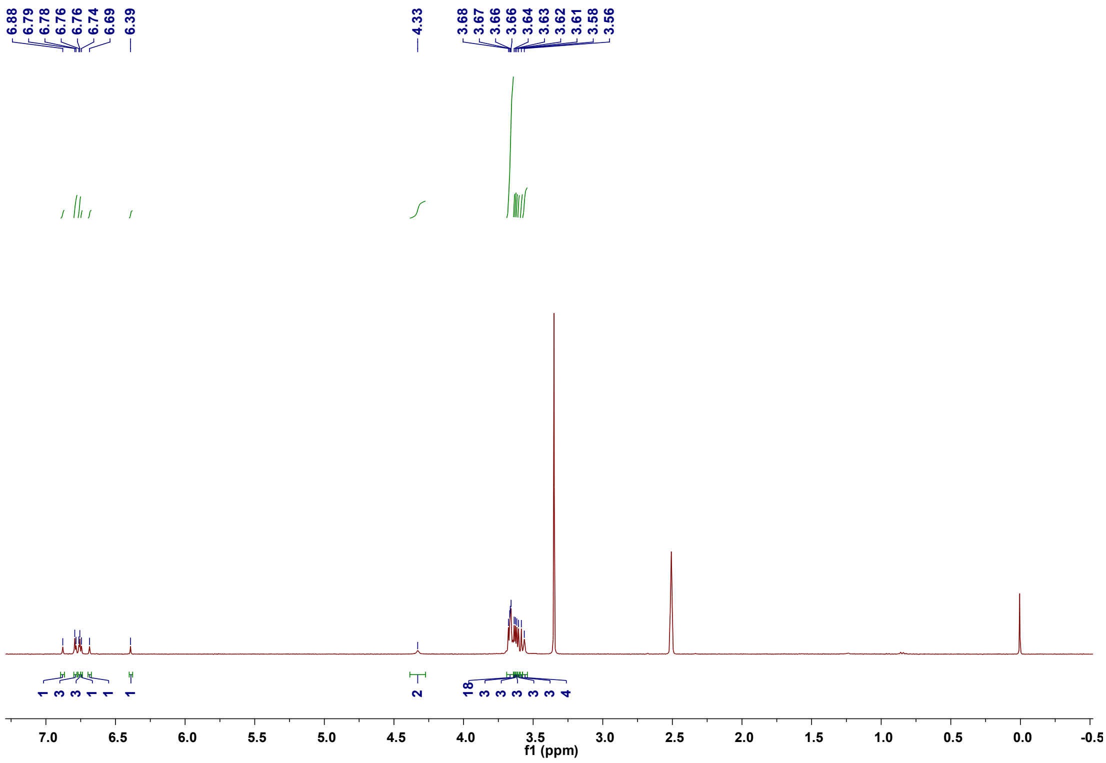

^{1}H NMR spectrum (400 MHz, DMSO-d_6, 298 K) of compound **4**.

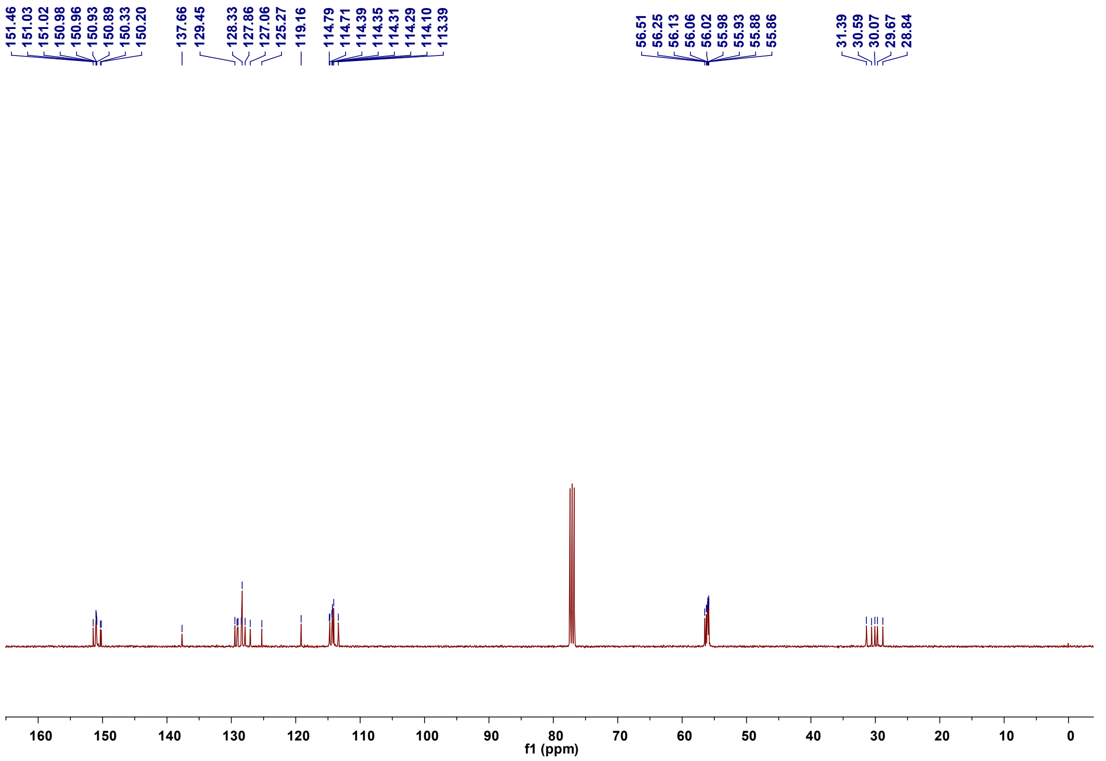

^{13}C NMR spectrum (100 MHz, $CDCl_3$, 298 K) of compound **4**.

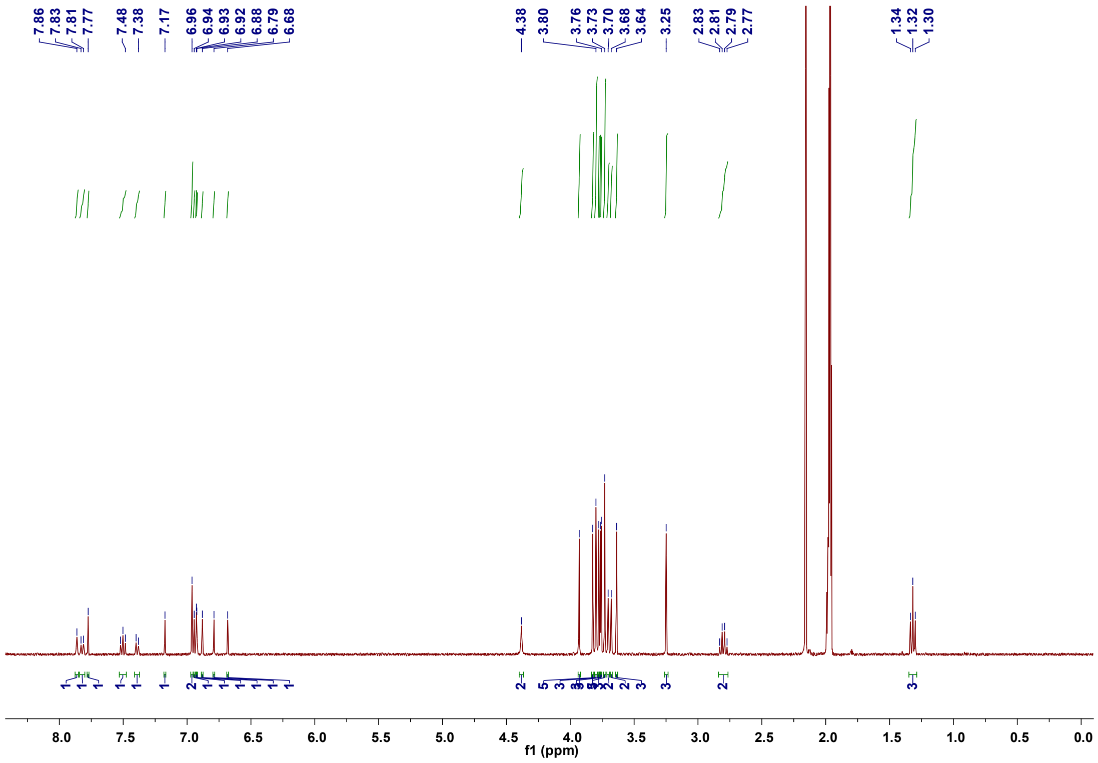

^{1}H NMR spectrum (400 MHz, CD_3CN, 298 K) of compound **AzoP[5]A-1**$_{E}$.

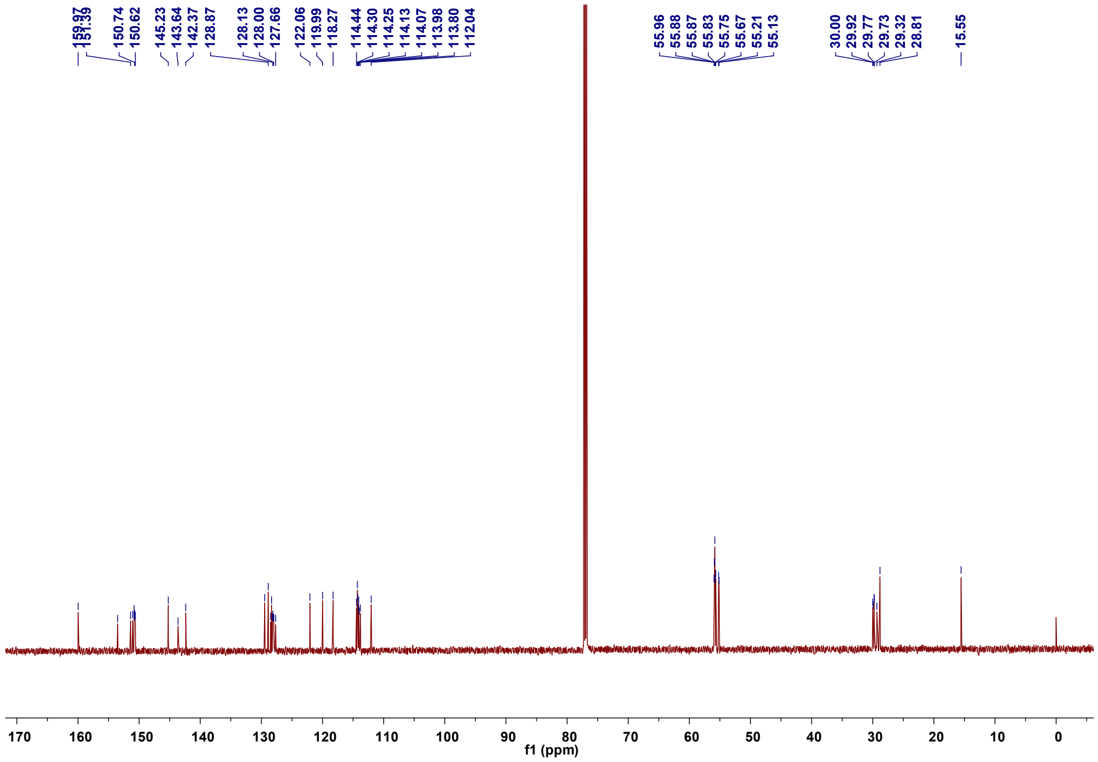

^{13}C NMR spectrum (100 MHz, $CDCl_3$, 298 K) of compound **AzoP[5]A-1**$_{E}$.

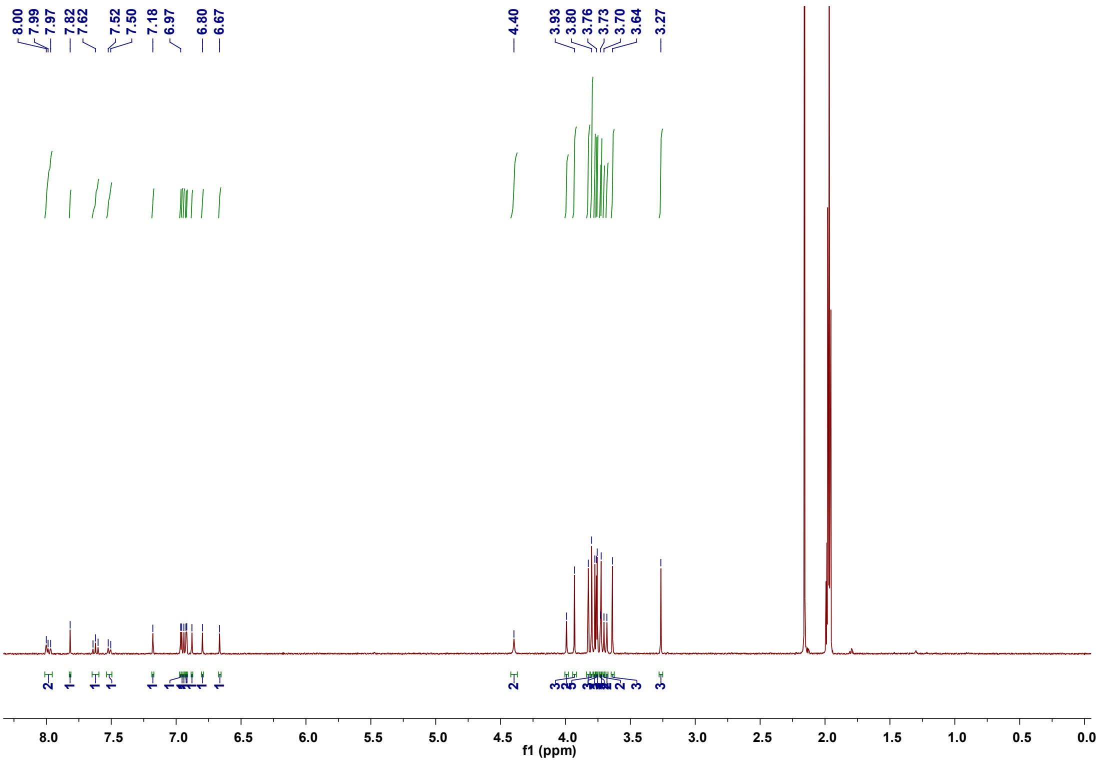

^{1}H NMR spectrum (400 MHz, CD_3CN, 298 K) of compound **AzoP[5]A-2**$_{E}$.

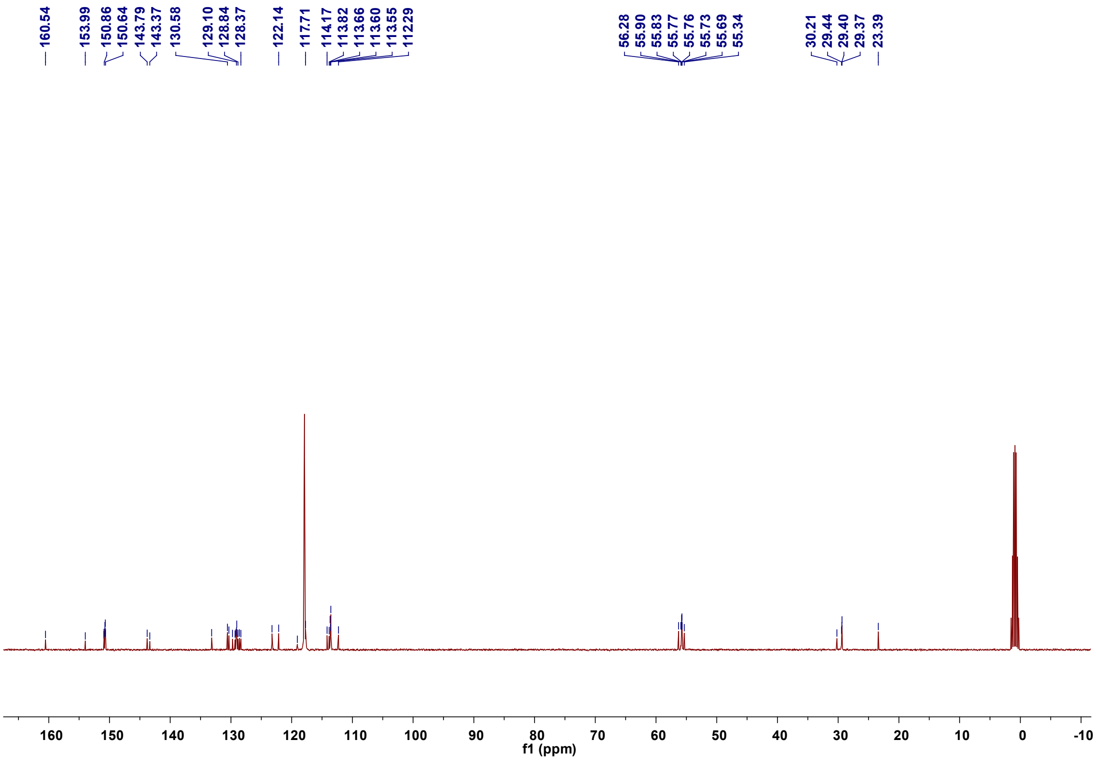

^{13}C NMR spectrum (100 MHz, CD_3CN, 298 K) of compound **AzoP5A-2**$_{E}$.

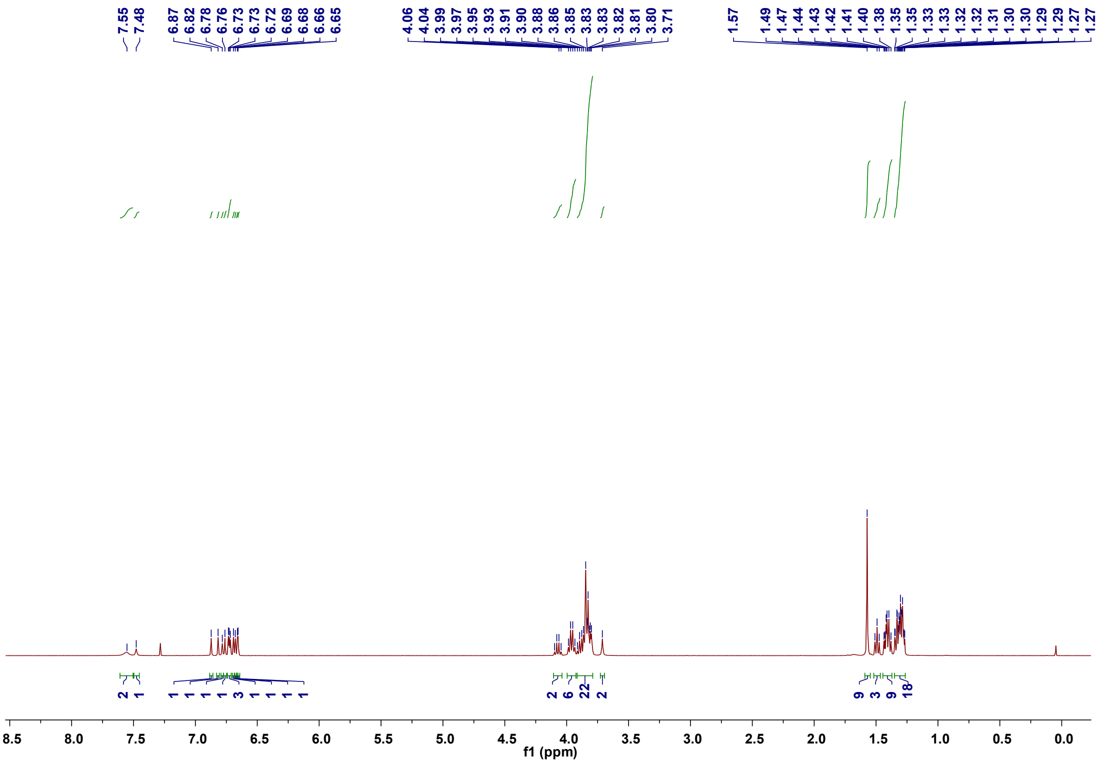

^{1}H NMR spectrum (400 MHz, $CDCl_3$, 298 K) of compound **9**.

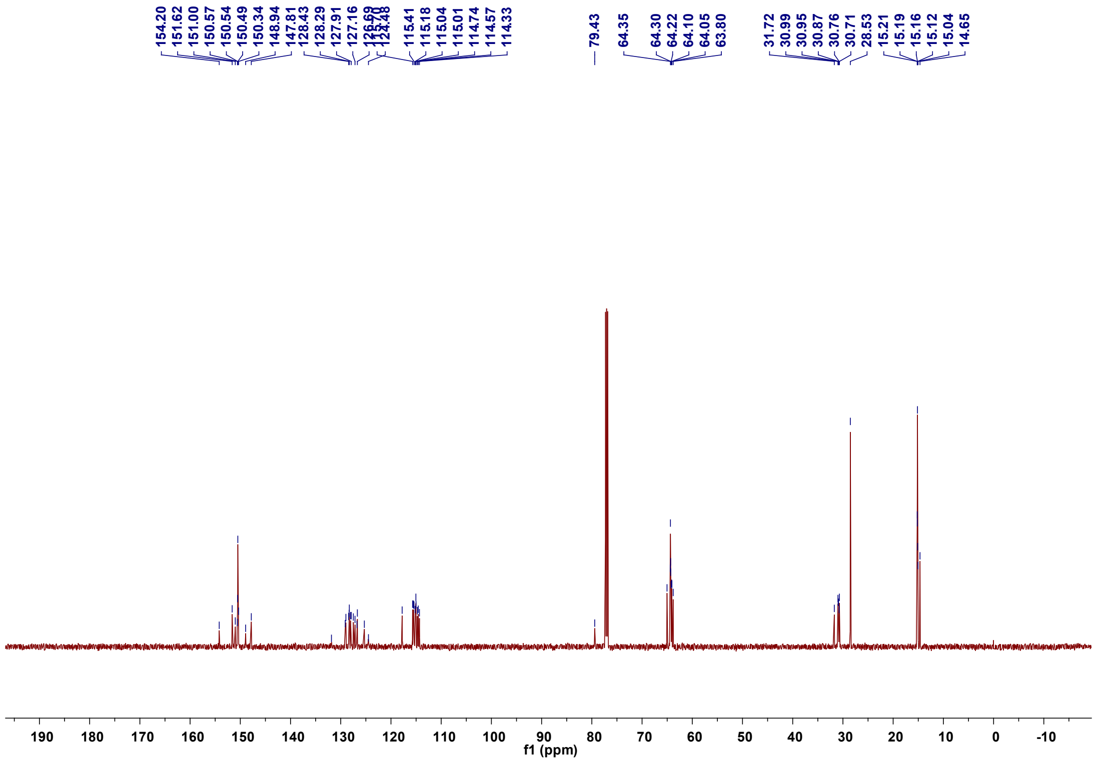

^{13}C NMR spectrum (150 MHz, $CDCl_3$, 298 K) of compound **9**.

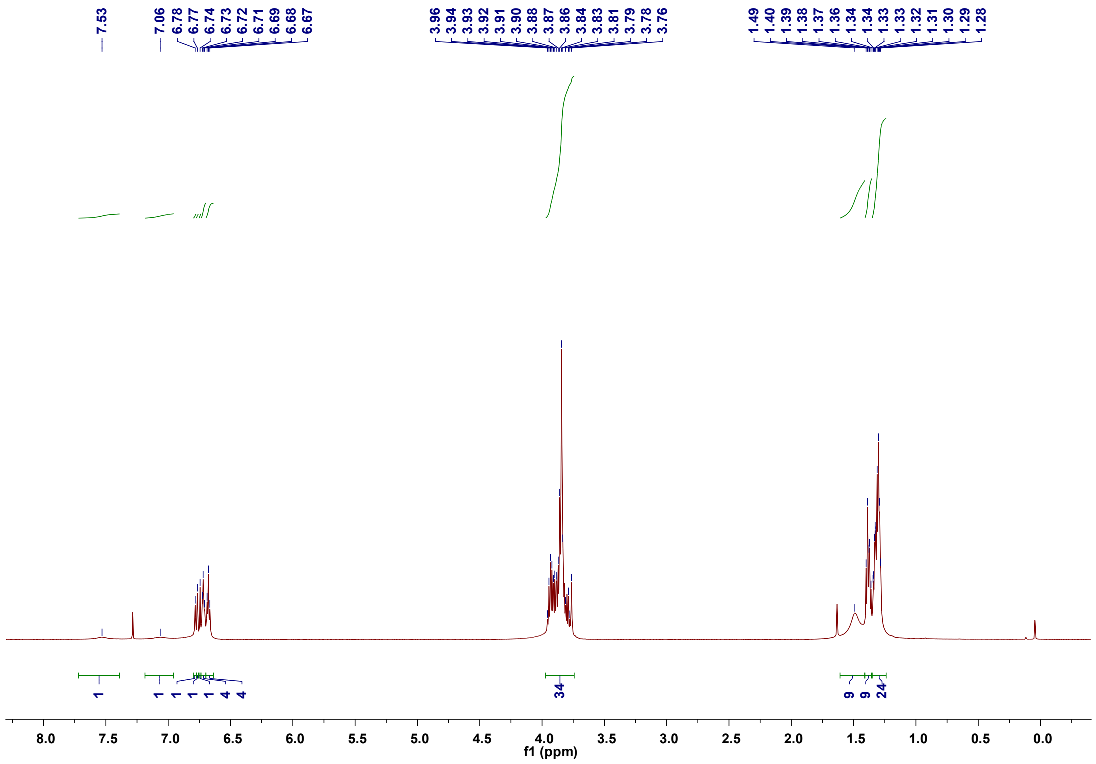

^{1}H NMR spectrum (600 MHz, $CDCl_3$, 298 K) of compound **10**.

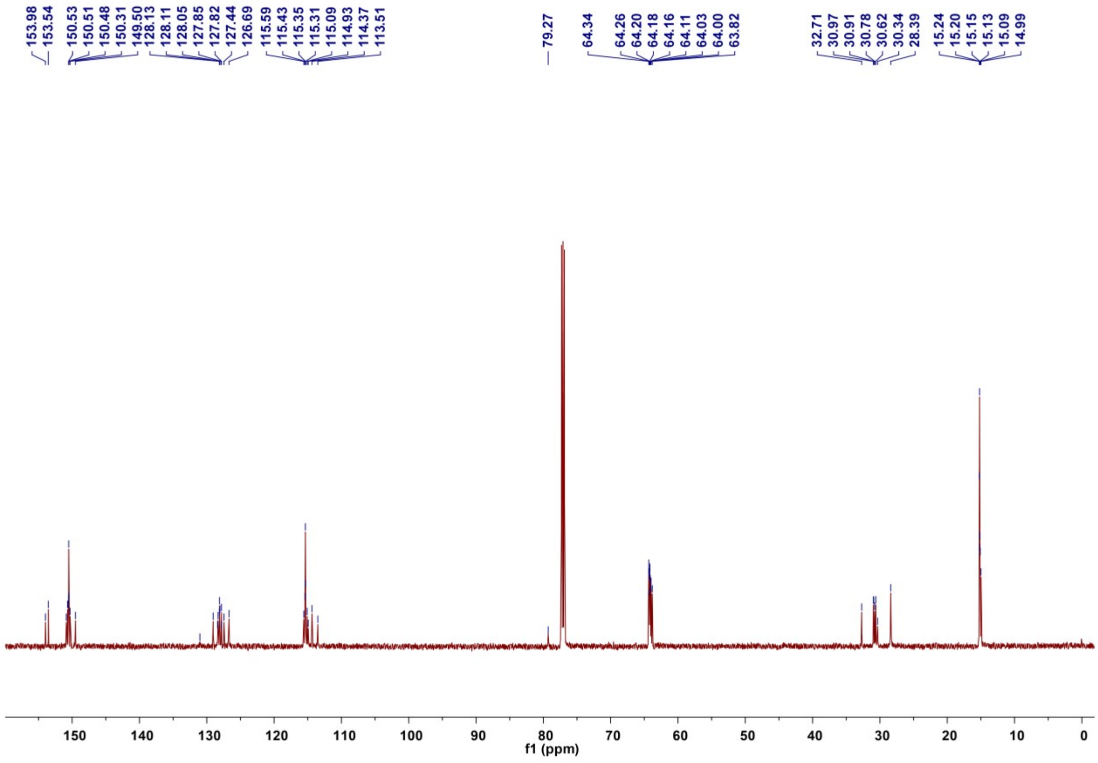

^{13}C NMR spectrum (150 MHz, $CDCl_3$, 298 K) of compound **10**.

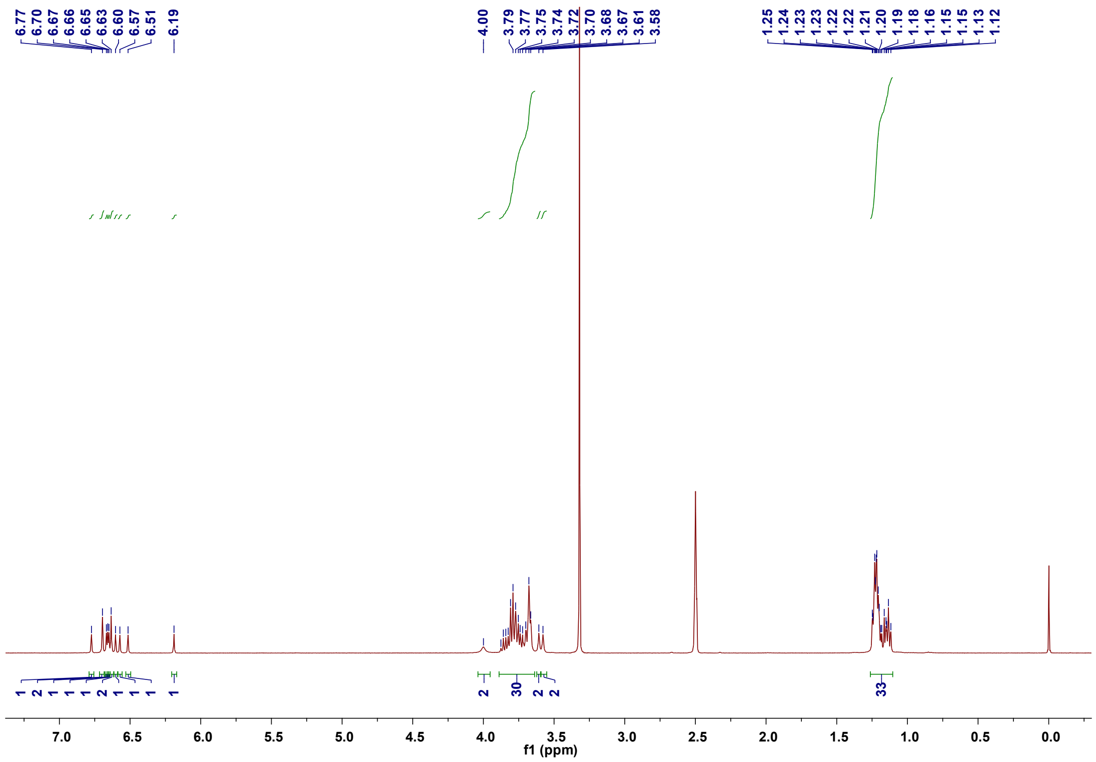

^{1}H NMR spectrum (400 MHz, DMSO-d_6, 298 K) of compound **11**.

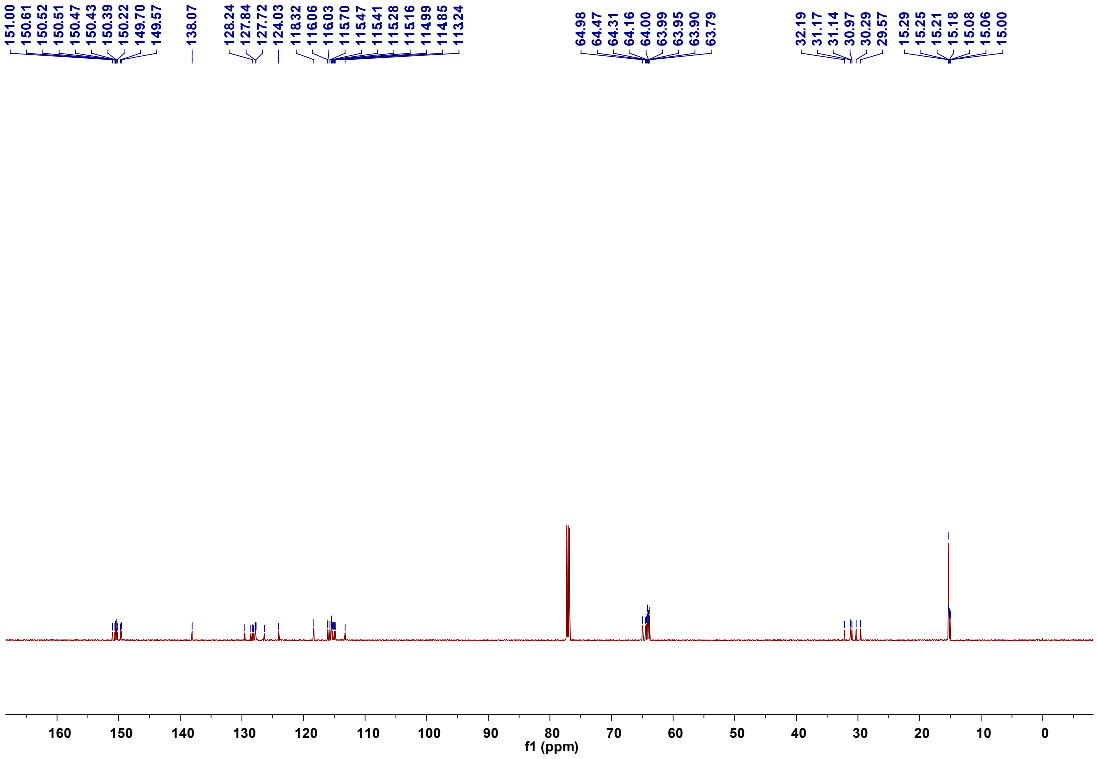

^{13}C NMR spectrum (150 MHz, $CDCl_3$, 298 K) of compound **11**.

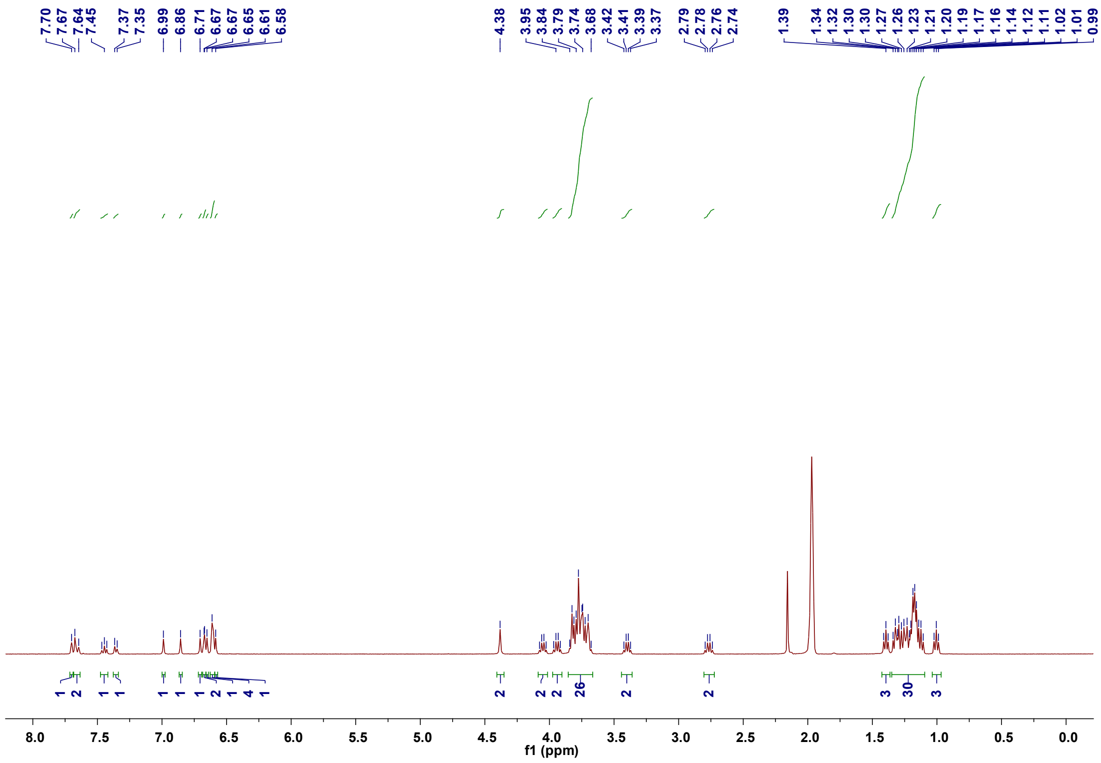

^{1}H NMR spectrum (400 MHz, CD_3CN, 298 K) of compound **AzoP[6]A-1**$_E$.

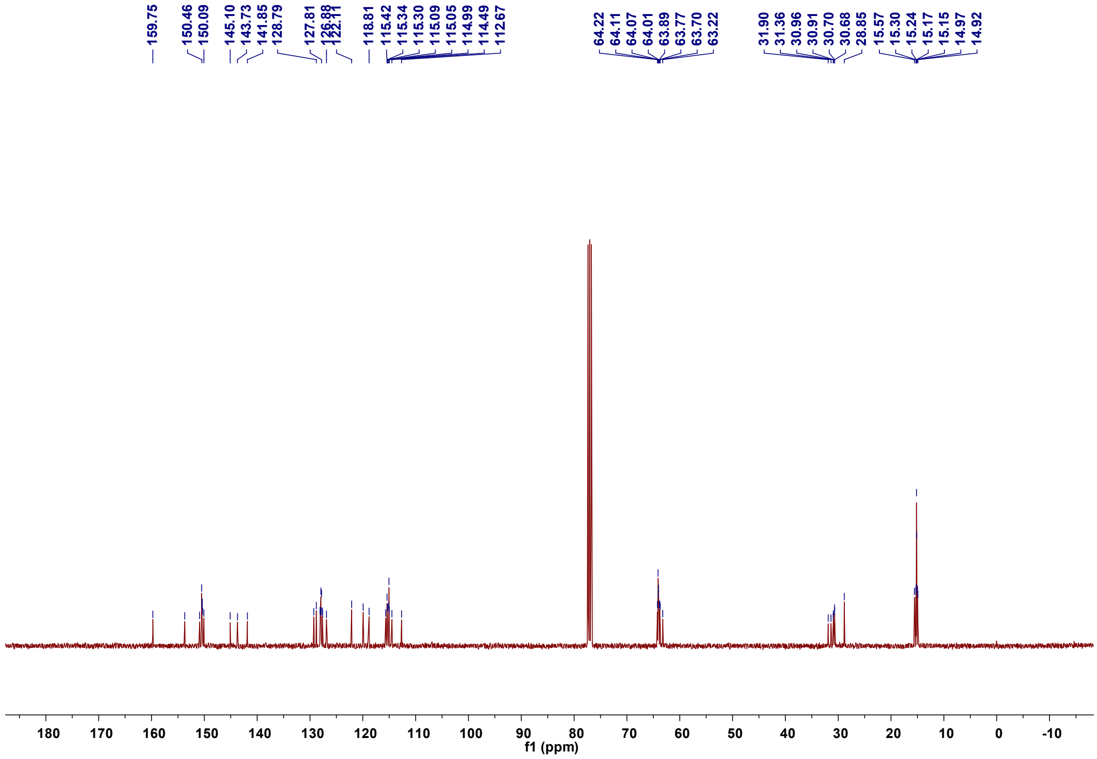

^{13}C NMR spectrum (100 MHz, $CDCl_3$, 298 K) of compound **AzoP[6]A-1**$_E$.

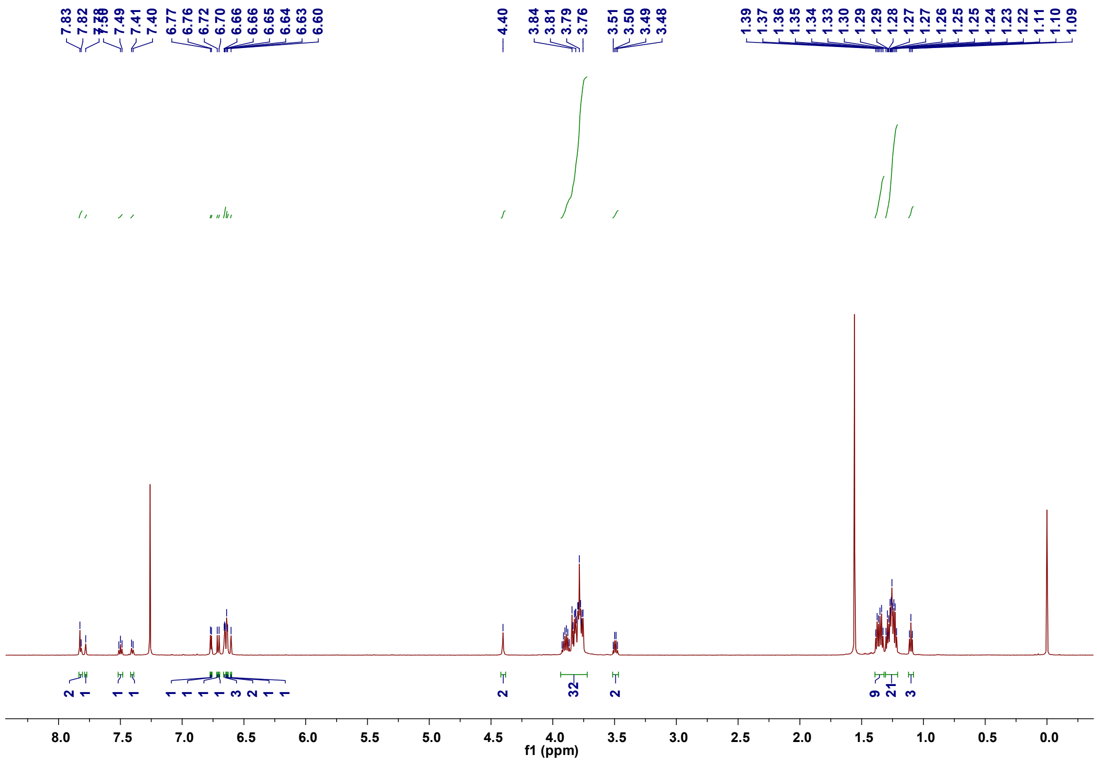

^{1}H NMR spectrum (600 MHz, $CDCl_3$, 298 K) of compound **AzoP[6]A-2**$_E$.

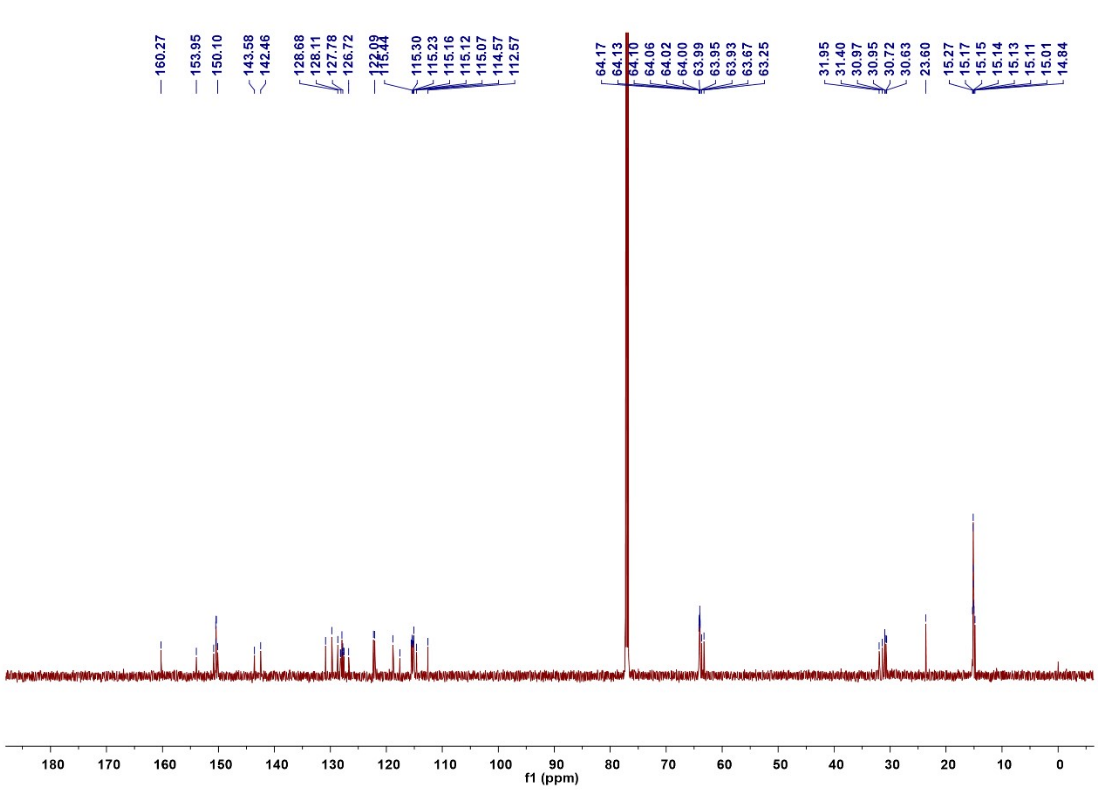

^{13}C NMR spectrum (150 MHz, $CDCl_3$, 298 K) of compound **AzoP[6]A-2**$_E$.

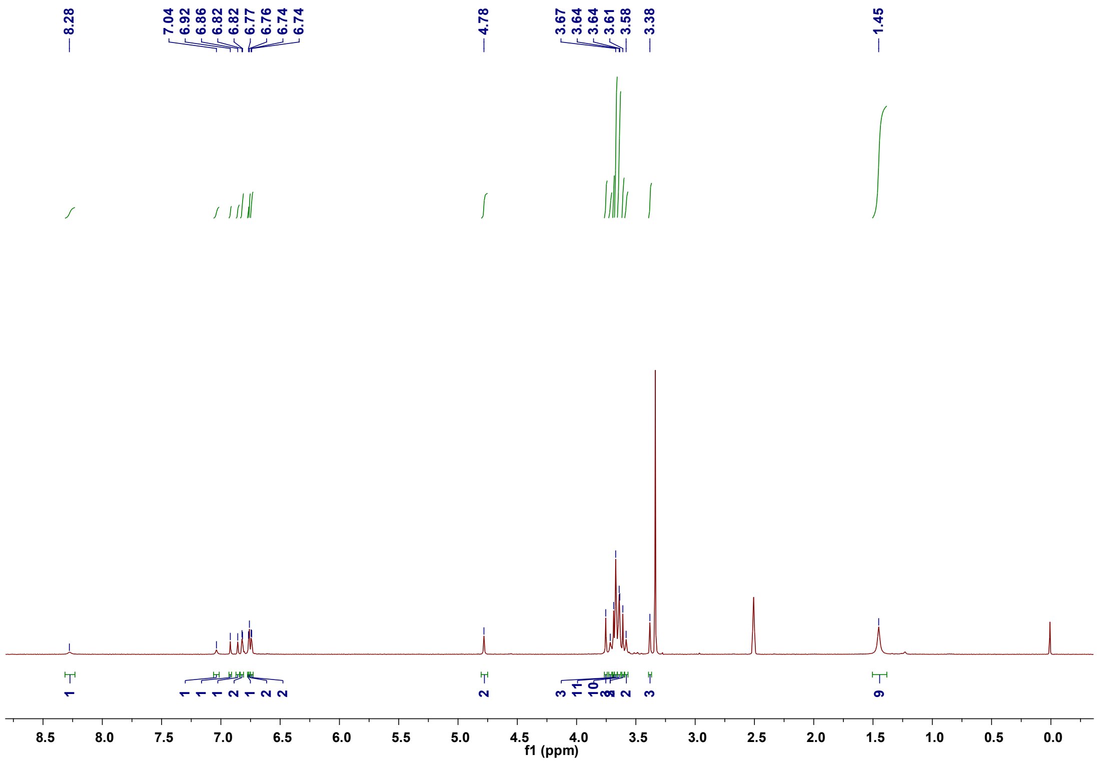

^{1}H NMR spectrum (400 MHz, DMSO-d_6, 298 K) of compound **13**.

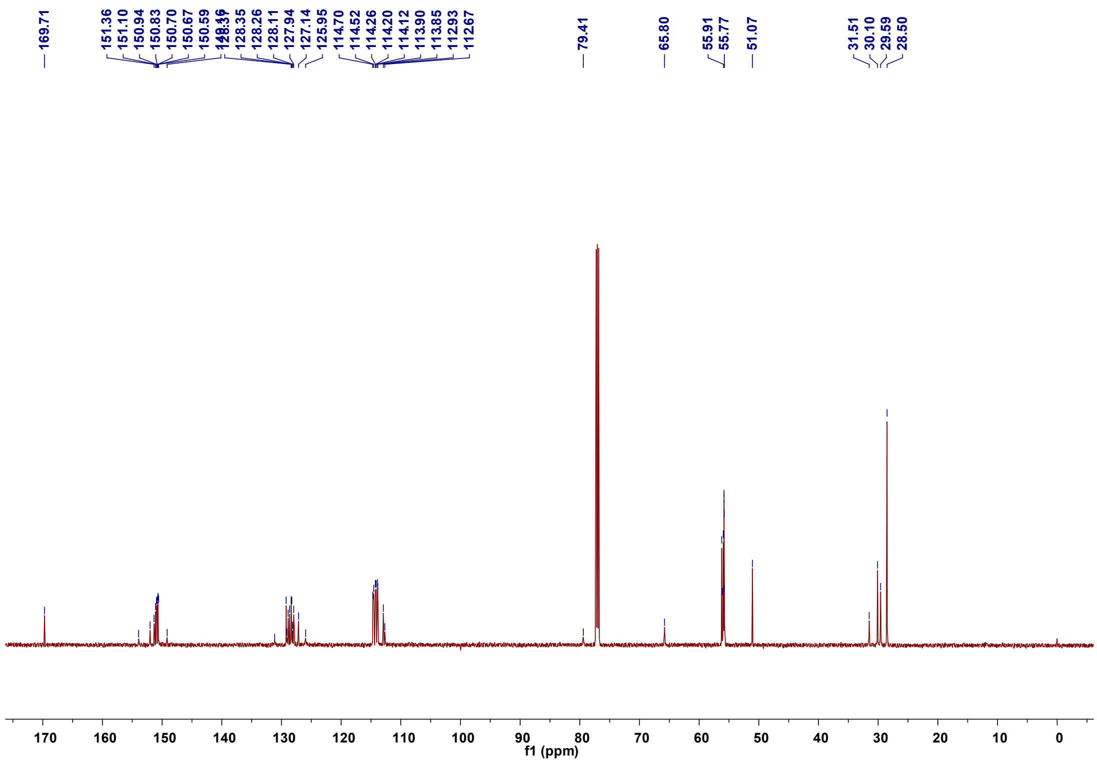

^{13}C NMR spectrum (150 MHz, $CDCl_3$, 298 K) of compound **13**.

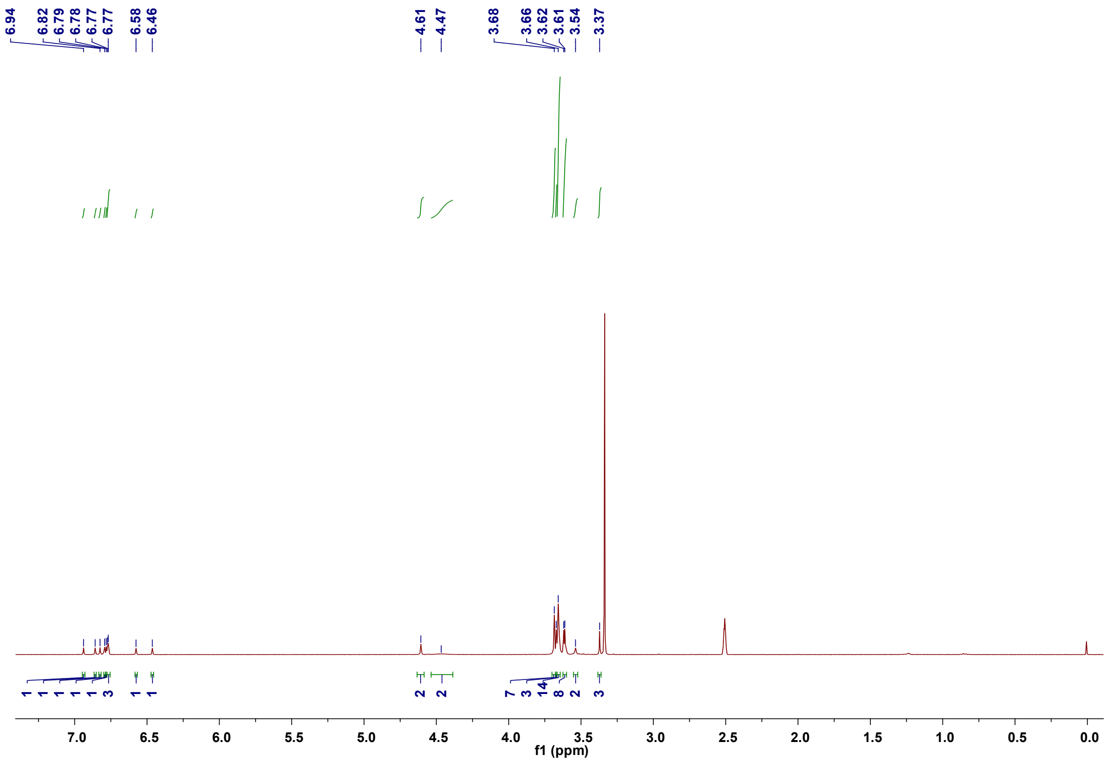

^{1}H NMR spectrum (400 MHz, DMSO-d_6, 298 K) of compound **14**.

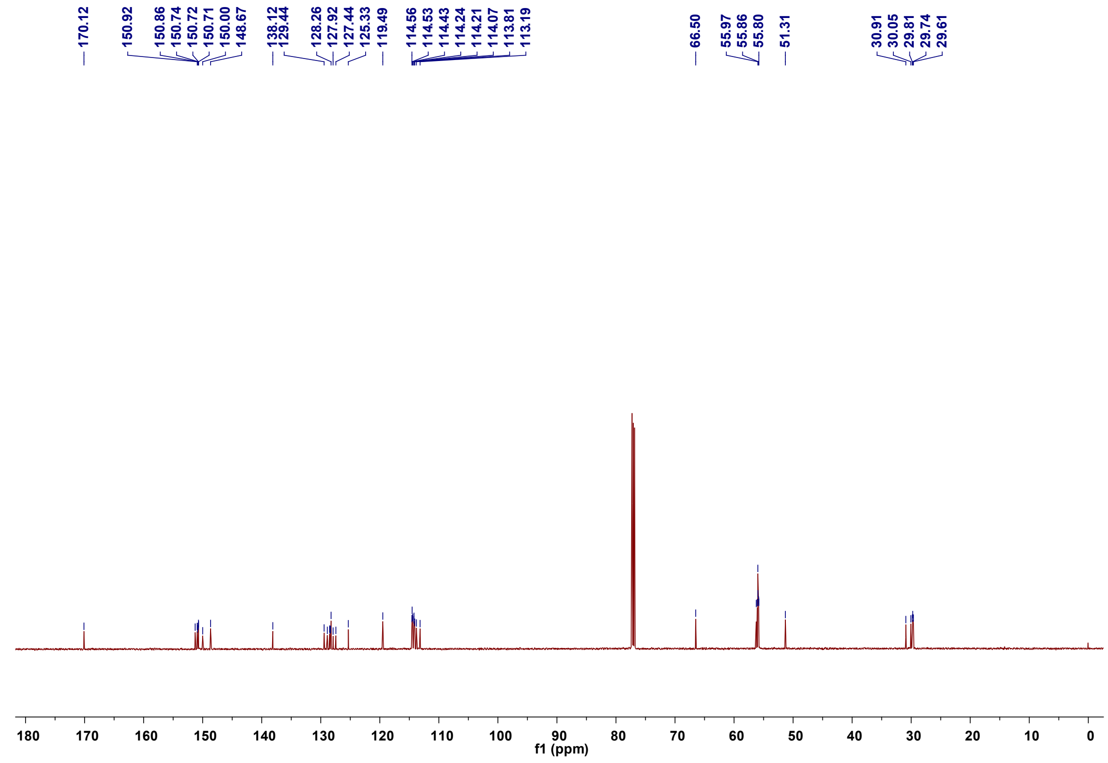

^{13}C NMR spectrum (150 MHz, $CDCl_3$, 298 K) of compound **14**.

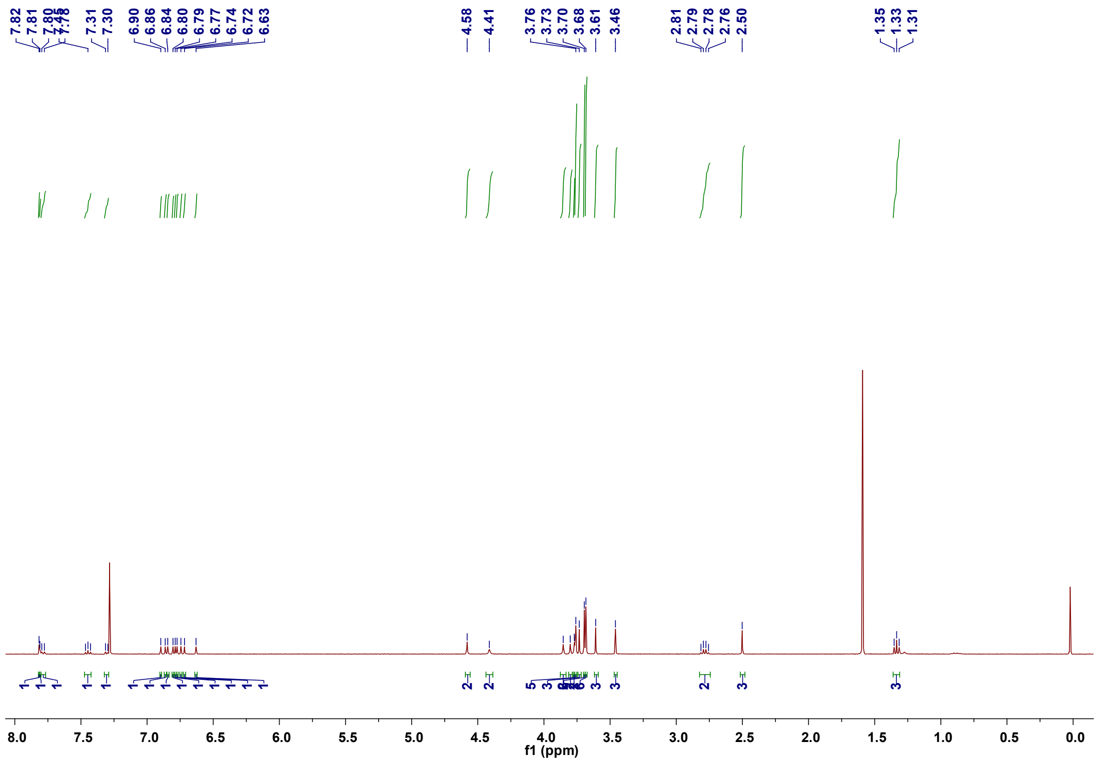

^{1}H NMR spectrum (400 MHz, $CDCl_3$, 298 K) of compound **15**.

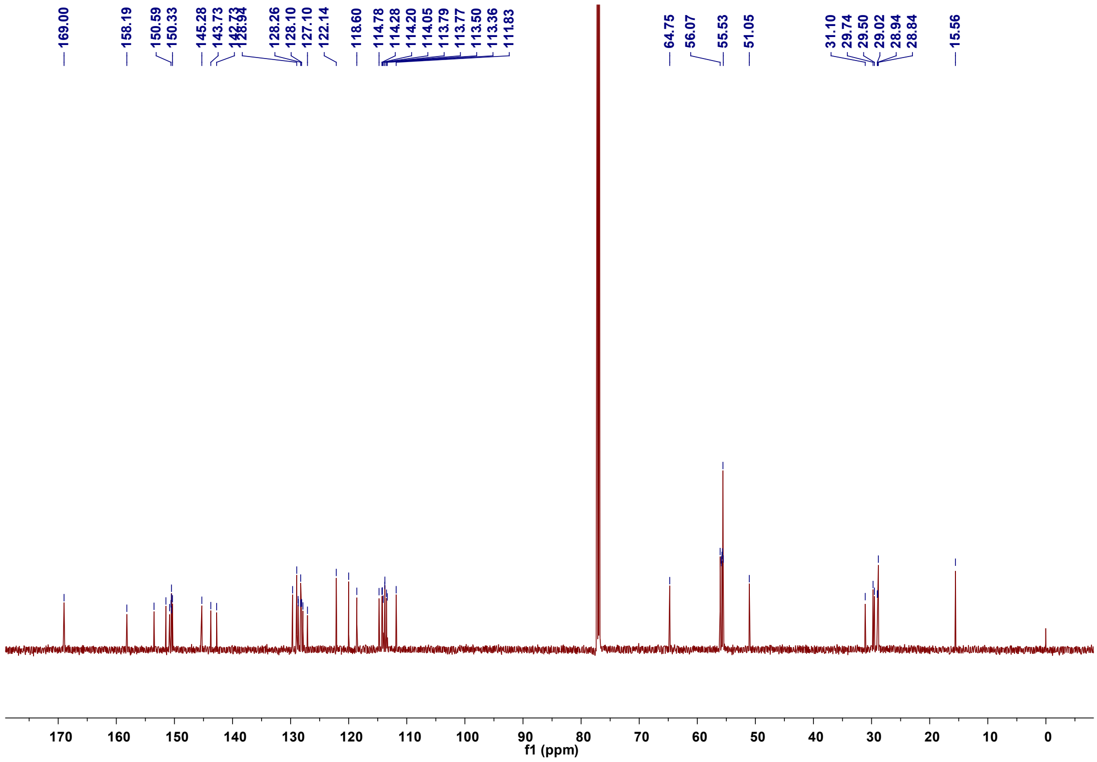

^{13}C NMR spectrum (150 MHz, $CDCl_3$, 298 K) of compound **15**.

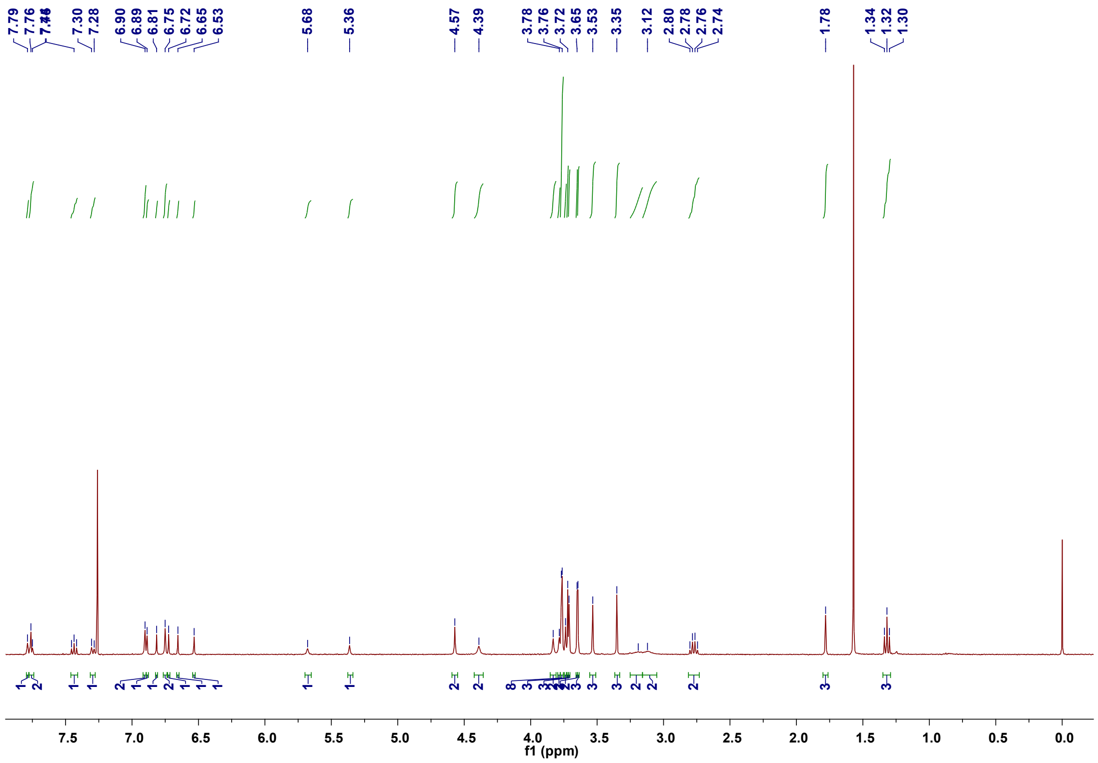

^{1}H NMR spectrum (600 MHz, $CDCl_3$, 298 K) of compound **18**.

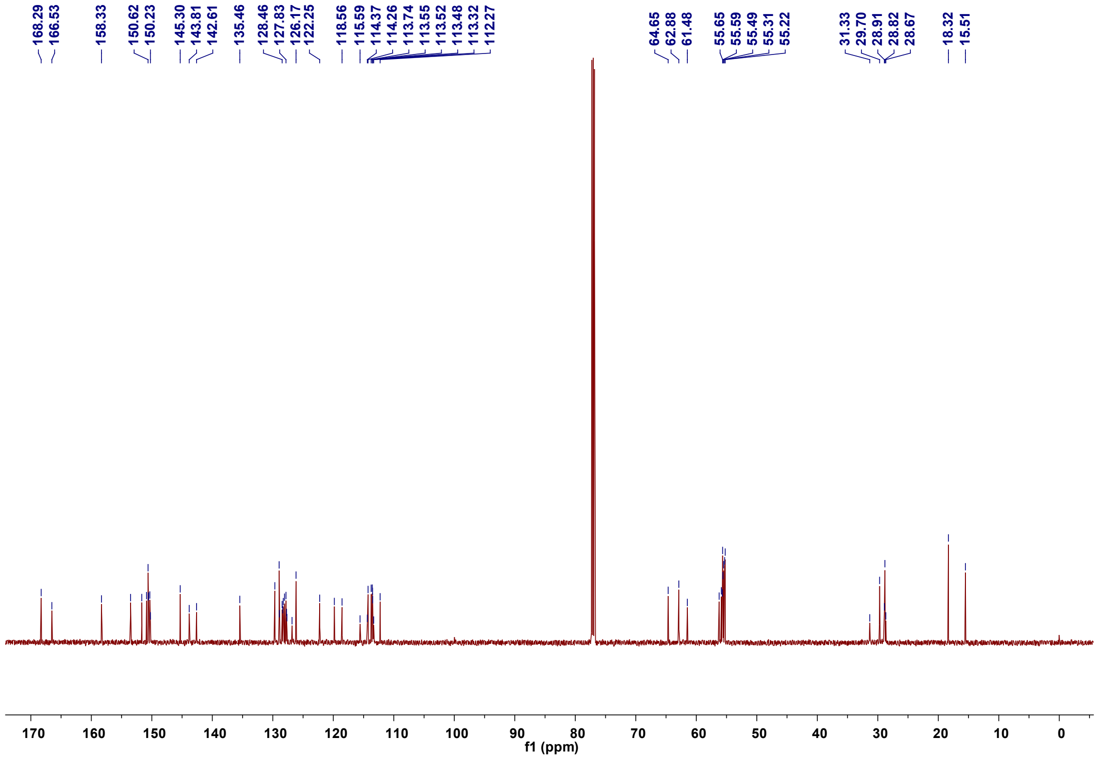

^{13}C NMR spectrum (150 MHz, $CDCl_3$, 298 K) of compound **18**.

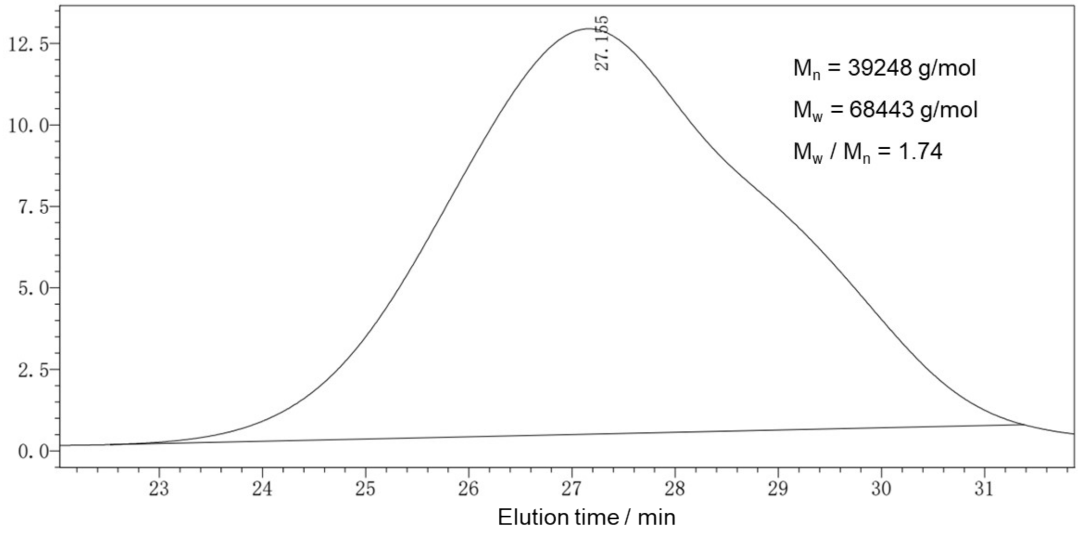

GPC trace of the polymer *E*-**P1.**

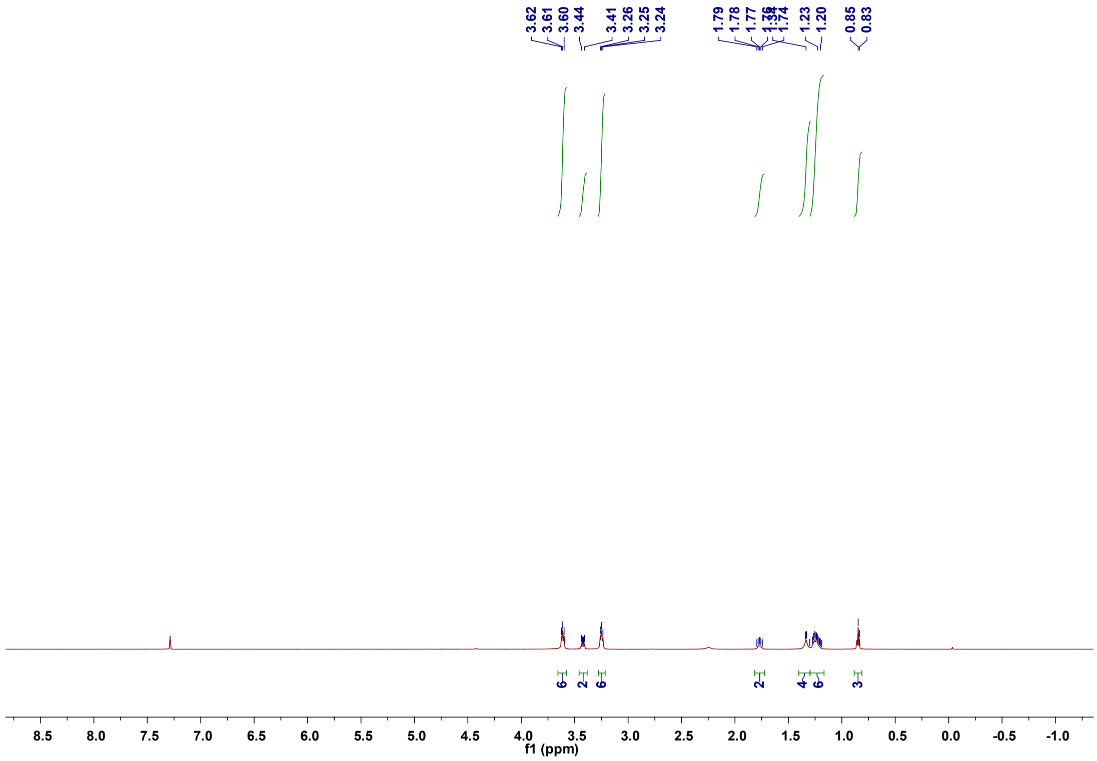

^{1}H NMR spectrum (600 MHz, $CDCl_3$, 298 K) of compound **G6**.

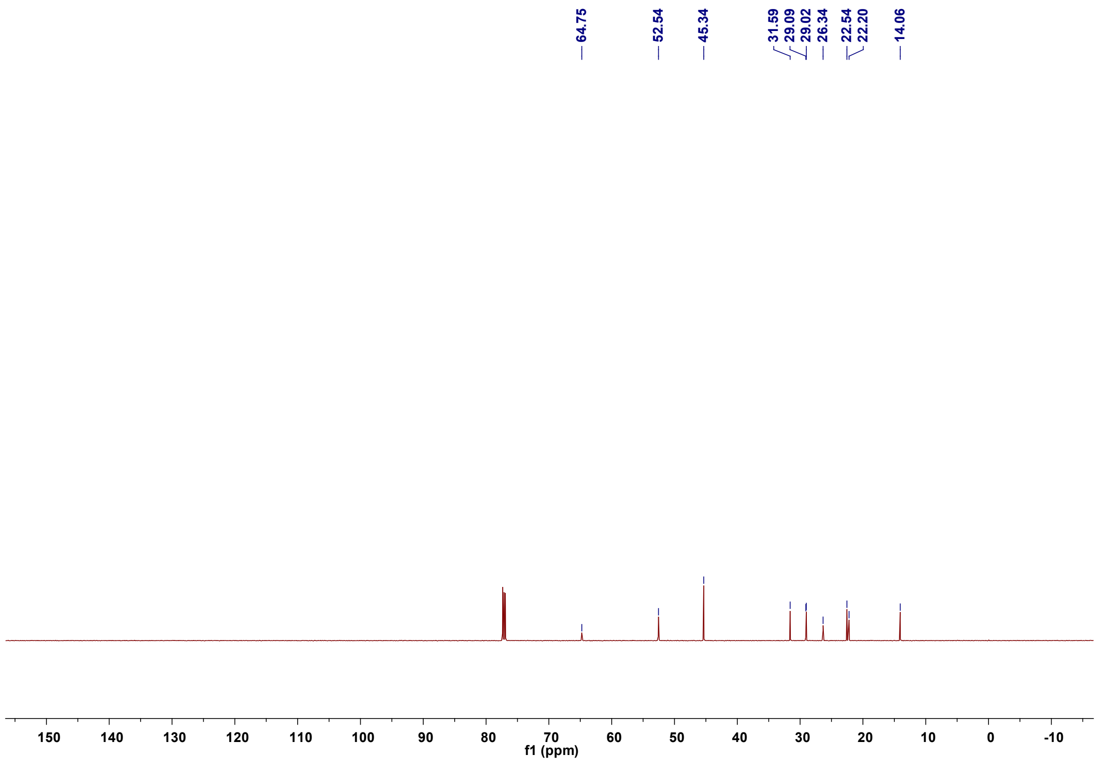

^{13}C NMR spectrum (150 MHz, $CDCl_3$, 298 K) of compound **G6**.

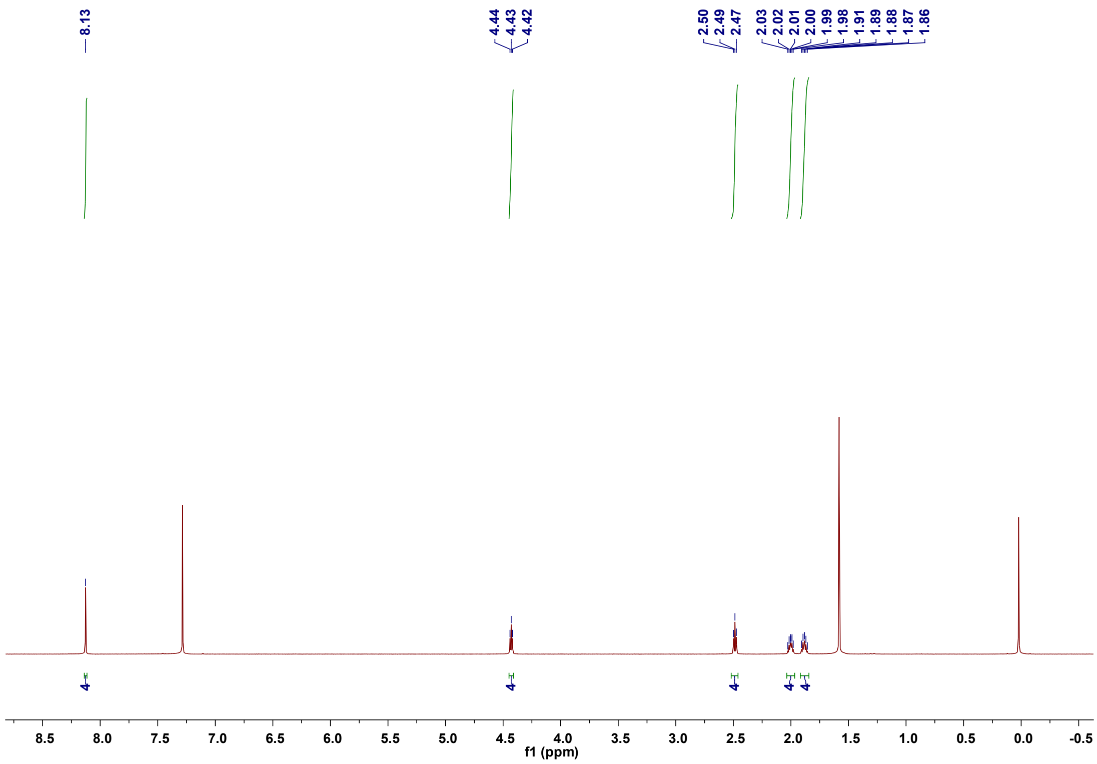

^{1}H NMR spectrum (600 MHz, $CDCl_3$, 298 K) of compound **L1**.

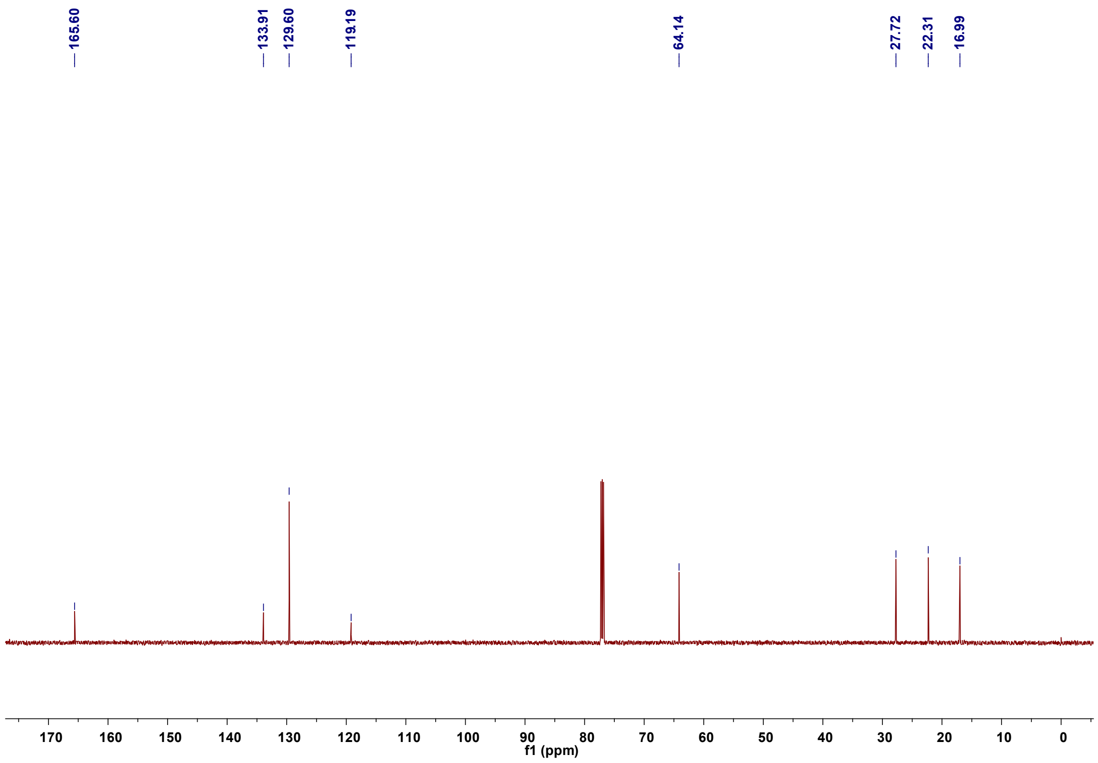

^{13}C NMR spectrum (150 MHz, $CDCl_3$, 298 K) of compound **L1**.

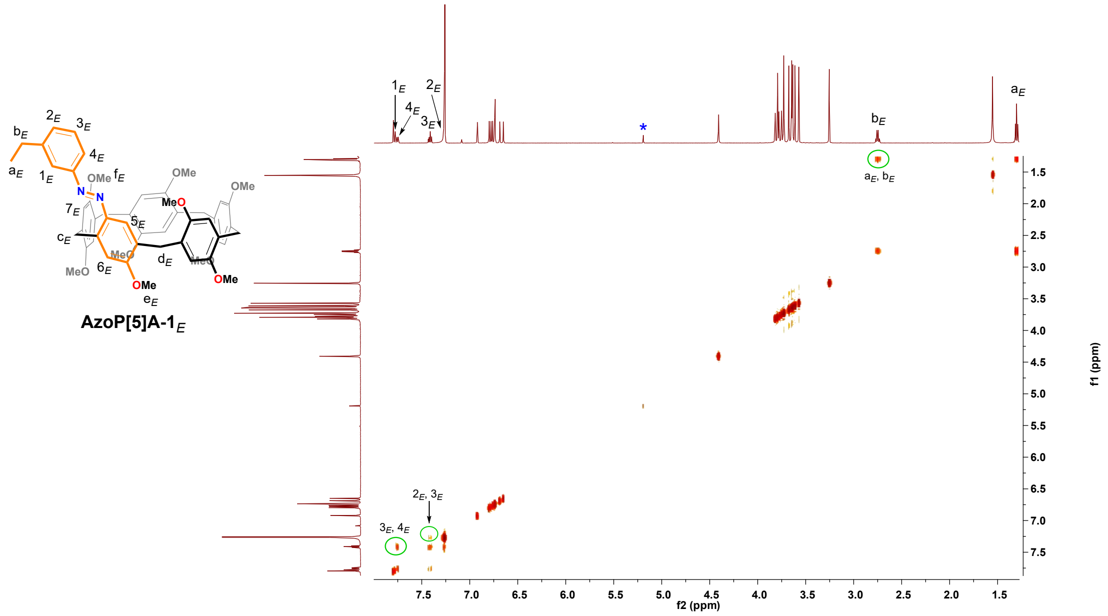

2D COSY-NMR spectrum (600 MHz, 4.0 mM, $CDCl_3$, 298K) for the solution of **AzoP[5]A-1**$_E$. The blue star marked signal refers to CH_2Cl_2.

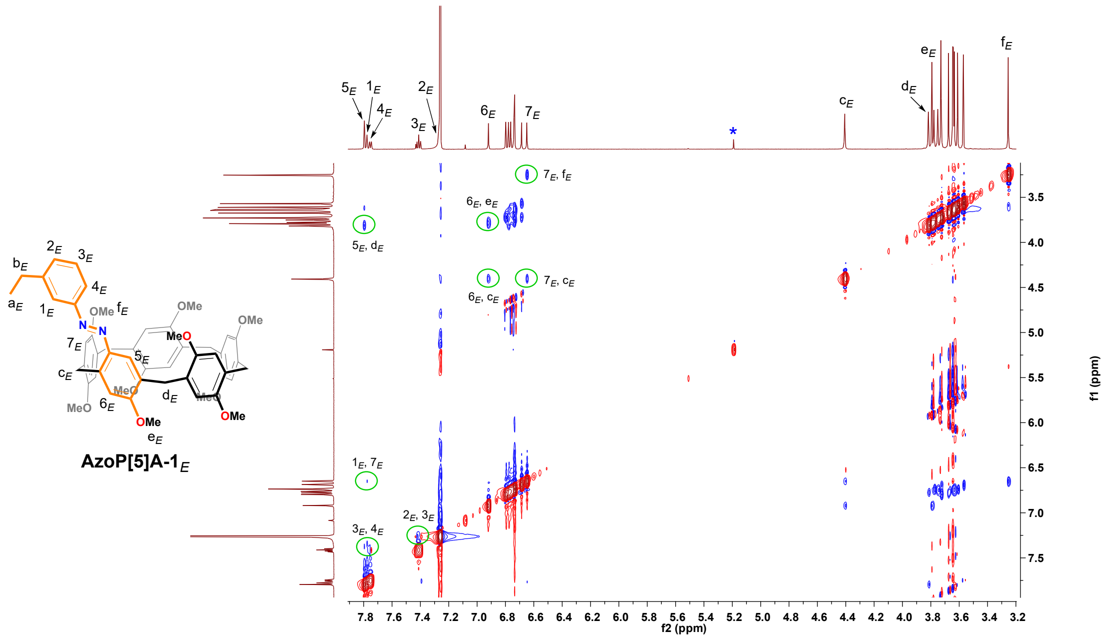

Partial 2D NOESY-NMR spectrum (600 MHz, 4.0 mM, $CDCl_3$, 298K) for the solution of **AzoP[5]A-1**$_E$. The blue star marked signal refers to CH_2Cl_2.

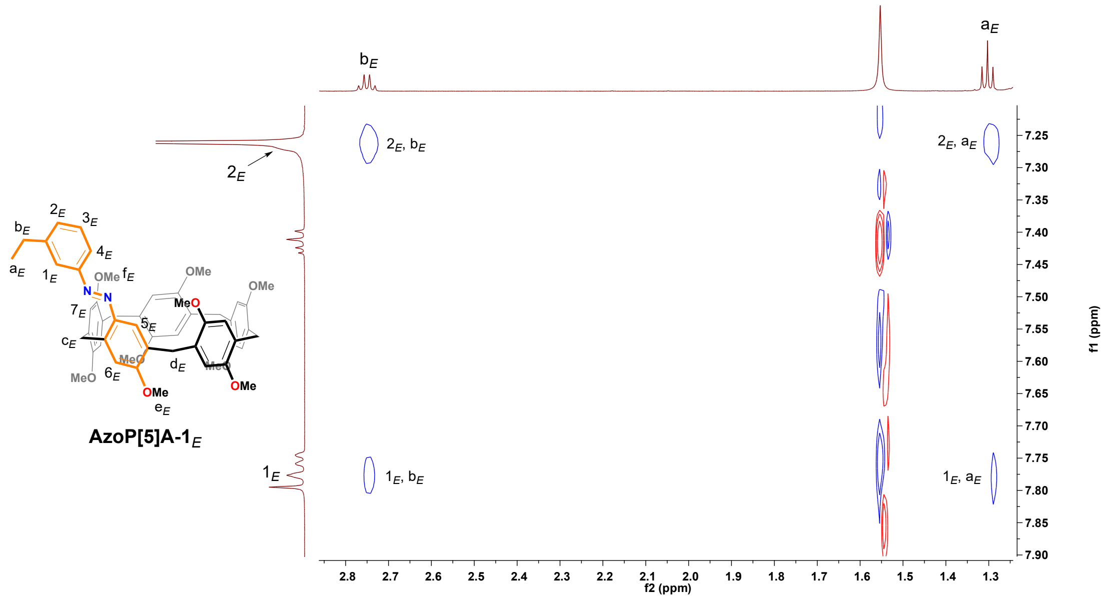

Partial 2D NOESY-NMR spectrum (600 MHz, 4.0 mM, $CDCl_3$, 298K) for the solution of **AzoP[5]A-1**$_E$.

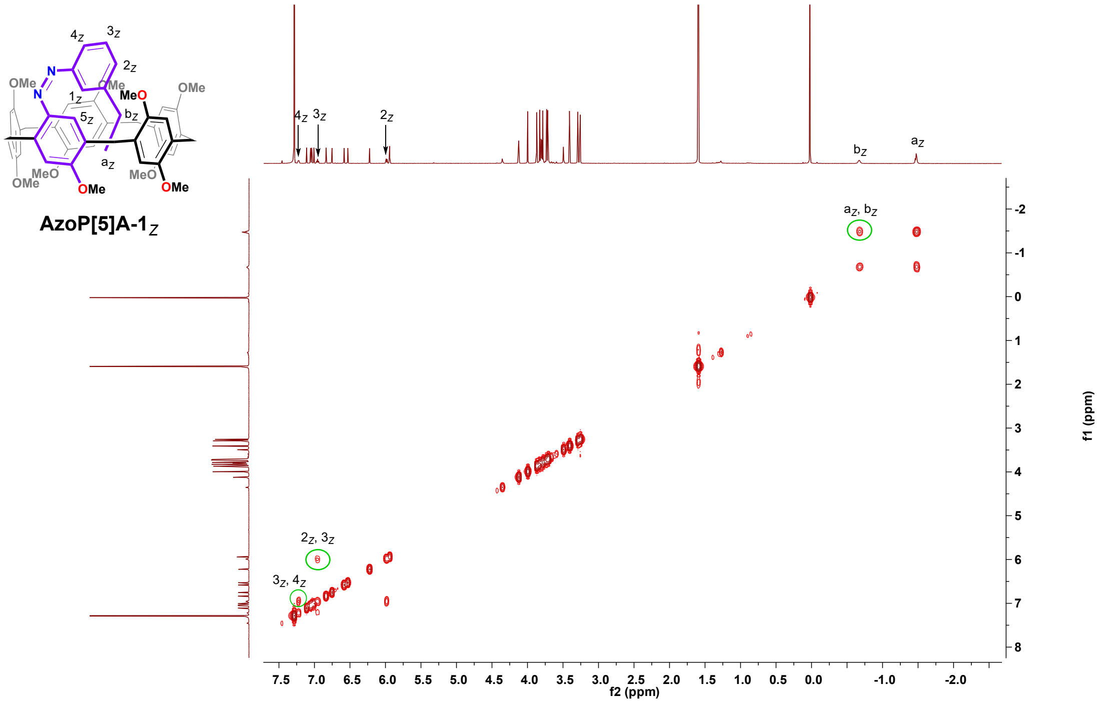

2D COSY-NMR spectrum (600 MHz, 4.0 mM, $CDCl_3$, 298K) for the 390 nm UV light irradiated solution of **AzoP[5]A-1**.

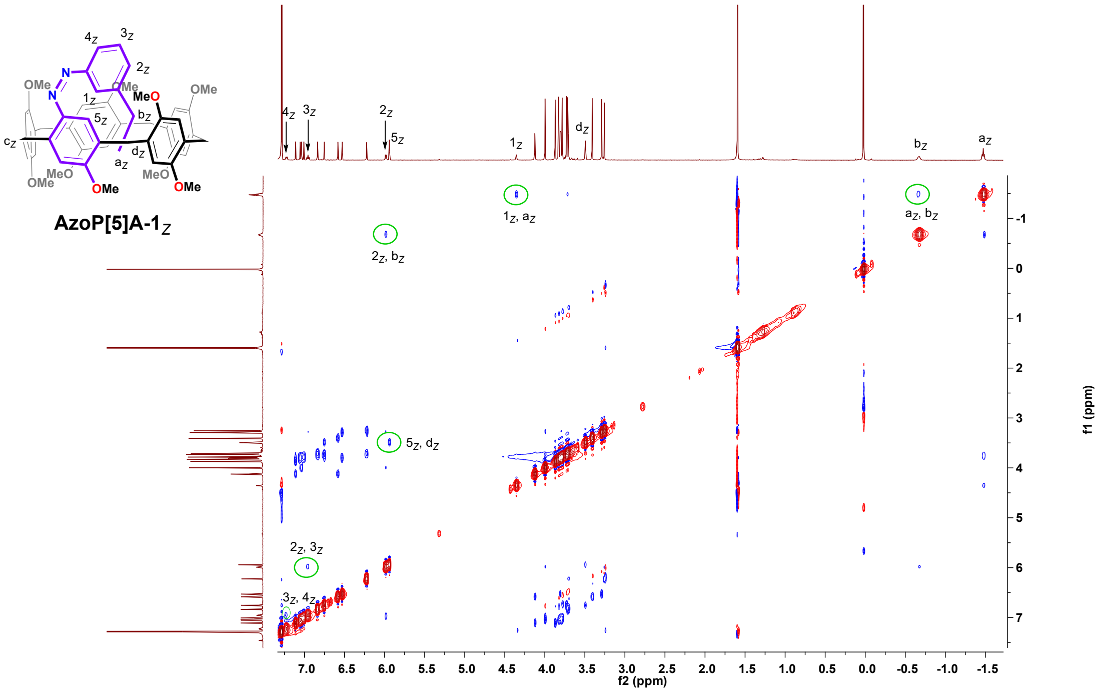

2D NOESY-NMR spectrum (600 MHz, 4.0 mM, $CDCl_3$, 298K) for the 390 nm UV light irradiated solution of **AzoP[5]A-1**.

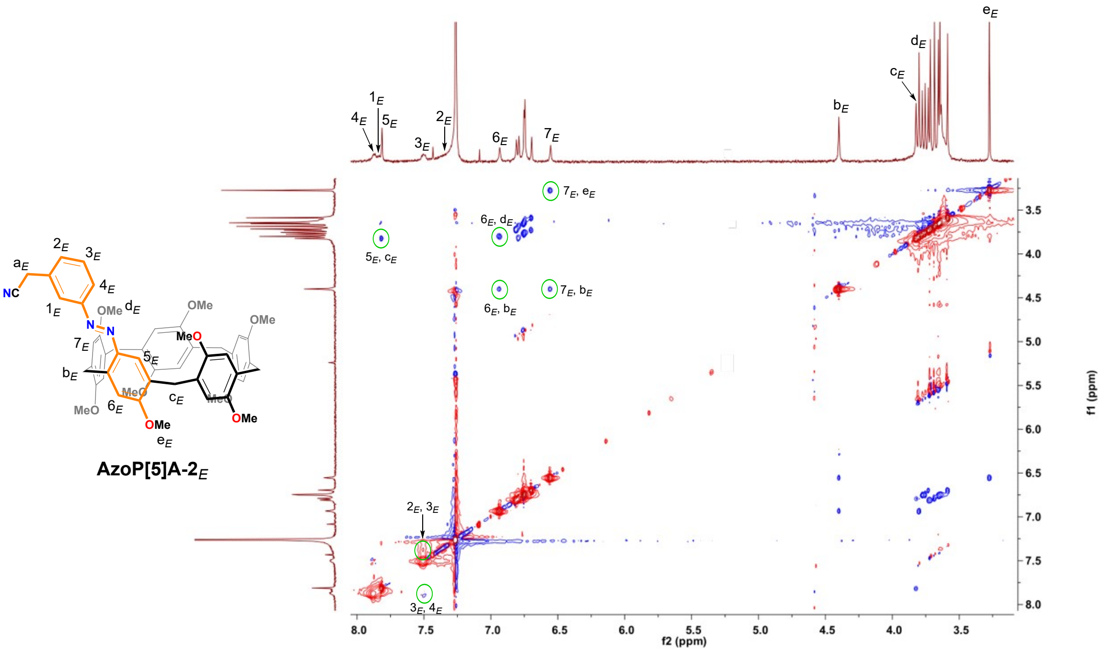

2D NOESY-NMR spectrum (600 MHz, 2.0 mM, $CDCl_3$, 298K) for the solution of **AzoP[5]A-2**$_E$.

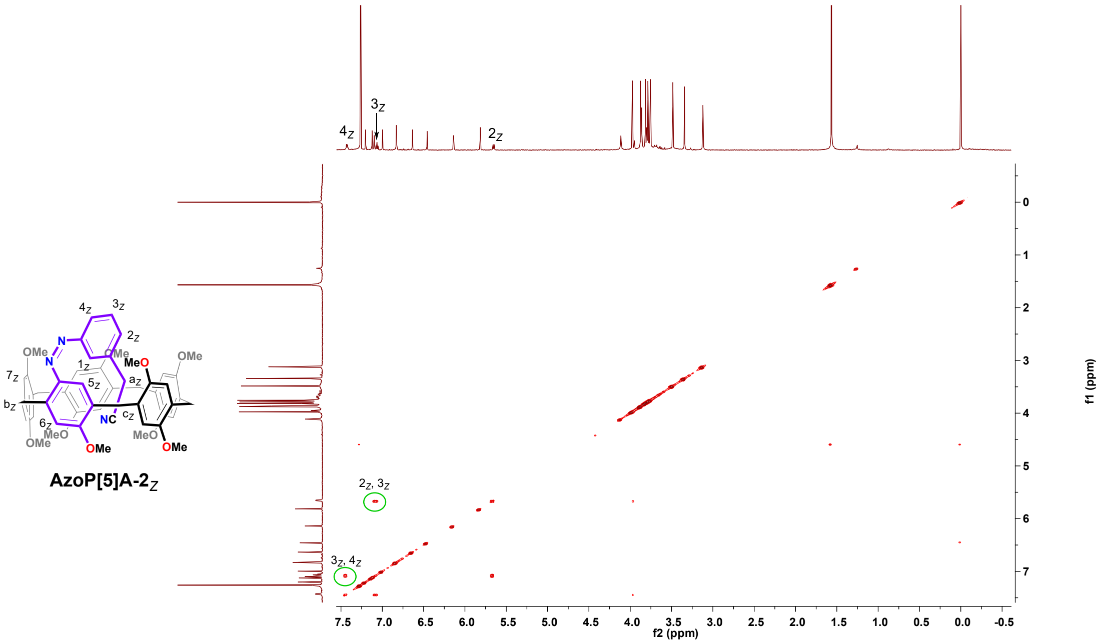

2D COSY-NMR spectrum (600 MHz, 4.0 mM, $CDCl_3$, 298K) for the 390 nm UV light irradiated solution of **AzoP[5]A-2**.

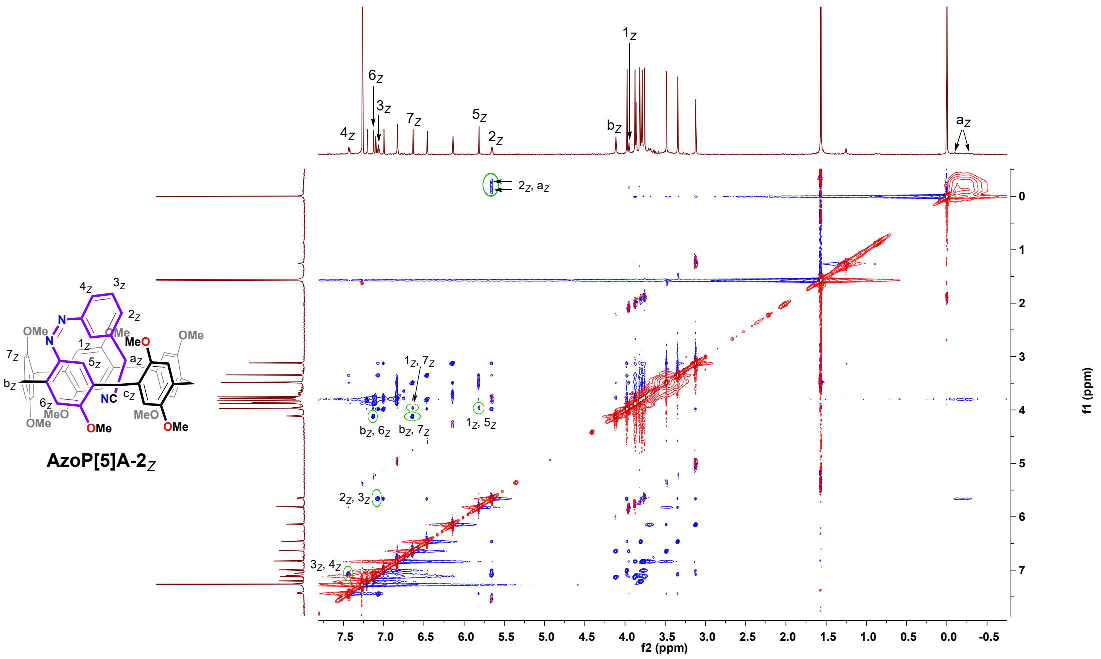

2D NOESY-NMR spectrum (600 MHz, 4.0 mM, $CDCl_3$, 298K) for the 390 nm UV light irradiated solution of **AzoP[5]A-2**.

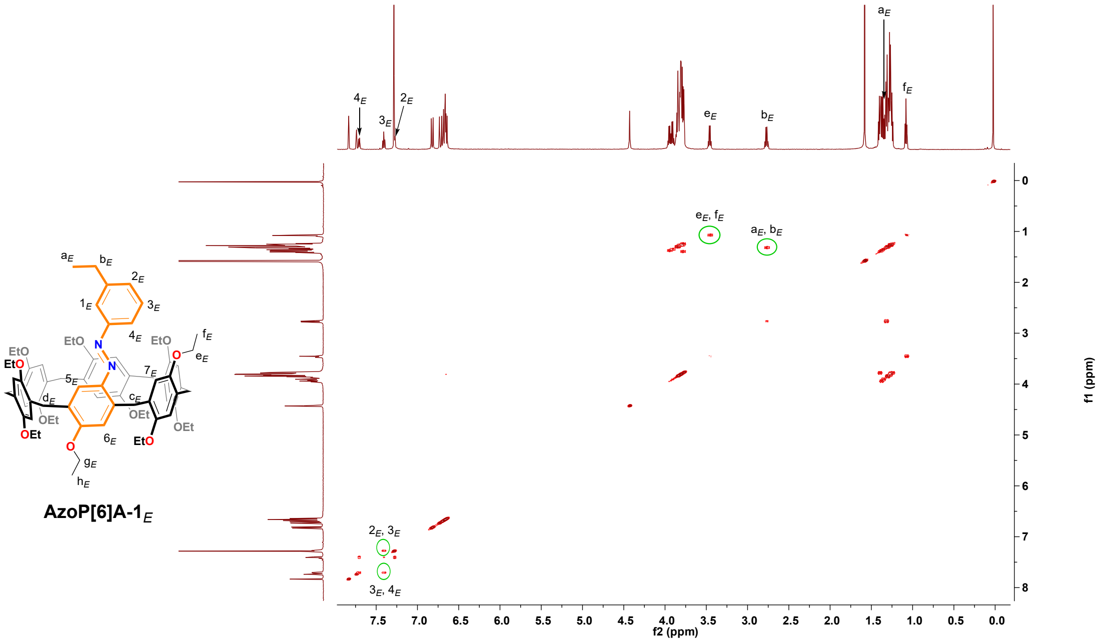

2D COSY-NMR spectrum (600 MHz, 4.0 mM, $CDCl_3$, 298K) for the solution of **AzoP[6]A-1**$_E$.

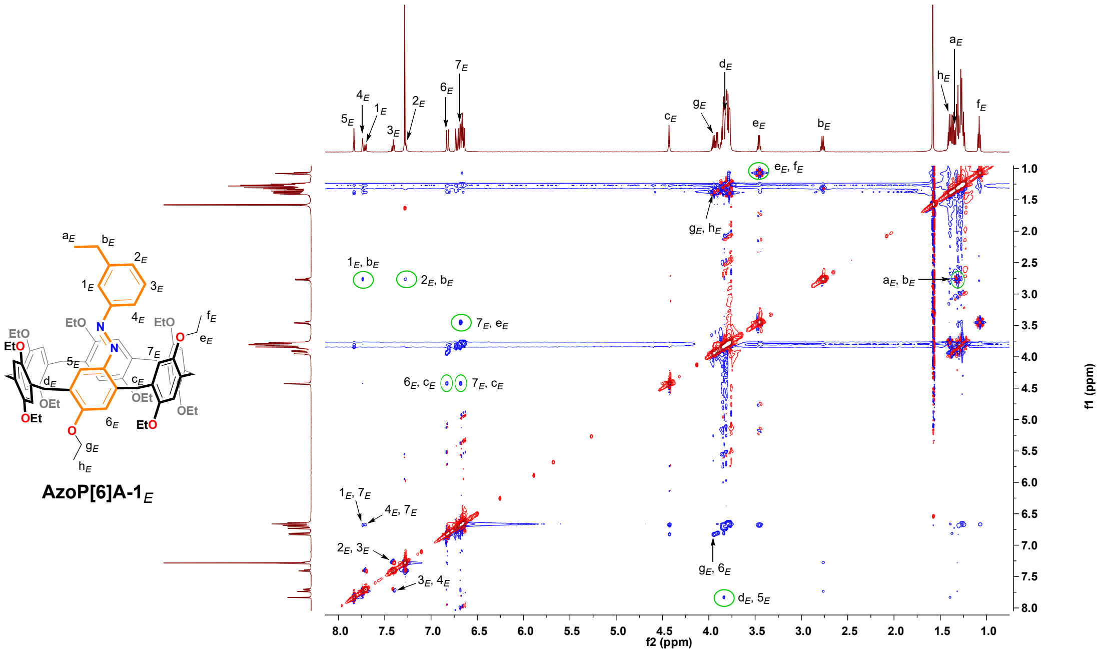

2D NOESY-NMR spectrum (600 MHz, 4.0 mM, $CDCl_3$, 298K) for the solution of **AzoP[6]A-1**$_E$.

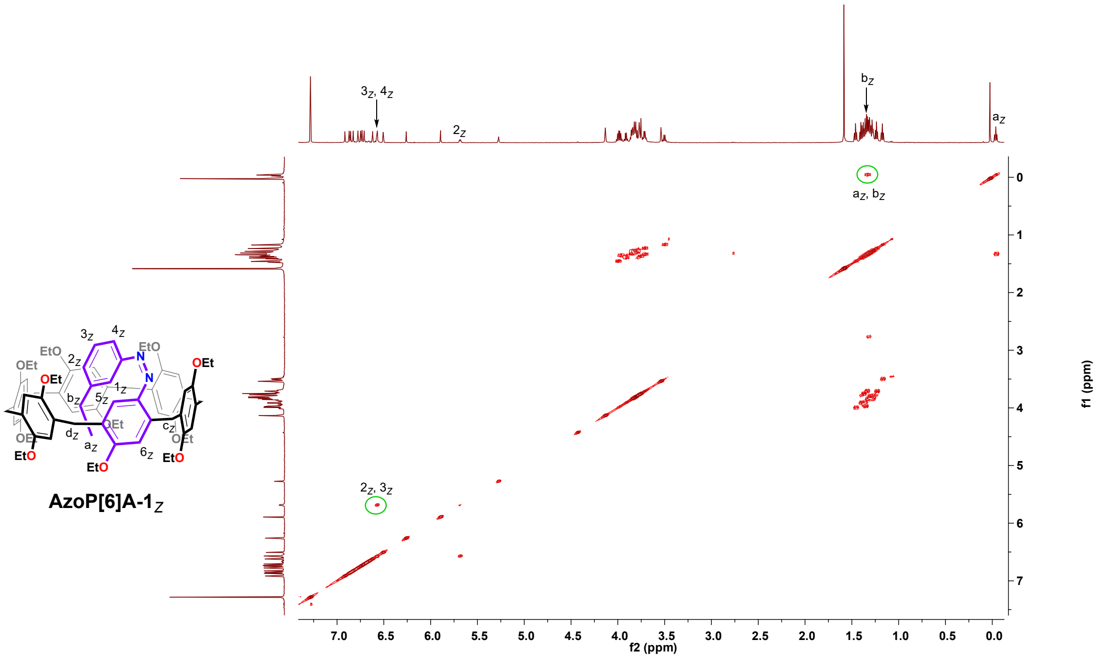

2D COSY-NMR spectrum (600 MHz, 4.0 mM, $CDCl_3$, 298K) for the 390 nm UV light irradiated solution of **AzoP[6]A-1**.

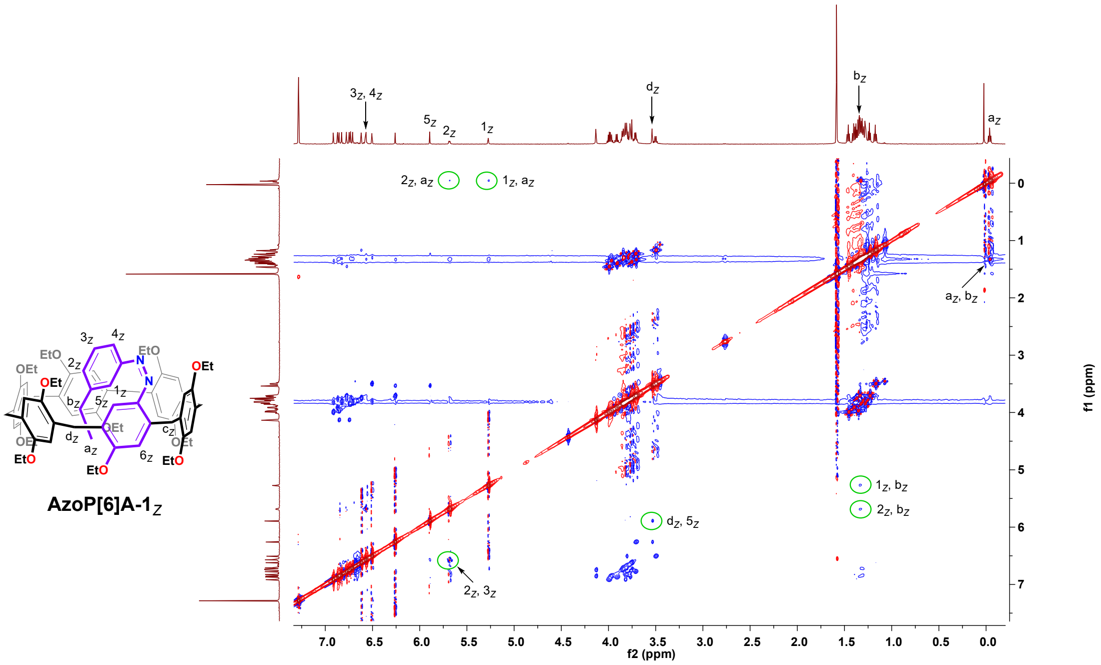

2D NOESY-NMR spectrum (600 MHz, 4.0 mM, $CDCl_3$, 298K) for the 390 nm UV light irradiated solution of **AzoP[6]A-1**.

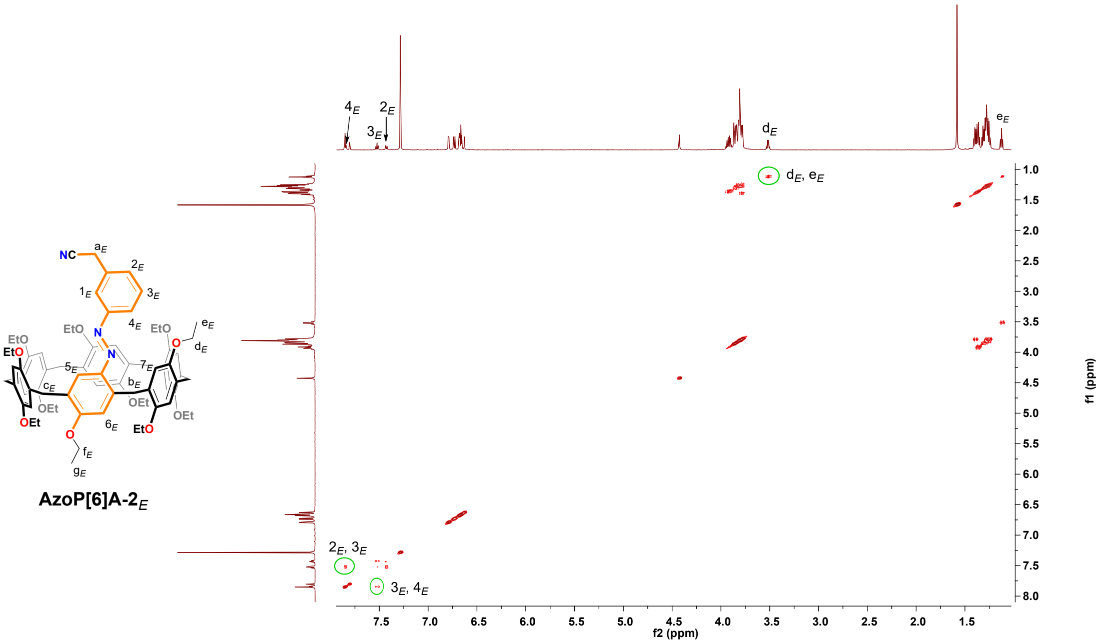

2D COSY-NMR spectrum (600 MHz, 4.0 mM, $CDCl_3$, 298K) for the solution of **AzoP[6]A-2**$_E$.

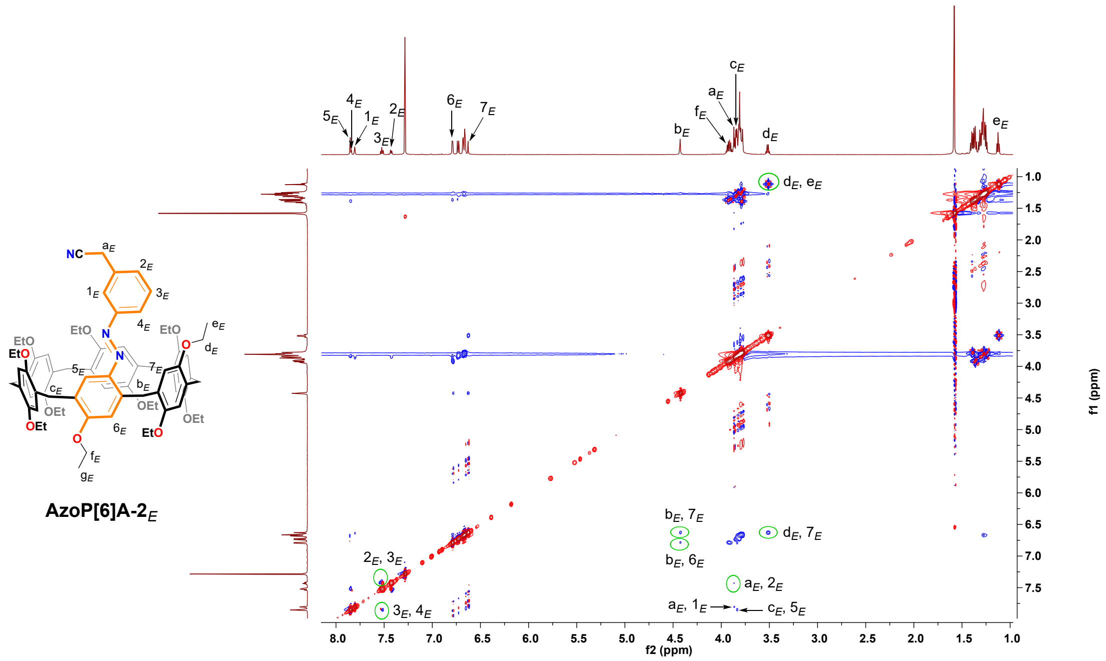

2D NOESY-NMR spectrum (600 MHz, 4.0 mM, $CDCl_3$, 298K) for the solution of **AzoP[6]A-2$_E$**.

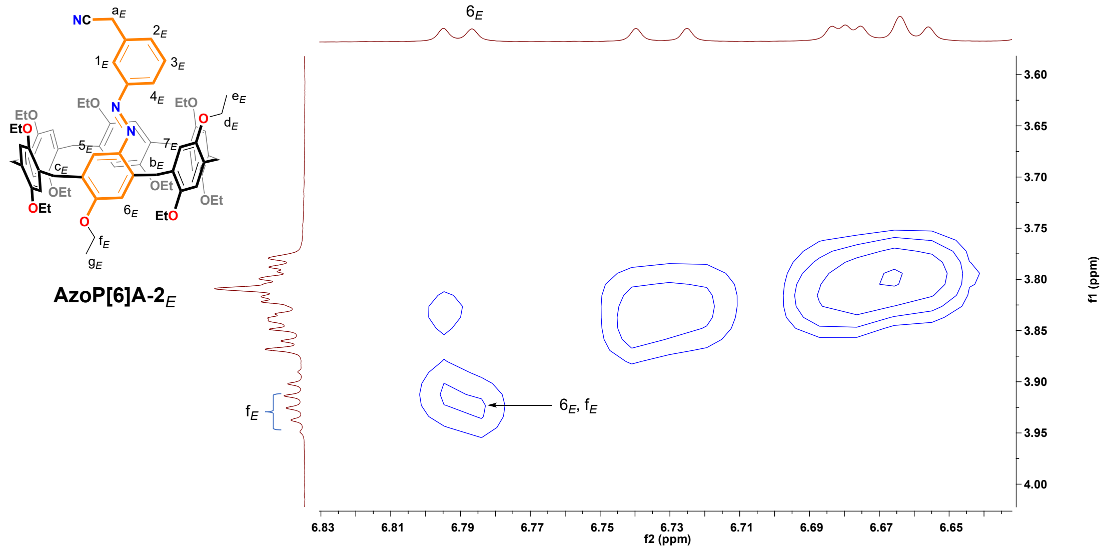

Partial 2D NOESY-NMR spectrum (600 MHz, 4.0 mM, $CDCl_3$, 298K) for the solution of **AzoP[6]A-2$_E$**.

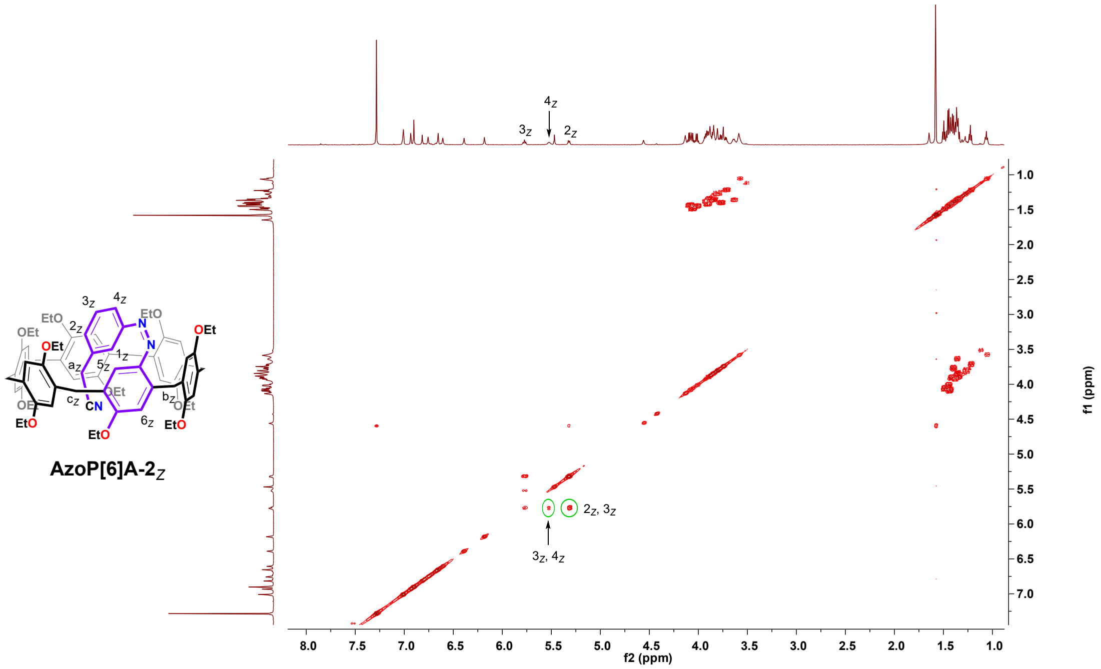

2D COSY-NMR spectrum (600 MHz, 4.0 mM, $CDCl_3$, 298K) for the 390 nm UV light irradiated solution of **AzoP[6]A-2**.

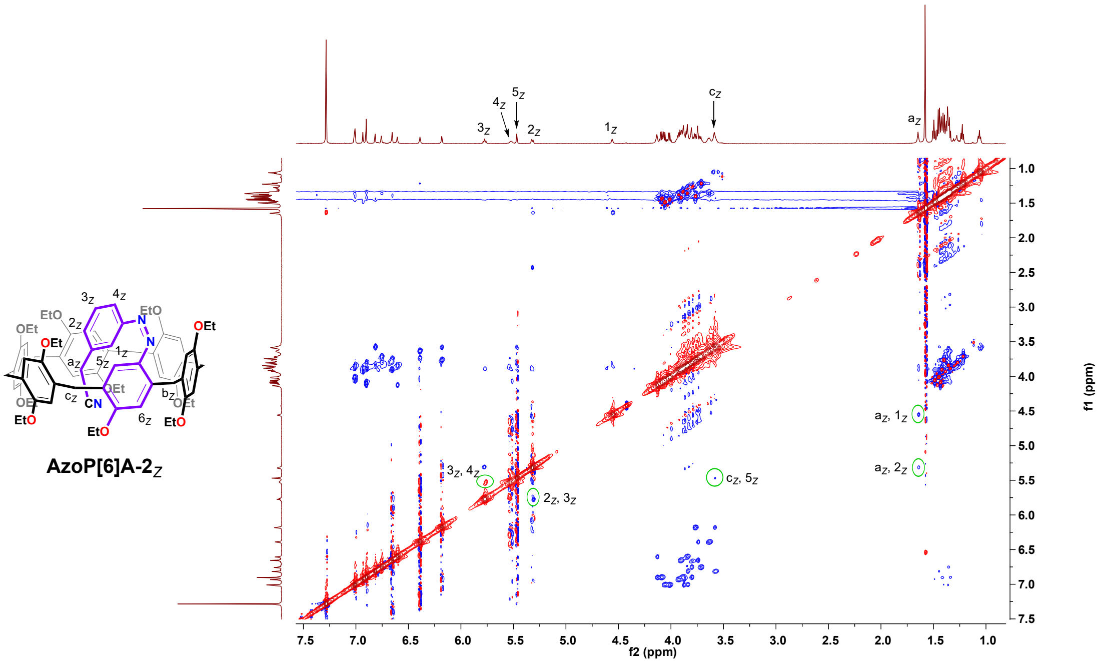

2D NOESY-NMR spectrum (600 MHz, 4.0 mM, $CDCl_3$, 298K) for the 390 nm UV light irradiated solution of **AzoP[6]A-2**.

References

[S1] C. Han, D. Zhao, Z. Lü, F. Zhan, L. Zhang, S. Dong and L. Jin, *Eur. J. Org. Chem.,* 2019, **2019**, 2508-2512.

[S2] H. Song, J. Wei, Z. Wang, Y. Liu, S. Zhao, X. Cai, Y. Xiao, L. Yang, P. Bai, L. Fang, F. Yang, S. Zheng, W. Zhang, J. Pan and C. Xu, *ACS Catal.*, 2024, **14**, 12372-12384.

[S3] M. R. Avei, S. Etezadi, B. Captain and A. E. Kaifer, *Commun. Chem.,* 2020, **3**, 117.

[S4] S. Fredrich, R. Göstl, M. Herder, L. Grubert and S. Hecht, *Angew. Chem., Int. Ed.,* 2016, **55**, 1208.

[S5] H. Xi, Z. Zhang, W. Zhang, M. Li, C. Lian, Q. Luo, H. Tian and W.-H. Zhu, *J. Am. Chem. Soc.,* 2019, **141**, 18467.

[S6] M. Montalti, A. Credi, L. Prodi and M. T. Gandolfi, *Handbook of Photochemistry*, Page 601-616. CRC Press (Taylor & Francis Group), 2006.

[S7] A. P. Glaze, H. G. Heller and J. Whittall, *J. Chem. Soc. Perk. Trans.,* 1992, **2**,591.

[S8] X. Shu, J. Fan, J. Li, X. Wang, W. Chen, X. Jia and C. Li, *Org. Biomol. Chem.,* 2012, **10**, 3393-3397.

[S9] X. Shu, W. Chen, D. Hou, Q. Meng, R. Zheng and C. Li, *Chem. Commun.,* 2014, **50**, 4820-4823.

[S10] X. Shu, S. Chen, J. Li, Z. Chen, L. Weng, X. Jia and C. Li, *Chem. Commun.,* 2012, **48**, 2967-2969.
